# Difluoroalkylation of Tertiary Amides and Lactams by an Iridium-Catalyzed Reductive Reformatsky Reaction

**DOI:** 10.1021/acs.orglett.2c00438

**Published:** 2022-03-08

**Authors:** Phillip Biallas, Ken Yamazaki, Darren J. Dixon

**Affiliations:** Chemistry Research Laboratory, Department of Chemistry, University of Oxford, 12 Mansfield Road, Oxford OX1 2JD, U.K.

## Abstract

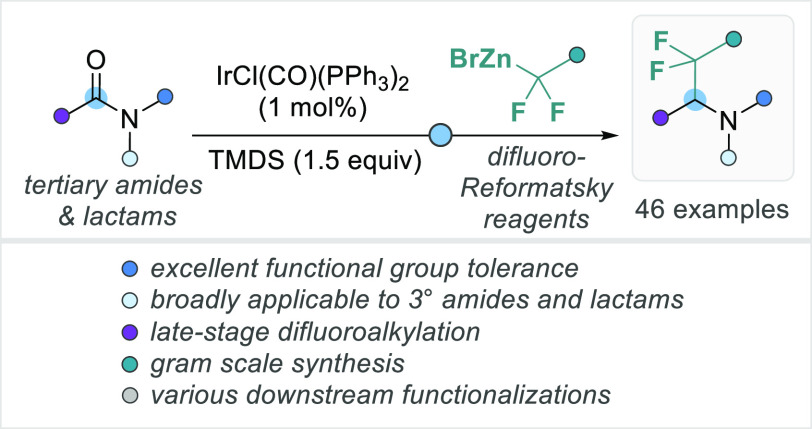

An iridium-catalyzed, reductive alkylation of abundant tertiary lactams and amides using 1–2 mol % of Vaska’s complex (IrCl(CO)(PPh_3_)_2_), tetramethyldisiloxane (TMDS), and difluoro-Reformatsky reagents (BrZnCF_2_R) for the general synthesis of medicinally relevant α-difluoroalkylated tertiary amines is described. A broad scope (46 examples), including *N*-aryl- and *N*-heteroaryl-substituted lactams, demonstrated an excellent functional group tolerance. Furthermore, late-stage drug functionalizations, a gram-scale synthesis, and common downstream transformations proved the potential synthetic relevance of this new methodology.

The incorporation of the *gem*-difluoromethylene (−CF_2_−) group, an oxygen bioisostere,^1^ into organic molecules has gained considerable attention in pharmaceutical and agrochemical research as well as in materials science, due to the unique influence of fluorine atoms on physical, chemical, and biological properties.^2^ More specifically, the β,β-difluoro-α-amino motif represents a key building block in many bioactive molecules, owing to the electronic influence of the fluorine atoms on the neighboring nitrogen center. The strong electron-withdrawing character of β-fluorine substitution on amines or nitrogen-containing heterocycles significantly lowers their basicity and p*K*_a_, which in turn influence critical parameters in medicinal lead optimization, such as physicochemical properties, binding affinities and absorption, distribution, metabolism, and excretion (ADME).^3^ The relevance of this structural motif in drug discovery is further exemplified by the large variety of β,β-difluoro-α-amino-containing pharmaceutical compounds such as gemcitabine,^4^ cedazuridine,^5^ eflornithine,^6^ GDC-0077,^7^ and glecaprevir^8^ (Scheme 1A). Therefore, the development of new concise and selective methods for the late-stage introduction of *gem*-difluoromethylene units onto nitrogen-containing scaffolds remains an attractive goal in synthetic chemistry.^9^

**Scheme 1:**
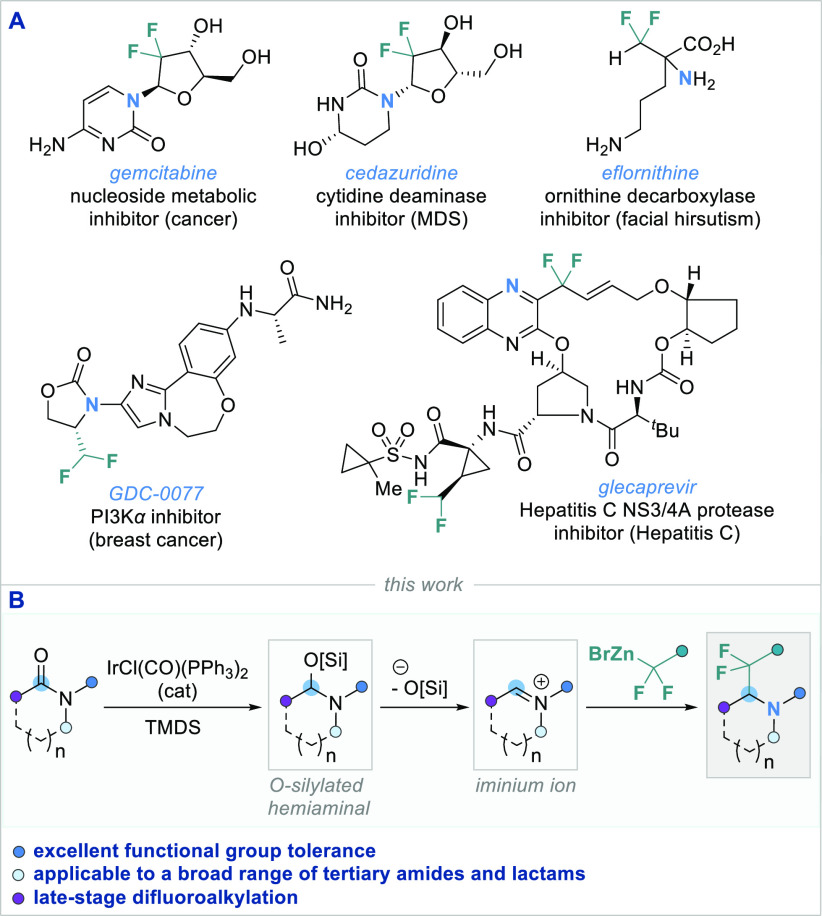
(A) Drug Molecules Containing the *gem-*Difluoro Motif and (B) Reductive Functionalization of Amides and Lactams by an Iridium-Catalyzed Reformatsky Reaction

In the past decade, several research groups have become involved in the challenging late-stage reductive C–C coupling of amides with organometallic reagents for the synthesis of α-functionalized amines.^10^ Stoichiometric approaches for the reductive functionalization of different amide classes, including lactams with various organometallic reagents, have been reported by Huang,^11^ Sato and Chida,^12^ and Chiba and our group.^13^ These methods employ DIBAL-H, Schwartz’s reagent (Cp_2_ZrHCl), triflic anhydride/metal hydride, or a NaH/NaI composite as the stoichiometric reductants. A highly chemoselective reductive functionalization of amides can be achieved by a transition-metal-catalyzed approach, as demonstrated by our group^14^ and others.^15^ Using catalytic amounts of Vaska’s complex (IrCl(CO)(PPh_3_)_2_) and 1,1,3,3-tetramethyldisiloxane (TMDS) led to the formation of metastable *O*-silylated hemiaminal intermediates, which are precursors to reactive iminium ions that can undergo subsequent nucleophilic functionalization.

Continuing our group’s ongoing efforts on reductive iridium-catalyzed C–C bond-forming reactions, we envisioned combining amide functionalization with commonly known difluoromethylene sources to form highly desirable and medicinally relevant α-difluoroalkylated amines (Scheme 1B). The ethoxycarbonyl-difluoromethyl (−CF_2_CO_2_Et) moiety is a versatile difluoromethylene source, due to its potential as a handle for further modifications into various functional groups.^16^ In addition to cross coupling,^17^ C–H functionalization,^16,17a,18^ and radical addition,^18a,19^ this difluoro-methylene-containing unit is traditionally introduced via nucleophilic attack of the corresponding difluoro-Reformatsky reagent (BrZnCF_2_CO_2_Et) on carbonyl groups, imines, or azodicarboxylates.^20^ This long-serving reagent with its efficacious reactivity toward various electrophiles caught our attention for its potential unprecedented deployment in a general late-stage amide functionalization approach, and herein we wish to report our findings.

*N*,*N*-Dimethyl-1-naphthamide **1a** was chosen as a model substrate for the reductive functionalization with difluoro-organozinc reagent **2a′**, which was freshly prepared from the corresponding ethyl bromodifluoroacetate (**2a**) and zinc in THF. We were very pleased that staged treatment of a toluene solution of **1a** with 1 mol % of Vaska’s complex, 2.0 equiv of TMDS, and 1.1 equiv of difluoro-organozinc reagent **2a′** gave the desired tertiary amine **3a** in promising 53% yield, alongside minor amounts of secondary alcohol **4** and overreduction product **5** (Scheme 2, entry 1). Increasing the equivalents of organozinc reagent **2a′** improved the yield of desired product **3a** slightly (Scheme 2, entry 2). More significantly, lowering the amount of TMDS to 1.5 equiv drastically reduced the rate of overreduction and allowed access to synthetically useful yields of functionalization product **3a** (Scheme 2, entry 3). Finally, changing the concentration of organozinc reagent **2a′** by dilution provided a 75% isolated yield (Scheme 2, entry 4). Further changes to the reaction conditions, such as using different solvent combinations, temperatures, or reaction times, did not have a positive effect on the reaction outcome (see SI for full optimization details).

**Scheme 2:**
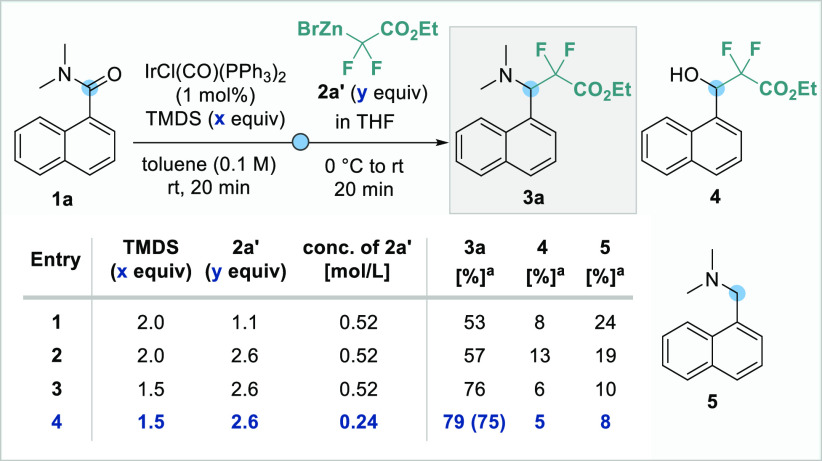
Reaction Optimization NMR yield using 1,3,5-trimethoxybenzene as an internal standard; isolated yield in parentheses.

With optimized conditions in hand, we then examined the reaction scope with respect to tertiary amides and lactams **1** (Scheme 3). Satisfyingly, several *N*,*N*-dimethyl-benzamides **1a**–**1f** with electron-deficient and electron-rich substituents in *ortho* or *para* positions, as well as furan substrate **1g**, could be successfully converted into the corresponding difluoromethylated tertiary amines **3b**–**3g** in good isolated yields (62–84%). Pyrrolidine-, piperidine-, morpholine-, azepane-, and azocane-derived amides **3h**–**3o** were reductively functionalized in good to excellent yields (69–98%) while demonstrating tolerance to various substituents such as boronic ester, acetal, iodo, or nitro groups. *N*,*N*-Dibenzylamide **1p**, *N*,*N*-benzylethylamide **1q**, and anilide **1r** were successfully employed to furnish the desired products **3p**–**3r** in 64–87% yields. However, increased amounts of TMDS (2.5 equiv) and Vaska’s complex (2 mol %) were used to force the slow reduction step of these more challenging substrates to full conversion. Anilide **1s**, bearing an ethyl ester moiety, was converted into amine **3s** in the same way, albeit in a diminished 56% yield. Weinreb amide **1t** reacted smoothly to product **3t** in 91% yield, while α,β-unsaturated amides gave difluoro products **3u** and **3v** in moderate 34% and 58% yields, which is due to competing conjugate addition. Furthermore, aliphatic amides **1w** and **1x** underwent reductive functionalization in 73% and 68% yields. Encouraged by these results, we also envisioned including lactams in the substrate scope. Five- and six-membered lactams **1y** and **1z** gave the corresponding difluoroalkylated pyrrolidine **3y** and piperidine **3z** in moderate 60% and 42% yields, despite slightly reoptimized reaction conditions. For these products, we observed significantly higher yields by reducing the time between the addition of TMDS and the organozinc bromides and by changing the solvent from toluene to THF or 2-methyl-THF.^21^ Very pleasingly, *N*-benzyl-, *N*-phenyl-, *N*-pyridyl- and *N*-pyrimidyl-substituted difluoroalkylated azepanes **3aa**–**3ae** were obtained in overall good yields (57–81%) under the standard reaction conditions. This method was also successfully applied to the late-stage functionalization of the active pharmaceutical ingredients (APIs) piperine (**1af**), napropamide (**1ag**), acetyletamivan (**1ah**), and CX-546 (**1ai**). The corresponding difluorinated drug derivates **3af**–**3ai** were isolated in good yields (56–80%), highlighting the potential application of this method for pharmaceutical drug discovery and lead structure optimization. No C–C coupling was observed using secondary amides, and mainly aldehyde formation was witnessed after aqueous workup.

**Scheme 3:**
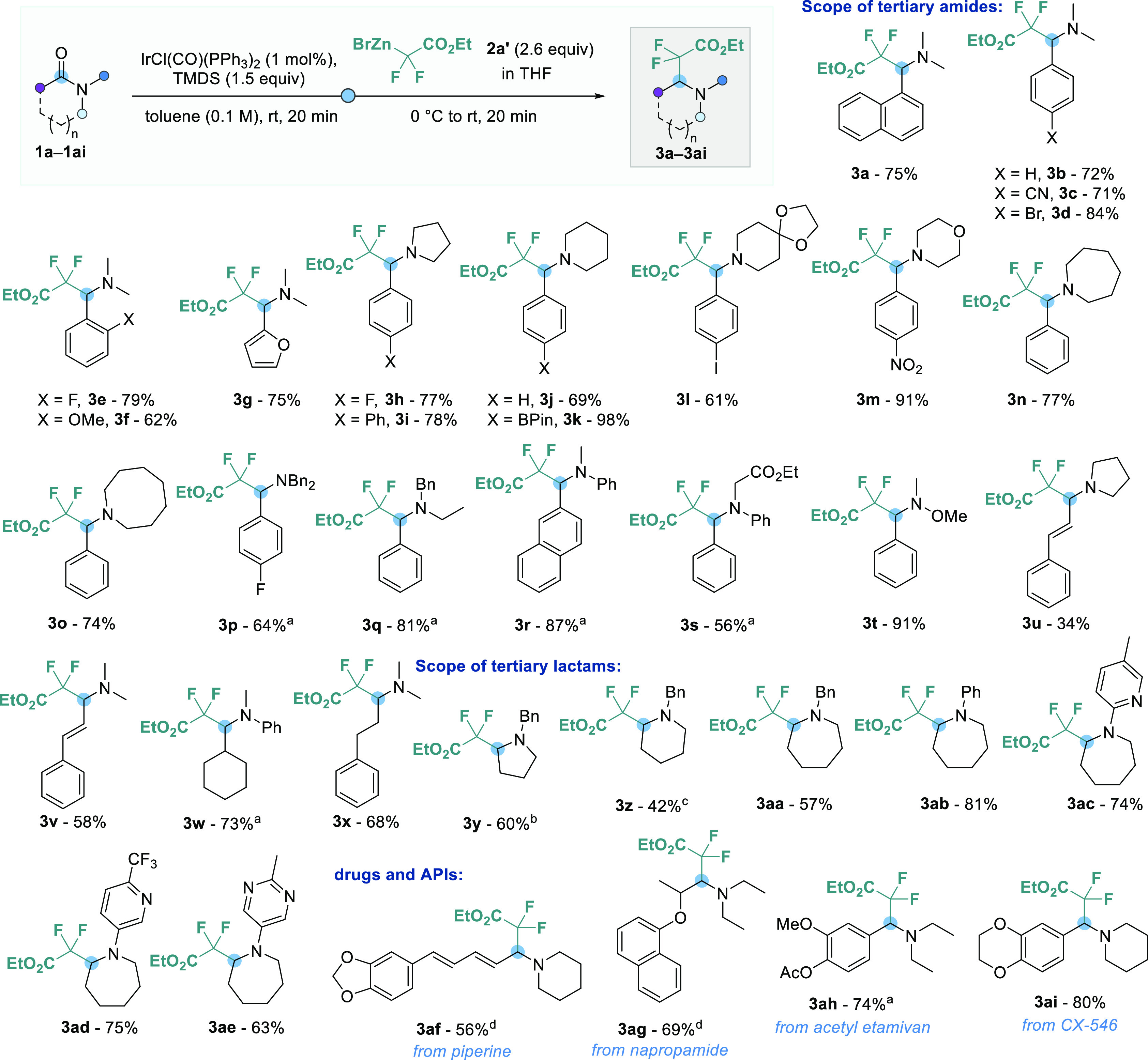
Reaction Scope of Tertiary Amides and Lactams 2.5 equiv of TMDS and 2 mol % of IrCl(CO)(PPh_3_)_2_ were used. 2-Methyl-THF was used as the solvent in the first step and stirred for 2 min. THF was used as the solvent in the first step. 2.5 equiv of TMDS and 2 mol % of IrCl(CO)(PPh_3_)_2_ were used, and the first step was stirred for 1 h. Standard conditions: amide or lactam **1** (0.15 mmol), IrCl(CO)(PPh_3_)_2_ (1 mol %), TMDS (0.23 mmol), toluene (1.50 mL), and **2a′** (0.40 mmol) in THF (1.63 mL); isolated yields are given.

Next, we assessed the scope of the difluoro-organozinc reagents **2′** and were again pleased to find that azepan-1-yl(phenyl)methanone (**1n**) could be readily functionalized with benzyl, trimethylsilyethyl, and isopropyl difluoroacetates **2b′**–**2d′** to form **3aj**–**3al** in good yields (63–85%) (Scheme 4). Vaska’s complex (2 mol %) and 2.5 equiv of TMDS were used to ensure that starting amide **1n** was fully converted into the silylated hemiaminal intermediate before adding the nucleophile. Employing l-menthol- and glycerol-derived difluoroacetates **2e′** and **2f′**, products **3am** and **3an** were isolated in 49% and 64% yields as 1.2:1 and 1:1 mixtures of diastereomers, respectively. Sterically demanding benzhydryl difluoroacetate **2g′** could be introduced efficiently in 74% yield to give tertiary amine **3ao**. Notably, difluoroacetamide-containing zinc bromides **2h′** and **2i′** could also be used under the same reaction conditions to furnish amines **3ap** and **3aq** in near quantitative yields. Using morpholine-derived difluoroacetamide **2j′**, **3ar** was obtained in good yield (63%). Further reduction of the difluoroacetamide moiety in these products was not observed under the reported reaction conditions, which can be explained by the active iridium catalyst being quenched by the organozinc bromides upon addition. Highlighting lactams as suitable feedstock compounds, the reductive functionalization of **1ab** with benzyl difluoroacetate **2b′** and difluoroacetamide **2h′** gave the C2-difluoroalkylated saturated nitrogen-containing heterocyclic amines **3as** and **3at** in 42% and 53% yields, respectively.

**Scheme 4:**
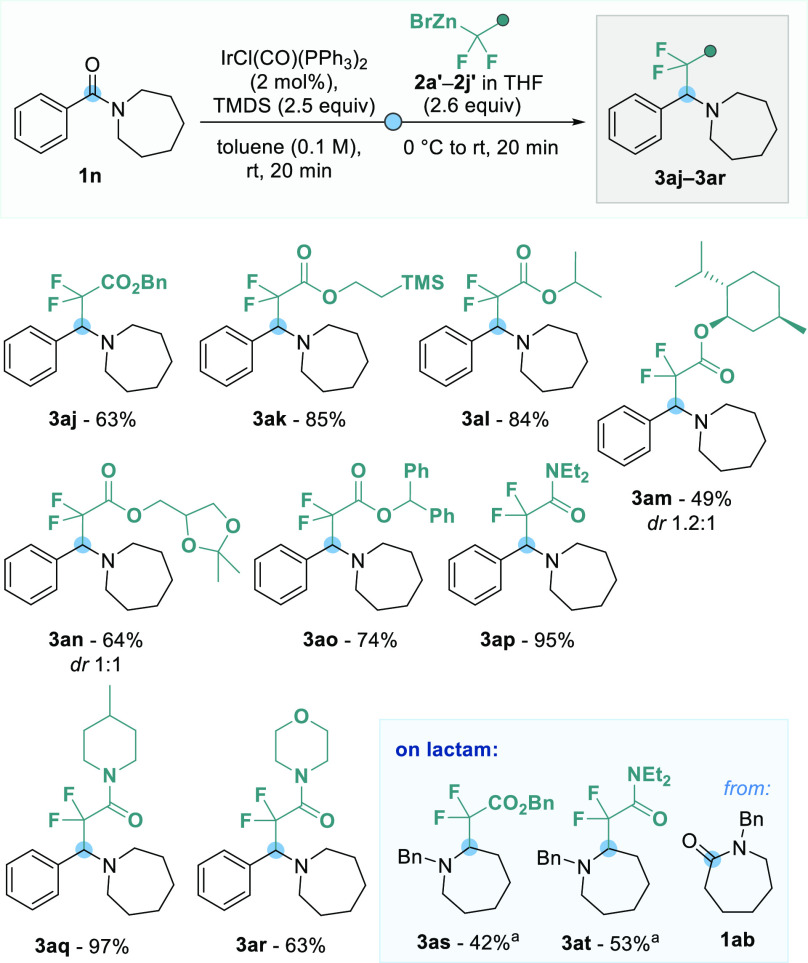
Reaction Scope of Difluoro-Organozinc Reagents Lactam **1ab** (0.15 mmol), 1.5 equiv of TMDS, and 1 mol % of IrCl(CO)(PPh_3_)_2_ were used. Standard conditions: amide **1n** (0.15 mmol), IrCl(CO)(PPh_3_)_2_ (2 mol %), TMDS (0.38 mmol), toluene (1.50 mL), **2′** (0.40 mmol) in THF; isolated yields are given.

To showcase the synthetic utility of this methodology, we performed a gram-scale reductive difluoroalkylation of amide **1b**, generating tertiary amine **3b** in a 67% (1.15 g, 4.47 mmol) yield (Scheme 5), which was comparable to the small-scale reaction. Identifying the ester moiety in **3b** as a useful handle for downstream derivatizations, we synthesized several CF_2_-containing compounds **6**–**10** by standard organic procedures. Primary alcohol **6** was obtained in 81% yield by reduction with sodium borohydride. Addition of a methanolic ammonia solution gave corresponding primary amide **7** in 85% yield. Tertiary alcohol **8** was formed in 61% yield, using 2.1 equiv of Grignard reagent. Saponification and subsequent acidification furnished carboxylic acid **9** in quantitative yield. Finally, enol ether **10** was installed in 42% yield by employing the Tebbe reagent under basic reaction conditions.

**Scheme 5:**
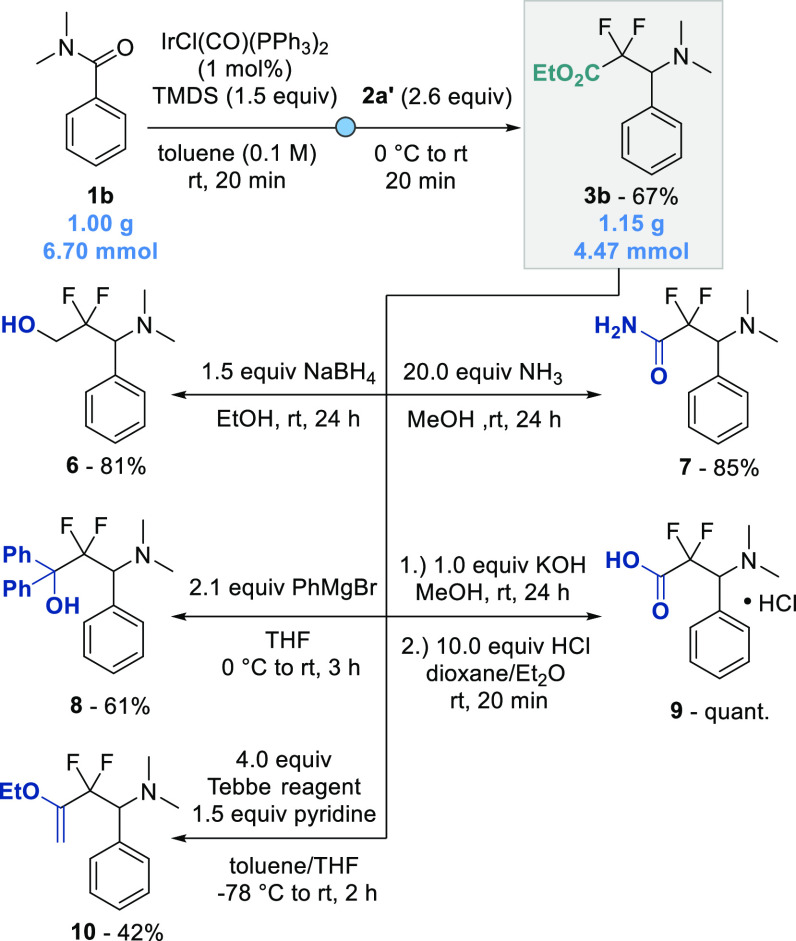
Gram-Scale Reaction and Downstream Functionalization Isolated yields are given.

In conclusion, a broadly applicable and efficient method for the synthesis of acyclic and cyclic α-difluoroalkylated tertiary amines with good overall yields has been developed. The mild iridium-catalyzed reductive difluoroalkylation shows excellent functional group tolerance with respect to both coupling partners: amides/lactams and organozinc reagents, which are among other things highlighted by the late-stage derivatization of four drug molecules. Furthermore, the reaction was readily performed on a gram scale without a significant loss in yield, and several CF_2_-containing derivates were made by common downstream transformations, altogether demonstrating the potential utility of the method developed herein as a useful tool in current and future drug discovery programs.

## Supporting Information Available

General information, full optimization details, experimental procedures, compound characterization, and NMR spectra. (PDF)
