# Supplementary material for: Difluoroalkylation of Tertiary Amides and Lactams by an Iridium-Catalyzed Reductive Reformatsky Reaction
Source: Org Lett. 2022 Mar 8;24(10):2002–7. doi: 10.1021/acs.orglett.2c00438 (PMC9082613; doi:10.1021/acs.orglett.2c00438)
Supplement: Supplementary file 1 — ol2c00438_si_001.pdf [file ol2c00438_si_001.pdf]

Supporting information

for

Difluoroalkylation of tertiary amides and lactams by an iridium-catalyzed reductive Reformatsky reaction

Phillip Biallas, Ken Yamazaki, Darren J. Dixon*

Department of Chemistry, University of Oxford, Chemistry Research Laboratory, 12 Mansfield Road, Oxford, OX1 3TA, United Kingdom

*darren.dixon@chem.ox.ac.uk

Contents

1. General information 1

2. Full Optimization details 2

3. General procedures 4

4. Synthesis and characterization of starting materials 7

5. Synthesis and characterization of products 12

6. NMR Spectra 40

7. References 126

1. General information

Proton, carbon and fluorine NMR spectra were recorded on *Bruker AVIII HD* 400 MHz (^{1}H NMR at 400 MHz, ^{13}C NMR at 101 MHz, and ^{19}F NMR at 377 MHz) or *Bruker AVII* 500 MHz (^{1}H NMR at 500 MHz, ^{13}C NMR at 126 MHz). Chemical shifts for protons are reported in parts per million downfield from $Si(CH_3)_4$ and are referenced to residual protium in the deuterated solvent ($CHCl_3$ at 7.26 ppm, DMSO at 3.31 (H_2O), 2.50 depending on solvent used). Chemical shifts for fluorines are reported in parts per million downfield from $CFCl_3$. NMR data are presented in the following format: chemical shift (multiplicity [app = apparent, br = broad, d = doublet, t = triplet, q = quartet, dd = doublet of doublets), dt = doublet of triplets), dq = doublet of quartets), ddd = doublet of doublet of doublets), m = multiplet], coupling constant [in Hz], number of equivalent nuclei by integration).

High-resolution mass spectra (ESI, APCI) were performed on a *Thermo Orbitrap Exactive MS* or an *Agilent 7200 Accurate Mass Q-TOF GC-MS* (EI). Infrared spectra were recorded on a *Bruker Tensor 27* FT-IR spectrometer as a thin film. Only selected maximum absorbances are reported (in vmax (cm^{-1})). Melting points were recorded using a Leica Galen III hot-stage microscope apparatus and are reported uncorrected in degrees Celcius (°C). Analytical thin-layer chromatography (TLC) was performed on *Merck* silica gel 60 F254 plates and visualised with UV light (254 or 365 nm), and/or $KMnO_4$. Silica gel column chromatography was performed using 60 Å silica gel 40-63 µm purchased from *Sigma-Aldrich*.

All reactions were carried out under an Argon atmosphere unless stated otherwise and performed using reagents obtained from *Sigma-Aldrich*, *Acros Organics*, *Alfa Aesar*, *STREM* or *Fluorochem* without further purification. Inert atmosphere techniques, such as Schlenk technique, were used for the handling of air/moisture sensitive reagents. Toluene (anhydrous), 2-Methyltetrahydrofuran (anhydrous) and DCM (anhydrous) were used as supplied. Tetrahydrofuran was used from a Solvent Purification System; dried by filtration through activated alumina (powder ~150 mesh, pore size 58 Å, basic, Sigma-Aldrich), degassed with Argon and stored over 3Å molecular sieves. Deuterated solvents were used as supplied. An oil bath was used for reactions requiring heating. Temperatures quoted are external. Solvents were removed under reduced pressure using *Büchi* Rotavapor apparatus. Tertiary amides and lactams **1a** - **1aa** were synthesized following previous literature reports and spectral data match those reported. [1-11] Bromodifluoroacetamides **2h** and **2j** were synthesized following a previous literature report and spectral data match those reported. [12]

2. Full Optimization details

Procedure for the optimization of the reductive coupling:

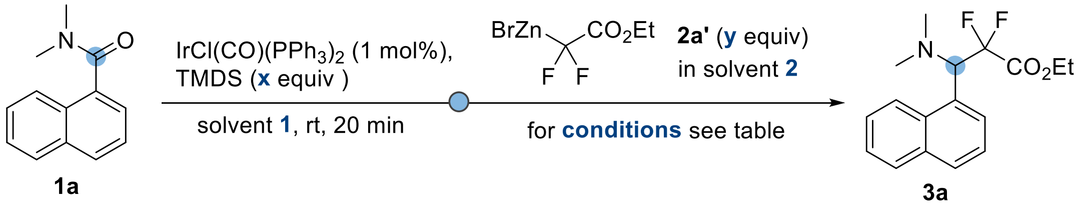

Step 1, formation of the organozinc reagent: An oven-dried 10 mL round bottom-flask, equipped with a magnetic stirring bar and a septum, was three times vacuumed and backfilled with Argon. Zinc powder (144 mg, 2.21 mmol, 1.5 equiv) was added and suspended in anhydrous solvent **2**. TMSCl (48 mg, 56 µL, 0.44 mmol, 30 mol%) was added and the reaction mixture was stirred at room temperature for 15 min. Then ethyl bromodifluoroacetate **2a** (299 mg, 189 µL, 1.47 mmol, 1.0 equiv) was added dropwise to the solution and significant exotherm was usually observed after the addition. The mixture was allowed to cool to room temperature by stirring an additional 20 min and was directly used in the next step. The concentration of the corresponding organozinc reagent **2a'** was determined according to a literature report by titration with I_2.[13]

Step 2, reductive functionalization of amide 1a: An oven-dried 10 mL round bottom-flask, equipped with a magnetic stirring bar and a septum, was three times vacuumed and backfilled with Argon. The flask was then initially charged with Vaska's catalyst (1.2 mg, 1.51 µmol, 1 mol%), *N,N*-dimethyl-1-naphthamide **1a** (30.0 mg, 0.15 mmol, 1.0 equiv) and anhydrous solvent **1**. TMDS (**X** equiv) was added and the resulting mixture was stirred for 20 min. Freshly prepared organozinc solution **2a'** (**Y** equiv) was added, followed by additional stirring (for detailed **conditions** see table). A 1,3,5-trimethoxybenzene solution (0.50 mL, 0.10 M in toluene, 50.2 µmol, 0.33 equiv) was added and the reaction mixture was quenched with a saturated, aqueous NH_4Cl-solution (10 mL) and extracted with DCM (3 x 5 mL). The combined organic phases were washed with brine (10 mL), dried over Na_2SO_4, filtered and concentrated *in vacuo*. The yield of tertiary amide **3a** was then determined by ^{1}H NMR with 1,3,5-trimethoxybenzene as an internal standard.

Optimization table:

Entry	Solvent 1	Solvent 2	TMDS (x equiv)	2a' (y equiv)	conc. of 1a [mol/L]	conc. of 2a' [mol/L]	conditions	Yield 3a [%]
**1**	toluene	THF	2.0	1.1	0.10	0.52	0 °C to rt, 20 min	53
**2**	toluene	THF	2.0	2.6	0.10	0.52	0 °C to rt, 20 min	57
**3**	THF	THF	2.0	2.6	0.10	0.52	0 °C to rt, 20 min	40
**4**	DCM	THF	2.0	2.6	0.10	0.52	0 °C to rt, 20 min	55
**5**	toluene	toluene	2.0	2.6	0.10	0.52	0 °C to rt, 20 min	0
**6**	toluene	THF	1.2	2.6	0.10	0.52	0 °C to rt, 20 min	30
**7**	toluene	THF	1.5	2.6	0.10	0.52	0 °C to rt, 20 min	76
**8**	toluene	THF	1.5	2.0	0.10	0.52	0 °C to rt, 20 min	76
**9**	toluene	THF	1.5	3.0	0.10	0.52	0 °C to rt, 20 min	76
**10**	toluene	THF	1.5	3.5	0.10	0.52	0 °C to rt, 20 min	56
**11**	toluene	THF	1.5	1.5	0.10	0.52	0 °C to rt, 20 min	43
**12**	toluene	THF	1.5	1.2	0.10	0.52	0 °C to rt, 20 min	51
**13**	toluene	THF	1.5	2.6	0.20	0.52	0 °C to rt, 20 min	68
**14**	toluene	THF	1.5	2.6	0.40	0.52	0 °C to rt, 20 min	64
**15**	toluene	THF	1.5	2.6	0.05	0.52	0 °C to rt, 20 min	69
**16**	toluene	THF	1.5	2.6	0.10	0.52	0 °C, 20 min	62
**17**	toluene	THF	1.5	2.6	0.10	0.52	rt, 20 min	73
**18**	toluene	THF	1.5	2.6	0.10	0.52	rt to 40 °C, 20 min	68
**19**	toluene	THF	1.5	2.6	0.10	0.52	0 °C to rt, 10 min	73
**20**	toluene	THF	1.5	2.6	0.10	0.52	0 °C to rt, 60 min	70
**21**	toluene	THF	1.5	2.6	0.10	0.52	0 °C to rt, 24 h	75
**22**	toluene	THF	1.5	2.6	0.10	0.48	0 °C to rt, 20 min	73
**23**	**toluene**	**THF**	**1.5**	**2.6**	**0.10**	**0.24**	**0 °C to rt, 20 min**	**79**
**24**	toluene	THF	1.5	2.6	0.10	0.09	0 °C to rt, 20 min	70

3. General procedures

Procedure A: Synthesis of (hetero)aromatic substituted lactams

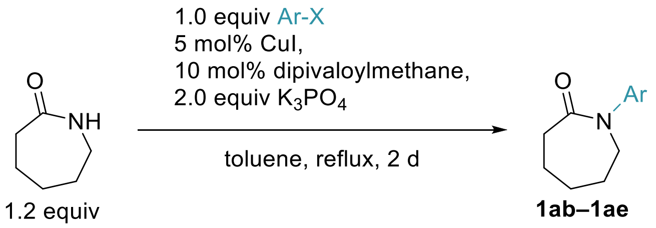

An oven-dried microwave tube was initially charged with ε-caprolactam (1.2 equiv), CuI (5 mol%), K_3PO_4 (2.0 equiv) and aryl bromide or aryl iodide (1.0 equiv). The tube was sealed and three times vacuumed and backfilled with Argon. Dipivaloylmethane (10 mol%) and anhydrous toluene (1.0 M) were added *via* syringe and the reaction mixture was allowed to stir under reflux for 2 d. Then the mixture was allowed to cool to room temperature, diluted with DCM and washed with water. The aqueous phase was extracted with DCM and the combined organic phases were washed with brine (50 mL), dried over Na_2SO_4, filtered and concentrated *in vacuo*. The crude residue was purified *via* silica gel column chromatography to furnish the corresponding lactams **1ab**–**1ae**.

Procedure B: Synthesis of bromodifluoroacetates

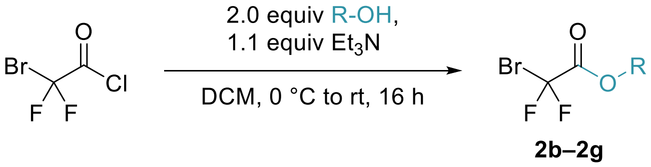

An oven-dried round bottom-flask, equipped with a magnetic stirring bar and a septum, was three times vacuumed and backfilled with Argon. Anhydrous DCM (0.2 M) and 2-bromo-2,2-difluoroacetyl chloride (1.0 equiv) were added and the solution was cooled to 0 °C. The corresponding alcohol (2.0 equiv) and triethylamine (1.1 equiv) were then added dropwise over 20 min. The mixture was allowed to warm up to room temperature and stirring was continued for 16 h. The crude reaction mixture was diluted with DCM and washed with water. The aqueous phase was extracted with DCM and the combined organic phases were washed with brine (50 mL), dried over Na_2SO_4, filtered, concentrated *in vacuo* and the residue was purified *via* silica gel column chromatography to give bromodifluoroacetates **2b**–**2g**.

Procedure C: Scope of amides and lactams

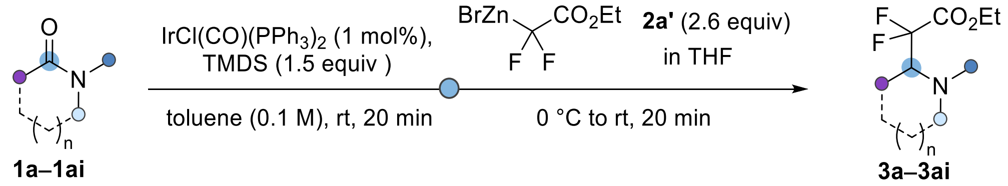

Step 1, formation of the organozinc reagent: An oven-dried 10 mL round bottom-flask, equipped with a magnetic stirring bar and a septum, was three times vacuumed and backfilled with Argon. Zinc powder (144 mg, 2.21 mmol, 1.5 equiv) was added and suspended in 2.5 mL anhydrous THF (0.6 M). TMSCl (48 mg, 56 µL, 0.44 mmol, 30 mol%) was added and the reaction mixture was stirred at room temperature for 15 min. Then ethyl bromodifluoroacetate **2a** (299 mg, 189 µL, 1.47 mmol, 1.0 equiv) was added dropwise to the solution and significant exotherm was usually observed after the addition. The mixture was allowed to cool to room temperature by stirring an additional 20 min and was directly used in the next step. The concentration of the corresponding organozinc reagent **2a'** was determined according to a literature report by titration with I_2 to be 0.24 mol/L.[13]

Step 2, reductive functionalization of amides: An oven-dried 10 mL round bottom-flask, equipped with a magnetic stirring bar and a septum, was three times vacuumed and backfilled with Argon. The flask was then initially charged with Vaska's catalyst (1 - 2 mol%), amide or lactam **1** (0.15 mmol, 1.0 equiv) and 1.5 mL anhydrous toluene (0.1 M). TMDS (1.5 - 2.5 equiv) was added and the resulting mixture was stirred for 20 min. The solution was cooled to 0 °C and the freshly prepared organozinc solution **2a'** (1.63 mL, 0.24 M in THF, 0.40 mmol, 2.6 equiv) was added, followed by an additional stirring for 20 min at room temperature. Then the reaction mixture was quenched with a saturated, aqueous NH_4Cl-solution (10 mL) and extracted with DCM (3 x 5 mL). The combined organic phases were washed with brine (10 mL), dried over Na_2SO_4, filtered and concentrated *in vacuo*. The crude residue was purified *via* silica gel column chromatography to furnish the corresponding tertiary amines **3a**–**3ai**.

Procedure D: Scope of difluoro-organozinc bromides

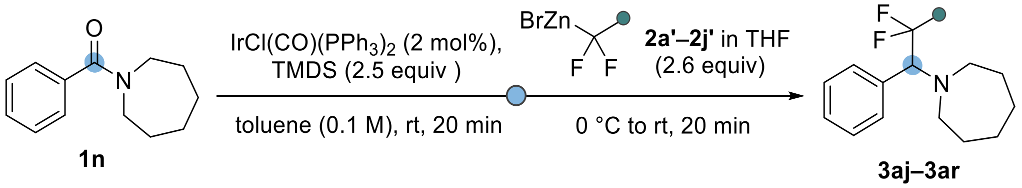

Step 1, formation of the organozinc reagent: An oven-dried 10 mL round bottom-flask, equipped with a magnetic stirring bar and a septum, was three times vacuumed and backfilled with Argon. Zinc powder (192 mg, 2.94 mmol, 1.5 equiv) was added and suspended in 3.3 mL anhydrous THF (0.6 M). TMSCl (63.9 mg, 75 µL, 0.59 mmol, 30 mol%) was added and the reaction mixture was stirred at room temperature for 15 min. Then bromodifluoroacetate or -acetamide **2** (1.96 mmol, 1.0 equiv) was added dropwise to the solution and significant exotherm was usually observed after the addition. The mixture was allowed to cool to room temperature by stirring an additional 20 min and was directly used in the next step. The concentration of the corresponding organozinc reagent **2’** was determined according to a literature report by titration with I_2.[13]

Step 2, reductive functionalization of amide 1n: An oven-dried 10 mL round bottom-flask, equipped with a magnetic stirring bar and a septum, was three times vacuumed and backfilled with Argon. The flask was then initially charged with Vaska’s catalyst (2.4 mg, 3.05 µmol, 2 mol%), azepan-1-yl(phenyl)methanone (**1n**) (31.0 mg, 0.15 mmol, 1.0 equiv) and 1.5 mL anhydrous toluene (0.1 M). TMDS (51.2 mg, 67 µL, 0.38 mmol, 2.5 equiv) was added and the resulting mixture was stirred for 20 min. The solution was cooled to 0 °C and the freshly prepared organozinc solution **2’** (2.6 equiv) was added, followed by an additional stirring for 20 min at room temperature. Then the reaction mixture was quenched with a saturated, aqueous NH_4Cl-solution (10 mL) and extracted with DCM (3 x 5 mL). The combined organic phases were washed with brine (10 mL), dried over Na_2SO_4, filtered and concentrated *in vacuo*. The crude residue was purified *via* silica gel column chromatography to furnish the corresponding tertiary amines **3aj**–**3ar**.

4. Synthesis and characterization of starting materials

1-phenylazepan-2-one (1ab)

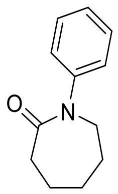

Procedure A was followed using ε-caprolactam (1.00 g, 8.84 mmol, 1.2 equiv), CuI (70.1 mg, 0.37 mmol, 5 mol%), K_3PO_4 (3.13 g, 14.7 mmol, 2.0 equiv) and iodobenzene (1.50 g, 0.82 mL, 7.36 mmol, 1.0 equiv). Silica gel column chromatography (EA/P 1:1 → 4:1) gave the desired lactam **1ab** as a colorless solid, 50% (700 mg, 3.70 mmol). NMR spectra matched those reported in literature.[14] **^{1}H NMR** (400 MHz, $CDCl_3$): δ 7.41 – 7.32 (m, 2H), 7.25 – 7.18 (m, 3H), 3.75 (s, 2H), 2.75 – 2.66 (m, 2H), 1.89 – 1.76 (m, 6H). **^{13}C NMR** (101 MHz, $CDCl_3$): δ 175.6, 144.7, 129.2, 126.5, 126.4, 53.2, 37.9, 30.0, 29.1, 23.7.

1-(5-methylpyridin-2-yl)azepan-2-one (1ac)

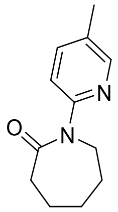

Procedure A was followed using ε-caprolactam (1.00 g, 8.84 mmol, 1.2 equiv), CuI (70.1 mg, 0.37 mmol, 5 mol%), K_3PO_4 (3.13 g, 14.7 mmol, 2.0 equiv) and 2-bromo-5-methylpyridine (1.27 g, 7.36 mmol, 1.0 equiv). Silica gel column chromatography (EA/P 1:1 → 9:1) gave the desired lactam **1ac** as a brown oil, 20% (305 mg, 1.49 mmol). **IR** (thin film): ν_{max} (cm^{-1}) = 2927, 2857, 1662, 1478, 1404, 1379, 1267, 1213, 1191, 983, 827. **^{1}H NMR** (400 MHz, $CDCl_3$): δ 8.25 – 8.19 (m, 1H), 7.66 – 7.40 (m, 2H), 4.04 – 3.97 (m, 2H), 2.76 – 2.66 (m, 2H), 2.28 (s, 3H), 1.89 – 1.74 (m, 6H). **^{13}C NMR** (101 MHz, $CDCl_3$): δ 175.9, 152.9, 148.1, 137.7, 130.0, 120.5, 49.0, 38.6, 29.8, 29.0, 23.7, 17.9. **HRMS** (ESI): m/z calculated for $C_{12}H_{16}N_2O$ requires 205.1335 for $[M+H]^+$, found 205.1337.

1-(6-(trifluoromethyl)pyridin-3-yl)azepan-2-one (1ad)

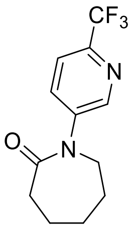

Procedure A was followed using ε-caprolactam (400 mg, 3.53 mmol, 1.2 equiv), CuI (28.1 mg, 0.15 mmol, 5 mol%), K_3PO_4 (1.25 g, 5.89 mmol, 2.0 equiv) and 2-bromo-5-(trifluoromethyl)pyridine (666 mg, 2.95 mmol, 1.0 equiv). Silica gel column chromatography (EA/P 7:3 → 9:1) gave the desired lactam **1ad** as a colorless solid, 26% (200 mg, 0.77 mmol). **m.p.**: 98–102 °C. **IR** (thin film): ν_{max} (cm^{-1}) = 2934, 2861, 1661, 1441, 1338, 1131, 1083, 838, 701. **^{1}H NMR** (400 MHz, $CDCl_3$): δ 8.62 (d, J = 2.4 Hz, 1H), 7.79 (dd, J = 8.5, 2.5 Hz, 1H), 7.67 (dd, J = 8.4, 0.7 Hz, 1H), 3.87 – 3.79 (m, 2H), 2.78 – 2.69 (m, 2H), 1.92 – 1.79 (m, 6H). **^{13}C NMR** (101 MHz, $CDCl_3$): δ 175.8, 147.1, 145.1 (q, J = 35.6 Hz), 143.1, 134.4, 121.6 (q, J = 273.4 Hz), 120.7 (q, J = 2.7 Hz), 52.5, 29.7, 29.1, 23.5. **^{19}F NMR** (377 MHz, $CDCl_3$): δ -67.6. **HRMS** (ESI): m/z calculated for $C_{12}H_{13}F_3N_2O$ requires 259.1053 for $[M+H]^+$, found 259.1053.

1-(2-methylpyrimidin-5-yl)azepan-2-one (1ae)

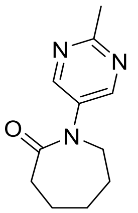

Procedure A was followed using ε-caprolactam (500 mg, 4.42 mmol, 1.2 equiv), CuI (35.1 mg, 0.18 mmol, 5 mol%), K_3PO_4 (1.56 g, 7.36 mmol, 2.0 equiv) and 5-bromo-2-methylpyrimidine (637 mg, 3.68 mmol, 1.0 equiv). Silica gel column chromatography (EA/MeOH 9:1) gave the desired lactam **1ae** as a pale brown solid, 10% (75.0 mg, 0.37 mmol). **m.p.**: 58–62 °C. **IR** (thin film): ν_{max} (cm^{-1}) = 2930, 2858, 1657, 1447, 1409, 1255, 1219, 1197, 982, 745. **^{1}H NMR** (400 MHz, $CDCl_3$): δ 8.54 (s, 2H), 3.79 – 3.72 (m, 2H), 2.73 – 2.69 (m, 5H), 1.90 – 1.77 (m, 6H). **^{13}C NMR** (101 MHz, $CDCl_3$): δ 175.9, 165.4, 154.0, 136.7, 52.6, 37.5, 29.8, 29.2, 25.7, 23.5. **HRMS** (ESI): m/z calculated for $C_{11}H_{15}N_3O$ requires 206.1288 for $[M+H]^+$, found 206.1290.

Acetyl etamivan (1ah)

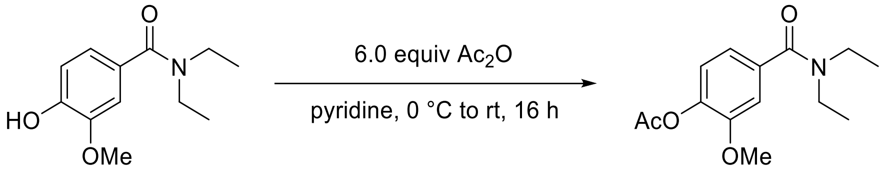

Etamivan (150 mg, 0.67 mmol, 1.0 equiv) was dissolved in 0.76 mL of pyridine (0.88 M). Acetic anhydride (0.38 mL, 412 mg, 4.03 mmol, 6.0 equiv) was then added dropwise at 0 °C and the mixture was left stirring at room temperature overnight. The mixture was then concentrated under reduced pressure. Silica gel column chromatography of the crude residue (EA/P 7:3 → 4:1) gave acetylated etamivan **1ah** as a colorless oil, quant. (178 mg, 0.67 mmol). **IR** (thin film): ν_{max} (cm^{-1}) = 2974, 2938, 1765, 1625, 1461, 1292, 1262, 1191, 1160, 1120, 1031, 902, 818, 795. **^{1}H NMR** (400 MHz, $CDCl_3$): δ 7.06 – 6.97 (m, 2H), 6.92 (dd, J = 8.0, 1.8 Hz, 1H), 3.83 (s, 3H), 3.51 (s, 2H), 3.30 (s, 2H), 2.30 (s, 3H), 1.18 (s, 6H). **^{13}C NMR** (101 MHz, $CDCl_3$): δ 170.6, 168.8, 151.4, 140.6, 135.9, 122.8, 118.7, 111.2, 56.1, 43.5, 39.5, 20.8, 14.3, 12.9. **HRMS** (ESI): m/z calculated for $C_{14}H_{19}NO_4$ requires 266.1387 for $[M+H]^+$, found 266.1388.

benzyl 2-bromo-2,2-difluoroacetate (2b)

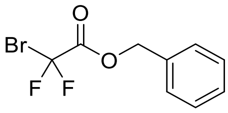

Procedure B was followed using 2-bromo-2,2-difluoroacetyl chloride (2.00 g, 0.98 mL, 10.3 mmol, 1.0 equiv), benzyl alcohol (2.24 g, 2.14 mL, 20.7 mmol, 2.0 equiv) and Et_3N (1.15 g, 1.59 mL, 11.4 mmol, 1.1 equiv). Silica gel column chromatography (P/Et_2O 97:3 → 95:5) gave bromodifluoroester **2b** as a colorless liquid, 84% (2.30 g, 8.68 mmol). NMR spectra matched those reported in literature.[15] **^{1}H NMR** (400 MHz, $CDCl_3$): δ 7.47 – 7.35 (m, 3H), 5.37 (s, 2H). **^{13}C NMR** (101 MHz, $CDCl_3$): δ 159.6 (t, J = 31.5 Hz), 133.6, 129.3, 129.0, 128.7, 108.9 (t, J = 314.4 Hz), 69.9. **^{19}F NMR** (377 MHz, $CDCl_3$): δ -60.7.

2-(trimethylsilyl)ethyl 2-bromo-2,2-difluoroacetate (2c)

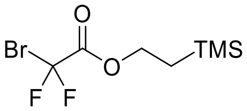

Procedure B was followed using 2-bromo-2,2-difluoroacetyl chloride (2.00 g, 0.98 mL, 10.3 mmol, 1.0 equiv), 2-(trimethylsilyl)ethan-1-ol (2.45 g, 2.96 mL, 20.7 mmol, 2.0 equiv) and Et_3N (1.15 g, 1.59 mL, 11.4 mmol, 1.1 equiv). Silica gel column chromatography (P/Et_2O 95:5) gave bromodifluoroester **2c** as a colorless liquid, 77% (2.20 g, 8.00 mmol). NMR spectra matched those reported in literature.[15] **^{1}H NMR** (400 MHz, $CDCl_3$): δ 4.49 – 4.40 (m, 2H), 1.18 – 1.08 (m, 2H), 0.08 (s,

9H). **^{13}C NMR** (101 MHz, $CDCl_3$): δ 159.8 (t, J = 31.0 Hz), 109.1 (t, J = 314.5 Hz), 67.5, 17.3, -1.4. **^{19}F NMR** (377 MHz, $CDCl_3$): δ -60.7.

isopropyl 2-bromo-2,2-difluoroacetate (2d)

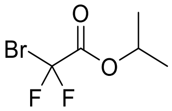

Procedure B was followed using 2-bromo-2,2-difluoroacetyl chloride (2.50 g, 1.22 mL, 12.9 mmol, 1.0 equiv), propan-2-ol (1.55 g, 1.93 mL, 25.9 mmol, 2.0 equiv) and Et_3N (1.44 g, 1.98 mL, 14.2 mmol, 1.1 equiv). Silica gel column chromatography (P/Et_2O 95:5) gave bromodifluoroester **2d** as a volatile, colorless liquid, 64% (1.80 g, 8.29 mmol). NMR spectra matched those reported in literature.[12] **^{1}H NMR** (400 MHz, $CDCl_3$): δ 5.19 (hept, J = 6.3 Hz, 1H), 1.37 (d, J = 6.3 Hz, 6H). **^{13}C NMR** (101 MHz, $CDCl_3$): δ 159.3 (t, J = 30.9 Hz), 109.2 (t, J = 314.6 Hz), 73.4, 21.4. **^{19}F NMR** (377 MHz, $CDCl_3$): δ -61.0.

(1*R*,2*S*,5*R*)-2-isopropyl-5-methylcyclohexyl 2-bromo-2,2-difluoroacetate (2e)

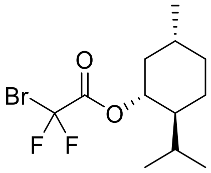

Procedure B was followed using 2-bromo-2,2-difluoroacetyl chloride (2.00 g, 0.98 mL, 10.3 mmol, 1.0 equiv), L-menthol (3.23 g, 20.7 mmol, 2.0 equiv) and Et_3N (1.15 g, 1.59 mL, 11.4 mmol, 1.1 equiv). Silica gel column chromatography (P/Et_2O 95:5) gave bromodifluoroester **2e** as a colorless liquid, 80% (2.60 g, 8.30 mmol). NMR spectra matched those reported in literature.[15] **^{1}H NMR** (400 MHz, $CDCl_3$): δ 4.84 (td, J = 11.0, 4.5 Hz, 1H), 2.11 – 2.01 (m, 1H), 1.98 – 1.84 (m, 1H), 1.79 – 1.66 (m, 2H), 1.60 – 1.45 (m, 2H), 1.23 – 1.01 (m, 2H), 0.99 – 0.84 (m, 7H), 0.79 (d, J = 6.9 Hz, 3H). **^{13}C NMR** (101 MHz, $CDCl_3$): δ 159.4 (t, J = 30.8 Hz), 109.1 (t, J = 314.8 Hz), 79.7, 47.0, 40.0, 34.1, 31.6, 23.5, 22.0, 20.7, 16.3. **^{19}F NMR** (377 MHz, $CDCl_3$): δ -60.8 (d, J = 8.7 Hz).

(2,2-dimethyl-1,3-dioxolan-4-yl)methyl 2-bromo-2,2-difluoroacetate (2f)

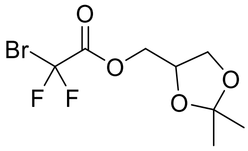

Procedure B was followed using 2-bromo-2,2-difluoroacetyl chloride (2.00 g, 0.98 mL, 10.3 mmol, 1.0 equiv), (2,2-dimethyl-1,3-dioxolan-4-yl)methanol (2.73 g, 20.7 mmol, 2.0 equiv) and Et_3N (1.15 g, 1.59 mL, 11.4 mmol, 1.1 equiv). Silica gel column chromatography (P/Et_2O 95:5) gave bromodifluoroester **2f** as a colorless liquid, 62% (1.86 g, 6.43 mmol). **IR** (thin film): ν_{max} (cm^{-1}) = 2990,

2891, 1777, 1301, 1160, 1124, 1057, 971, 839, 708. **^{1}H NMR** (400 MHz, $CDCl_3$): δ 4.43 – 4.30 (m, 3H), 4.16 – 4.06 (m, 1H), 3.87 – 3.78 (m, 1H), 1.43 (d, J = 0.8 Hz, 3H), 1.36 (d, J = 0.8 Hz, 3H). **^{13}C NMR** (101 MHz, $CDCl_3$): δ 159.5 (t, J = 31.8 Hz), 110.4, 108.6 (t, J = 314.2 Hz), 72.9, 67.6, 66.0, 26.7, 25.4. **^{19}F NMR** (377 MHz, $CDCl_3$): δ -60.7. **HRMS** (EI): m/z calculated for $C_8H_{11}BrF_2O_4$ requires 272.9569 for $[M-CH_3]^+$, found 272.9567.

benzhydryl 2-bromo-2,2-difluoroacetate (2g)

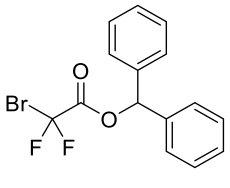

Procedure B was followed using 2-bromo-2,2-difluoroacetyl chloride (2.00 g, 0.98 mL, 10.3 mmol, 1.0 equiv), diphenylmethanol (3.81 g, 20.7 mmol, 2.0 equiv) and Et_3N (1.15 g, 1.59 mL, 11.4 mmol, 1.1 equiv). Silica gel column chromatography (P/Et_2O 95:5) gave bromodifluoroester **2g** as a colorless liquid, 77% (2.70 g, 7.91 mmol). **IR** (thin film): ν_{max} (cm^{-1}) = 3066, 3035, 1774, 1289, 1167, 1121, 958, 742, 709. **^{1}H NMR** (400 MHz, $CDCl_3$): δ 7.47 – 7.33 (m, 10H), 7.04 – 6.98 (m, 1H). **^{13}C NMR** (101 MHz, $CDCl_3$): δ 158.7 (t, J = 31.6 Hz), 138.1, 128.9, 128.9, 127.2, 109.0 (t, J = 314.5 Hz), 81.2. **^{19}F NMR** (377 MHz, $CDCl_3$): δ -57.4 – -64.9 (m). **HRMS** (EI): m/z calculated for $C_{15}H_{11}BrF_2O_2$ requires 339.9905 for $[M]^+$, found 339.9898.

2-bromo-2,2-difluoro-1-(4-methylpiperidin-1-yl)ethan-1-one (2i)

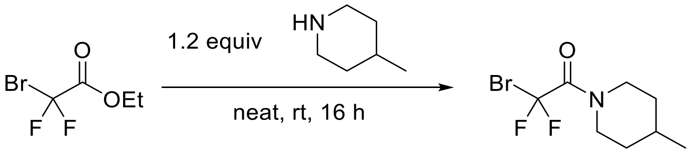

An oven-dried round bottom-flask, equipped with a magnetic stirring bar and a septum, was three times vacuumed and backfilled with Argon. Ethyl 2-bromo-2,2-difluoroacetate (2.00 g, 1.27 mL, 9.85 mmol, 1.0 equiv) was added, followed by dropwise addition of 4-methylpiperidine (1.17 g, 1.40 mL, 11.8 mL, 1.2 equiv). The resulting solution was left to stir over night, then diluted with DCM (30 mL) and washed with water. The aqueous phase was extracted with DCM and the combined organic phases were washed with brine (50 mL), dried over Na_2SO_4, filtered, concentrated *in vacuo* and purified *via* silica gel column chromatography (Et_2O/P 3:2 → 7:3). Bromodifluoroamide **2i** was obtained as a colorless liquid, 87% (2.20 g, 8.59 mmol). **IR** (thin film): ν_{max} (cm^{-1}) = 2928, 2873, 1679, 1453, 1168, 1138, 1114, 961, 847, 719, 649. **^{1}H NMR** (400 MHz, $CDCl_3$): δ 4.50 – 4.40 (m, 1H), 4.20 – 4.09 (m, 1H), 3.12 – 3.01 (m, 1H), 2.81 – 2.69 (m, 1H), 1.79 – 1.58 (m, 3H), 1.31 – 1.11 (m, 2H), 0.96 (d, J = 6.4 Hz, 3H). **^{13}C NMR** (101 MHz, $CDCl_3$): δ 157.8 (t, J = 26.0 Hz), 111.0 (t, J = 314.5 Hz), 47.0 (t, J = 4.2 Hz), 44.5,

34.1, 33.7, 30.9, 21.5. **^{19}F NMR** (377 MHz, $CDCl_3$): δ -53.78 (d, J = 30.3 Hz). **HRMS** (APCI): m/z calculated for $C_8H_{12}BrF_2NO$ requires 256.0143 for $[M+H]^+$, found 256.0141.

5. Synthesis and characterization of products

ethyl 3-(dimethylamino)-2,2-difluoro-3-(naphthalen-1-yl)propanoate (3a)

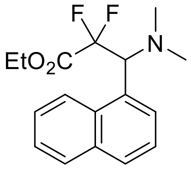

Procedure C was followed using N,N-dimethyl-1-naphthamide (**1a**) (30.0 mg, 0.15 mmol, 1.0 equiv), TMDS (30.3 mg, 40 µL, 0.23 mmol, 1.5 equiv) and Vaska's catalyst (1.2 mg, 1.51 µmol, 1 mol%). Silica gel column chromatography (P/Et_2O/Et_3N 97:3:1 → 95:5:1) gave the desired product **3a** as a colorless oil, 75% (34.6 mg, 0.11 mmol). **IR** (thin film): ν_{max} (cm^{-1}) = 2940, 2873, 2836, 2795, 1772, 1757, 1299, 1219, 1186, 1097, 1082, 1064, 1048, 798, 780. **^{1}H NMR** (400 MHz, $CDCl_3$): δ 8.18 (d, *J* = 8.6 Hz, 1H), 7.91 – 7.82 (m, 2H), 7.76 (d, *J* = 7.3 Hz, 1H), 7.61 – 7.44 (m, 3H), 5.24 – 5.12 (m, 1H), 4.17 – 4.00 (m, 2H), 2.42 (s, 6H), 1.01 (t, *J* = 7.1 Hz, 3H). **^{13}C NMR** (126 MHz, $CDCl_3$): δ 164.1 (t, *J* = 32.0 Hz), 134.1, 133.3, 129.4, 129.2, 128.3 – 128.2 (m), 127.9 (t, *J* = 3.3 Hz), 126.6, 125.7, 125.0, 123.5, 117.7 (t, *J* = 259.2 Hz), 64.5 – 63.5 (m), 62.7, 43.5 (t, *J* = 2.0 Hz), 13.7. **^{19}F NMR** (377 MHz, $CDCl_3$): δ -106.6 (d, *J* = 279.1 Hz), -109.6 (dd, *J* = 255.4, 16.9 Hz). **HRMS** (ESI): m/z calculated for $C_{17}H_{19}F_2NO_2$ requires 308.1457 for $[M+H]^+$, found 308.1457.

ethyl 3-(dimethylamino)-2,2-difluoro-3-phenylpropanoate (3b)

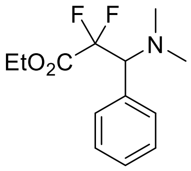

Procedure C was followed using *N,N*-dimethylbenzamide (**1b**) (23.0 mg, 0.15 mmol, 1.0 equiv), TMDS (31.1 mg, 41 µL, 0.23 mmol, 1.5 equiv) and Vaska's catalyst (1.2 mg, 1.54 µmol, 1 mol%). Silica gel column chromatography (P/Et_2O/Et_3N 98:2:1 → 97:3:1) gave the desired product **3b** as a colorless oil, 72% (28.4 mg, 0.11 mmol). **IR** (thin film): ν_{max} (cm^{-1}) = 2962, 2924, 2851, 1771, 1669, 1460, 1375, 1313, 1151, 1103, 803, 704. **^{1}H NMR** (400 MHz, $CDCl_3$): δ 7.38 (s, 5H), 4.35 – 4.16 (m, 3H), 2.27 (s, 6H), 1.28 (t, *J* = 7.1 Hz, 3H). **^{13}C NMR** (126 MHz, $CDCl_3$): δ 164.3 (dd, *J* = 33.3, 30.5 Hz), 130.8 (d, *J* = 2.6 Hz), 130.3, 128.7, 128.4, 117.0 (dd, *J* = 261.3, 255.7 Hz), 70.1 (dd, *J* = 26.8, 19.7 Hz), 62.7, 42.9, 14.0. **^{19}F NMR**

(377 MHz, $CDCl_3$): δ -105.9 (dd, *J* = 258.2, 9.7 Hz), -113.9 (dd, *J* = 256.4, 23.4 Hz). **HRMS** (ESI): m/z calculated for $C_{13}H_{17}F_2NO_2$ requires 258.1300 for $[M+H]^+$, found 258.1301.

ethyl 3-(4-cyanophenyl)-3-(dimethylamino)-2,2-difluoropropanoate (3c)

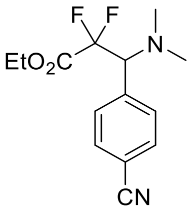

Procedure C was followed using 4-cyano-*N,N*-dimethylbenzamide (**1c**) (26.0 mg, 0.15 mmol, 1.0 equiv), TMDS (30.1 mg, 40 µL, 0.23 mmol, 1.5 equiv) and Vaska's catalyst (1.2 mg, 1.49 µmol, 1 mol%). Silica gel column chromatography (P/EA/Et_3N 80:20:1) gave the desired product **3c** as a colorless oil, 71% (30.0 mg, 0.11 mmol). **IR** (thin film): ν_{max} (cm^{-1}) = 2986, 2945, 2876, 2839, 2793, 2230, 1772, 1319, 1302, 1228, 1187, 1095, 1066, 816, 688. **^{1}H NMR** (400 MHz, $CDCl_3$): δ 7.81 – 7.62 (m, 2H), 7.50 (d, *J* = 8.4 Hz, 2H), 4.51 – 4.16 (m, 3H), 2.25 (s, 6H), 1.32 (t, *J* = 7.1 Hz, 3H). **^{13}C NMR** (101 MHz, $CDCl_3$): δ 163.8 (dd, *J* = 33.4, 30.0 Hz), 135.9, 132.1, 131.3 (d, *J* = 3.2 Hz), 118.5, 116.6 (dd, *J* = 262.8, 255.2 Hz), 112.7, 69.7 (dd, *J* = 27.9, 19.7 Hz), 63.0, 47.2 – 39.9 (m), 14.1. **^{19}F NMR** (377 MHz, $CDCl_3$): δ -104.1 (dd, *J* = 260.1, 8.8 Hz), -115.3 (dd, *J* = 260.1, 24.2 Hz). **HRMS** (ESI): m/z calculated for $C_{14}H_{16}F_2N_2O_2$ requires 283.1253 for $[M+H]^+$, found 283.1254.

ethyl 3-(4-bromophenyl)-3-(dimethylamino)-2,2-difluoropropanoate (3d)

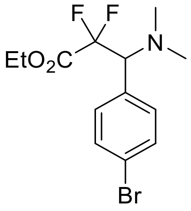

Procedure C was followed using 4-bromo-*N,N*-dimethylbenzamide (**1d**) (34.0 mg, 0.15 mmol, 1.0 equiv), TMDS (30.0 mg, 40 µL, 0.23 mmol, 1.5 equiv) and Vaska's catalyst (1.2 mg, 1.49 µmol, 1 mol%). Silica gel column chromatography (P/EA/Et_3N 95:5:1) gave the desired product **3d** as a colorless oil, 84% (41.9 mg, 0.12 mmol). **IR** (thin film): ν_{max} (cm^{-1}) = 2944, 2875, 2837, 2792, 1313, 1294, 1227, 1185, 1094, 1066, 1012, 874, 803, 729. **^{1}H NMR** (400 MHz, $CDCl_3$): δ 7.61 – 7.44 (m, 2H), 7.36 – 7.19 (m, 2H), 4.38 – 4.24 (m, 2H), 4.17 (dd, *J* = 23.4, 8.9 Hz, 1H), 2.24 (s, 6H), 1.31 (t, *J* = 7.1 Hz, 3H). **^{13}C NMR** (101 MHz, $CDCl_3$): δ 164.1 (dd, *J* = 33.4, 30.3 Hz), 132.3 (d, *J* = 2.8 Hz), 131.6, 129.3, 123.0, 116.8 (dd, *J* = 262.0, 255.3 Hz), 69.5 (dd, *J* = 27.3, 19.7 Hz), 62.8, 45.0 – 41.0 (m), 14.1. **^{19}F NMR** (377 MHz, $CDCl_3$): δ -105.1 (dd, *J* = 258.3, 10.1 Hz), -114.8 (dd, *J* = 258.4, 24.1 Hz). **HRMS** (ESI): m/z calculated for $C_{13}H_{16}BrF_2NO_2$ requires 336.0405 for $[M+H]^+$, found 336.0407.

ethyl 3-(dimethylamino)-2,2-difluoro-3-(2-fluorophenyl)propanoate (3e)

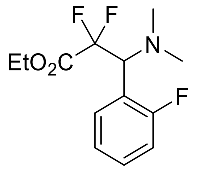

Procedure C was followed using 2-fluoro-*N,N*-dimethylbenzamide (**1e**) (25.0 mg, 0.15 mmol, 1.0 equiv), TMDS (30.1 mg, 40 µL, 0.23 mmol, 1.5 equiv) and Vaska's catalyst (1.2 mg, 1.50 µmol, 1 mol%). Silica gel column chromatography (P/EA/Et_3N 98:2:1 → 96:4:1) gave the desired product **3e** as a colorless oil, 79% (32.6 mg, 0.12 mmol). **IR** (thin film): ν_{max} (cm^{-1}) = 2947, 2876, 2839, 2794, 1775, 1761, 1490, 1458, 1309, 1218, 1096, 1066, 761. **^{1}H NMR** (400 MHz, $CDCl_3$): δ 7.59 (t, *J* = 7.7 Hz, 1H), 7.43 – 7.31 (m, 1H), 7.22 – 7.08 (m, 2H), 4.81 (dd, *J* = 24.2, 8.9 Hz, 1H), 4.43 – 4.24 (m, 2H), 2.27 (s, 6H), 1.31 (t, *J* = 7.1 Hz, 3H). **^{13}C NMR** (126 MHz, $CDCl_3$): δ 164.1 (dd, *J* = 33.8, 30.4 Hz), 162.1 (d, *J* = 246.4 Hz), 131.6 – 131.2 (m), 130.3 (d, *J* = 8.7 Hz), 123.9 (d, *J* = 3.7 Hz), 117.7 (d, *J* = 14.7 Hz), 117.0 (dd, *J* = 261.2, 255.1 Hz), 115.6 (d, *J* = 24.0 Hz), 62.8, 60.6 (ddd, *J* = 27.5, 19.9, 2.9 Hz), 42.9, 14.0. **^{19}F NMR** (377 MHz, $CDCl_3$): δ -104.9 (dd, *J* = 257.6, 10.2 Hz), -114.7 (dd, *J* = 257.6, 25.5 Hz), -115.3 – -116.1 (m). **HRMS** (ESI): m/z calculated for $C_{13}H_{16}F_3NO_2$ requires 276.1206 for $[M+H]^+$, found 276.1206.

ethyl 3-(dimethylamino)-2,2-difluoro-3-(2-methoxyphenyl)propanoate (3f)

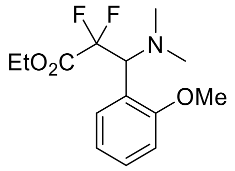

Procedure C was followed using 2-methoxy-*N,N*-dimethylbenzamide (**1f**) (27.0 mg, 0.15 mmol, 1.0 equiv), TMDS (30.4 mg, 40 µL, 0.23 mmol, 1.5 equiv) and Vaska's catalyst (1.2 mg, 1.51 µmol, 1 mol%). Silica gel column chromatography (P/EA/Et_3N 97:3:1 → 90:10:1) gave the desired product **3f** as a colorless oil, 62% (26.8 mg, 93.3 µmol). **IR** (thin film): ν_{max} (cm^{-1}) = 2945, 2839, 2792, 1772, 1491, 1463, 1310, 1244, 1092, 1062, 1031, 758. **^{1}H NMR** (400 MHz, $CDCl_3$): δ 7.63 – 7.47 (m, 1H), 7.32 (ddd, *J* = 8.3, 7.4, 1.7 Hz, 1H), 7.10 – 6.84 (m, 2H), 5.06 (dd, *J* = 24.2, 10.7 Hz, 1H), 4.38 – 4.20 (m, 2H), 3.83 (s, 3H), 2.26 (s, 6H), 1.27 (t, *J* = 7.1 Hz, 3H). **^{13}C NMR** (101 MHz, $CDCl_3$): δ 164.6 (dd, *J* = 33.8, 30.8 Hz), 158.7, 131.0 (dd, *J* = 5.2, 1.7 Hz), 129.7, 120.2, 119.3, 118.3 – 114.7 (m), 110.9, 62.5, 60.1 (dd, *J* = 26.6, 19.6 Hz), 55.7, 45.2 – 42.1 (m), 14.0. **^{19}F NMR** (377 MHz, $CDCl_3$): δ -106.0 (dd, *J* = 255.2, 11.9 Hz), -113.2 (dd, *J* = 255.5, 25.1 Hz). **HRMS** (ESI): m/z calculated for $C_{14}H_{19}F_2NO_3$ requires 288.1406 for $[M+H]^+$, found 288.1405.

ethyl 3-(dimethylamino)-2,2-difluoro-3-(furan-2-yl)propanoate (3g)

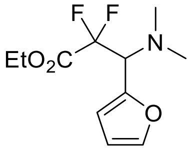

Procedure C was followed using *N,N*-dimethylfuran-2-carboxamide (**1g**) (21.0 mg, 0.15 mmol, 1.0 equiv), TMDS (30.4 mg, 40 µL, 0.23 mmol, 1.5 equiv) and Vaska's catalyst (1.2 mg, 1.51 µmol, 1 mol%). Silica gel column chromatography (P/EA/Et_3N 99:1:1 → 95:5:1) gave the desired product **3g** as a colorless oil, 64% (23.7 mg, 95.9 µmol). **IR** (thin film): ν_{max} (cm^{-1}) = 2984, 2947, 2878, 2841, 2796, 1775, 1334, 1223, 1098, 1061, 743. **^{1}H NMR** (400 MHz, $CDCl_3$): δ 7.46 (dd, *J* = 1.8, 0.8 Hz, 1H), 6.60 – 6.32 (m, 2H), 4.48 – 4.27 (m, 3H), 2.28 (s, 6H), 1.34 (t, *J* = 7.1 Hz, 3H). **^{13}C NMR** (101 MHz, $CDCl_3$): δ 163.9 (dd, *J* = 33.1, 30.0 Hz), 146.1, 143.1, 115.9 (dd, *J* = 261.7, 255.2 Hz), 111.3 (d, *J* = 2.9 Hz), 110.3, 63.8 (dd, *J* = 27.6, 20.5 Hz), 62.8, 45.8 – 40.4 (m), 14.1. **^{19}F NMR** (377 MHz, $CDCl_3$): δ -104.3 (dd, *J* = 258.1, 8.7 Hz), -117.2 (dd, *J* = 258.0, 24.2 Hz). **HRMS** (ESI): m/z calculated for $C_{11}H_{15}F_2NO_3$ requires 248.1093 for $[M+H]^+$, found 248.1095.

ethyl 2,2-difluoro-3-(4-fluorophenyl)-3-(pyrrolidin-1-yl)propanoate (3h)

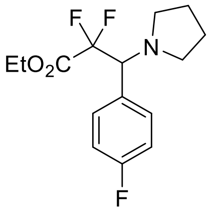

Procedure C was followed using (4-fluorophenyl)(pyrrolidin-1-yl)methanone (**1h**) (29.0 mg, 0.15 mmol, 1.0 equiv), TMDS (30.2 mg, 40 µL, 0.23 mmol, 1.5 equiv) and Vaska's catalyst (1.2 mg, 1.50 µmol, 1 mol%). Silica gel column chromatography (P/Et_2O/Et_3N 98:2:1 → 97:3:1) gave the desired product **3h** as a colorless oil, 77% (34.6 mg, 0.11 mmol). **IR** (thin film): ν_{max} (cm^{-1}) = 2975, 2942, 2812, 1773, 1606, 1511, 1309, 1230, 1125, 1088, 1063, 858. **^{1}H NMR** (400 MHz, $CDCl_3$): δ 7.42 – 7.32 (m, 2H), 7.13 – 6.98 (m, 2H), 4.29 – 4.15 (m, 3H), 2.77 – 2.38 (m, 4H), 1.67 (td, *J* = 6.1, 3.3 Hz, 4H), 1.22 (t, *J* = 7.1 Hz, 3H). **^{13}C NMR** (126 MHz, $CDCl_3$): δ 164.1 (t, *J* = 31.9 Hz), 163.0 (d, *J* = 247.5 Hz), 132.2 (d, *J* = 8.1 Hz), 128.4, 116.4 (t, *J* = 258.8 Hz), 115.3 (d, *J* = 21.3 Hz), 68.1 (t, *J* = 22.9 Hz), 62.7, 51.2, 23.3, 14.0. **^{19}F NMR** (377 MHz, $CDCl_3$): δ -109.2 – -111.0 (m), -112.9 – -114.0 (m). **HRMS** (ESI): m/z calculated for $C_{15}H_{18}F_3NO_2$ requires 302.1362 for $[M+H]^+$, found 302.1363.

ethyl 3-([1,1'-biphenyl]-4-yl)-2,2-difluoro-3-(pyrrolidin-1-yl)propanoate (3i)

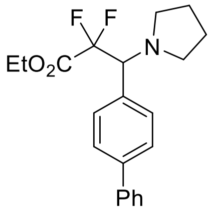

Procedure C was followed using [1,1'-biphenyl]-4-yl(pyrrolidin-1-yl)methanone (**1i**) (38.0 mg, 0.15 mmol. 1.0 equiv), TMDS (30.5 mg, 40 µL, 0.23 mmol, 1.5 equiv) and Vaska's catalyst (1.2 mg, 1.51 µmol, 1 mol%). Silica gel column chromatography (P/Et_2O/Et_3N 98:2:1 → 97:3:1) gave the desired product **3i** as a colorless oil, 78% (42.2 mg, 0.12 mmol). **IR** (thin film): ν_{max} (cm^{-1}) = 2972, 2817, 1772, 1487, 1310, 1298, 1123, 1088, 1061, 763, 744, 699. **^{1}H NMR** (400 MHz, $CDCl_3$): δ 7.63 – 7.56 (m, 4H), 7.50 – 7.41 (m, 4H), 7.39 – 7.33 (m, 1H), 4.35 – 4.15 (m, 3H), 2.73 – 2.57 (m, 4H), 1.76 – 1.64 (m, 4H), 1.23 (t, *J* = 7.2 Hz, 3H). **^{13}C NMR** (126 MHz, $CDCl_3$): δ 164.2 (t, *J* = 32.0 Hz), 141.4, 140.6, 131.3, 131.0, 128.9, 127.6, 127.2, 127.0, 116.6 (t, *J* = 261.1, 259.1 Hz), 68.5 (t, *J* = 22.9 Hz), 62.6, 51.2, 23.3, 13.9. **^{19}F NMR** (377 MHz, $CDCl_3$): δ -109.9 (dd, *J* = 35.5, 16.4 Hz). **HRMS** (ESI): m/z calculated for $C_{21}H_{23}F_2NO_2$ requires 360.1770 for $[M+H]^+$, found 360.1770.

ethyl 2,2-difluoro-3-phenyl-3-(piperidin-1-yl)propanoate (3j)

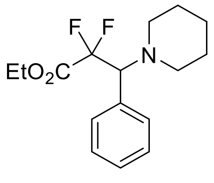

Procedure C was followed using phenyl(piperidin-1-yl)methanone (**1j**) (29.0 mg, 0.15 mmol, 1.0 equiv), TMDS (30.9 mg, 41 µL, 0.23 mmol, 1.5 equiv) and Vaska's catalyst (1.2 mg, 1.53 µmol, 1 mol%). Silica gel column chromatography (P/Et_2O/Et_3N 98:2:1) gave the desired product **3j** as a colorless oil, 69% (31.5 mg, 0.11 mmol). **IR** (thin film): ν_{max} (cm^{-1}) = 2936, 2853, 2813, 1773, 1759, 1308, 1208, 1101, 1059, 716, 702. **^{1}H NMR** (400 MHz, $CDCl_3$): δ 7.42 – 7.34 (m, 5H), 4.45 – 4.27 (m, 2H), 4.22 (dd, *J* = 26.4, 7.7 Hz, 1H), 2.75 – 2.64 (m, 2H), 2.34 – 2.24 (m, 2H), 1.55 – 1.46 (m, 4H), 1.37 (t, *J* = 7.2 Hz, 3H), 1.32 – 1.24 (m, 2H). **^{13}C NMR** (126 MHz, $CDCl_3$): δ 164.7 (dd, *J* = 33.8, 30.0 Hz), 130.8, 130.7 (d, *J* = 3.1 Hz), 128.5, 128.2, 117.5 (dd, *J* = 263.6, 254.3 Hz), 71.1 (dd, *J* = 28.7, 18.8 Hz), 62.6, 51.8, 26.7, 24.1, 14.2. **^{19}F NMR** (377 MHz, $CDCl_3$): δ -103.1 (dd, *J* = 255.2, 8.3 Hz), -115.8 (dd, *J* = 255.3, 26.9 Hz). **HRMS** (ESI): m/z calculated for $C_{16}H_{21}F_2NO_2$ requires 298.1613 for $[M+H]^+$, found 298.1610.

ethyl 2,2-difluoro-3-(piperidin-1-yl)-3-(4-(4,4,5,5-tetramethyl-1,3,2-dioxaborolan-2-yl)phenyl)propanoate (3k)

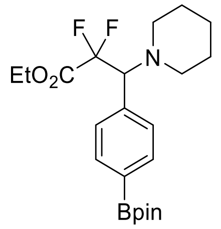

Procedure C was followed using piperidin-1-yl(4-(4,4,5,5-tetramethyl-1,3,2-dioxaborolan-2-yl)phenyl)methanone (**1k**) (47.0 mg, 0.15 mmol, 1.0 equiv), TMDS (30.0 mg, 40 µL, 0.23 mmol, 1.5 equiv) and Vaska's catalyst (1.2 mg, 1.49 µmol, 1 mol%). Silica gel column chromatography (P/EA 8:2) gave the desired product **3k** as a colorless solid, 98% (61.8 mg, 0.15 mmol). **m.p.**: 130–134 °C. **IR** (thin film): ν_{max} (cm^{-1}) = 2979, 2855, 28131774, 1361, 1144, 1067, 1059, 858. **^{1}H NMR** (400 MHz, $CDCl_3$): δ 7.81 (d, *J* = 8.1 Hz, 2H), 7.38 (d, *J* = 7.8 Hz, 2H), 4.44 – 4.27 (m, 2H), 4.23 (dd, *J* = 26.3, 7.5 Hz, 1H), 2.77 – 2.59 (m, 2H), 2.37 – 2.21 (m, 2H), 1.56 – 1.43 (m, 4H), 1.41 – 1.30 (m, 15H), 1.30 – 1.21 (m, 2H). **^{13}C NMR** (101 MHz, $CDCl_3$): δ 164.6 (dd, *J* = 34.0, 29.9 Hz), 134.6, 133.9, 130.0 (d, *J* = 3.0 Hz), 117.5 (dd, *J* = 263.7, 254.5 Hz), 84.0, 71.2 (dd, *J* = 28.7, 18.8 Hz), 62.6, 51.9, 26.7, 25.0 (d, *J* = 3.7 Hz), 24.1, 14.2. **^{19}F NMR** (377 MHz, $CDCl_3$): δ -103.1 (dd, *J* = 255.7, 8.1 Hz), -115.7 (dd, *J* = 256.0, 26.9 Hz). **HRMS** (ESI): m/z calculated for $C_{22}H_{32}BF_2NO_4$ requires 424.2467 for $[M+H]^+$, found 424.2465.

ethyl 2,2-difluoro-3-(4-iodophenyl)-3-(1,4-dioxa-8-azaspiro[4.5]decan-8-yl)propanoate (3l)

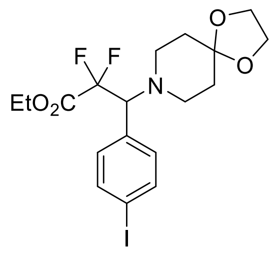

Procedure C was followed using (4-iodophenyl)(1,4-dioxa-8-azaspiro[4.5]decan-8-yl)methanone (**1l**) (56.0 mg, 0.15 mmol, 1.0 equiv), TMDS (30.2 mg, 40 µL, 0.23 mmol, 1.5 equiv) and Vaska's catalyst (1.2 mg, 1.50 µmol, 1 mol%). Silica gel column chromatography ($P/Et_2O/Et_3N$ 90:10:1 → 80:20:1) gave the desired product **3l** as a colorless solid, 61% (44.0 mg, 91.1 µmol). **m.p.**: 148–152 °C. **IR** (thin film): ν_{max} (cm^{-1}) = 2968, 2889, 2847, 2828, 1763, 1313, 1213, 1095, 1083, 1062, 836, 731. **^{1}H NMR** (400 MHz, $CDCl_3$): δ 7.73 – 7.67 (m, 2H), 7.16 – 7.09 (m, 2H), 4.42 – 4.29 (m, 2H), 4.24 (dd, *J* = 25.6, 7.3 Hz, 1H), 3.86 (s, 4H), 2.83 – 2.73 (m, 2H), 2.45 – 2.35 (m, 2H), 1.65 (t, *J* = 5.7 Hz, 4H), 1.36 (t, *J* = 7.1 Hz, 3H). **^{13}C NMR** (126 MHz, $CDCl_3$): δ 164.3 (t, *J* = 31.5, 31.1 Hz), 137.7, 132.3, 130.1, 117.0 (dd, *J* = 261.6, 255.8 Hz), 106.5, 95.0, 69.8 (dd, *J* = 29.0, 19.2 Hz), 64.3, 62.9, 48.7, 35.5, 14.2. **^{19}F NMR** (377 MHz, $CDCl_3$): δ -

103.1 (dd, *J* = 257.1, 8.1 Hz), -116.4 (dd, *J* = 257.1, 26.2 Hz). **HRMS** (ESI): m/z calculated for $C_{18}H_{22}F_2INO_4$ requires 482.0634 for $[M+H]^+$, found 482.0632.

ethyl 2,2-difluoro-3-morpholino-3-(4-nitrophenyl)propanoate (3m)

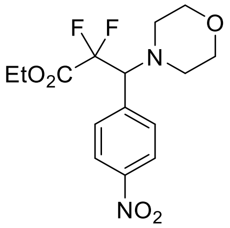

Procedure C was followed using morpholino(4-nitrophenyl)methanone (**1m**) (36.0 mg, 0.15 mmol, 1.0 equiv), TMDS (30.7mg, 41 µL, 0.23 mmol, 1.52 equiv) and Vaska's catalyst (1.2 mg, 1.5 µmol, 1 mol%). Silica gel column chromatography (P/EA/Et_3N 70:30:1) gave the desired product **3m** as a colorless oil, 91% (47.5 mg, 0.14 mmol). **IR** (thin film): $ν_{max}$ (cm^{-1}) = 2916, 2856, 1771, 1523, 1349, 1313, 1115, 1096, 1064, 1011, 856, 710. **^{1}H NMR** (400 MHz, $CDCl_3$): δ 8.29 – 8.22 (m, 2H), 7.57 (d, *J* = 8.8 Hz, 2H), 4.51 – 4.26 (m, 3H), 3.68 – 3.55 (m, 4H), 2.88 – 2.61 (m, 2H), 2.44 – 2.34 (m, 2H), 1.37 (t, *J* = 7.1 Hz, 3H). **^{13}C NMR** (101 MHz, $CDCl_3$): δ 163.7 (dd, *J* = 33.1, 29.8 Hz), 148.3, 137.7, 131.4 (d, *J* = 3.2 Hz), 123.6, 116.5 (dd, *J* = 263.4, 255.8 Hz), 70.0 (dd, *J* = 28.5, 19.5 Hz), 67.2, 63.2, 50.9, 14.2. **^{19}F NMR** (377 MHz, $CDCl_3$): δ -103.4 (dd, *J* = 260.2, 7.6 Hz), -114.8 (dd, *J* = 260.4, 24.8 Hz). **HRMS** (ESI): m/z calculated for $C_{15}H_{18}F_2N_2O_5$ requires 345.1257 for $[M+H]^+$, found 345.1257.

ethyl 3-(azepan-1-yl)-2,2-difluoro-3-phenylpropanoate (3n)

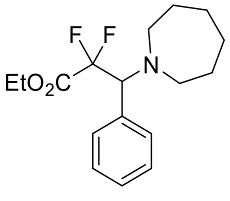

Procedure C was followed using azepan-1-yl(phenyl)methanone (**1n**) (31.0 mg, 0.15 mmol, 1.0 equiv), TMDS (30.7 mg, 40 µL, 0.23 mmol, 1.5 equiv) and Vaska's catalyst (1.2 mg, 1.52 µmol, 1 mol%). Silica gel column chromatography (P/Et_2O/Et_3N 99:1:1 → 98:2:1) gave the desired product **3n** as a colorless oil, 77% (36.6 mg, 72.9 µmol). **IR** (thin film): $ν_{max}$ (cm^{-1}) = 2927, 2854, 1773, 1757, 1453, 1318, 1226, 1094, 1063, 719, 701. **^{1}H NMR** (400 MHz, $CDCl_3$): δ 7.47 – 7.40 (m, 2H), 7.44 – 7.29 (m, 2H), 4.44 (dd, *J* = 26.6, 6.8 Hz, 1H), 4.41 – 4.28 (m, 2H), 2.93 (dt, *J* = 12.8, 5.0 Hz, 2H), 2.59 – 2.49 (m, 2H), 1.60 – 1.41 (m, 8H), 1.38 (t, *J* = 7.2 Hz, 3H). **^{13}C NMR** (101 MHz, $CDCl_3$): δ 164.8 (dd, *J* = 34.0, 30.3 Hz), 133.1, 130.0 (d, *J* = 2.7 Hz), 128.3, 128.2, 117.4 (dd, *J* = 262.9, 255.7 Hz), 70.2 (dd, *J* = 28.1, 18.7 Hz), 62.7, 53.3 (d, *J* = 3.0 Hz), 29.5, 26.9, 14.2. **^{19}F NMR** (377 MHz, $CDCl_3$): δ -101.9 (dd, *J* = 256.7, 7.9 Hz), -118.4 (dd, *J* = 256.8, 27.0 Hz). **HRMS** (ESI): m/z calculated for $C_{17}H_{23}F_2NO_2$ requires 312.1770 for $[M+H]^+$, found 312.1768.

ethyl 3-(azocan-1-yl)-2,2-difluoro-3-phenylpropanoate (3o)

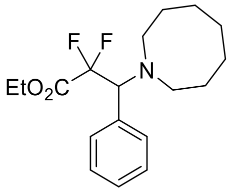

Procedure C was followed using azocan-1-yl(phenyl)methanone (**1o**) (33.0 mg, 0.15 mmol, 1.0 equiv), TMDS (30.6 mg, 40 µL, 0.23 mmol, 1.5 equiv) and Vaska's catalyst (1.2 mg, 1.52 µmol, 1 mol%). Silica gel column chromatography (P/Et_2O/Et_3N 99:1:1 → 98:2:1) gave the desired product **3o** as a colorless oil, 73% (36.0 mg, 72.9 µmol). **IR** (thin film): ν_{max} (cm^{-1}) = 2922, 2855, 1773, 1757, 1454, 1315, 1229, 1093, 1063, 1019, 723, 702. **^{1}H NMR** (400 MHz, $CDCl_3$): δ 7.46 – 7.32 (m, 5H), 4.42 (dd, *J* = 25.8, 8.0 Hz, 1H), 4.33 (q, *J* = 7.2 Hz, 2H), 2.90 – 2.79 (m, 2H), 2.56 – 2.45 (m, 2H), 1.60 – 1.41 (m, 10H), 1.34 (t, *J* = 7.2 Hz, 3H). **^{13}C NMR** (126 MHz, $CDCl_3$): δ 164.8 (dd, *J* = 33.4, 30.5 Hz), 132.6, 130.5, 128.3, 117.1 (dd, *J* = 262.0, 253.9 Hz), 70.2 (dd, *J* = 27.7, 18.9 Hz), 62.9, 52.5, 27.9, 27.3, 25.4, 14.1. **^{19}F NMR** (377 MHz, $CDCl_3$): δ -101.9 (dd, *J* = 259.9, 9.4 Hz), -115.7 (dd, *J* = 260.3, 26.6 Hz). **HRMS** (ESI): m/z calculated for $C_{18}H_{25}F_2NO_2$ requires 326.1926 for $[M+H]^+$, found 326.1924.

ethyl 3-(dibenzylamino)-2,2-difluoro-3-(4-fluorophenyl)propanoate (3p)

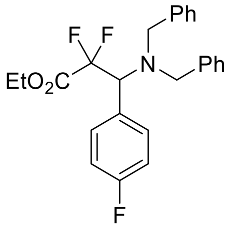

Procedure C was followed using *N,N*-dibenzyl-4-fluorobenzamide (**1p**) (48.0 mg, 0.15 mmol, 1.0 equiv), TMDS (50.5 mg, 66 µL, 0.38 mmol, 2.5 equiv) and Vaska's catalyst (2.4 mg, 3.01 µmol, 2 mol%). Silica gel column chromatography (P/Et_2O/Et_3N 99:1:1 → 98:2:1) gave the desired product **3p** as a colorless solid, 64% (41.4 mg, 96.9 µmol). **m.p.**: 118–122 °C. **IR** (thin film): ν_{max} (cm^{-1}) = 3029, 2928, 2844, 1770, 1511, 1309, 1232, 1113, 1063, 750, 729, 700. **^{1}H NMR** (400 MHz, $CDCl_3$): δ 7.40 – 7.29 (m, 2H), 7.29 – 7.14 (m, 10H), 7.11 – 7.02 (m, 2H), 4.35 – 4.21 (m, 2H), 4.01 – 3.93 (m, 3H), 3.04 (d, *J* = 13.4 Hz, 2H), 1.10 (t, *J* = 7.1 Hz, 3H). **^{13}C NMR** (126 MHz, $CDCl_3$): δ 164.4 – 163.6 (m), 163.0 (d, *J* = 247.8 Hz), 138.7, 132.8 (dd, *J* = 8.1, 2.4 Hz), 129.3, 128.5, 127.5, 126.3 (d, *J* = 3.5 Hz), 117.2 (dd, *J* = 259.6, 253.7 Hz), 115.5 (d, *J* = 21.1 Hz), 63.1 (dd, *J* = 28.8, 19.4 Hz), 63.0, 55.1 (d, *J* = 3.6 Hz), 13.8. **^{19}F NMR** (377 MHz, $CDCl_3$): δ -102.0 (dd, *J* = 257.1, 9.6 Hz), -108.6 – -119.5 (m). **HRMS** (ESI): m/z calculated for $C_{25}H_{24}F_3NO_2$ requires 428.1832 for $[M+H]^+$, found 428.1830.

ethyl 3-(benzyl(ethyl)amino)-2,2-difluoro-3-phenylpropanoate (3q)

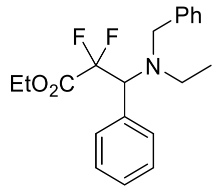

Procedure C was followed using *N*-benzyl-*N*-ethylbenzamide (**1q**) (36.0 mg, 0.15 mmol, 1.0 equiv), TMDS (50.5 mg, 66 µL, 0.38 mmol, 2.5 equiv) and Vaska's catalyst (2.4 mg, 3.01 µmol, 2 mol%). Silica gel column chromatography (P/Et_2O/Et_3N 99:1:1 → 98:2:1) gave the desired product **3q** as a colorless oil, 81% (42.5 mg, 0.12 mmol). **IR** (thin film): ν_{max} (cm^{-1}) = 2975, 2932, 2847, 1771, 1454, 1304, 1221, 1094, 1066, 719, 700. **^{1}H NMR** (400 MHz, $CDCl_3$): δ 7.43 – 7.29 (m, 5H), 7.28 – 7.13 (m, 5H), 4.39 (dd, J = 25.7, 7.8 Hz, 1H), 4.33 – 4.25 (m, 1H), 4.23 – 4.11 (m, 1H), 3.99 (d, J = 13.7 Hz, 1H), 3.00 (d, J = 13.7 Hz, 1H), 2.79 – 2.64 (m, 1H), 2.25 (dq, J = 13.4, 6.8 Hz, 1H), 1.23 (t, J = 7.2 Hz, 3H), 0.96 (t, J = 7.1 Hz, 3H). **^{13}C NMR** (126 MHz, $CDCl_3$): δ 164.5 (dd, J = 34.0, 29.9 Hz), 139.5, 131.3, 130.9 (d, J = 2.5 Hz), 128.9, 128.6, 128.4, 128.3, 127.1, 117.5 (dd, J = 261.2, 253.3 Hz), 64.6 (dd, J = 29.0, 19.2 Hz), 62.9, 55.3 (d, J = 3.7 Hz), 45.1 (d, J = 3.0 Hz), 14.0, 13.6. **^{19}F NMR** (377 MHz, $CDCl_3$): δ -101.8 (dd, J = 256.6, 9.2 Hz), -114.8 (dd, J = 256.7, 26.0 Hz). **HRMS** (ESI): m/z calculated for $C_{20}H_{23}F_2NO_2$ requires 348.1770 for $[M+H]^+$, found 348.1768.

ethyl 2,2-difluoro-3-(methyl(phenyl)amino)-3-(naphthalen-2-yl)propanoate (3r)

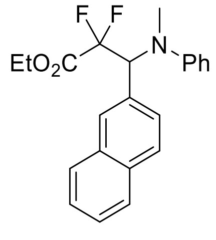

Procedure C was followed using *N*-methyl-*N*-phenyl-2-naphthamide (**1r**) (39.0 mg, 0.15 mmol, 1.0 equiv), TMDS (50.1 mg, 66 µL, 0.37 mmol, 2.5 equiv) and Vaska's catalyst (2.3 mg, 2.98 µmol, 2 mol%). Silica gel column chromatography (P/Et_2O/Et_3N 99:1:1 → 98:2:1) gave the desired product **3r** as a colorless solid, 87% (47.8 mg, 0.13 mmol). **m.p.**: 116–118 °C. **IR** (thin film): ν_{max} (cm^{-1}) = 3058, 2962, 2912, 1771, 1599, 1504, 1278, 1220, 1194, 1093, 1061, 748, 694. **^{1}H NMR** (400 MHz, $CDCl_3$): δ 7.97 (s, 1H), 7.90 – 7.78 (m, 3H), 7.56 – 7.47 (m, 2H), 7.41 (dd, J = 8.6, 1.9 Hz, 1H), 7.36 – 7.26 (m, 2H), 7.04 – 6.96 (m, 2H), 6.95 – 6.79 (m, 1H), 5.91 (dd, J = 19.9, 10.8 Hz, 1H), 4.24 – 4.00 (m, 2H), 2.81 (s, 3H), 1.13 (t, J = 7.2 Hz, 3H). **^{13}C NMR** (126 MHz, $CDCl_3$): δ 163.9 (dd, J = 33.0, 30.1 Hz), 150.0, 133.2, 133.0, 130.6 (d, J = 2.6 Hz), 129.4, 128.6, 128.5, 127.7, 127.6 – 127.6 (m), 126.7, 126.5, 126.3, 118.9, 116.8 (t, J = 261.1 Hz), 114.3, 63.9 (dd, J = 26.3, 21.3 Hz), 63.2, 33.8 (d, J = 3.7 Hz), 13.8. **^{19}F NMR** (377 MHz, $CDCl_3$):

δ -102.7 (dd, J = 257.6, 13.3 Hz), -112.1 (dd, J = 257.7, 22.7 Hz). **HRMS** (ESI): m/z calculated for $C_{22}H_{21}F_2NO_2$ requires 370.1613 for $[M+H]^+$, found 370.1614.

ethyl 3-((2-ethoxy-2-oxoethyl)(phenyl)amino)-2,2-difluoro-3-phenylpropanoate (3s)

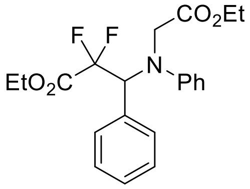

Procedure C (reduction time: 40 min) was followed using ethyl *N*-benzoyl-*N*-phenylglycinate (**1s**) (43.0 mg, 0.15 mmol, 1.0 equiv), TMDS (51.0 mg, 67 µL, 0.38 mmol, 2.5 equiv) and Vaska's catalyst (2.4 mg, 3.04 µmol, 2 mol%). Silica gel column chromatography (P/Et_2O/Et_3N 99:1:1 → 90:10:1) gave the desired product **3s** as a colorless oil, 56% (33.3 mg, 85.1 µmol). **IR** (thin film): ν_{max} (cm^{-1}) = 2984, 2919, 1755, 1731, 1599, 1504, 1301, 1185, 1097, 1063, 1044, 748, 697. **^{1}H NMR** (400 MHz, $CDCl_3$): δ 7.44 – 7.36 (m, 2H), 7.32 – 7.18 (m, 3H), 7.21 – 7.12 (m, 2H), 6.88 – 6.81 (m, 2H), 6.82 – 6.76 (m, 1H), 5.77 (dd, J = 18.6, 11.6 Hz, 1H), 4.18 – 3.74 (m, 6H), 1.13 – 0.95 (m, 6H). **^{13}C NMR** (101 MHz, $CDCl_3$): δ 170.1, 163.5 (dd, J = 32.4, 30.7 Hz), 148.3, 133.0, 129.4, 129.1 (t, J = 2.2 Hz), 128.8, 128.7, 119.8, 116.3 (t, J = 259.8 Hz), 115.0, 63.3 (dd, J = 25.4, 21.2 Hz), 63.2, 60.9, 48.8 (d, J = 2.7 Hz), 14.1, 13.7. **^{19}F NMR** (377 MHz, $CDCl_3$): δ -104.7 (dd, J = 262.9, 13.4 Hz), -111.6 (dd, J = 264.5, 21.0 Hz). **HRMS** (ESI): m/z calculated for $C_{21}H_{23}F_2NO_4$ requires 392.1668 for $[M+H]^+$, found 392.1668.

ethyl 2,2-difluoro-3-(methoxy(methyl)amino)-3-phenylpropanoate (3t)

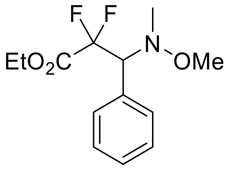

Procedure C was followed using *N*-methoxy-*N*-methylbenzamide (**1t**) (25.0 mg, 0.15 mmol, 1.0 equiv), TMDS (30.5 mg, 40 µL, 0.23 mmol, 1.5 equiv) and Vaska's catalyst (1.2 mg, 1.51 µmol, 1 mol%). Silica gel column chromatography (P/Et_2O/Et_3N 98:2:1 → 95:5:1) gave the desired product **3t** as a colorless oil, 91% (37.8 mg, 0.14 mmol). **IR** (thin film): ν_{max} (cm^{-1}) = 2987, 2967, 1771, 1310, 1299, 1098, 1069, 1039, 728, 701. **^{1}H NMR** (400 MHz, $CDCl_3$): δ 7.50 – 7.41 (m, 2H), 7.41 – 7.31 (m, 3H), 4.41 – 4.22 (m, 3H), 3.48 (s, 3H), 2.42 (s, 3H), 1.36 (t, J = 7.1 Hz, 3H). **^{13}C NMR** (101 MHz, $CDCl_3$): δ 164.1 (dd, J = 34.1, 30.8 Hz), 132.6, 130.4, 129.2, 128.5, 113.6 (dd, J = 264.8, 243.1 Hz), 74.4 (dd, J = 28.8, 19.6 Hz), 62.6, 59.8, 43.2, 14.1. **^{19}F NMR** (377 MHz, $CDCl_3$): δ -105.0 (d, J = 268.4 Hz), -122.0 (d, J = 326.5 Hz). **HRMS** (ESI): m/z calculated for $C_{13}H_{17}F_2NO_3$ requires 274.1249 for $[M+H]^+$, found 274.1251.

ethyl (*E*)-2,2-difluoro-5-phenyl-3-(pyrrolidin-1-yl)pent-4-enoate (3u)

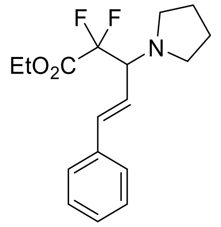

Procedure C was followed using (*E*)-3-phenyl-1-(pyrrolidin-1-yl)prop-2-en-1-one (**1u**) (30.0 mg, 0.15 mmol, 1.0 equiv), TMDS (30.0 mg, 40 µL, 0.22 mmol, 1.5 equiv) and Vaska's catalyst (1.2 mg, 1.49 µmol, 1 mol%). Silica gel column chromatography (P/EA/Et_3N 98:2:1 → 95:5:1) gave the desired product **3u** as a colorless oil, 34% (15.9 mg, 51.4 µmol). **IR** (thin film): ν_{max} (cm^{-1}) = 2967, 2938, 2819, 1772, 1307, 1200, 1113, 1075, 1042, 972, 752, 695. **^{1}H NMR** (400 MHz, $CDCl_3$): δ 7.46 – 7.38 (m, 2H), 7.38 – 7.23 (m, 3H), 6.65 (d, J = 15.9 Hz, 1H), 6.33 (dd, J = 15.9, 9.5 Hz, 1H), 4.41 – 4.24 (m, 2H), 3.91 (dt, J = 20.5, 9.4 Hz, 1H), 2.79 – 2.62 (m, 4H), 1.74 – 1.60 (m, 4H), 1.33 (t, J = 7.1 Hz, 3H). **^{13}C NMR** (101 MHz, $CDCl_3$): δ 164.4 (dd, J = 33.0, 30.5 Hz), 138.2, 136.3, 128.8, 128.4, 126.9, 118.5 (d, J = 4.0 Hz), 116.5 (dd, J = 259.2, 254.1 Hz), 66.0 (dd, J = 27.6, 21.5 Hz), 62.5, 49.6, 23.6, 14.1. **^{19}F NMR** (377 MHz, $CDCl_3$): δ -105.9 (dd, J = 253.6, 9.6 Hz), -116.7 (dd, J = 253.7, 21.3 Hz). **HRMS** (ESI): m/z calculated for $C_{17}H_{21}F_2NO_2$ requires 310.1613 for $[M+H]^+$, found 310.1614.

ethyl (E)-3-(dimethylamino)-2,2-difluoro-5-phenylpent-4-enoate (3v)

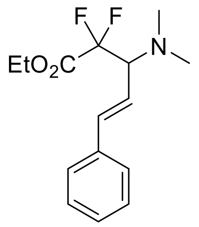

Procedure C was followed using *N,N*-dimethylcinnamamide (**1v**) (27.0 mg, 0.15 mmol, 1.0 equiv), TMDS (31.1 mg, 41 µL, 0.23 mmol, 1.5 equiv) and Vaska's catalyst (1.2 mg, 1.54 µmol, 1 mol%). Silica gel column chromatography (P/Et_2O/Et_3N 97:3:1 → 90:10:1) gave the desired product **3v** as a colorless oil, 58% (25.5 mg, 90.0 µmol). **IR** (thin film): ν_{max} (cm^{-1}) = 2986, 2974, 2875, 2837, 2794, 1768, 1305, 1213, 1178, 1127, 1080, 1057, 1042, 1026, 745, 692. **^{1}H NMR** (400 MHz, $CDCl_3$): δ 7.40 – 7.33 (m, 2H), 7.32 – 7.16 (m, 3H), 6.59 (d, J = 15.9 Hz, 1H), 6.23 (dd, J = 15.9, 9.5 Hz, 1H), 4.36 – 4.19 (m, 2H), 3.67 – 3.53 (m, 1H), 2.25 (dt, J = 22.1, 9.2 Hz, 6H), 1.27 (t, J = 7.1 Hz, 3H). **^{13}C NMR** (101 MHz, $CDCl_3$): δ 164.4 (dd, J = 33.7, 30.2 Hz), 138.9, 136.3, 128.8, 128.4, 126.9, 117.0 (d, J = 3.6 Hz), 116.6 (dd, J = 260.3, 253.5 Hz), 68.8 (dd, J = 28.4, 20.4 Hz), 62.6, 42.2, 14.1. **^{19}F NMR** (377 MHz, $CDCl_3$): δ -103.8 (dd, J = 256.5, 9.6 Hz), -118.5 (dd, J = 256.4, 22.4 Hz). **HRMS** (ESI): m/z calculated for $C_{15}H_{19}F_2NO_2$ requires 284.1457 for $[M+H]^+$, found 284.1457.

ethyl 3-cyclohexyl-2,2-difluoro-3-(methyl(phenyl)amino)propanoate (3w)

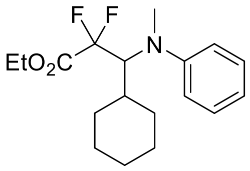

Procedure C was followed using *N*-methyl-*N*-phenylcyclohexanecarboxamide (**1w**) (33.0 mg, 0.15 mmol, 1.0 equiv), TMDS (51.0 mg, 67 µL, 0.38 mmol, 2.5 equiv) and Vaska's catalyst (2.4 mg, 3.04 µmol, 2 mol%). Silica gel column chromatography ($P/Et_2O/Et_3N$ 99:1:1 → 98:2:1) gave the desired product **3w** as a colorless oil, 73% (36.3 mg, 0.11 mmol). **IR** (thin film): ν_{max} (cm^{-1}) = . **^{1}H NMR** (400 MHz, $CDCl_3$): δ 7.24 – 7.17 (m, 2H), 6.83 – 6.69 (m, 3H), 4.25 – 4.12 (m, 1H), 4.10 – 3.78 (m, 2H), 2.78 (d, J = 1.8 Hz, 3H), 2.19 – 2.01 (m, 2H), 1.83 – 1.72 (m, 1H), 1.70 – 1.60 (m, 2H), 1.54 – 1.40 (m, 2H), 1.36 – 1.09 (m, 5H), 1.03 (t, J = 7.2 Hz, 3H). **^{13}C NMR** (126 MHz, $CDCl_3$): δ 164.1 (dd, J = 34.5, 30.1 Hz), 149.9, 129.2, 117.9 (t, J = 262.3 Hz), 117.8, 113.2, 65.0 (dd, J = 26.7, 23.2 Hz), 62.7, 36.2, 31.9 (d, J = 3.9 Hz), 30.5, 29.8 (d, J = 6.4 Hz), 26.3, 26.1, 26.0 (d, J = 2.0 Hz), 13.7. **^{19}F NMR** (377 MHz, $CDCl_3$): δ -89.7 – -99.5 (m), -115.2 (dd, J = 252.7, 21.8 Hz). **HRMS** (ESI): m/z calculated for $C_{18}H_{25}F_2NO_2$ requires 326.1926 for $[M+H]^+$, found 326.1925.

ethyl 3-(dimethylamino)-2,2-difluoro-5-phenylpentanoate (3x)

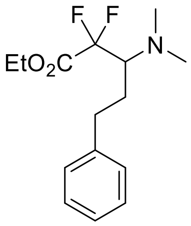

Procedure C was followed using *N,N*-dimethyl-3-phenylpropanamide (**1x**) (27.0 mg, 0.15 mmol, 1.0 equiv), TMDS (30.7 mg, 40 µL, 0.23 mmol, 1.5 equiv) and Vaska's catalyst (1.2 mg, 1.52 µmol, 1 mol%). Silica gel column chromatography ($P/Et_2O/Et_3N$ 98:2:1) gave the desired product **3x** as a colorless oil, 68% (29.6 mg, 0.10 mmol). **IR** (thin film): ν_{max} (cm^{-1}) = 2943, 2795, 1771, 1456, 1308, 1778, 1161, 1103, 1068, 1029, 852, 740, 699. **^{1}H NMR** (400 MHz, $CDCl_3$): δ 7.36 – 7.28 (m, 2H), 7.25 – 7.17 (m, 3H), 4.31 (qd, J = 7.1, 4.5 Hz, 2H), 3.24 – 3.08 (m, 1H), 2.74 (t, J = 8.1 Hz, 2H), 2.37 (s, 6H), 2.04 – 1.84 (m, 2H), 1.32 (t, J = 7.1 Hz, 3H). **^{13}C NMR** (126 MHz, $CDCl_3$): δ 164.7 (dd, J = 33.6, 30.5 Hz), 141.7, 128.6, 128.5, 126.2, 118.2 (dd, J = 261.3, 257.0 Hz), 64.4 (dd, J = 26.1, 21.5 Hz), 62.4, 41.7 (d, J = 3.0 Hz), 33.5 (d, J = 2.0 Hz), 24.4 (d, J = 2.8 Hz), 14.1. **^{19}F NMR** (377 MHz, $CDCl_3$): δ -103.0 (dd, J = 254.8, 10.8 Hz), -119.3 (dd, J = 254.9, 22.3 Hz). **HRMS** (ESI): m/z calculated for $C_{15}H_{21}F_2NO_2$ requires 286.1613 for $[M+H]^+$, found 286.1612.

ethyl 2-(1-benzylpyrrolidin-2-yl)-2,2-difluoroacetate (3y)

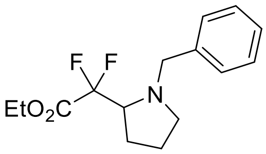

Procedure C (solvent for reduction: 2-Methyl-THF, reduction time: 2 min) was followed using 1-benzylpyrrolidin-2-one (**1y**) (26.0 mg, 0.15 mmol, 1.0 equiv), TMDS (29.9 mg, 39 µL, 0.22 mmol, 1.5 equiv) and Vaska's catalyst (1.2 mg, 1.48 µmol, 1 mol%). Silica gel column chromatography (P/EA/Et_3N 99:1:1 → 98:2:1) gave the desired product **3y** as a colorless oil, 60% (25.3 mg, 89.3 µmol). **IR** (thin film): ν_{max} (cm^{-1}) = 2978, 2804, 1769, 1756, 1310, 1103, 1030, 747, 729. **^{1}H NMR** (400 MHz, $CDCl_3$): δ 7.34 – 7.18 (m, 5H), 4.37 – 4.25 (m, 2H), 4.15 (d, J = 13.3 Hz, 1H), 3.51 (d, J = 13.4 Hz, 1H), 3.41 (tdd, J = 11.5, 9.8, 3.6 Hz, 1H), 2.91 (ddd, J = 9.4, 6.4, 2.9 Hz, 1H), 2.39 – 2.25 (m, 1H), 2.21 – 2.10 (m, 1H), 2.05 – 1.90 (m, 1H), 1.86 – 1.66 (m, 2H), 1.34 (t, J = 7.2 Hz, 3H). **^{13}C NMR** (101 MHz, $CDCl_3$): δ 164.6 (t, J = 32.7 Hz), 139.5, 128.6, 128.3, 127.0, 116.9 (dd, J = 255.5, 253.2 Hz), 65.7 (t, J = 25.0 Hz), 62.7, 60.4 (d, J = 1.7 Hz), 54.5, 26.1 (t, J = 3.5 Hz), 24.4, 14.1. **^{19}F NMR** (377 MHz, $CDCl_3$): δ -108.2 (dd, J = 255.2, 12.1 Hz), -114.1 (dd, J = 255.3, 12.7 Hz). **HRMS** (ESI): m/z calculated for $C_{15}H_{19}F_2NO_2$ requires 284.1457 for $[M+H]^+$, found 284.1456.

ethyl 2-(1-benzylpiperidin-2-yl)-2,2-difluoroacetate (3z)

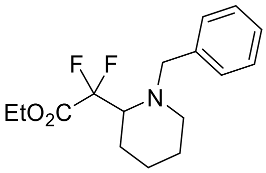

Procedure C (solvent for reduction: THF) was followed using 1-benzylpiperidin-2-one (**1z**) (29.0 mg, 0.15 mmol, 1.0 equiv), TMDS (30.9 mg, 41 µL, 0.23 mmol, 1.5 equiv) and Vaska's catalyst (1.2 mg, 1.53 µmol, 1 mol%). Silica gel column chromatography (P/EA/Et_3N 99:1:1 → 98:2:1) gave the desired product **3z** as a colorless oil, 42% (19.3 mg, 64.9 µmol). **IR** (thin film): ν_{max} (cm^{-1}) = 2939, 2857, 1769, 1453, 1337, 1124, 1089, 1073, 1058, 735, 699. **^{1}H NMR** (400 MHz, $CDCl_3$): δ 7.17 – 7.01 (m, 5H), 4.28 – 4.10 (m, 1H), 4.09 – 3.97 (m, 1H), 3.73 (d, J = 13.6 Hz, 1H), 3.63 (d, J = 13.6 Hz, 1H), 3.18 – 3.03 (m, 1H), 2.75 (ddt, J = 13.0, 9.6, 3.1 Hz, 1H), 2.25 (dt, J = 13.6, 4.7 Hz, 1H), 1.70 – 1.61 (m, 3H), 1.44 – 1.31 (m, 2H), 1.26 – 1.16 (m, 1H), 1.09 (t, J = 7.2 Hz, 3H). **^{13}C NMR** (101 MHz, $CDCl_3$): δ 164.7 (dd, J = 33.2, 30.8 Hz), 139.6, 128.6, 128.3, 127.1, 118.7 (dd, J = 260.0, 255.0 Hz), 62.6, 60.8 (dd, J = 26.5, 21.4 Hz), 58.1 (t, J = 2.4 Hz), 47.2 (d, J = 2.8 Hz), 21.6 (d, J = 2.4 Hz), 20.6, 20.3 (d, J = 2.4 Hz), 14.1. **^{19}F NMR** (377 MHz, $CDCl_3$): δ -102.4 (d, J = 254.1 Hz), -111.0 (d, J = 254.1 Hz). **HRMS** (ESI): m/z calculated for $C_{16}H_{21}F_2NO_2$ requires 298.1613 for $[M+H]^+$, found 298.1614.

ethyl 2-(1-benzylazepan-2-yl)-2,2-difluoroacetate (3aa)

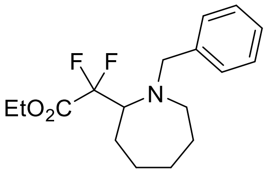

Procedure C was followed using 1-benzylazepan-2-one (**1aa**) (31.0 mg, 0.15 mmol, 1.0 equiv), TMDS (30.7 mg, 40 µL, 0.23 mmol, 1.5 equiv) and Vaska's catalyst (1.2 mg, 1.52 µmol, 1 mol%). Silica gel column chromatography (P/EA/Et_3N 99:1:1 → 98:2:1) gave the desired product **3aa** as a colorless oil, 57% (27.2 mg, 87.4 µmol). **IR** (thin film): ν_{max} (cm^{-1}) = 2928, 2855, 1771, 1754, 1453, 1324, 1295, 1210, 1126, 1076, 1056, 746, 729. **^{1}H NMR** (400 MHz, $CDCl_3$): δ 7.29 – 7.04 (m, 5H), 4.20 (q, J = 7.1 Hz, 2H), 4.12 (d, J = 13.8 Hz, 1H), 3.75 (d, J = 13.8 Hz, 1H), 3.49 – 3.34 (m, 1H), 2.83 – 2.48 (m, 2H), 2.09 – 1.96 (m, 1H), 1.87 – 1.41 (m, 5H), 1.36 – 1.16 (m, 5H). **^{13}C NMR** (101 MHz, $CDCl_3$): δ 165.0 (dd, J = 34.5, 31.0 Hz), 139.4, 128.5, 128.3, 127.0, 117.1 (dd, J = 259.9, 256.4 Hz), 65.3 (dd, J = 25.4, 21.5 Hz), 62.6, 57.0, 47.0, 29.0, 26.1 (d, J = 2.4 Hz), 25.8, 24.7, 14.2. **^{19}F NMR** (377 MHz, $CDCl_3$): δ -105.0 (dd, J = 254.5, 8.1 Hz), -120.9 (dd, J = 254.7, 21.0 Hz). **HRMS** (ESI): m/z calculated for $C_{17}H_{23}F_2NO_2$ requires 312.1770 for $[M+H]^+$, found 312.1770.

ethyl 2,2-difluoro-2-(1-phenylazepan-2-yl)acetate (3ab)

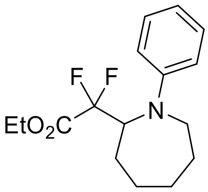

Procedure C was followed using 1-phenylazepan-2-one (**1ab**) (29.0 mg, 0.15 mmol, 1.0 equiv), TMDS (30.9 mg, 40 µL, 0.23 mmol, 1.5 equiv) and Vaska's catalyst (1.2 mg, 1.53 µmol, 1 mol%). Silica gel column chromatography (P/Et_2O/Et_3N 99:1:1 → 98:2:1) gave the desired product **3ab** as a colorless oil, 81% (37.3 mg, 0.12 mmol). **IR** (thin film): ν_{max} (cm^{-1}) = 2931, 2855, 1769, 1597, 1504, 1314, 1269, 1193, 1174, 1102, 1058, 746, 692. **^{1}H NMR** (400 MHz, $CDCl_3$): δ 7.28 – 7.17 (m, 2H), 6.84 (d, J = 8.6 Hz, 2H), 6.75 – 6.67 (m, 1H), 4.55 – 4.39 (m, 1H), 4.22 – 4.03 (m, 2H), 3.79 – 3.68 (m, 1H), 3.33 (ddd, J = 16.1, 11.7, 1.7 Hz, 1H), 2.28 – 2.16 (m, 1H), 1.94 – 1.77 (m, 3H), 1.77 – 1.53 (m, 2H), 1.42 – 1.26 (m, 2H), 1.16 (t, J = 7.1 Hz, 3H). **^{13}C NMR** (101 MHz, $CDCl_3$): δ 164.4 (dd, J = 32.9, 31.5 Hz), 148.0, 129.3, 117.0, 116.5 (t, J = 258.2 Hz), 112.8, 63.0, 58.3 (dd, J = 25.5, 21.5 Hz), 44.8 – 44.3 (m), 30.1, 27.4 (d, J = 3.2 Hz), 26.1, 24.5, 13.8. **^{19}F NMR** (377 MHz, $CDCl_3$): δ -112.0 (dd, J = 253.0, 12.5 Hz), -115.1 (dd, J = 253.0, 14.9 Hz). **HRMS** (ESI): m/z calculated for $C_{16}H_{21}F_2NO_2$ requires 298.1613 for $[M+H]^+$, found 298.1614.

ethyl 2,2-difluoro-2-(1-(5-methylpyridin-2-yl)azepan-2-yl)acetate (3ac)

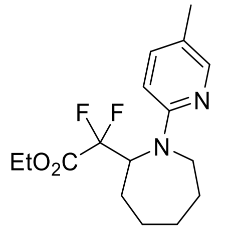

Procedure C was followed using 1-(5-methylpyridin-2-yl)azepan-2-one (**1ac**) (31.0 mg, 0.15 mmol, 1.0 equiv), TMDS (30.6 mg, 40 µL, 0.23 mmol, 1.5 equiv) and Vaska's catalyst (1.2 mg, 1.52 µmol, 1 mol%). Silica gel column chromatography (P/Et_2O/Et_3N 98:2:1 → 95:5:1) gave the desired product **3ac** as a colorless oil, 74% (35.2 mg, 0.11 mmol). **IR** (thin film): ν_{max} (cm^{-1}) = 2928, 2856, 1765, 1611, 1490, 1405, 1296, 1194, 1104, 1061, 807. **^{1}H NMR** (400 MHz, $CDCl_3$): δ 7.87 – 7.82 (m, 1H), 7.23 – 7.16 (m, 1H), 6.45 (d, J = 8.6 Hz, 1H), 5.51 – 5.35 (m, 1H), 3.96 (q, J = 7.1 Hz, 2H), 3.72 – 3.62 (m, 1H), 3.34 – 3.22 (m, 1H), 2.30 – 2.17 (m, 1H), 2.09 (s, 3H), 1.86 – 1.69 (m, 3H), 1.62 – 1.41 (m, 2H), 1.34 – 1.16 (m, 1H), 1.14 – 1.06 (m, 1H), 1.04 (t, J = 7.2 Hz, 3H). **^{13}C NMR** (101 MHz, $CDCl_3$): δ 164.3 (dd, J = 33.1, 31.0 Hz), 155.9, 147.3, 138.4, 121.2, 116.1 (t, J = 257.5 Hz), 105.8, 62.6, 54.7 (dd, J = 28.2, 21.1 Hz), 43.6 (d, J = 2.7 Hz), 30.2, 26.6, 26.5 (d, J = 2.6 Hz), 24.4, 17.3, 13.7. **^{19}F NMR** (377 MHz, $CDCl_3$): δ -109.7 (d, J = 250.0 Hz), -119.1 (dd, J = 251.8, 26.7 Hz). **HRMS** (ESI): m/z calculated for $C_{16}H_{22}F_2N_2O_2$ requires 313.1722 for $[M+H]^+$, found 313.1717.

ethyl 2,2-difluoro-2-(1-(6-(trifluoromethyl)pyridin-3-yl)azepan-2-yl)acetate (3ad)

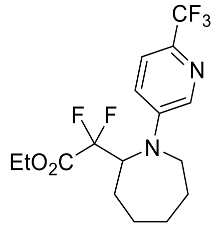

Procedure C was followed using 1-(6-(trifluoromethyl)pyridin-3-yl)azepan-2-one (**1ad**) (39.0 mg, 0.15 mmol, 1.0 equiv), TMDS (30.4 mg, 40 µL, 0.23 mmol, 1.5 equiv) and Vaska's catalyst (1.2 mg, 1.51 µmol, 1 mol%). Silica gel column chromatography (P/Et_2O/Et_3N 90:10:1 → 80:20:1) gave the desired product **3ad** as a colorless oil, 75% (41.3 mg, 0.11 mmol). **IR** (thin film): ν_{max} (cm^{-1}) = 2935, 2859, 1766, 1586, 1344, 1312, 1200, 1124, 1092, 1060, 828. **^{1}H NMR** (400 MHz, $CDCl_3$): δ 8.31 (d, J = 3.1 Hz, 1H), 7.48 (d, J = 8.9 Hz, 1H), 7.15 (dd, J = 9.0, 3.1 Hz, 1H), 4.49 – 4.33 (m, 1H), 4.26 (qd, J = 7.2, 3.0 Hz, 2H), 3.80 – 3.69 (m, 1H), 3.42 (ddd, J = 16.1, 11.7, 1.7 Hz, 1H), 2.25 – 2.11 (m, 1H), 1.99 – 1.80 (m, 3H), 1.77 – 1.53 (m, 2H), 1.42 – 1.30 (m, 1H), 1.27 (t, J = 7.2 Hz, 3H), 1.21 – 1.07 (m, 1H). **^{13}C NMR** (101 MHz, $CDCl_3$): δ 163.8 (t, J = 31.7 Hz), 145.9, 136.2 (q, J = 35.6 Hz), 134.5, 125.1 (q, J = 272.6 Hz), 120.9 (q, J = 2.7 Hz), 118.1 (d, J = 1.8 Hz), 116.1 (dd, J = 258.8, 256.3 Hz), 63.5, 58.8 (dd, J = 24.1, 21.2 Hz), 44.7, 29.7, 27.4 (d, J = 3.2 Hz), 25.7, 24.5, 14.0. **^{19}F NMR** (377 MHz, $CDCl_3$): δ -66.5, -111.2 (dd, J = 257.5, 10.6 Hz),

-115.8 (dd, J = 257.5, 16.0 Hz). **HRMS** (ESI): m/z calculated for $C_{16}H_{19}F_5N_2O_2$ requires 367.1439 for $[M+H]^+$, found 367.1440.

ethyl 2,2-difluoro-2-(1-(2-methylpyrimidin-5-yl)azepan-2-yl)acetate (3ae)

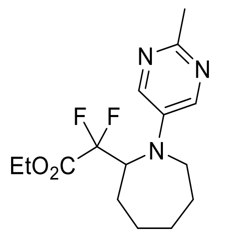

Procedure C was followed using 1-(2-methylpyrimidin-5-yl)azepan-2-one (**1ae**) (31.0 mg, 0.15 mmol, 1.0 equiv), TMDS (30.4 mg, 40 µL, 0.23 mmol, 1.5 equiv) and Vaska's catalyst (1.2 mg, 1.51 µmol, 1 mol%). Silica gel column chromatography (P/EA/Et_3N 60:40:1 → 50:50:1) gave the desired product **3ae** as a colorless oil, 63% (29.9 mg, 95.4 µmol). **IR** (thin film): ν_{max} (cm^{-1}) = 2931, 2857, 1769, 1470, 1297, 1175, 1061, 743. **^{1}H NMR** (400 MHz, $CDCl_3$): δ 8.24 (s, 2H), 4.37 – 4.17 (m, 3H), 3.73 – 3.60 (m, 1H), 3.35 (ddd, J = 16.1, 11.4, 2.0 Hz, 1H), 2.58 (s, 3H), 2.22 – 2.09 (m, 1H), 1.97 – 1.76 (m, 3H), 1.72 – 1.48 (m, 2H), 1.42 – 1.29 (m, 1H), 1.25 (t, J = 7.2 Hz, 3H), 1.19 – 1.06 (m, 1H). **^{13}C NMR** (101 MHz, $CDCl_3$): δ 163.8 (t, J = 31.9 Hz), 156.3, 140.8 (d, J = 1.7 Hz), 139.3, 116.2 (dd, J = 258.4, 256.1 Hz), 63.3, 58.6 (dd, J = 24.5, 21.5 Hz), 44.2, 29.8, 27.4 (d, J = 3.2 Hz), 25.6, 24.6, 24.5, 14.0. **^{19}F NMR** (377 MHz, $CDCl_3$): δ -112.1 (dd, J = 257.3, 11.4 Hz), -114.9 (dd, J = 257.4, 15.2 Hz). **HRMS** (ESI): m/z calculated for $C_{15}H_{21}F_2N_3O_2$ requires 314.1675 for $[M+H]^+$, found 314.1674.

ethyl (4E,6E)-7-(benzo[d][1,3]dioxol-5-yl)-2,2-difluoro-3-(piperidin-1-yl)hepta-4,6-dienoate (3af)

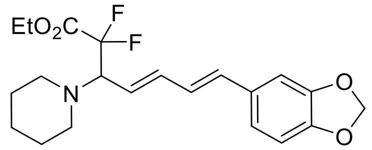

Procedure C (reduction time: 1 h) was followed using piperin (**1af**) (43.0 mg, 0.15 mmol, 1.0 equiv), TMDS (50.6 mg, 67 µL, 0.38 mmol, 2.5 equiv) and Vaska's catalyst (2.4 mg, 3.01 µmol, 2 mol%). Silica gel column chromatography (P/Et_2O/Et_3N 95:5:1 → 90:10:1) gave the desired product **3af** as a pale yellow oil, 56% (33.1 mg, 84.1 µmol). **IR** (thin film): ν_{max} (cm^{-1}) = 2934, 2853, 2810, 1772, 1504, 1490, 1446, 1310, 1251, 1199, 1100, 1039, 991, 931. **^{1}H NMR** (400 MHz, $CDCl_3$): δ 6.95 (d, J = 1.7 Hz, 1H), 6.84 (dd, J = 8.2, 1.7 Hz, 1H), 6.76 (d, J = 8.0 Hz, 1H), 6.66 (dd, J = 15.6, 10.3 Hz, 1H), 6.55 – 6.34 (m, 2H), 5.97 (s, 2H), 5.86 (dd, J = 15.2, 9.5 Hz, 1H), 4.45 – 4.24 (m, 2H), 3.55 (dt, J = 23.5, 8.9 Hz, 1H), 2.77 – 2.67 (m, 2H), 2.44 – 2.34 (m, 2H), 1.60 – 1.44 (m, 4H), 1.42 – 1.31 (m, 5H). **^{13}C NMR** (126 MHz, $CDCl_3$): δ 164.6 (dd, J = 33.5, 30.2 Hz), 148.3, 147.7, 138.5, 133.7, 131.6, 126.3, 121.7, 121.1 (d, J = 3.6 Hz), 117.0 (dd, J = 261.3, 253.3 Hz), 108.6, 105.6, 101.3, 70.0 (dd, J = 28.9, 20.0 Hz), 62.5, 51.4, 26.6, 24.3,

14.3. **^{19}F NMR** (377 MHz, $CDCl_3$): δ -102.5 (dd, J = 253.7, 9.0 Hz), -118.1 (dd, J = 253.6, 23.7 Hz). **HRMS** (ESI): m/z calculated for $C_{21}H_{25}F_2NO_4$ requires 394.1824 for $[M+H]^+$, found 394.1820.

ethyl 3-(diethylamino)-2,2-difluoro-4-(naphthalen-1-yloxy)pentanoate (3ag)

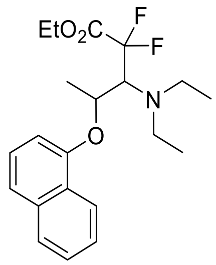

Procedure C (reduction time: 1 h) was followed using napropamide (**1ag**) (41.0 mg, 0.15 mmol, 1.0 equiv), TMDS (50.7 mg, 67 µL, 0.38 mmol, 2.5 equiv) and Vaska's catalyst (2.4 mg, 3.02 µmol, 2 mol%). Silica gel column chromatography ($P/Et_2O/Et_3N$ 99:1:1 → 98:2:1) gave the desired product **3ag** as a colorless oil, 69% (39.8 mg, 0.10 mmol) (55:45 mixture of diastereomers, based on ^{1}H NMR spectroscopic analysis). **IR** (thin film): ν_{max} (cm^{-1}) = 3054, 2976, 1773, 1755, 1579, 1399, 1311, 1265, 1237, 1144, 1097, 1076, 1040, 792, 728. **^{1}H NMR** (400 MHz, $CDCl_3$): δ 8.23 – 8.15 (m, 0.5H), 8.13 – 8.04 (m, 0.5H), 7.85 – 7.74 (m, 1H), 7.54 – 7.34 (m, 4H), 6.92 (d, J = 7.5 Hz, 0.5H), 6.88 (d, J = 7.3 Hz, 0.5H), 5.22 – 5.11 (m, 0.5H), 4.98 – 4.87 (m, 0.5H), 4.42 – 4.21 (m, 1H), 4.07 (dq, J = 10.7, 7.1 Hz, 0.5H), 3.94 – 3.79 (m, 1H), 3.79 – 3.68 (m, 0.5H), 3.03 – 2.70 (m, 4H), 1.51 (dd, J = 6.1, 4.2 Hz, 1.5H), 1.44 – 1.40 (m, 1.5H), 1.39 (t, J = 7.2 Hz, 1.5H), 1.13 (t, J = 7.1 Hz, 3H), 1.01 (t, J = 7.2 Hz, 3H), 0.89 (t, J = 7.1 Hz, 1.5H). **^{13}C NMR** (126 MHz, $CDCl_3$): δ 165.1 – 164.0 (m)(overlapped), 152.4, 152.2, 135.0, 134.8, 127.7, 127.6, 126.8, 126.5, 126.5, 126.0, 126.0, 125.3, 125.3, 122.4, 122.3, 121.1, 120.6, 120.9 – 116.4 (m)(overlapped), 107.3, 105.2, 72.2 – 72.0 (m), 70.6, 66.9 (dd, J = 25.5, 20.8 Hz), 66.1 (t, J = 20.1 Hz), 62.6, 62.6, 46.4, 45.6, 17.9, 17.4 (d, J = 6.9 Hz), 15.3, 14.8, 14.1, 13.5. **^{19}F NMR** (377 MHz, $CDCl_3$): δ -94.7 – -96.4 (m), -107.6 (dd, J = 265.1, 5.3 Hz), -114.3 (dd, J = 265.1, 24.2 Hz), -117.2 (dd, J = 252.3, 23.9 Hz). **HRMS** (ESI): m/z calculated for $C_{21}H_{27}F_2NO_3$ requires 380.2032 for $[M+H]^+$, found 380.2031.

ethyl 3-(4-acetoxy-3-methoxyphenyl)-3-(diethylamino)-2,2-difluoropropanoate (3ah)

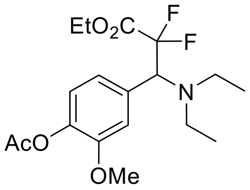

Procedure C was followed using acetyl etamivan (**1ah**) (40.0 mg, 0.15 mmol, 1.0 equiv), TMDS (50.6 mg, 67 µL, 0.38 mmol, 2.5 equiv) and Vaska's catalyst (2.4 mg, 3.02 µmol, 2 mol%). Silica gel column chromatography ($P/Et_2O/Et_3N$ 97:3:2 → 90:10:2) gave the desired product **3ah** as a colorless solid, 74% (41.6 mg, 0.11 mmol). **m.p.**: 86–90 °C. **IR** (thin film): ν_{max} (cm^{-1}) = 2968, 2931, 1761, 1514,

1461, 1369, 1295, 1211, 1094, 1065, 1019, 778, 705. **^{1}H NMR** (400 MHz, $CDCl_3$): δ 7.06 – 6.92 (m, 3H), 4.47 – 4.24 (m, 3H), 3.83 (s, 3H), 2.81 (dq, J = 14.3, 7.3 Hz, 2H), 2.36 – 2.23 (m, 5H), 1.35 (t, J = 7.2 Hz, 3H), 1.01 (t, J = 7.0 Hz, 6H). **^{13}C NMR** (126 MHz, $CDCl_3$): δ 169.0, 164.6 (dd, J = 33.7, 29.7 Hz), 150.8, 140.0, 130.9, 123.3 (d, J = 2.3 Hz), 122.5, 117.6 (dd, J = 262.1, 254.2 Hz), 114.7 (d, J = 3.0 Hz), 65.2 (dd, J = 29.0, 19.1 Hz), 62.8, 56.1, 44.9 (d, J = 3.8 Hz), 20.8, 14.1, 14.0. **^{19}F NMR** (377 MHz, $CDCl_3$): δ -101.9 (dd, J = 254.9, 10.0 Hz), -115.6 (dd, J = 255.0, 26.2 Hz). **HRMS** (ESI): m/z calculated for $C_{18}H_{25}F_2NO_5$ requires 374.1774 for $[M+H]^+$, found 374.1779.

ethyl 3-(2,3-dihydrobenzo[b][1,4]dioxin-6-yl)-2,2-difluoro-3-(piperidin-1-yl)propanoate (3ai)

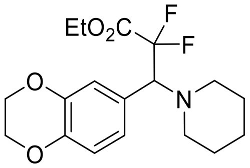

Procedure C was followed using CX-546 (**1ai**) (37.0 mg, 0.15 mmol, 1.0 equiv), TMDS (30.2 mg, 40 µL, 0.22 mmol, 1.5 equiv) and Vaska's catalyst (1.2 mg, 1.50 µmol, 1 mol%). Silica gel column chromatography (P/Et_2O/Et_3N 90:10:1 → 80:20:1) gave the desired product **3ai** as a colorless oil, 80% (42.7 mg, 0.12 mmol). **IR** (thin film): ν_{max} (cm^{-1}) = 2935, 2813, 1770, 1509, 1308, 1286, 1256, 1098, 1070, 921, 728, 707. **^{1}H NMR** (400 MHz, $CDCl_3$): δ 6.96 – 6.90 (m, 1H), 6.88 – 6.75 (m, 2H), 4.43 – 4.29 (m, 2H), 4.26 (s, 4H), 4.10 (dd, J = 26.5, 7.5 Hz, 1H), 2.71 – 2.61 (m, 2H), 2.33 – 2.19 (m, 2H), 1.61 – 1.43 (m, 4H), 1.37 (t, J = 7.1 Hz, 3H), 1.27 (p, J = 5.9 Hz, 2H). **^{13}C NMR** (101 MHz, $CDCl_3$): δ 164.7 (dd, J = 33.9, 30.2 Hz), 143.8, 143.1, 124.1 (d, J = 3.0 Hz), 123.8, 119.5 (d, J = 3.5 Hz), 117.5 (dd, J = 263.6, 254.1 Hz), 116.9, 70.6 (dd, J = 28.8, 18.9 Hz), 64.5, 64.4, 62.6, 51.7, 26.7, 24.1, 14.2. **^{19}F NMR** (377 MHz, $CDCl_3$): δ -102.8 (dd, J = 254.5, 8.4 Hz), -116.2 (dd, J = 254.8, 27.1 Hz). **HRMS** (ESI): m/z calculated for $C_{18}H_{23}F_2NO_4$ requires 356.1668 for $[M+H]^+$, found 356.1667.

benzyl 3-(azepan-1-yl)-2,2-difluoro-3-phenylpropanoate (3aj)

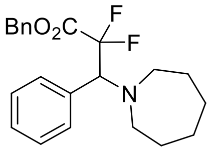

Procedure D was followed using (2-(benzyloxy)-1,1-difluoro-2-oxoethyl)zinc(II) bromide (**2b'**) (2.20 mL, 0.18 M in THF, 0.40 mmol, 2.6 equiv). Silica gel column chromatography (P/Et_2O/Et_3N 99:1:1 → 98:2:1) gave the desired product **3aj** as a colorless oil, 63% (36.2 mg, 96.9 µmol). **IR** (thin film): ν_{max} (cm^{-1}) = 2928, 2851, 1772, 1454, 1317, 1224, 1094, 1061, 698. **^{1}H NMR** (400 MHz, $CDCl_3$): δ 7.49 – 7.31 (m, 10H), 5.31 (s, 2H), 4.45 (dd, J = 26.7, 6.6 Hz, 1H), 2.95 – 2.85 (m, 2H), 2.55 – 2.44 (m, 2H), 1.60 – 1.31 (m, 8H). **^{13}C NMR** (126 MHz, $CDCl_3$): δ 164.6 (t, J = 32.9 Hz), 134.7, 133.0, 130.0, 128.9, 128.8, 128.8,

128.4, 128.2, 117.4 (t, J = 263.1, 257.0 Hz), 70.1 (dd, J = 28.0, 18.7 Hz), 68.3, 53.2 (d, J = 3.1 Hz), 29.4, 26.9. **^{19}F NMR** (377 MHz, $CDCl_3$): δ -101.4 (dd, J = 257.0, 7.4 Hz), -118.0 (dd, J = 257.1, 27.4 Hz). **HRMS** (ESI): m/z calculated for $C_{22}H_{25}F_2NO_2$ requires 374.1926 for $[M+H]^+$, found 374.1930.

2-(trimethylsilyl)ethyl 3-(azepan-1-yl)-2,2-difluoro-3-phenylpropanoate (3ak)

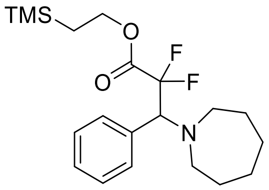

Procedure D was followed using (1,1-difluoro-2-oxo-2-(2-(trimethylsilyl)ethoxy)ethyl)zinc(II) bromide (**2c'**) (2.09 mL, 0.19 M in THF, 0.40 mmol, 2.6 equiv). Silica gel column chromatography (P/Et_2O/Et_3N 99:1:1) gave the desired product **3ak** as a colorless oil, 85% (49.5 mg, 0.13 mmol). **IR** (thin film): ν_{max} (cm^{-1}) = 2926, 2853, 1771, 1754, 1251, 1226, 1096, 1049, 858, 838, 727. **^{1}H NMR** (400 MHz, $CDCl_3$): δ 7.47 – 7.31 (m, 5H), 4.51 – 4.32 (m, 3H), 2.98 – 2.87 (m, 2H), 2.60 – 2.49 (m, 2H), 1.58 – 1.50 (m, 6H), 1.47 – 1.40 (m, 2H), 1.16 – 1.05 (m, 2H), 0.09 (s, 9H). **^{13}C NMR** (101 MHz, $CDCl_3$): δ 165.0 (dd, J = 33.8, 30.3 Hz), 133.2, 130.0 (d, J = 2.7 Hz), 128.3, 128.2, 117.4 (dd, J = 263.1, 255.8 Hz), 70.2 (dd, J = 28.1, 18.7 Hz), 65.2, 53.2 (d, J = 3.1 Hz), 29.5, 27.0, 17.5, -1.4. **^{19}F NMR** (377 MHz, $CDCl_3$): δ -101.9 (dd, J = 256.1, 7.7 Hz), -118.3 (dd, J = 256.6, 27.0 Hz). **HRMS** (ESI): m/z calculated for $C_{20}H_{31}F_2NO_2Si$ requires 384.2165 for $[M+H]^+$, found 384.2159.

isopropyl 3-(azepan-1-yl)-2,2-difluoro-3-phenylpropanoate (3al)

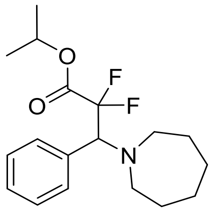

Procedure D (scale doubled in first step) was followed using (1,1-difluoro-2-isopropoxy-2-oxoethyl)zinc(II) bromide (**2d'**) (4.72 mL, 0.08 M in THF, 0.40 mmol, 2.6 equiv). Silica gel column chromatography (P/Et_2O/Et_3N 99:1:1) gave the desired product **3al** as a colorless oil, 84% (41.5 mg, 0.13 mmol). **IR** (thin film): ν_{max} (cm^{-1}) = 2929, 2853, 1769, 1752, 1300, 1090, 1061, 712, 701. **^{1}H NMR** (400 MHz, $CDCl_3$): δ 7.48 – 7.41 (m, 2H), 7.39 – 7.33 (m, 3H), 5.17 (hept, J = 6.3 Hz, 1H), 4.42 (dd, J = 26.1, 7.2 Hz, 1H), 2.98 – 2.88 (m, 2H), 2.59 – 2.49 (m, 2H), 1.63 – 1.39 (m, 8H), 1.36 (d, J = 6.3 Hz, 3H), 1.33 (d, J = 6.3 Hz, 3H). **^{13}C NMR** (101 MHz, $CDCl_3$): δ 164.2 (dd, J = 33.7, 30.1 Hz), 133.1, 130.1 (d, J = 2.7 Hz), 128.3, 128.2, 117.2 (dd, J = 262.2, 255.6 Hz), 70.9, 70.0 (dd, J = 27.9, 18.7 Hz), 53.2 (d, J = 3.1 Hz), 29.4, 27.0, 21.8, 21.7. **^{19}F NMR** (377 MHz, $CDCl_3$): δ -102.5 (dd, J = 256.9, 8.4 Hz), -117.7 (dd, J =

257.0, 26.8 Hz). **HRMS** (ESI): m/z calculated for $C_{18}H_{25}F_2NO_2$ requires 326.1926 for $[M+H]^+$, found 326.1925.

(1*R*,2*S*,5*R*)-2-isopropyl-5-methylcyclohexyl 3-(azepan-1-yl)-2,2-difluoro-3-phenylpropanoate (3am)

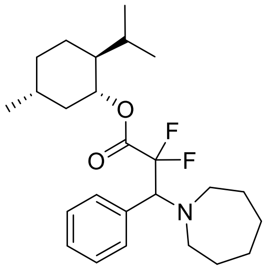

Procedure D was followed using (1,1-difluoro-2-(((1R,2S,5R)-2-isopropyl-5-methylcyclohexyl)oxy)-2-oxoethyl)zinc(II) bromide (**2e'**) (2.66 mL, 0.15 M in THF, 0.40 mmol, 2.6 equiv). Silica gel column chromatography (P/Et_2O/Et_3N 99:1:1) gave the desired product **3am** as a colorless oil, 49% (31.3 mg, 74.3 µmol) (55:45 mixture of diastereomers, based on ^1H NMR spectroscopic analysis). **IR** (thin film): ν_{max} (cm^{-1}) = 2955, 2928, 2869, 1768, 1750, 1454, 1299, 1091, 1062, 726, 701. **^{1}H NMR** (400 MHz, $CDCl_3$): δ 7.46 – 7.40 (m, 2H), 7.39 – 7.30 (m, 3H), 4.91 – 4.77 (m, 1H), 4.48 – 4.35 (m, 1H), 3.00 – 2.89 (m, 2H), 2.63 – 2.49 (m, 2H), 2.15 – 1.96 (m, 1H), 1.95 – 1.79 (m, 1H), 1.77 – 1.65 (m, 2H), 1.62 – 1.39 (m, 10H), 1.15 – 0.99 (m, 2H), 0.96 – 0.86 (m, 7H), 0.78 (d, J = 7.0 Hz, 1.7H), 0.72 (d, J = 6.9 Hz, 1.3H). **^{13}C NMR** (126 MHz, $CDCl_3$): δ 164.7 – 163.6 (m) (overlapped), 133.0, 133.0, 130.3 – 129.8 (m) (overlapped), 128.2, 128.1, 128.0 (overlapped), 119.3 – 114.9 (m) (overlapped), 70.3 – 69.3 (m) (overlapped), 53.0 (d, J = 3.0 Hz), 52.9 (d, J = 3.1 Hz), 46.9, 46.8, 40.6, 40.4, 34.1, 34.0, 31.4, 31.4, 29.1 (overlapped), 27.0, 26.9, 26.0, 25.9, 23.3 (overlapped), 22.0, 21.9, 20.8, 20.7, 16.2, 16.1. **^{19}F NMR** (377 MHz, $CDCl_3$): δ -101.2 – -104.7 (m), -112.8 – -117.1 (m). **HRMS** (ESI): m/z calculated for $C_{25}H_{37}F_2NO_2$ requires 422.2865 for $[M+H]^+$, found 422.2862.

(2,2-dimethyl-1,3-dioxolan-4-yl)methyl 3-(azepan-1-yl)-2,2-difluoro-3-phenylpropanoate (3an)

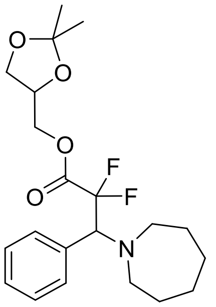

Procedure D was followed using (2-((2,2-dimethyl-1,3-dioxolan-4-yl)methoxy)-1,1-difluoro-2-oxoethyl)zinc(II) bromide (**2f'**) (2.54 mL, 0.16 M in THF, 0.40 mmol, 2.6 equiv). Silica gel column chromatography (P/Et_2O/Et_3N 95:5:1 → 90:10:1) gave the desired product **3an** as a colorless oil, 64% (38.5 mg, 96.9 µmol) (1:1 mixture of diastereomers, based on ^1H NMR spectroscopic analysis). **IR** (thin

film): ν_{max} (cm^{-1}) = 2944, 2967, 1669, 1380, 1209, 1184, 1053, 705. **^{1}H NMR** (400 MHz, $CDCl_3$): δ 7.48 – 7.40 (m, 2H), 7.39 – 7.32 (m, 3H), 4.51 – 4.35 (m, 2H), 4.34 – 4.27 (m, 2H), 4.17 – 4.06 (m, 1H), 3.81 (dd, J = 5.7, 1.6 Hz, 0.5H), 3.79 (dd, J = 5.7, 1.6 Hz, 0.5H), 2.97 – 2.87 (m, 2H), 2.64 – 2.50 (m, 2H), 1.58 – 1.48 (m, 6H), 1.47 – 1.45 (m, 3H), 1.44 – 1.40 (m, 2H), 1.38 (s, 3H). **^{13}C NMR** (101 MHz, $CDCl_3$): δ 165.0 – 164.2 (m) (overlapped), 132.9 (overlapped), 130.0, 130.0, 128.4 (overlapped), 128.3 (overlapped), 120.1 – 114.7 (m) (overlapped), 110.2, 110.2, 73.2, 73.2, 70.2 (dd, J = 28.0, 18.5 Hz) (overlapped), 66.7, 66.5, 66.4, 66.4, 53.2 (overlapped), 29.5, 29.5, 27.0, 27.0, 26.8 (overlapped), 25.5, 25.5. **^{19}F NMR** (377 MHz, $CDCl_3$): δ -101.5 (dt, *J* = 257.5, 8.1 Hz), -117.9 (ddd, *J* = 257.4, 65.0, 27.0 Hz). **HRMS** (ESI): m/z calculated for $C_{21}H_{29}F_2NO_4$ requires 398.2137 for $[M+H]^+$, found 398.2137.

benzhydryl 3-(azepan-1-yl)-2,2-difluoro-3-phenylpropanoate (3ao)

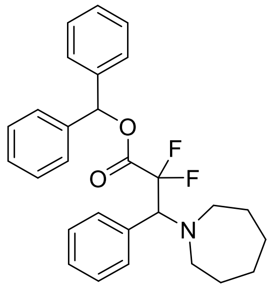

Procedure D was followed using (2-(benzhydryloxy)-1,1-difluoro-2-oxoethyl)zinc(II) bromide (**2g'**) (2.89 mL, 0.14 M in THF, 0.40 mmol, 2.6 equiv). Silica gel column chromatography (P/Et_2O/Et_3N 99:1:1 → 98:2:1) gave the desired product **3ao** as a pale yellow oil, 74% (51.0 mg, 0.11 mmol). **IR** (thin film): ν_{max} (cm^{-1}) = 2928, 2856, 1772, 1674, 1453, 1185, 1087, 1045, 744, 700. **^{1}H NMR** (400 MHz, $CDCl_3$): δ 7.46 – 7.28 (m, 14H), 7.19 – 7.08 (m, 1H), 7.04 (s, 1H), 4.48 (dd, J = 26.2, 6.8 Hz, 1H), 2.91 – 2.80 (m, 2H), 2.46 – 2.36 (m, 2H), 1.39 – 1.30 (m, 6H), 1.29 – 1.16 (m, 2H). **^{13}C NMR** (101 MHz, $CDCl_3$): δ 163.8 (dd, *J* = 34.5, 30.6 Hz), 139.2 (d, *J* = 10.1 Hz), 133.1, 130.0 (d, *J* = 2.5 Hz), 128.8, 128.7, 128.5, 128.3, 128.3, 128.3, 128.2, 127.6, 127.2, 117.4 (dd, *J* = 262.5, 256.1 Hz), 79.2, 70.0 (dd, *J* = 27.5, 18.6 Hz), 53.1, 29.1, 26.9. **^{19}F NMR** (377 MHz, $CDCl_3$): δ -102.5 (dd, J = 256.9, 8.4 Hz), -117.7 (dd, J = 257.0, 26.8 Hz). **HRMS** (ESI): m/z calculated for $C_{28}H_{29}F_2NO_2$ requires 450.2239 for $[M+H]^+$, found 450.2236.

3-(azepan-1-yl)-N,N-diethyl-2,2-difluoro-3-phenylpropanamide (3ap)

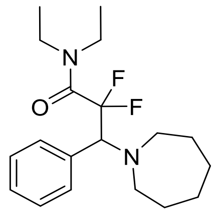

Procedure D was followed using (2-(diethylamino)-1,1-difluoro-2-oxoethyl)zinc(II) bromide (**2h'**) (1.65 mL, 0.24 M in THF, 0.40 mmol, 2.6 equiv). Silica gel column chromatography (P/Et_2O/Et_3N 97:3:1

→ 95:5:1) gave the desired product **3ap** as a colorless oil, 95% (49.0 mg, 0.14 mmol). **IR** (thin film): ν_{max} (cm^{-1}) = 2927, 2852, 1655, 1451, 1174, 1081, 1036, 803, 728, 701. **^{1}H NMR** (400 MHz, $CDCl_3$): δ 7.45 – 7.37 (m, 2H), 7.36 – 7.27 (m, 3H), 4.73 (dd, J = 23.7, 9.2 Hz, 1H), 3.67 – 3.55 (m, 1H), 3.52 – 3.40 (m, 2H), 3.39 – 3.26 (m, 1H), 2.97 – 2.87 (m, 2H), 2.69 – 2.59 (m, 2H), 1.65 – 1.40 (m, 8H), 1.26 (t, J = 7.0 Hz, 3H), 1.15 (t, J = 7.1 Hz, 3H). **^{13}C NMR** (126 MHz, $CDCl_3$): δ 163.7 (t, J = 28.3 Hz), 134.9, 129.9, 128.1, 127.6, 120.0 (dd, J = 264.5, 259.7 Hz), 70.2 (dd, J = 25.8, 19.2 Hz), 53.7, 42.6, 29.6, 27.1, 14.9, 12.4. **^{19}F NMR** (377 MHz, $CDCl_3$): δ -98.4 (dd, J = 267.6, 10.7 Hz), -110.1 (dd, J = 267.9, 25.2 Hz). **HRMS** (ESI): m/z calculated for $C_{19}H_{28}F_2N_2O$ requires 339.2242 for $[M+H]^+$, found 339.2241.

3-(azepan-1-yl)-2,2-difluoro-1-(4-methylpiperidin-1-yl)-3-phenylpropan-1-one (3aq)

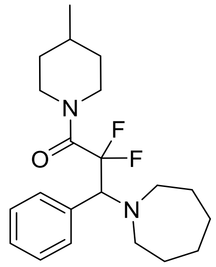

Procedure D was followed using (1,1-difluoro-2-(4-methylpiperidin-1-yl)-2-oxoethyl)zinc(II) bromide (**2i'**) (2.36 mL, 0.17 M in THF, 0.40 mmol, 2.6 equiv). Silica gel column chromatography (P/Et_2O/Et_3N 99:1:1 → 98:2:1) gave the desired product **3aq** as a colorless oil, 97% (54.0 mg, 0.15 mmol). **IR** (thin film): ν_{max} (cm^{-1}) = 2924, 2852, 1666, 1450, 1175, 1082, 1045, 970, 730, 701. **^{1}H NMR** (400 MHz, $CDCl_3$): δ 7.44 – 7.38 (m, 2H), 7.37 – 7.28 (m, 3H), 4.73 – 4.44 (m, 2H), 4.38 – 4.28 (m, 1H), 3.14 – 2.99 (m, 1H), 2.98 – 2.88 (m, 2H), 2.72 – 2.61 (m, 3H), 1.79 – 1.60 (m, 3H), 1.59 – 1.42 (m, 8H), 1.26 – 1.09 (m, 2H), 0.96 (d, J = 6.4 Hz, 3H). **^{13}C NMR** (126 MHz, $CDCl_3$): δ 162.5 (t, J = 27.8 Hz), 134.9 (d, J = 29.5 Hz), 129.9 (d, J = 9.4 Hz), 128.1, 127.7, 119.7 (td, J = 261.2, 12.1 Hz), 70.7 (dt, J = 82.6, 22.3 Hz), 53.4 (d, J = 34.0 Hz), 46.5 (d, J = 10.5 Hz), 44.4, 34.9 (d, J = 7.0 Hz), 34.0, 31.2, 29.6, 27.2 (d, J = 7.7 Hz), 21.8. **^{19}F NMR** (377 MHz, $CDCl_3$): δ -98.5 (td, J = 271.0, 270.3, 12.3 Hz), -106.2 (ddd, J = 269.0, 167.6, 24.3 Hz). **HRMS** (ESI): m/z calculated for $C_{21}H_{30}F_2N_2O$ requires 365.2399 for $[M+H]^+$, found 365.2399.

3-(azepan-1-yl)-2,2-difluoro-1-morpholino-3-phenylpropan-1-one (3ar)

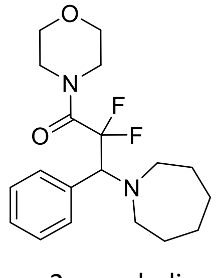

Procedure D was followed using (1,1-difluoro-2-morpholino-2-oxoethyl)zinc(II) bromide (**2j'**) (1.05 mL, 0.38 M in THF, 0.40 mmol, 2.6 equiv). Silica gel column chromatography (P/Et_2O/Et_3N 95:5:1 →

90:10:1) gave the desired product **3ar** as a colorless oil, 63% (33.7 mg, 95.6 µmol). **IR** (thin film): ν_{max} (cm^{-1}) = 2923, 2853, 1670, 1440, 1173, 1117, 1080, 1045, 1009, 730, 702. **^{1}H NMR** (400 MHz, $CDCl_3$): δ 7.44 – 7.38 (m, 2H), 7.38 – 7.27 (m, 3H), 4.61 (dd, J = 22.5, 11.2 Hz, 1H), 3.90 – 3.52 (m, 8H), 3.01 – 2.86 (m, 2H), 2.79 – 2.59 (m, 2H), 1.67 – 1.45 (m, 8H). **^{13}C NMR** (126 MHz, $CDCl_3$): δ 163.0 (t, J = 28.4 Hz), 134.5, 129.9, 128.3, 127.9, 119.7 (t, J = 262.2 Hz), 72.2 – 69.9 (m), 67.0, 53.6, 47.0, 43.9, 29.5, 27.2. **^{19}F NMR** (377 MHz, $CDCl_3$): δ -98.2 (dd, J = 269.6, 11.9 Hz), -106.9 (dd, J = 269.6, 22.7 Hz). **HRMS** (ESI): m/z calculated for $C_{19}H_{26}F_2N_2O_2$ requires 353.2035 for $[M+H]^+$, found 353.2036.

benzyl 2-(1-benzylazepan-2-yl)-2,2-difluoroacetate (3as)

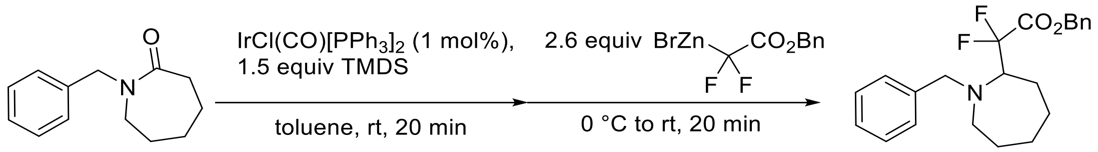

Step 1, formation of organozinc reagent: An oven-dried 10 mL round bottom-flask, equipped with a magnetic stirring bar and a septum, was three times vacuumed and backfilled with Argon. Zinc powder (192 mg, 2.94 mmol, 1.5 equiv) was added and suspended in 3.3 mL anhydrous THF (0.6 M). TMSCl (63.9 mg, 75 µL, 0.59 mmol, 30 mol%) was added and the reaction mixture was stirred at room temperature for 15 min. Then benzyl 2-bromo-2,2-difluoroacetate **2b** (520 mg, 1.96 mmol, 1.0 equiv) was added dropwise to the solution and significant exotherm was usually observed after the addition. The mixture was allowed to cool to room temperature by stirring an additional 20 min and was directly used in the next step. The concentration of the corresponding organozinc reagent **2b'** was determined according to a literature report by titration with I_2 to be 0.18 mol/L.[13]

Step 2, reductive functionalization of amides: An oven-dried 10 mL round bottom-flask, equipped with a magnetic stirring bar and a septum, was three times vacuumed and backfilled with Argon. The flask was then initially charged with Vaska's catalyst (1.2 mg, 1.52 µmol, 2 mol%), 1-benzylazepan-2-one (**1ab**) (31.0 mg, 0.15 mmol, 1.0 equiv) and 1.5 mL anhydrous toluene (0.1 M). TMDS (30.7 mg, 40 µL, 0.23 mmol, 1.5 equiv) was added and the resulting mixture was stirred for 20 min. The solution was cooled to 0 °C and the freshly prepared organozinc solution **2b'** (2.20 mL, 0.18 M in THF, 0.40 mmol, 2.6 equiv) was added, followed by an additional stirring for 20 min at room temperature. Then the reaction mixture was quenched with a saturated, aqueous NH_4Cl-solution (10 mL) and extracted with DCM (3 x 5 mL). The combined organic phases were washed with brine (10 mL), dried over Na_2SO_4, filtered and concentrated *in vacuo*. Silica gel column chromatography (P/Et_2O/Et_3N 99:1:1 → 98:2:1) gave the desired product **3as** as a colorless oil, 42% (24.1 mg, 64.5 µmol). **IR** (thin film): ν_{max} (cm^{-1}) = 2989, 2855, 1771, 1455, 1291, 1211, 1124, 1076, 1055, 748, 731, 698. **^{1}H NMR** (400 MHz, $CDCl_3$): δ 7.35 – 7.04 (m, 10H), 5.15 (d, J = 2.0 Hz, 2H), 4.10 (d, J = 13.8 Hz, 1H), 3.70 (d, J = 13.8 Hz, 1H), 3.49 – 3.32 (m, 1H), 2.70 – 2.59 (m, 1H), 2.55 – 2.44 (m, 1H), 2.05 – 1.94 (m, 1H), 1.86 – 1.72 (m, 2H), 1.73 –

1.63 (m, 1H), 1.61 – 1.51 (m, 1H), 1.50 – 1.39 (m, 1H), 1.36 – 1.21 (m, 2H). **^{13}C NMR** (126 MHz, $CDCl_3$): δ 164.8 (dd, J = 34.9, 32.0 Hz), 139.3, 134.6, 128.8, 128.8, 128.7, 128.5, 128.4, 127.0, 119.4 – 115.0 (m), 68.2, 65.3 (dd, J = 25.1, 21.8 Hz), 56.9, 47.0, 28.9, 26.0, 25.8, 24.7. **^{19}F NMR** (377 MHz, $CDCl_3$): δ -98.2 – -110.2 (m), -120.5 (dd, J = 254.9, 25.2 Hz). **HRMS** (ESI): m/z calculated for $C_{22}H_{25}F_2NO_2$ requires 374.1926 for $[M+H]^+$, found 374.1929.

2-(1-benzylazepan-2-yl)-*N,N*-diethyl-2,2-difluoroacetamide (3at)

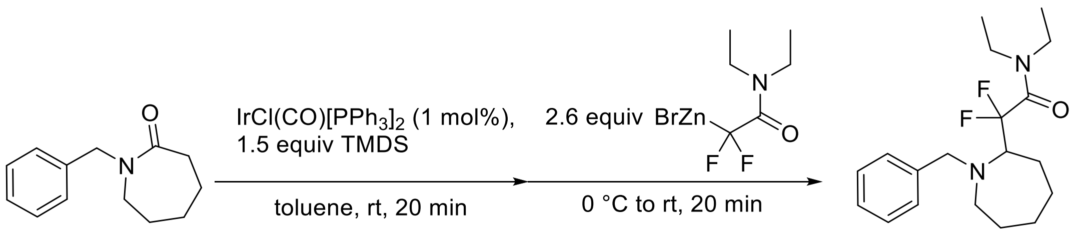

Step 1, formation of organozinc reagent: An oven-dried 10 mL round bottom-flask, equipped with a magnetic stirring bar and a septum, was three times vacuumed and backfilled with Argon. Zinc powder (193 mg, 2.95 mmol, 1.5 equiv) was added and suspended in 3.3 mL anhydrous THF (0.6 M). TMSCl (64.0 mg, 75 µL, 0.59 mmol, 30 mol%) was added and the reaction mixture was stirred at room temperature for 15 min. Then 2-bromo-*N,N*-diethyl-2,2-difluoroacetamide **2h** (452 mg, 1.96 mmol, 1.0 equiv) was added dropwise to the solution and significant exotherm was usually observed after the addition. The mixture was allowed to cool to room temperature by stirring an additional 20 min and was directly used in the next step. The concentration of the corresponding organozinc reagent **2h'** was determined according to a literature report by titration with I_2 to be 0.24 mol/L.[13]

Step 2, reductive functionalization of amides: An oven-dried 10 mL round bottom-flask, equipped with a magnetic stirring bar and a septum, was three times vacuumed and backfilled with Argon. The flask was then initially charged with Vaska's catalyst (1.2 mg, 1.52 µmol, 2 mol%), 1-benzylazepan-2-one (**1ab**) (31.0 mg, 0.15 mmol, 1.0 equiv) and 1.5 mL anhydrous toluene (0.1 M). TMDS (30.7 mg, 40 µL, 0.23 mmol, 1.5 equiv) was added and the resulting mixture was stirred for 20 min. The solution was cooled to 0 °C and the freshly prepared organozinc solution **2h'** (1.65 mL, 0.24 M in THF, 0.40 mmol, 2.6 equiv) was added, followed by an additional stirring for 20 min at room temperature. Then the reaction mixture was quenched with a saturated, aqueous NH_4Cl-solution (10 mL) and extracted with DCM (3 x 5 mL). The combined organic phases were washed with brine (10 mL), dried over Na_2SO_4, filtered and concentrated *in vacuo*. Silica gel column chromatography ($P/Et_2O/Et_3N$ 99:1:1 → 98:2:1) gave the desired product **3at** as a colorless oil, 53% (27.5 mg, 81.3 µmol). **IR** (thin film): ν_{max} (cm^{-1}) = 2925, 2853, 1652, 1462, 1364, 1175, 1044, 1031, 727, 699. **^{1}H NMR** (400 MHz, $CDCl_3$): δ 7.28 – 7.26 (m, 4H), 7.24 – 7.17 (m, 1H), 4.19 (d, J = 13.7 Hz, 1H), 3.95 (d, J = 13.7 Hz, 1H), 3.68 – 3.55 (m, 1H), 3.53 – 3.40 (m, 3H), 3.39 – 3.28 (m, 1H), 2.91 – 2.79 (m, 1H), 2.76 – 2.66 (m, 1H), 2.17 – 2.06 (m, 1H), 1.97 –

1.87 (m, 1H), 1.86 – 1.77 (m, 1H), 1.77 – 1.66 (m, 2H), 1.56 – 1.39 (m, 1H), 1.35 – 1.24 (m, 2H), 1.24 – 1.12 (m, 6H). **^{13}C NMR** (126 MHz, $CDCl_3$): δ 164.1 (t, J = 28.6 Hz), 140.0, 128.7, 128.2, 126.8, 119.8 (dd, J = 261.6, 258.3 Hz), 65.7 (dd, J = 24.0, 21.1 Hz), 58.4, 46.5, 42.6 (dd, J = 9.5, 4.5 Hz), 42.5, 29.7, 27.5, 26.0, 24.5, 14.9, 12.6. **^{19}F NMR** (377 MHz, $CDCl_3$): δ -103.2 (dd, J = 262.8, 11.5 Hz), -112.8 (dd, J = 262.4, 19.7 Hz). **HRMS** (ESI): m/z calculated for $C_{19}H_{28}F_2N_2O$ requires 339.2242 for $[M+H]^+$, found 339.2234.

Large scale synthesis of ethyl 3-(dimethylamino)-2,2-difluoro-3-phenylpropanoate (3b)

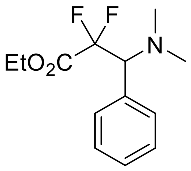

Step 1, formation of the organozinc reagent: An oven-dried 250 mL round bottom-flask, equipped with a magnetic stirring bar and a septum, was three times vacuumed and backfilled with Argon. Zinc powder (4.83 g, 73.9 mmol, 1.5 equiv) was added and suspended in 82 mL anhydrous THF (0.6 M). TMSCl (1.61 g, 1.88 mL, 14.8 mmol, 30 mol%) was added and the reaction mixture was stirred at room temperature for 15 min. Then ethyl bromodifluoroacetate **2a** (10.0 g, 6.33 mL, 49.3 mmol, 1.0 equiv) was added dropwise to the solution and significant exotherm was usually observed after the addition. The mixture was allowed to cool to room temperature by stirring an additional 20 min and was directly used in the next step. The concentration of the corresponding organozinc reagent **2a'** was determined according to a literature report by titration with I_2 to be 0.24 mol/L.[13]

Step 2, reductive functionalization of amides: An oven-dried 250 mL round bottom-flask, equipped with a magnetic stirring bar and a septum, was three times vacuumed and backfilled with Argon. The flask was then initially charged with Vaska's catalyst (52.3 mg, 67.0 µmol, 1 mol%), *N,N*-dimethylbenzamide (**1b**) (1.00 g, 6.70 mmol, 1.0 equiv) and 67 mL anhydrous toluene (0.1 M). TMDS (1.35 g, 1.78 mL, 10.1 mmol, 1.5 equiv) was added and the resulting mixture was stirred for 20 min. The solution was cooled to 0 °C and the freshly prepared organozinc solution **2a'** (72.6 mL, 0.24 M in THF, 17.4 mmol, 2.6 equiv) was added, followed by an additional stirring for 20 min at room temperature. Then the reaction mixture was quenched with a saturated, aqueous NH_4Cl-solution (100 mL) and extracted with DCM (3 x 50 mL). The combined organic phases were washed with brine (100 mL), dried over Na_2SO_4, filtered and concentrated *in vacuo*. Silica gel column chromatography ($P/Et_2O/Et_3N$ 98:2:1 → 97:3:1) gave the desired product **3b** as a colorless oil, 72% (28.4 mg, 0.11 mmol). **IR** (thin film): ν_{max} (cm^{-1}) = 2962, 2924, 2851, 1771, 1669, 1460, 1375, 1313, 1151, 1103, 803, 704. **^{1}H NMR** (400 MHz, $CDCl_3$): δ 7.38 (s, 5H), 4.35 – 4.16 (m, 3H), 2.27 (s, 6H), 1.28 (t, *J* = 7.1 Hz, 3H). **^{13}C NMR** (126 MHz, $CDCl_3$): δ 164.3 (dd, *J* = 33.3, 30.5 Hz), 130.8 (d, *J* = 2.6 Hz), 130.3, 128.7, 128.4, 117.0 (dd, *J* = 261.3, 255.7 Hz), 70.1 (dd, *J* = 26.8, 19.7 Hz), 62.7, 42.9, 14.0. **^{19}F NMR** (377 MHz, $CDCl_3$):

δ -105.9 (dd, *J* = 258.2, 9.7 Hz), -113.9 (dd, *J* = 256.4, 23.4 Hz). **HRMS** (ESI): m/z calculated for $C_{13}H_{17}F_2NO_2$ requires 258.1300 for $[M+H]^+$, found 258.1301.

3-(dimethylamino)-2,2-difluoro-3-phenylpropan-1-ol (6)

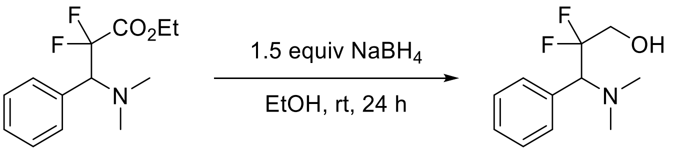

Ethyl 3-(dimethylamino)-2,2-difluoro-3-phenylpropanoate **3b** (40.0 mg, 0.16 mmol, 1.0 equiv) was added to an oven-dried 5 mL round bottom-flask, equipped with a magnetic stirring bar and a septum. The flask was three times vacuumed and backfilled with Argon and 0.91 mL of anhydrous ethanol was added, followed by sodium borohydride (8.8 mg, 0.23 mmol, 1.5 equiv). The reaction mixture was stirred for 24 h at room temperature, then quenched with a saturated, aqueous NH_4Cl-solution (5 mL) and extracted with DCM (3 x 5 mL). The combined organic phases were washed with brine (5 mL), dried over Na_2SO_4, filtered and concentrated *in vacuo*. Silica gel column chromatography (P/EA 3:2 → 2:3) gave primary alcohol **6** as a colorless solid, 81% (27.0 mg, 0.13 mmol). **m.p.**: 40–42 °C. **IR** (thin film): ν_{max} (cm^{-1}) = 3365, 2946, 2873, 2835, 2790, 1475, 1167, 1080, 1064, 757, 704. ^{1}**H NMR** (400 MHz, $CDCl_3$): δ 7.39 (s, 5H), 4.75 (s, 1H), 4.11 – 3.81 (m, 3H), 2.29 (s, 6H). ^{13}**C NMR** (126 MHz, $CDCl_3$): δ 130.9 (d, J = 3.2 Hz), 130.0, 128.6, 128.3, 121.4 (dd, J = 254.4, 245.7 Hz), 71.8 (dd, J = 30.0, 20.8 Hz), 66.2 (dd, J = 33.5, 30.3 Hz), 43.0 – 42.8 (m). ^{19}**F NMR** (377 MHz, $CDCl_3$): δ -103.2 – -106.0 (m), -112.7 – -115.5 (m). **HRMS** (ESI): m/z calculated for $C_{11}H_{15}F_2NO$ requires 216.1194 for $[M+H]^+$, found 216.1195.

3-(dimethylamino)-2,2-difluoro-3-phenylpropanamide (7)

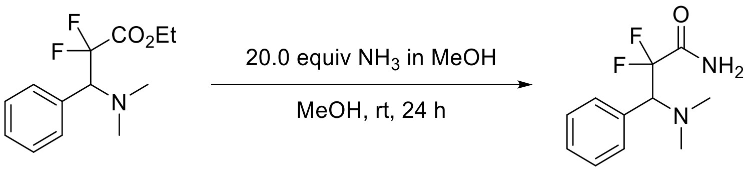

Ethyl 3-(dimethylamino)-2,2-difluoro-3-phenylpropanoate **3b** (40.0 mg, 0.16 mmol, 1.0 equiv) was dissolved in 0.62 mL methanol and an ammonia solution (0.44 mL, 7.00 N in MeOH, 3.11 mmol, 20.0 equiv) was added. The reaction mixture was stirred for 24 h at room temperature, then quenched with a saturated, aqueous NH_4Cl-solution (5 mL) and extracted with DCM (3 x 5 mL). The combined organic phases were washed with brine (5 mL), dried over Na_2SO_4, filtered and concentrated *in vacuo*. Silica gel column chromatography (P/EA 2:3 → 3:7) gave primary amide **7** as a colorless solid, 85% (30.0 mg, 0.13 mmol). **m.p.**: 120–122 °C. **IR** (thin film): ν_{max} (cm^{-1}) = 3341, 3194, 2948, 2875, 2836, 2792, 1703, 1604, 1220, 1185, 1111, 1043, 752, 703. ^{1}**H NMR** (400 MHz, $CDCl_3$): δ 7.37 (s, 5H), 6.75 (s, 1H), 6.24 (s, 1H), 4.32 (dd, J = 25.0, 7.9 Hz, 1H), 2.27 (s, 6H). ^{13}**C NMR** (126 MHz, $CDCl_3$): δ 166.7 (dd, J = 30.1, 28.0

Hz), 130.6, 130.6, 128.6, 128.4, 117.9 (dd, J = 262.9, 256.7 Hz), 69.0 (dd, J = 26.7, 19.4 Hz), 43.4 – 43.1 (m). **^{19}F NMR** (377 MHz, $CDCl_3$): δ -105.2 (dd, J = 261.0, 9.7 Hz), -115.0 (dd, J = 261.1, 25.8 Hz). **HRMS** (ESI): m/z calculated for $C_{11}H_{14}F_2N_2O$ requires 229.1147 for $[M+H]^+$, found 229.1149.

3-(dimethylamino)-2,2-difluoro-1,1,3-triphenylpropan-1-ol (8)

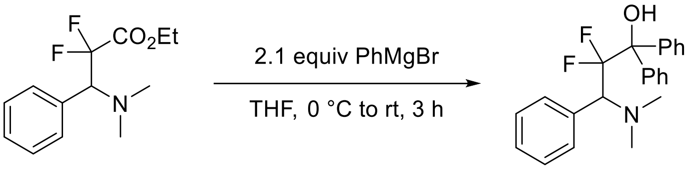

An oven-dried 5 mL round bottom-flask, equipped with a magnetic stirring bar and a septum, was three times vacuumed and backfilled with Argon. Ethyl 3-(dimethylamino)-2,2-difluoro-3-phenylpropanoate **3b** (40.0 mg, 0.16 mmol, 1.0 equiv) was dissolved in 0.62 mL anhydrous THF and cooled to 0 °C. A phenylmagnesium bromide solution (0.11 mL, 3.00 M in Et_2O, 0.33 mmol, 2.1 equiv) was added dropwise. The reaction mixture was allowed to warm to room temperature and stirred for 3 h, then quenched with a saturated, aqueous NH_4Cl-solution (5 mL) and extracted with DCM (3 x 5 mL). The combined organic phases were washed with brine (5 mL), dried over Na_2SO_4, filtered and concentrated *in vacuo*. Silica gel column chromatography (P/Et_2O 95:5 → 9:1) gave tertiary alcohol **8** as a colorless oil, 61% (34.7 mg, 94.4 µmol). **IR** (thin film): ν_{max} (cm^{-1}) = 3060, 2882, 2844, 1495, 1453, 1067, 754, 739, 702. **^{1}H NMR** (400 MHz, $CDCl_3$): δ 8.64 (s, 1H), 7.76 – 7.68 (m, 2H), 7.64 – 7.56 (m, 2H), 7.38 – 7.11 (m, 11H), 4.02 (d, J = 30.4 Hz, 1H), 2.09 (s, 6H). **^{13}C NMR** (126 MHz, $CDCl_3$): δ 143.9 (dd, J = 5.0, 2.2 Hz), 140.9, 131.2 (d, J = 3.6 Hz), 120.0 – 128.7 (m), 128.7, 128.5, 128.2, 128.2 – 127.9 (m), 127.8, 127.8, 127.5, 127.0 (d, J = 4.8 Hz), 121.9 (dd, J = 265.0, 256.8 Hz), 81.9 (t, J = 24.1 Hz), 69.9 (dd, J = 31.7, 20.6 Hz), 42.2. **^{19}F NMR** (377 MHz, $CDCl_3$): δ -99.0 (d, J = 267.6 Hz), -116.8 (dd, J = 267.6, 30.7 Hz). **HRMS** (ESI): m/z calculated for $C_{23}H_{23}F_2NO$ requires 368.1820 for $[M+H]^+$, found 368.1822.

3-(dimethylamino)-2,2-difluoro-3-phenylpropanoic acid hydrochloride (9)

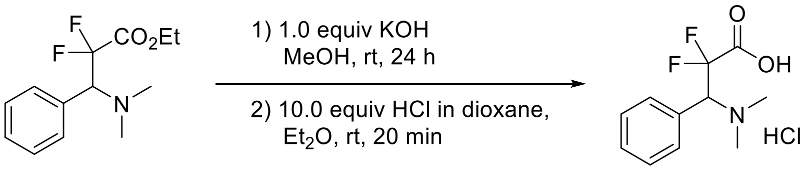

Potassium hydroxide (52 mg, 0.79 mmol, 1.0 equiv) was dissolved in 0.72 mL methanol. Ethyl 3-(dimethylamino)-2,2-difluoro-3-phenylpropanoate **3b** (203 mg, 0.79 mmol, 1.0 equiv) was added and the resulting reaction mixture was stirred over night at room temperature. The solvent was removed *in vacuo* and the remaining colorless solid was suspended in 10 mL of diethyl ether. A HCl solution (1.97 mL, 4.00 M in dioxane, 7.88 mmol, 10.0 equiv) was added and the suspension was stirred for 20 min at room temperature, then filtered and rinsed with diethyl ether. Drying under vacuum furnished

acid **9** as a colorless solid, quant. (209 mg, 0.79 mmol). **m.p.**: >260 °C. **IR** (thin film): ν_{max} (cm^{-1}) = 2968, 2888, 2583, 2359, 2160, 1728, 1460, 1229, 1116, 1072, 768, 755, 700, 685. **^{1}H NMR** (400 MHz, D_2O): δ 7.67 – 7.50 (m, 5H), 5.04 (dd, J = 19.3, 7.7 Hz, 1H), 3.14 (s, 3H), 2.82 (s, 3H). **^{13}C NMR** (126 MHz, D_2O): δ 165.8 (t, J = 26.4 Hz), 131.3, 130.3, 129.6, 126.4 (d, J = 6.5 Hz), 114.9 (dd, J = 263.8, 258.8 Hz), 70.8 (dd, J = 24.3, 20.5 Hz), 43.9, 42.6. **^{19}F NMR** (377 MHz, D_2O): δ -99.2 (dd, J = 248.9, 9.1 Hz), -112.0 (dd, J = 249.6, 20.2 Hz). **HRMS** (ESI): m/z calculated for $C_{11}H_{13}F_2NO_2$ requires 230.0987 for $[M+H]^+$, found 230.0986.

3-ethoxy-2,2-difluoro-*N,N*-dimethyl-1-phenylbut-3-en-1-amine (10)

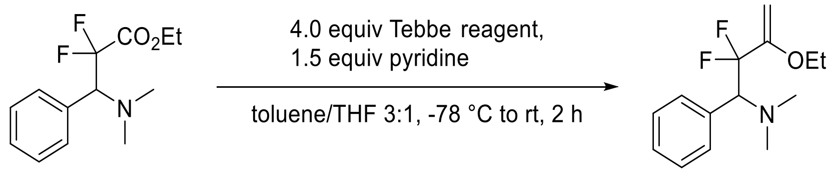

An oven-dried 50 mL round bottom-flask, equipped with a magnetic stirring bar and a septum, was three times vacuumed and backfilled with Argon. Ethyl 3-(dimethylamino)-2,2-difluoro-3-phenylpropanoate **3b** (100 mg, 0.39 mmol, 1.0 equiv) and pyridine (46.1 mg, 47 µL, 0.58 mmol, 1.5 equiv) were dissolved in 17.3 mL anhydrous toluene/THF 3:1 and cooled to -78 °C. A solution of the Tebbe reagent (3.11 mL, 0.50 M in THF, 1.55 mmol, 4.0 equiv) was added dropwise. The reaction mixture was allowed to warm to room temperature and stirred for 2 h, then quenched with an aqueous 1 N NaOH solution (20 mL) and extracted with Et_2O (3 x 15 mL). The combined organic phases were washed with brine (20 mL), dried over Na_2SO_4, filtered and concentrated *in vacuo*. Column chromatography on basic alumina (Brockmann I) (P/EA 9:1) gave enolether **10** as a colorless oil, 42% (42.0 mg, 0.16 mmol). **IR** (thin film): ν_{max} (cm^{-1}) = 2940, 2830, 2787, 1644, 1315, 1215, 1181, 1096, 1066, 1030, 824, 753, 702. **^{1}H NMR** (400 MHz, $CDCl_3$): δ 7.38 – 7.26 (m, 5H), 4.59 (d, J = 3.1 Hz, 1H), 4.20 – 4.06 (m, 2H), 3.83 – 3.46 (m, 2H), 2.30 (s, 6H), 1.27 (t, J = 7.0 Hz, 3H). **^{13}C NMR** (101 MHz, $CDCl_3$): δ 155.9 (t, J = 28.0 Hz), 132.8 (d, J = 3.2 Hz), 130.6 (t, J = 2.0 Hz), 128.0, 127.9, 120.0 (t, J = 249.5 Hz), 84.9 (t, J = 5.9 Hz), 70.1 (dd, J = 25.1, 22.4 Hz), 63.8, 43.6, 14.3. **^{19}F NMR** (377 MHz, $CDCl_3$): δ -105.1 (dd, J = 254.2, 18.6 Hz), -107.9 (dd, J = 254.3, 18.1 Hz). **HRMS** (ESI): m/z calculated for $C_{14}H_{19}F_2NO$ requires 256.1507 for $[M+H]^+$, found 256.1509.

6. NMR Spectra

1ac – ^{1}H NMR (400 MHz, $CDCl_3$)

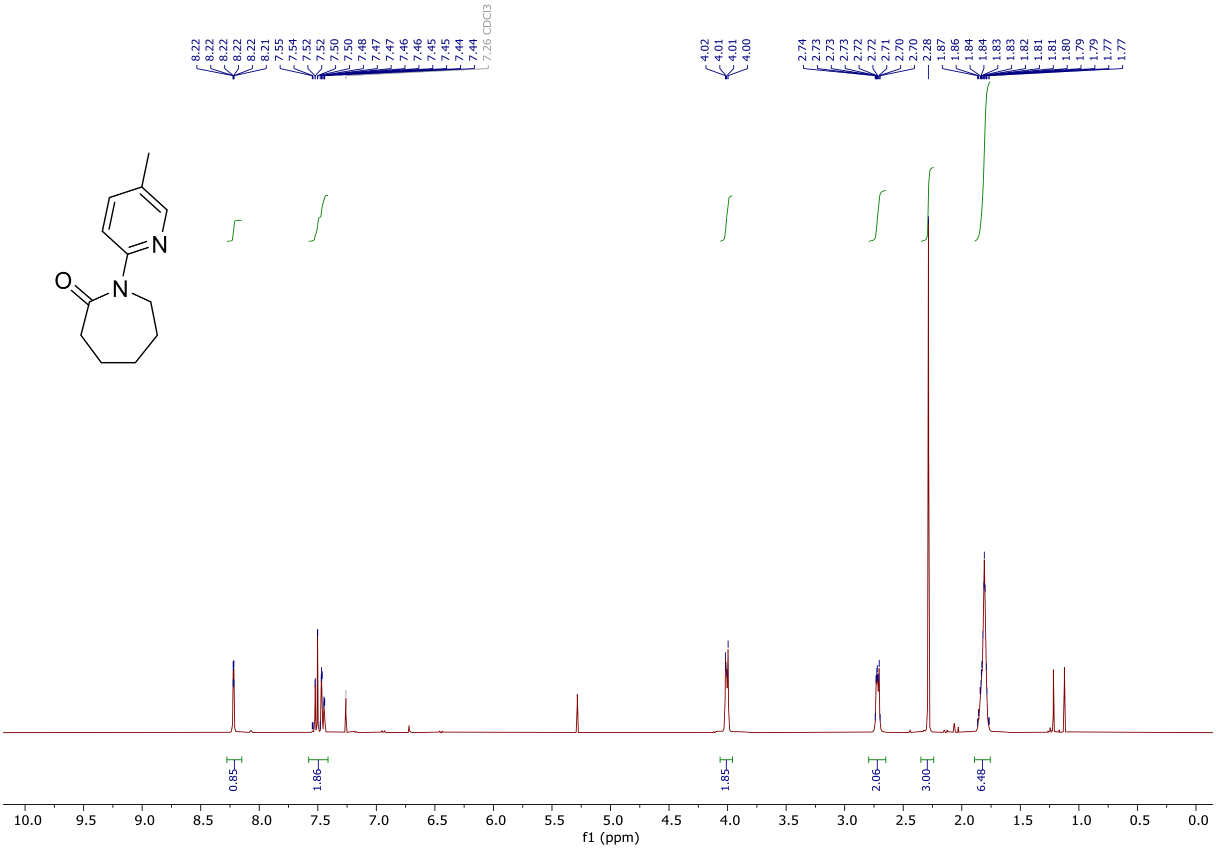

1ac – ^{13}C NMR (101 MHz, $CDCl_3$)

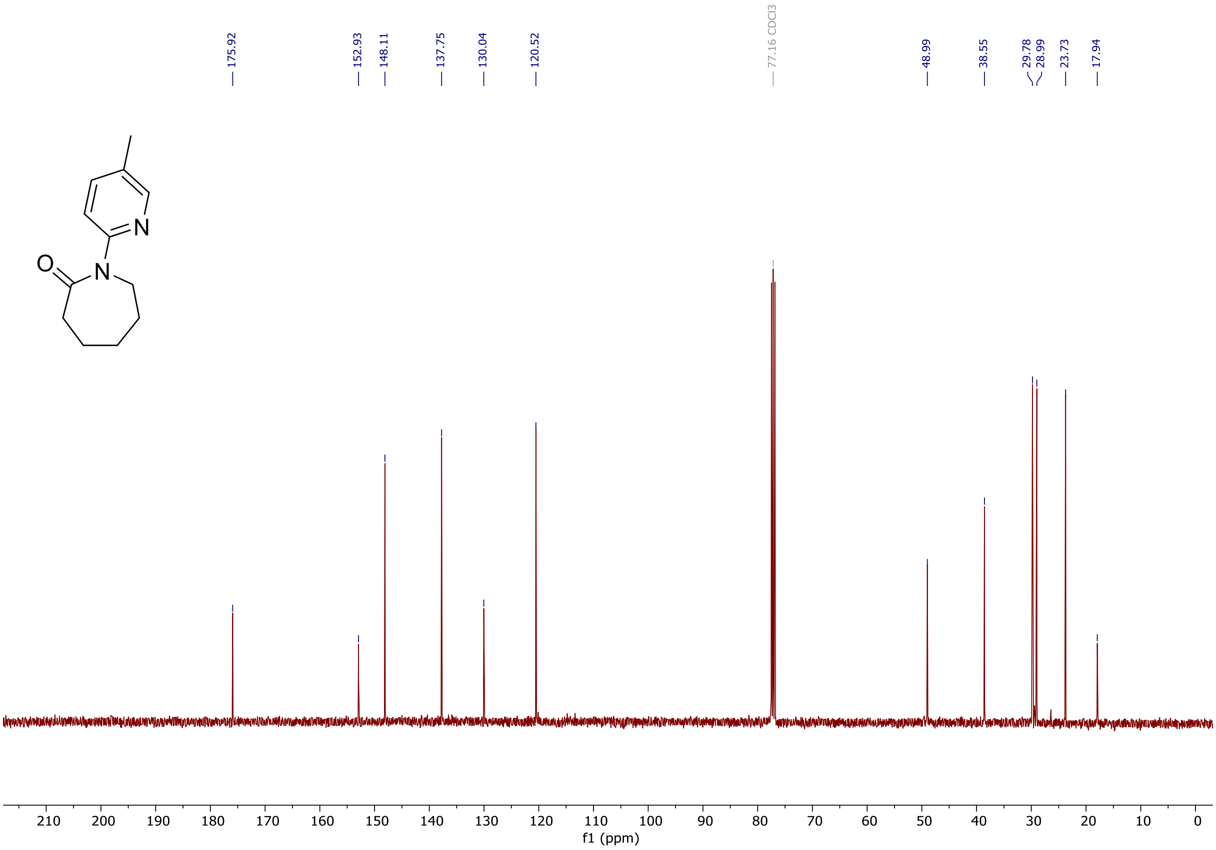

1ad – ^{1}H NMR (400 MHz, CDCl$_3$)

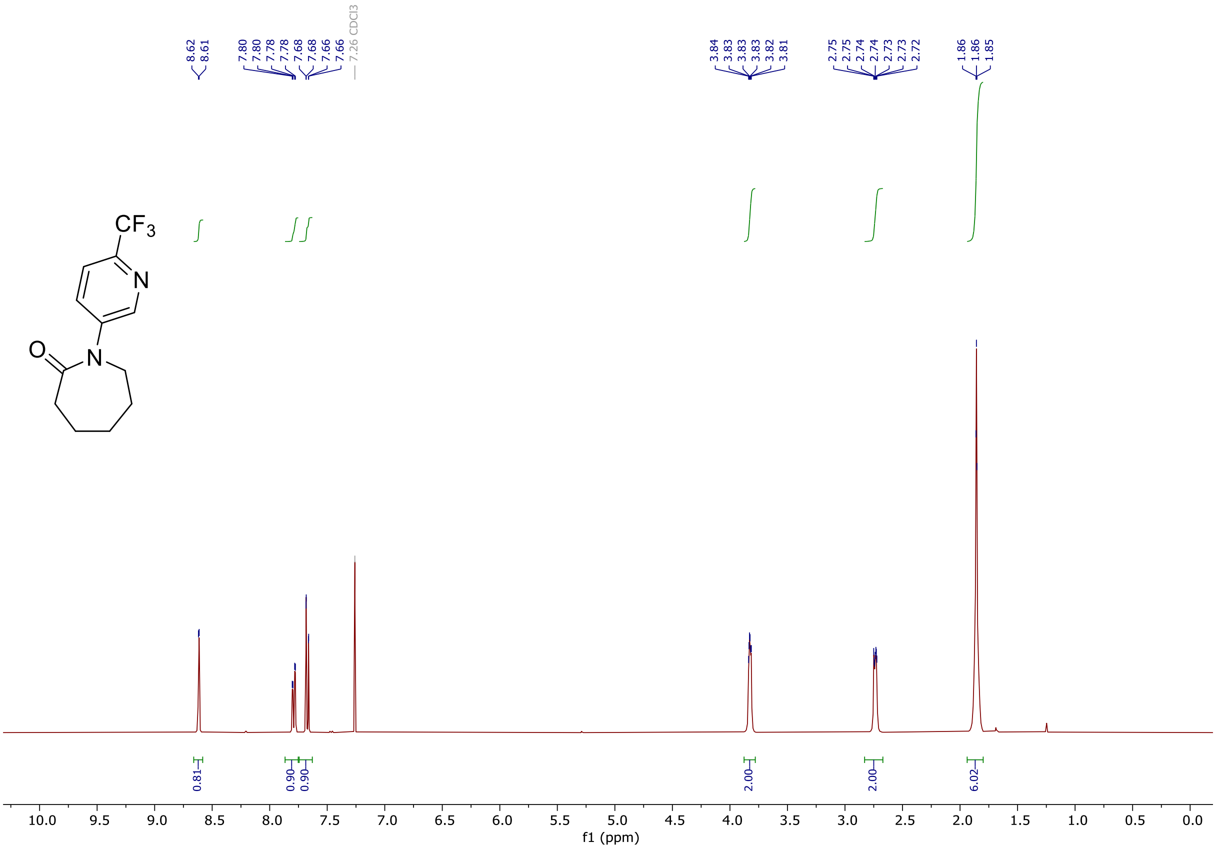

1ad – ^{13}C NMR (101 MHz, CDCl$_3$)

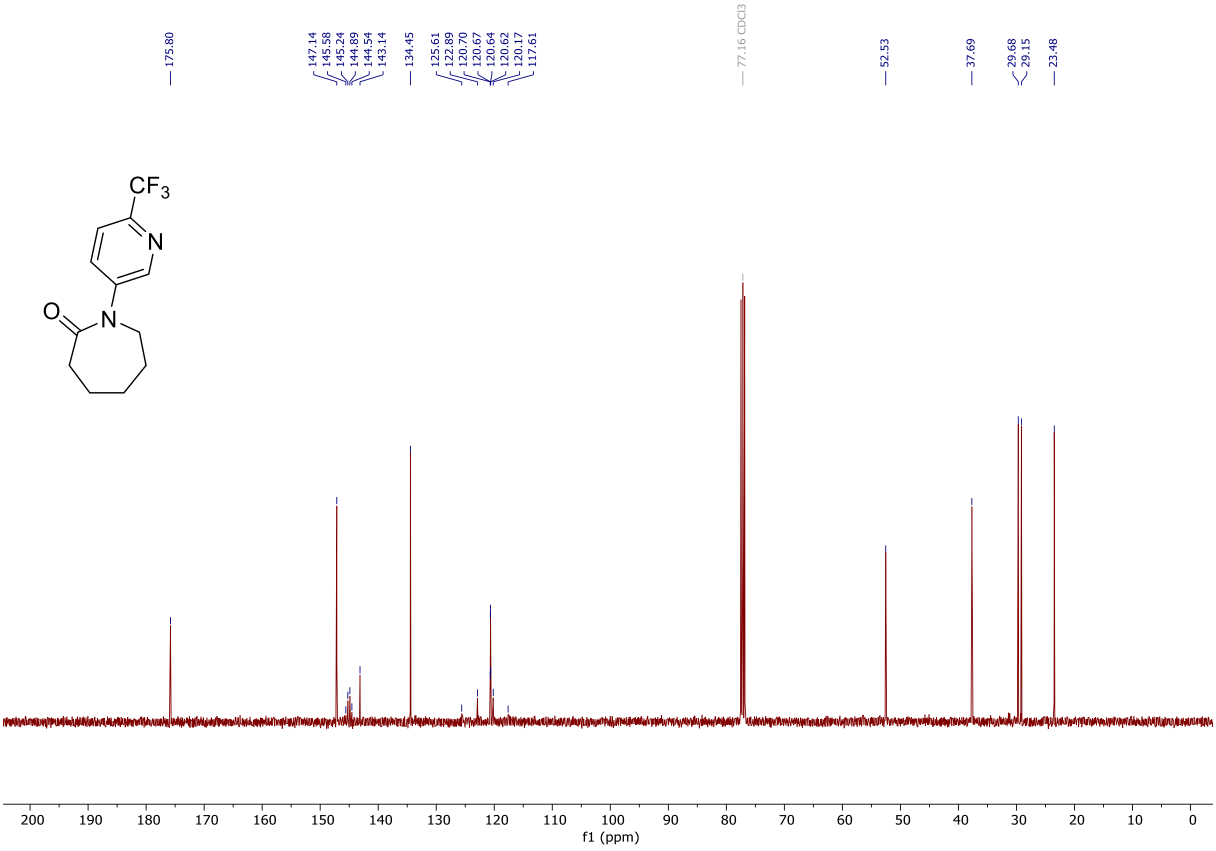

1ad – ^{19}F NMR (377 MHz, $CDCl_3$)

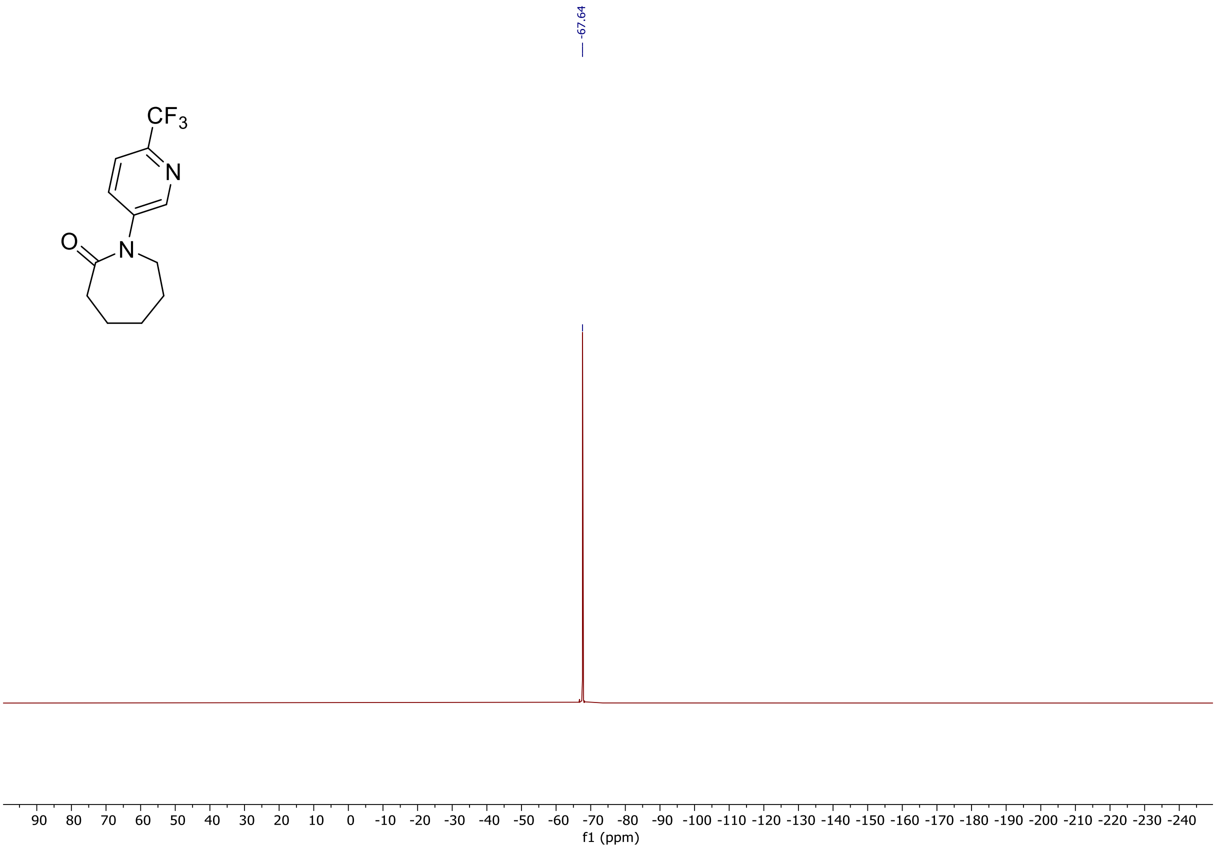

1ae – ^{1}H NMR (400 MHz, $CDCl_3$)

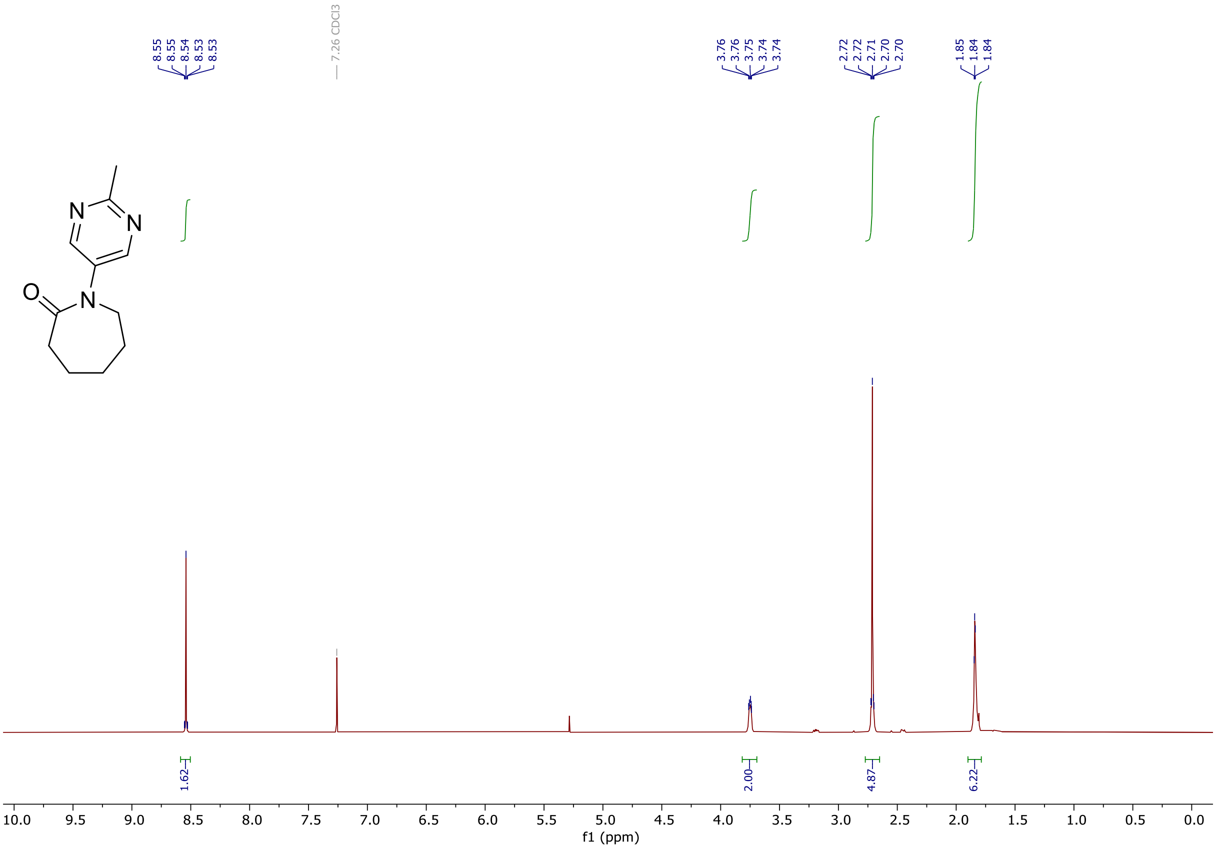

1ae – ^{13}C NMR (101 MHz, $CDCl_3$)

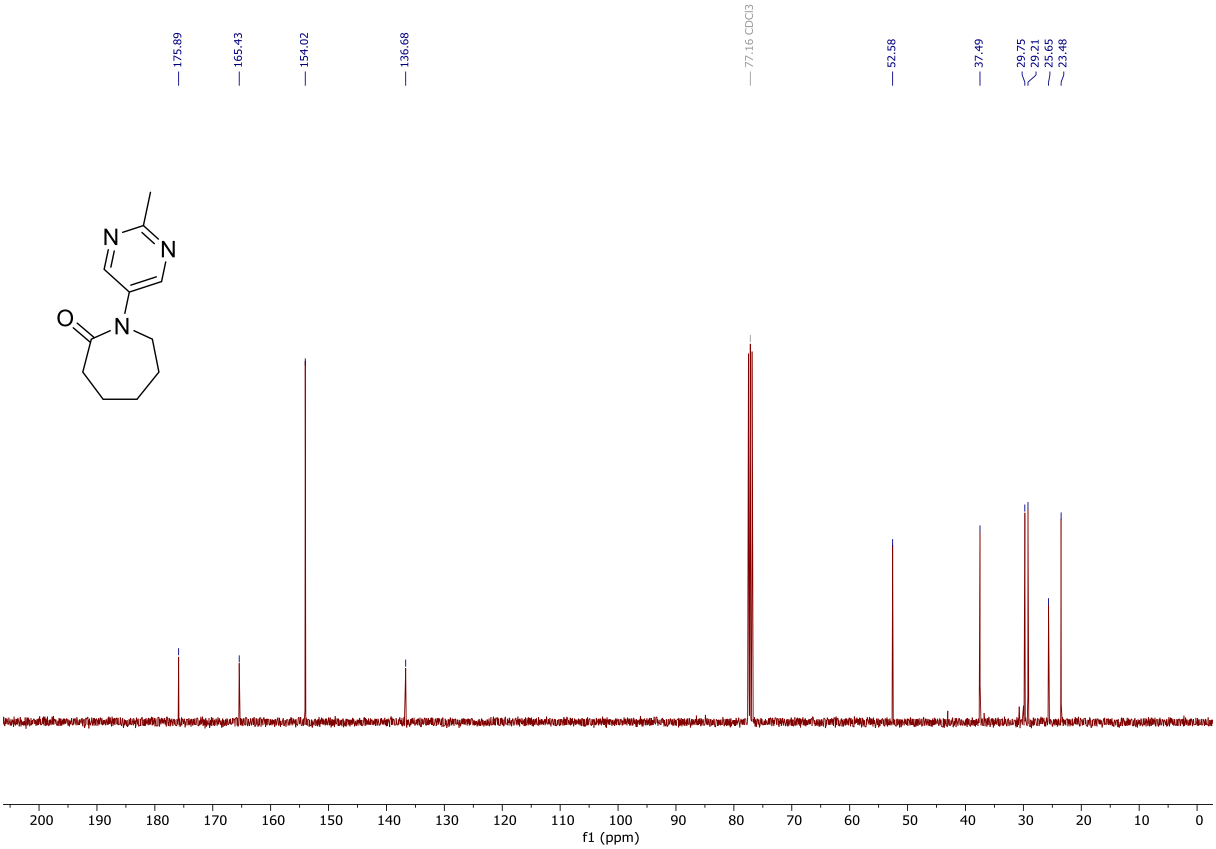

1ah – ^{1}H NMR (400 MHz, $CDCl_3$)

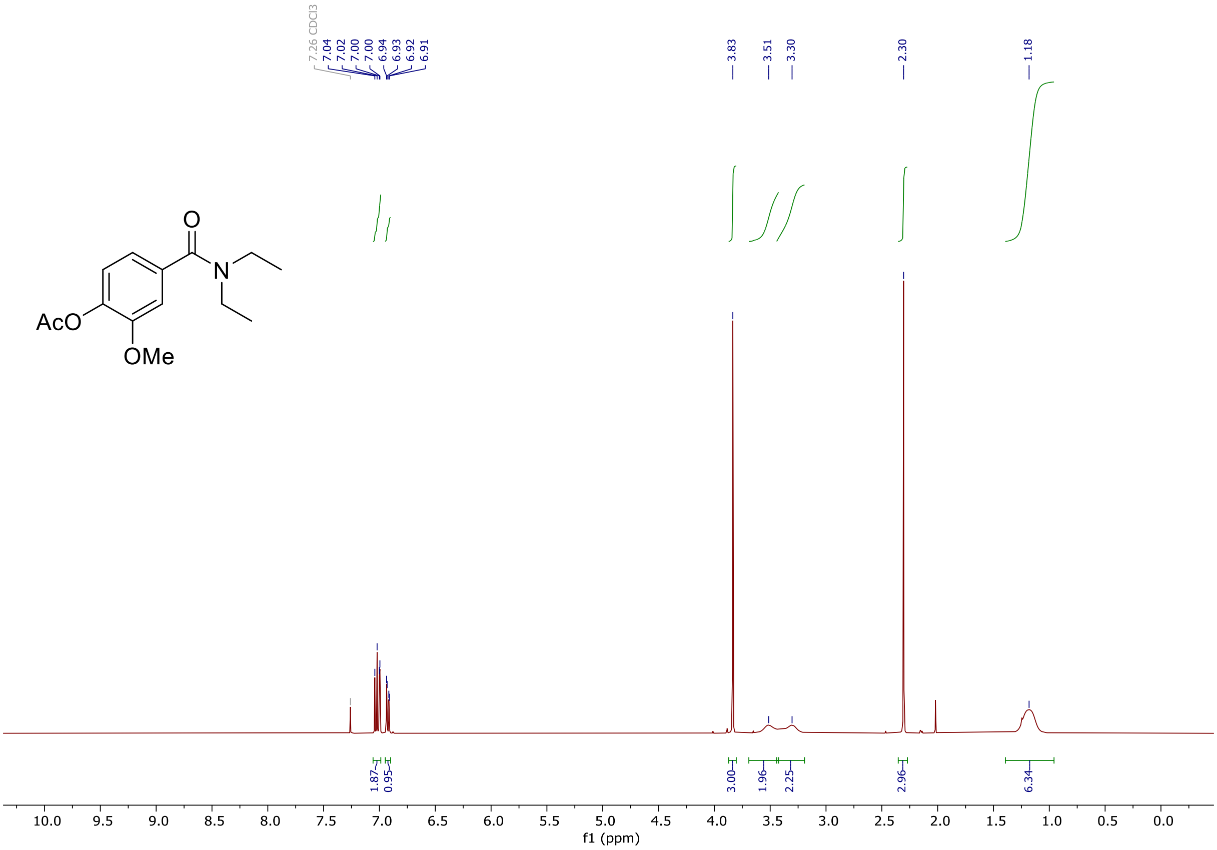

1ah – ^{13}C NMR (101 MHz, CDCl$_3$)

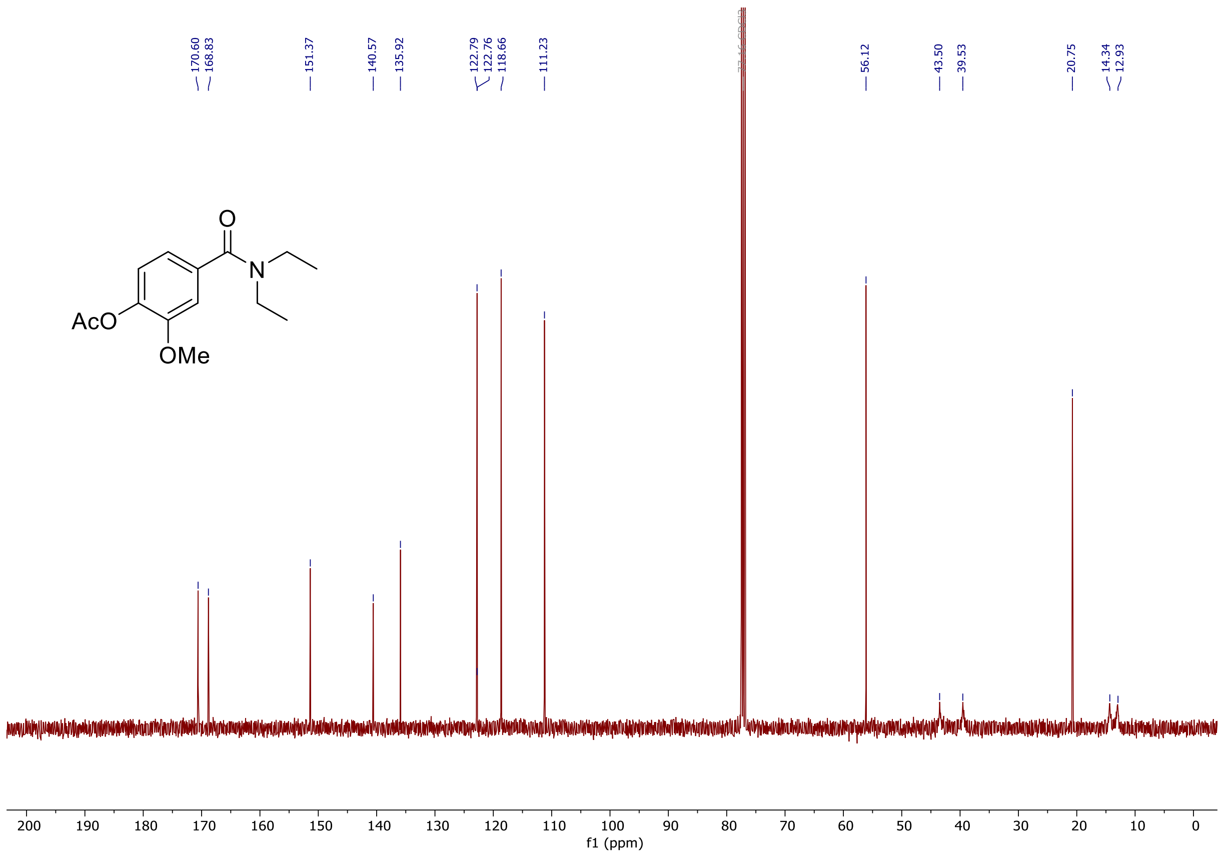

2f – ^{1}H NMR (400 MHz, CDCl$_3$)

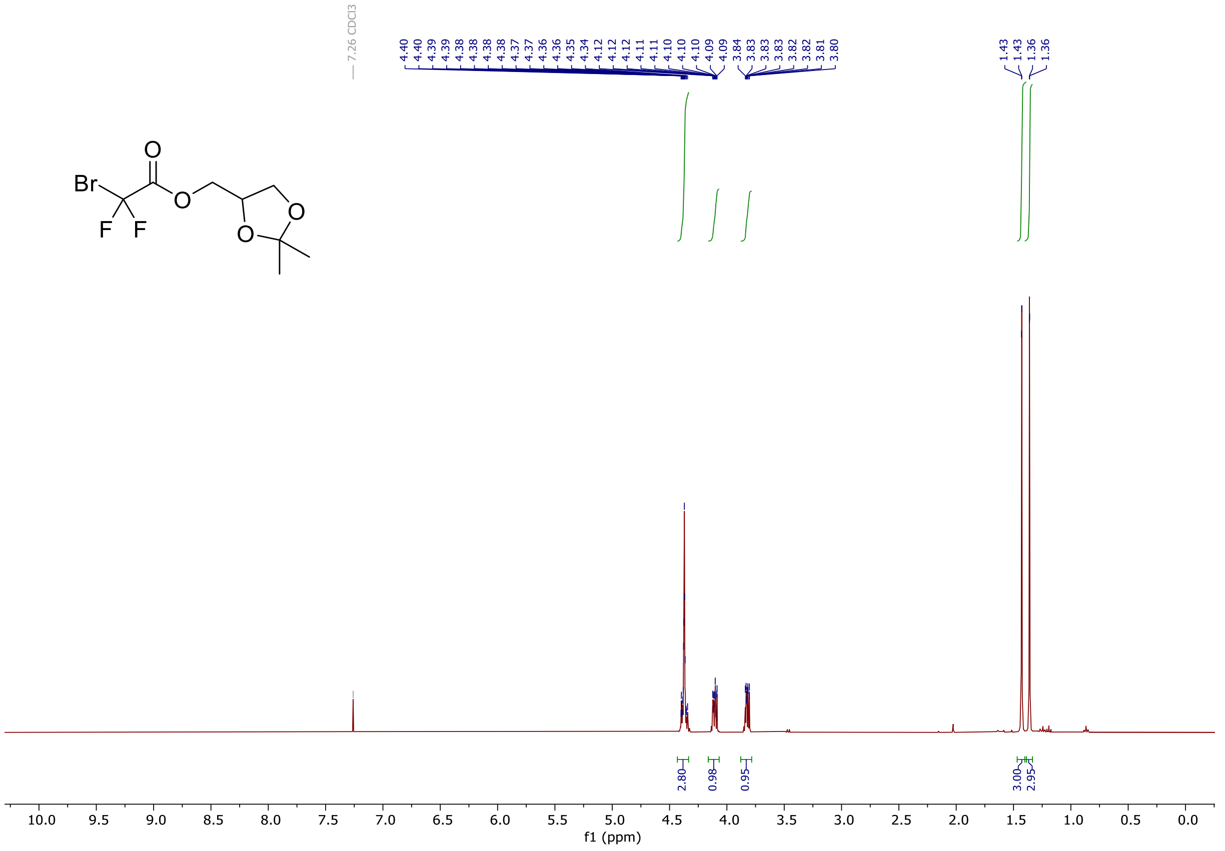

2f – ^{13}C NMR (101 MHz, $CDCl_3$)

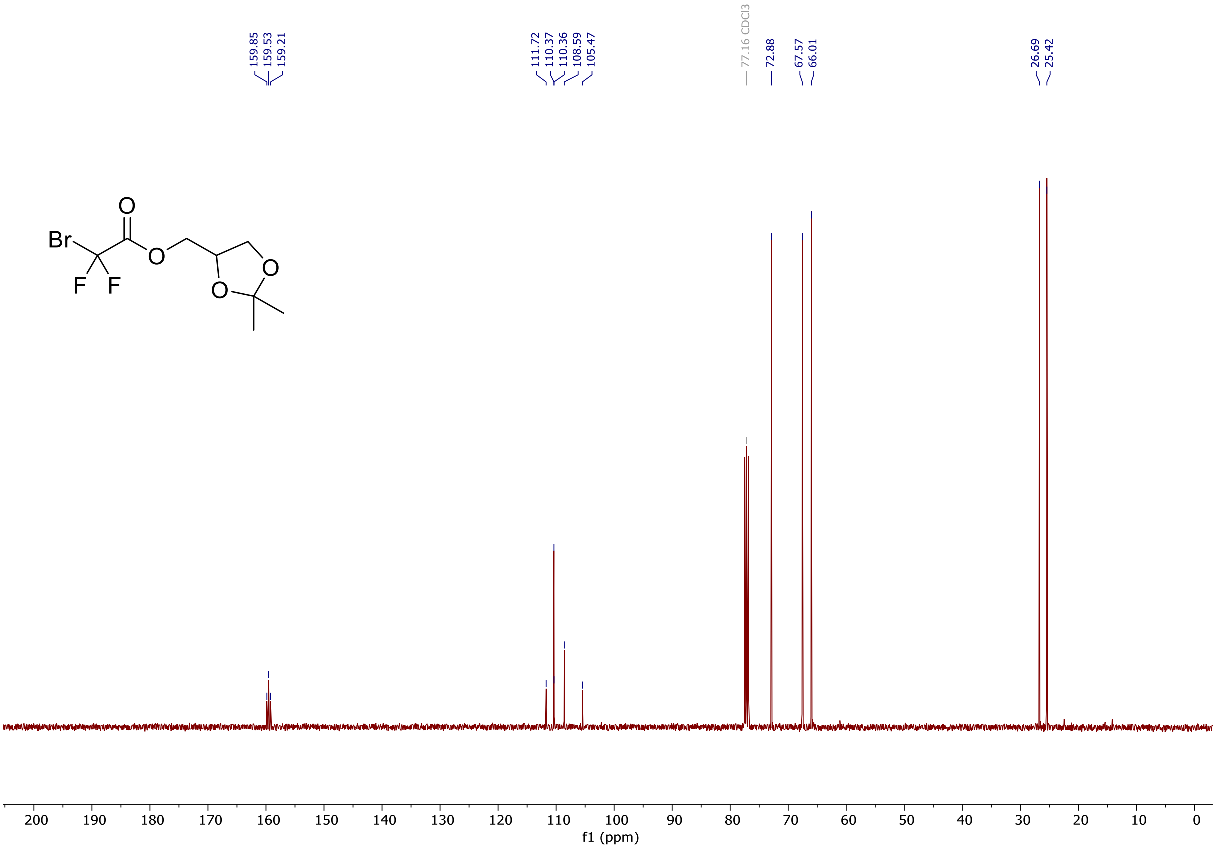

2f – ^{19}F NMR (377 MHz, $CDCl_3$)

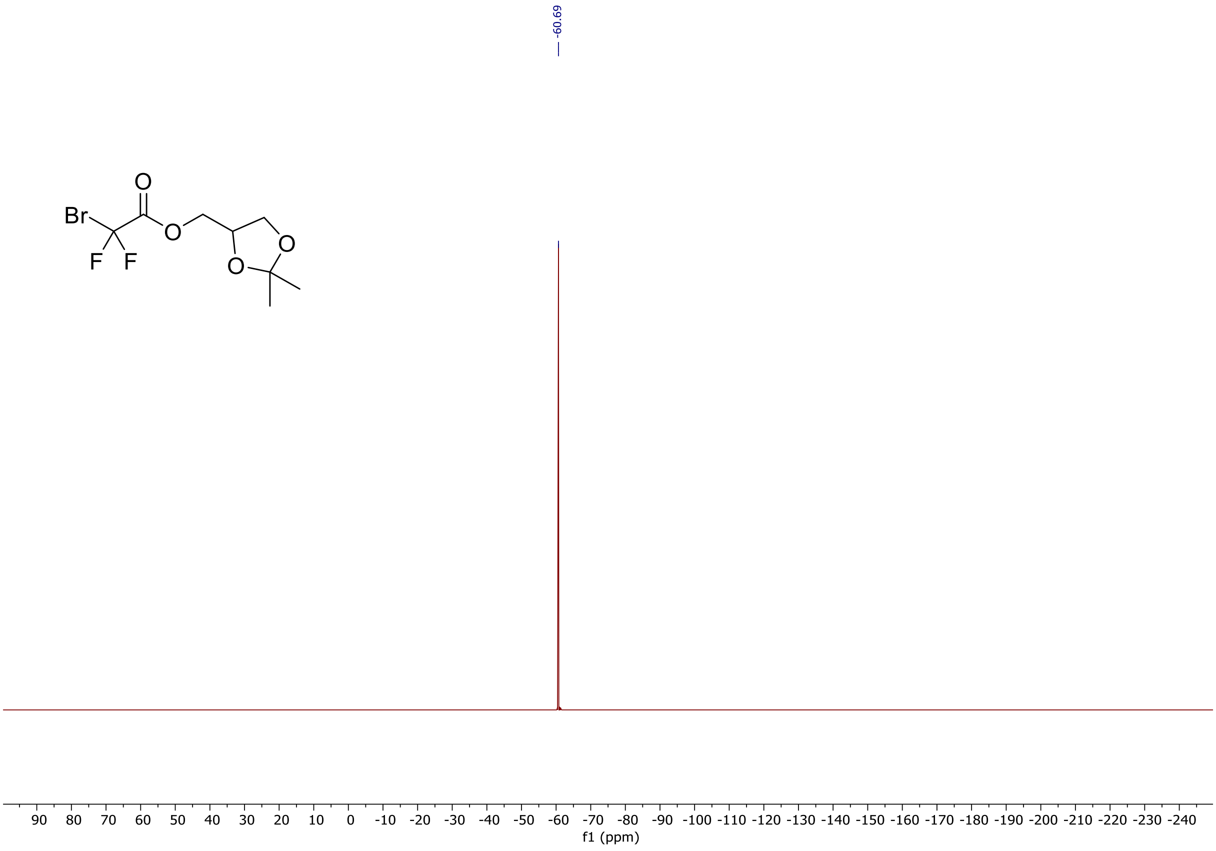

2g – ^{1}H NMR (400 MHz, $CDCl_3$)

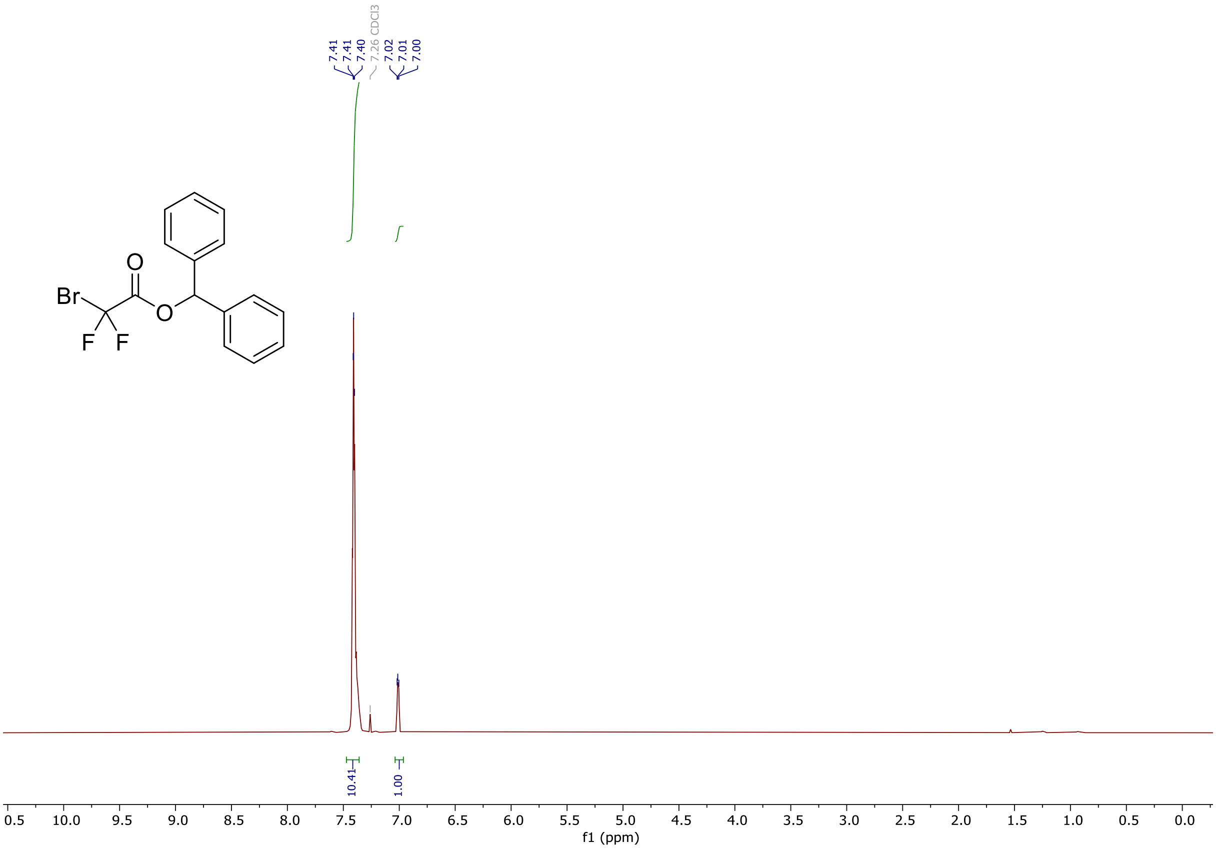

2g – ^{13}C NMR (101 MHz, $CDCl_3$)

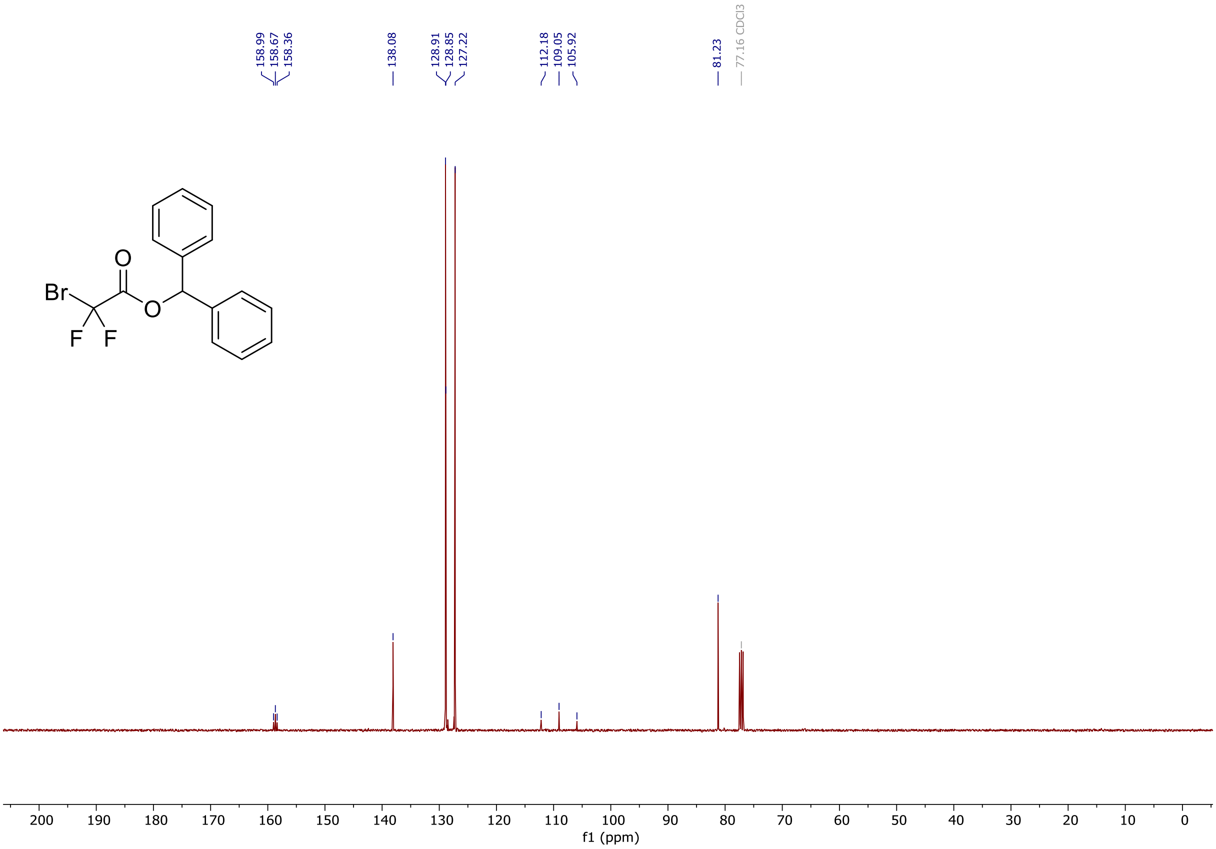

2g – ^{19}F NMR (377 MHz, $CDCl_3$)

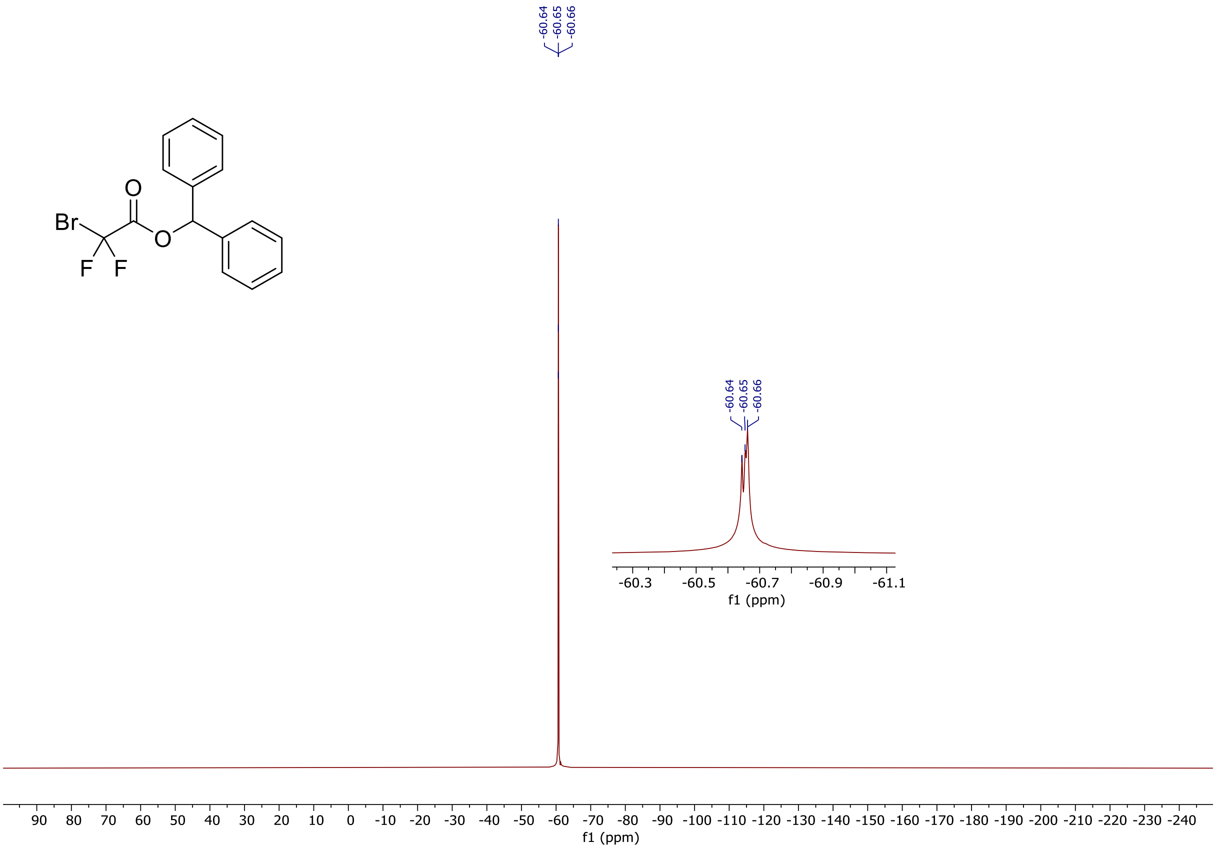

2j – ^{1}H NMR (400 MHz, $CDCl_3$)

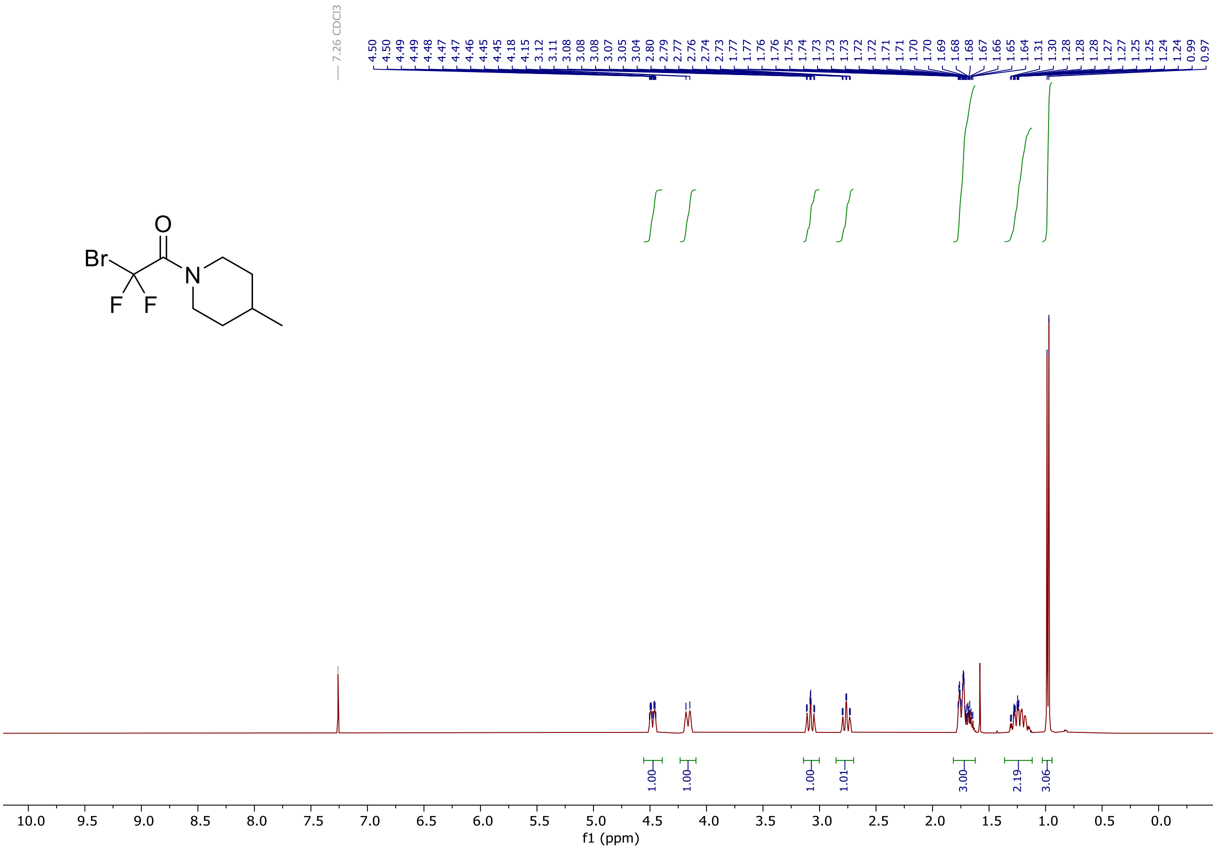

2j – ^{13}C NMR (101 MHz, CDCl$_3$)

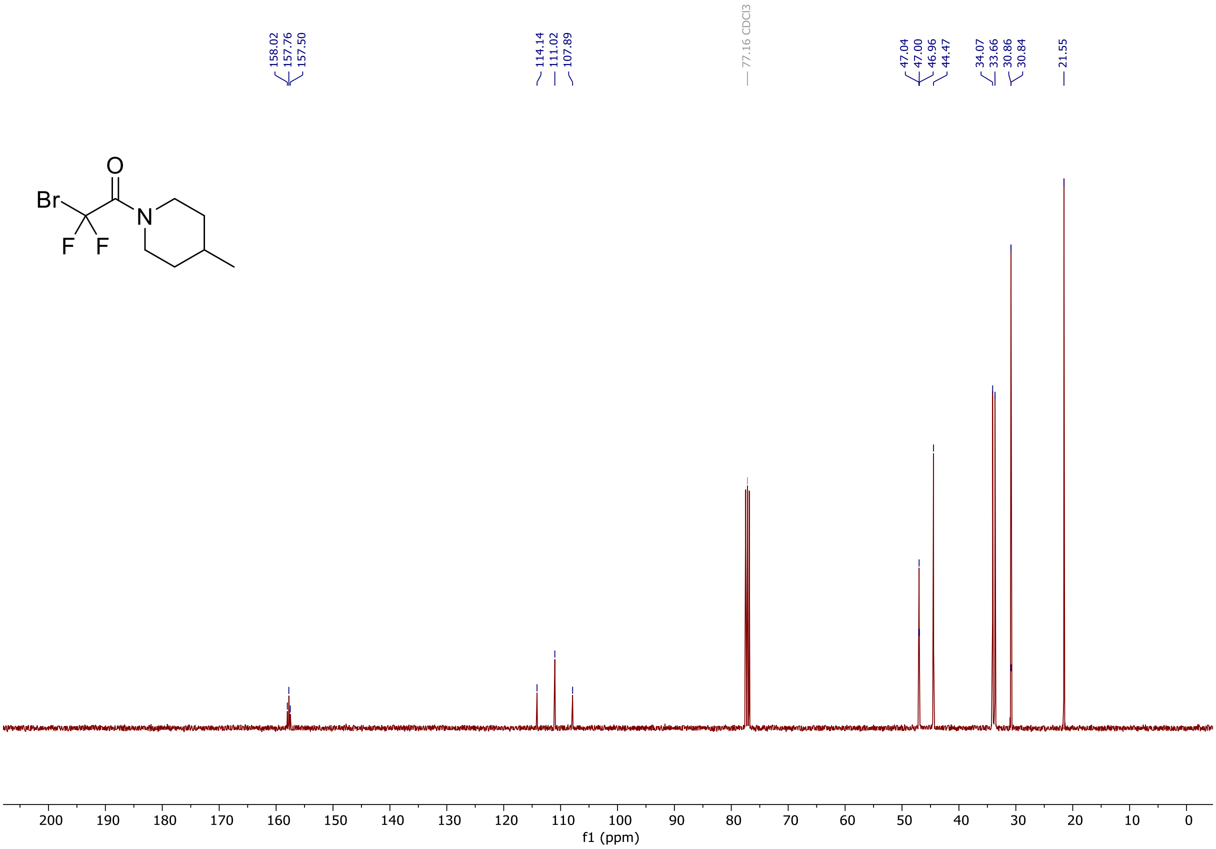

2j – ^{19}F NMR (377 MHz, CDCl$_3$)

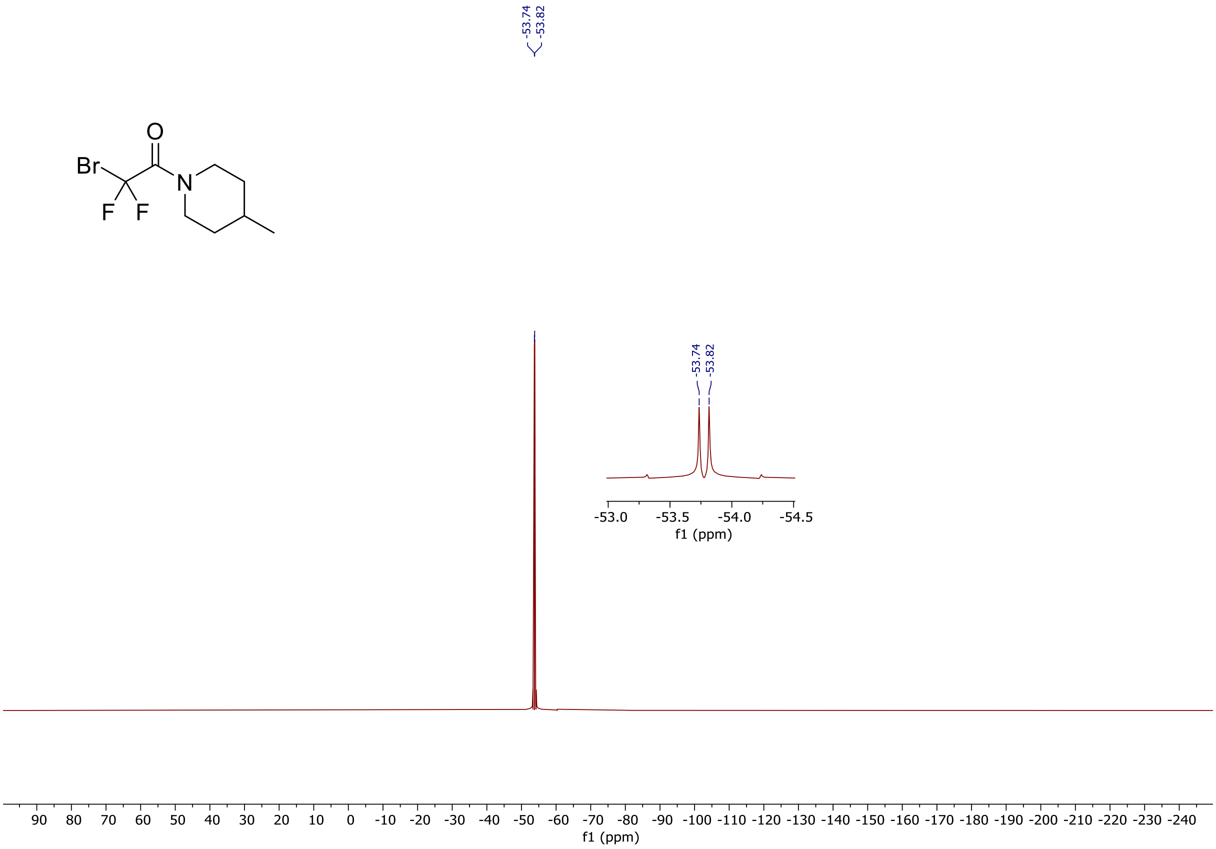

3a – ^{1}H NMR (400 MHz, $CDCl_3$)

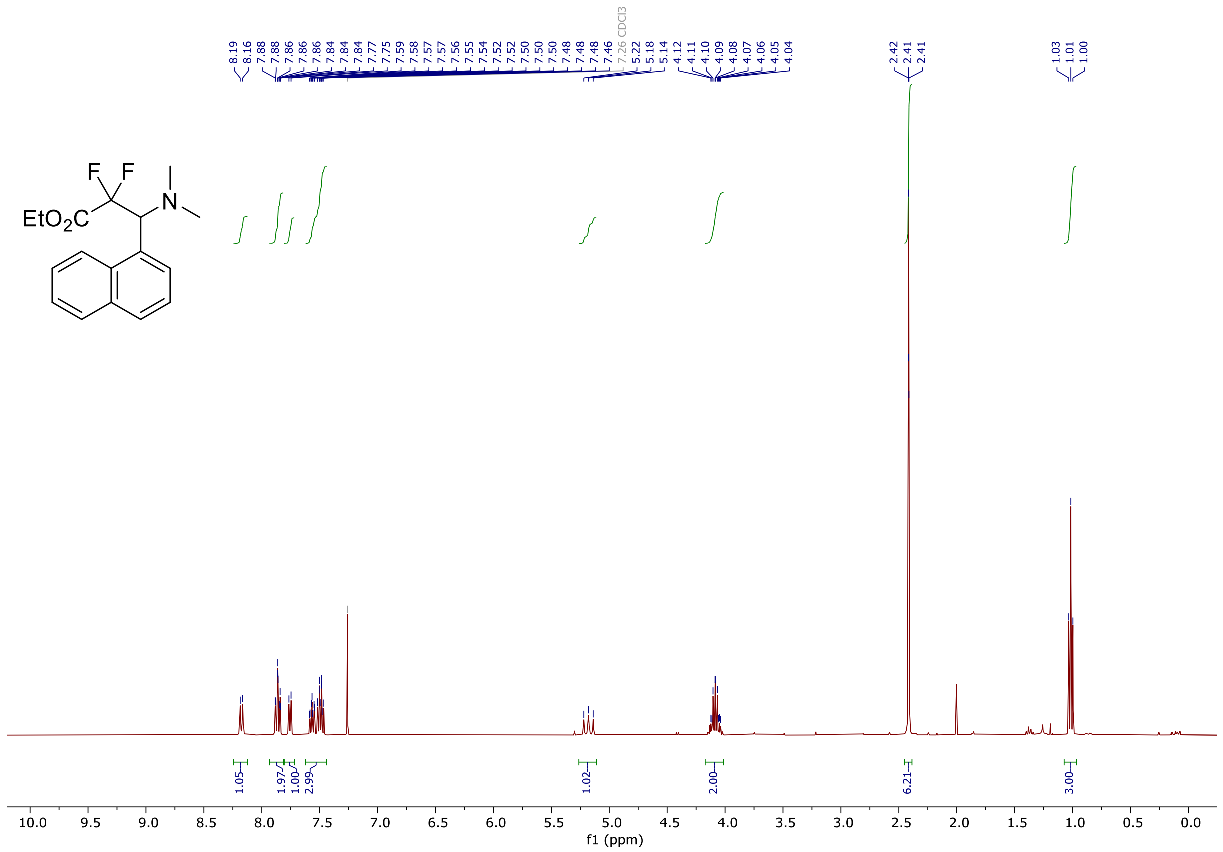

3a – ^{13}C NMR (126 MHz, $CDCl_3$)

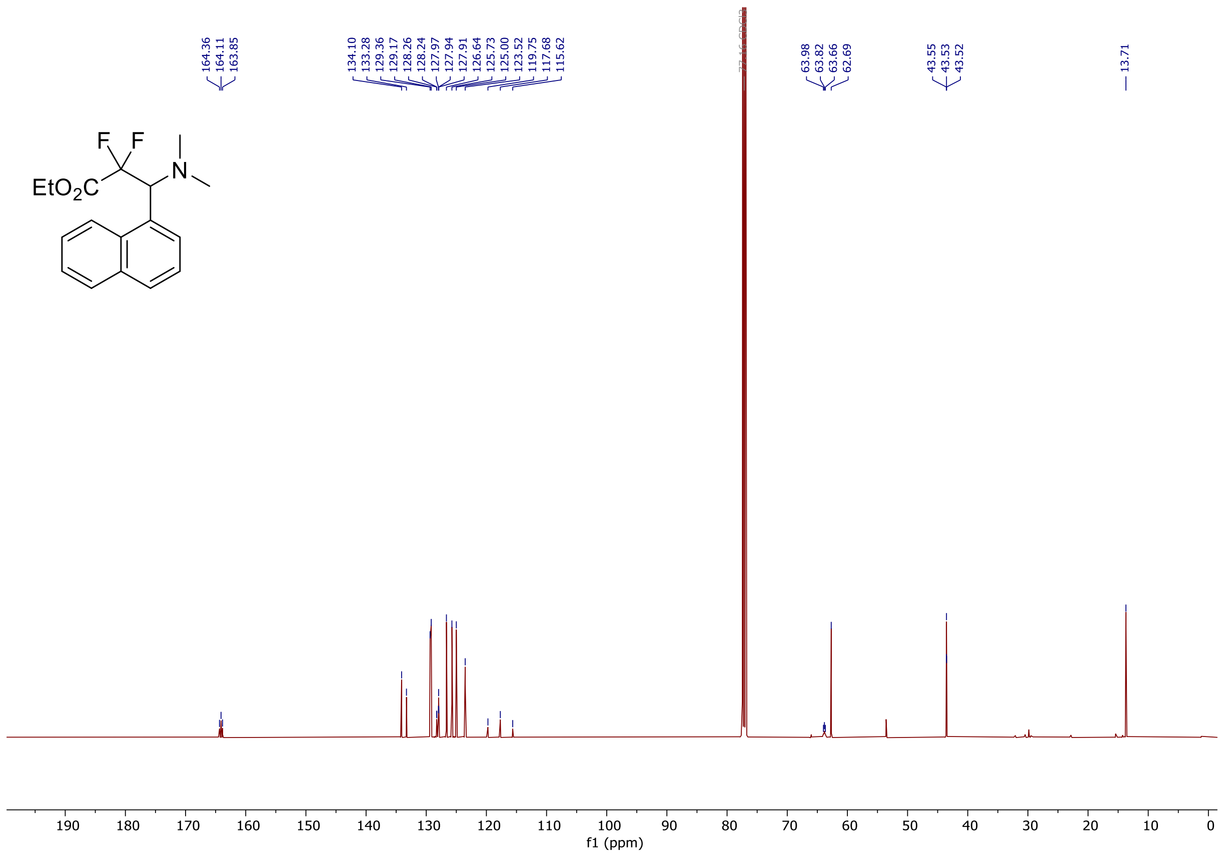

3a – ^{19}F NMR (377 MHz, $CDCl_3$)

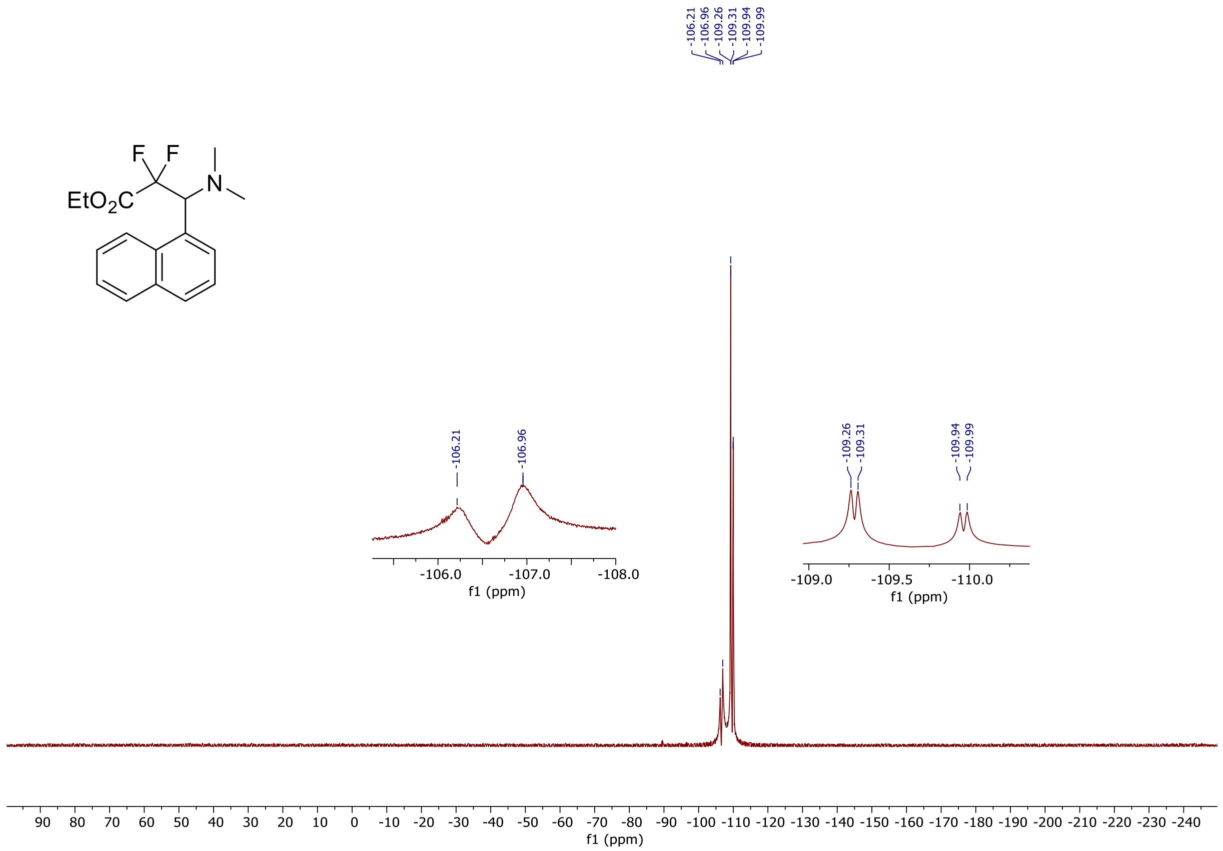

3b – ^{1}H NMR (400 MHz, $CDCl_3$)

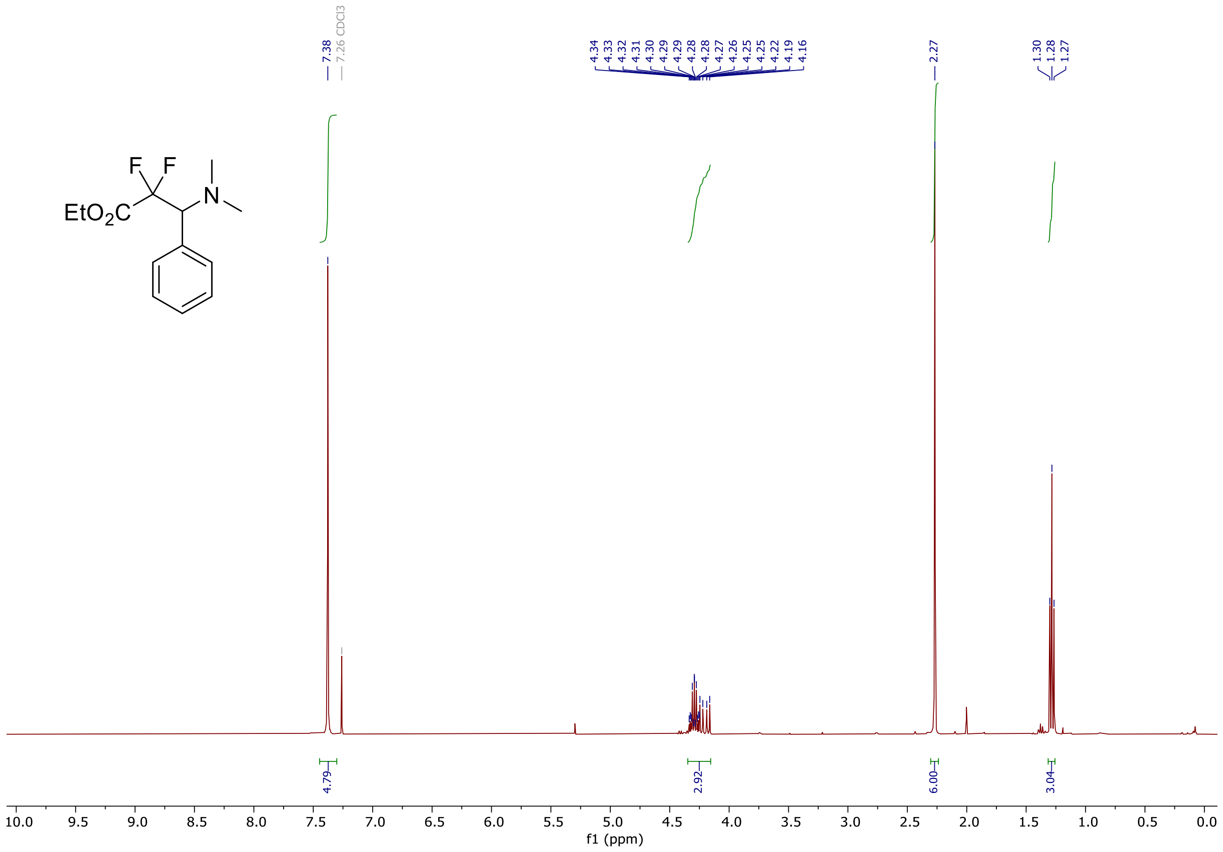

3b – ^{13}C NMR (126 MHz, CDCl$_3$)

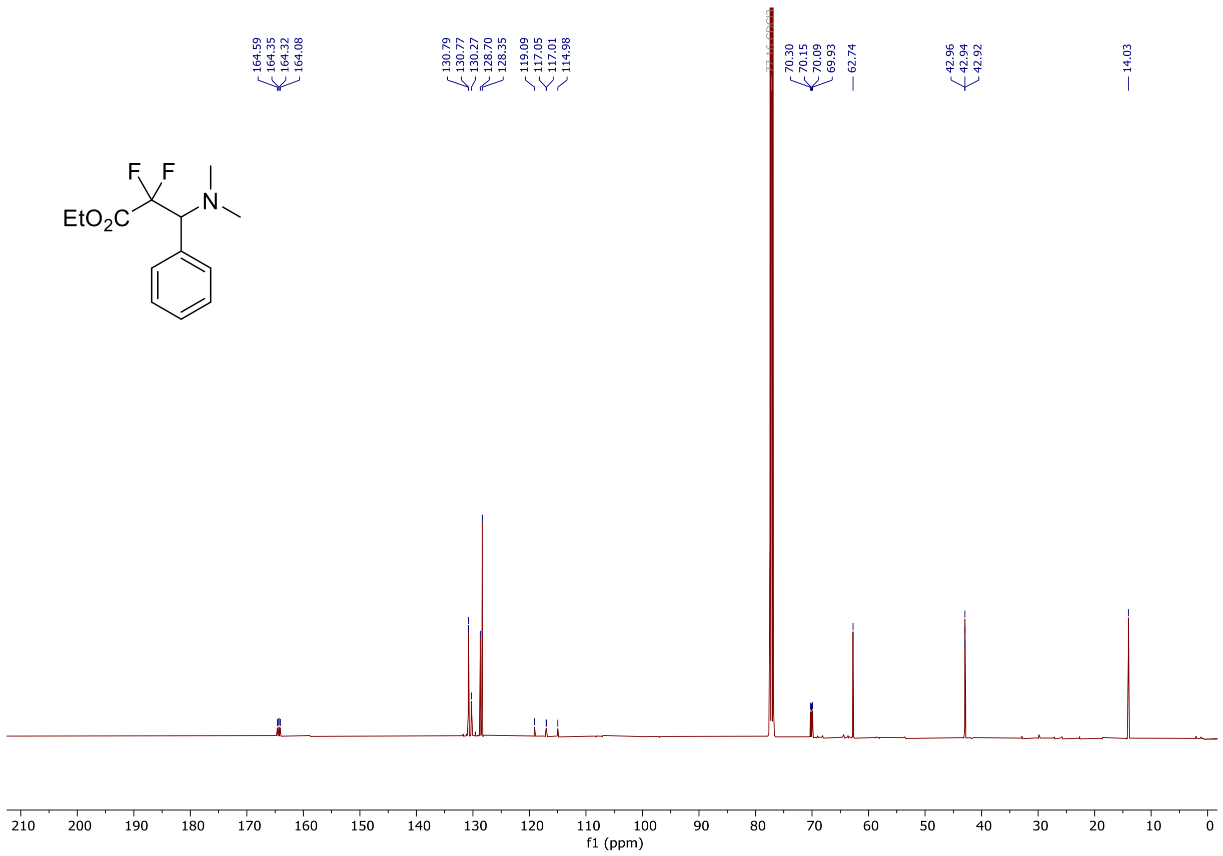

3b – ^{19}F NMR (377 MHz, CDCl$_3$)

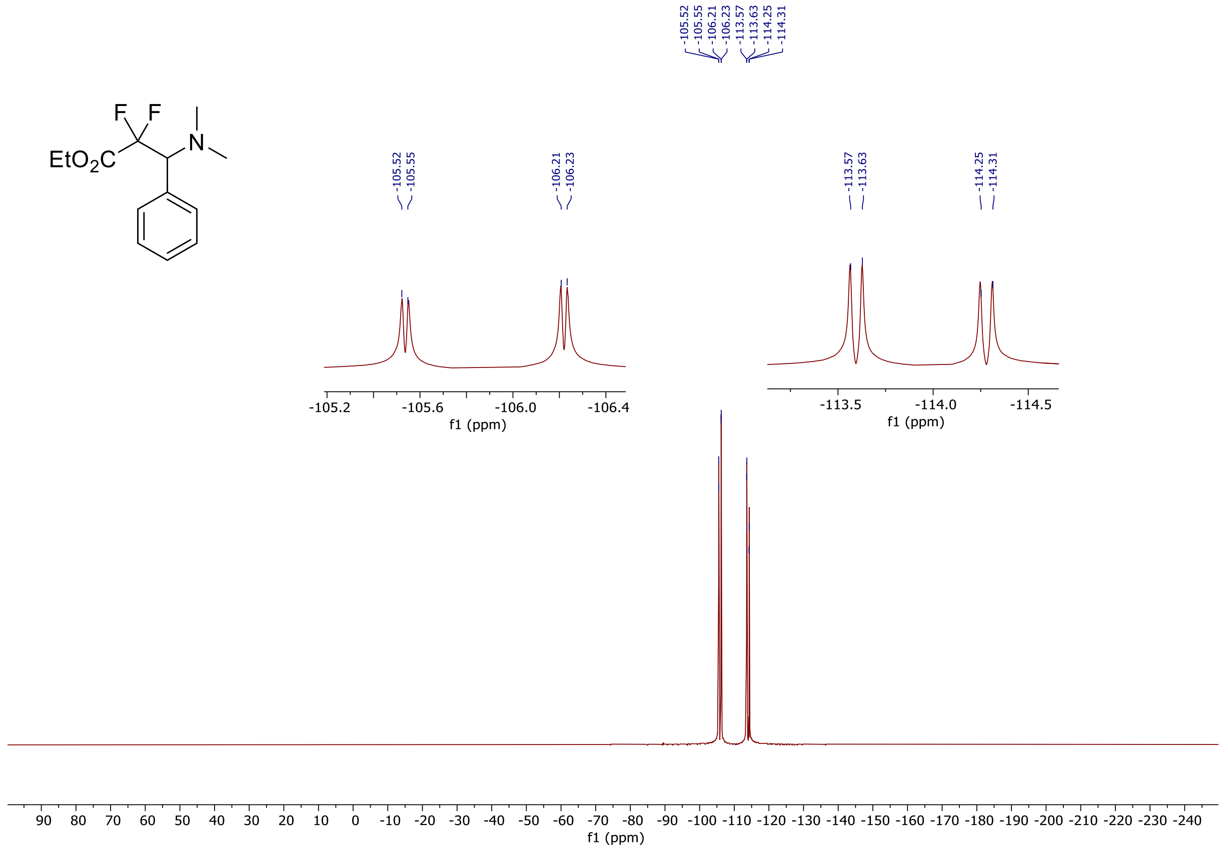

3c – ^{1}H NMR (400 MHz, $CDCl_3$)

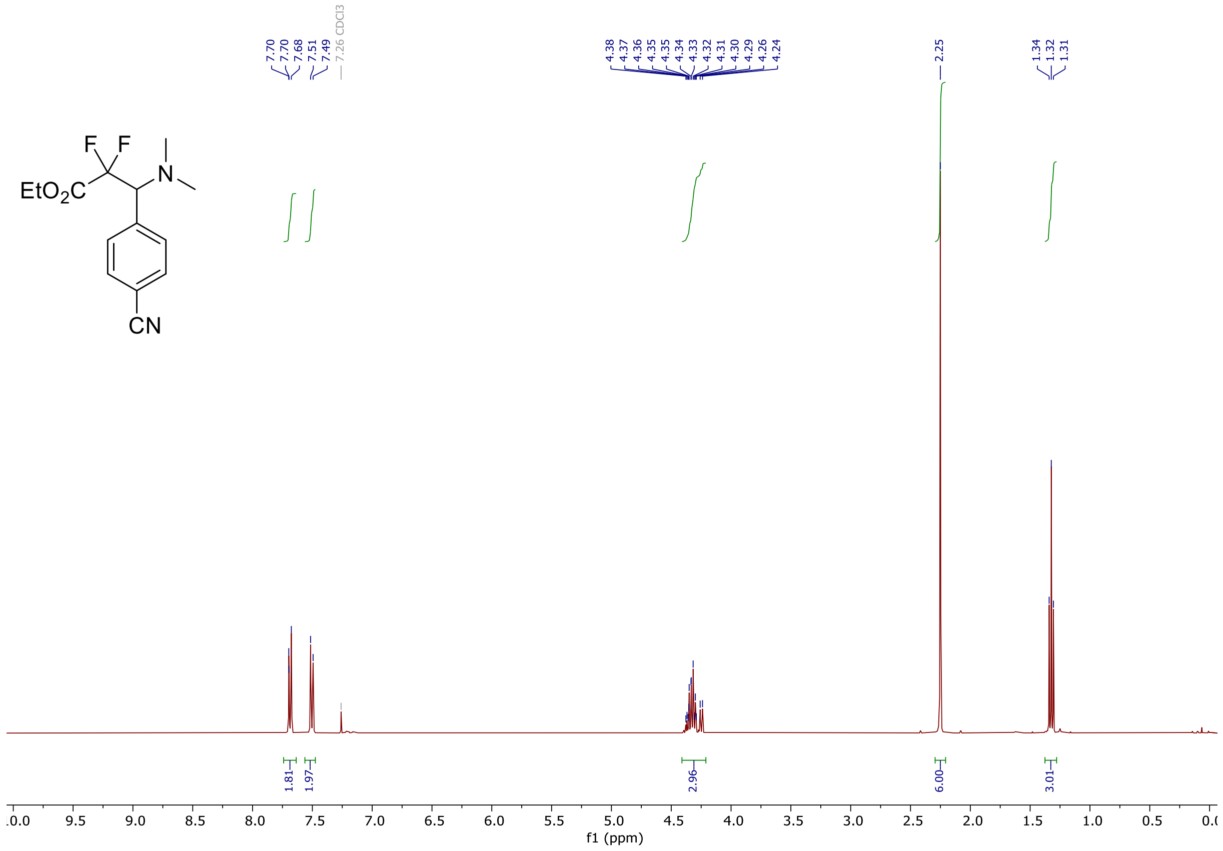

3c – ^{13}C NMR (101 MHz, $CDCl_3$)

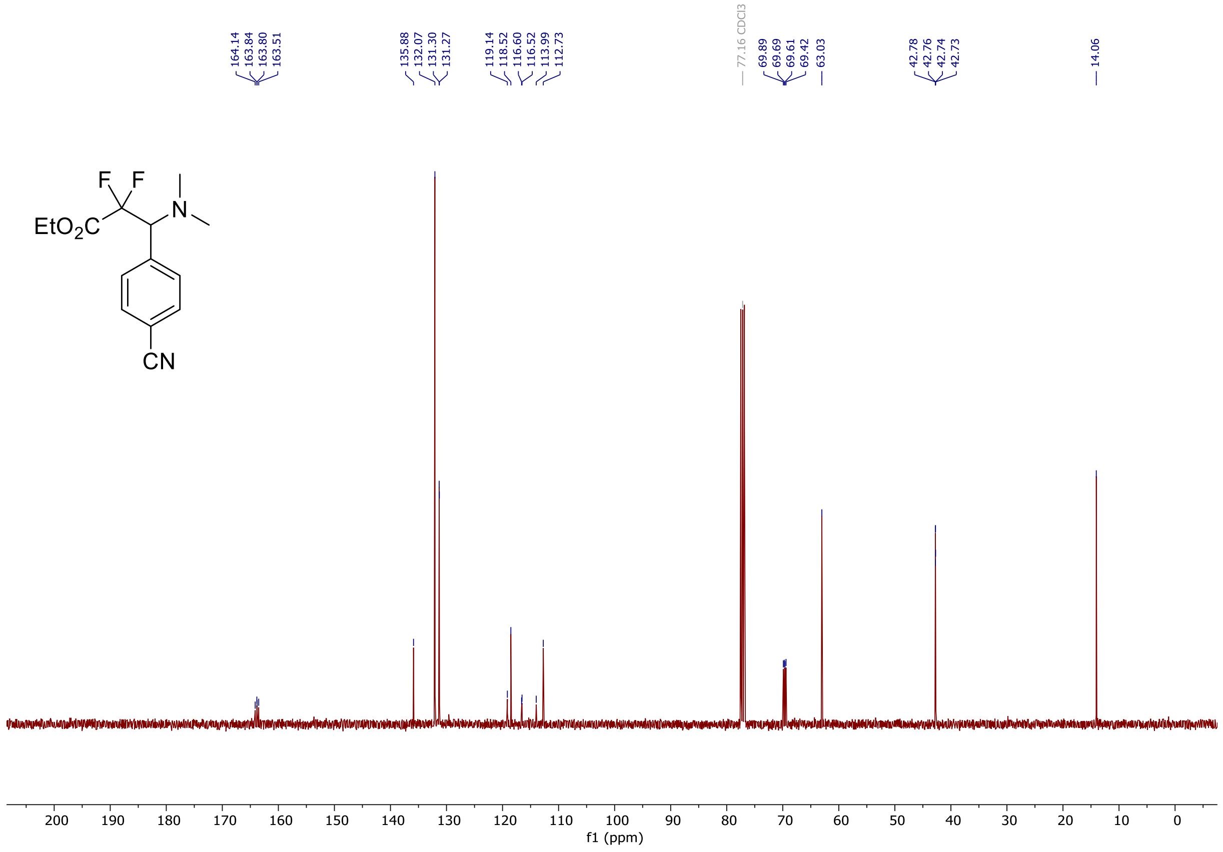

3c – ^{19}F NMR (377 MHz, $CDCl_3$)

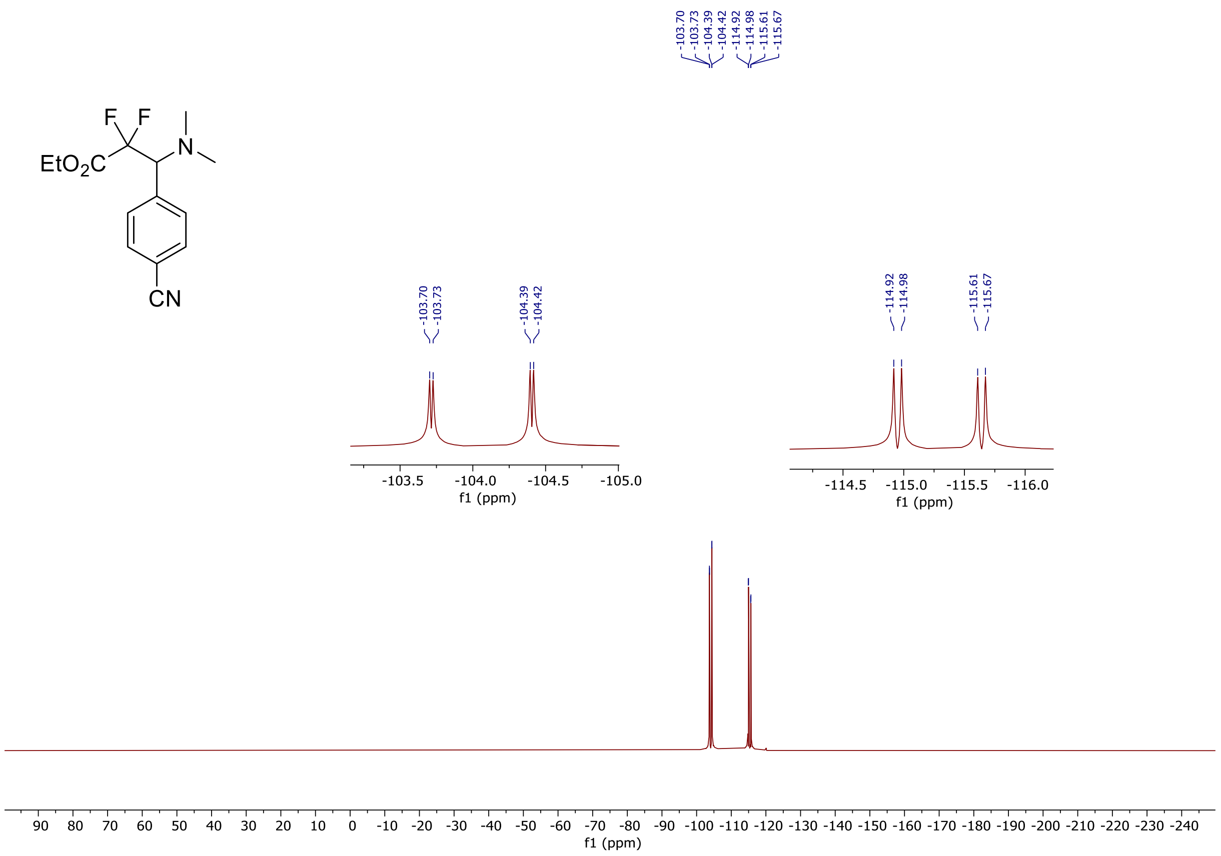

3d – ^{1}H NMR (400 MHz, $CDCl_3$)

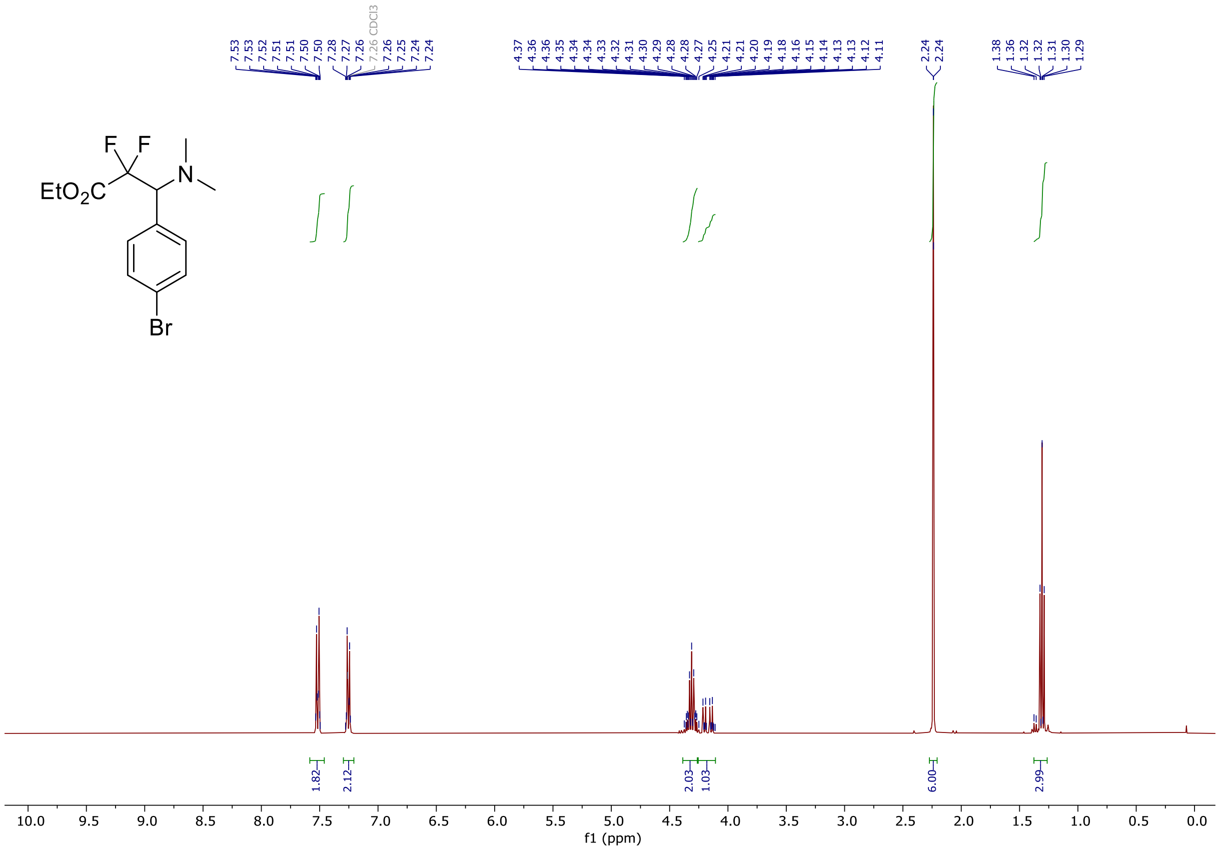

3d – ^{13}C NMR (101 MHz, $CDCl_3$)

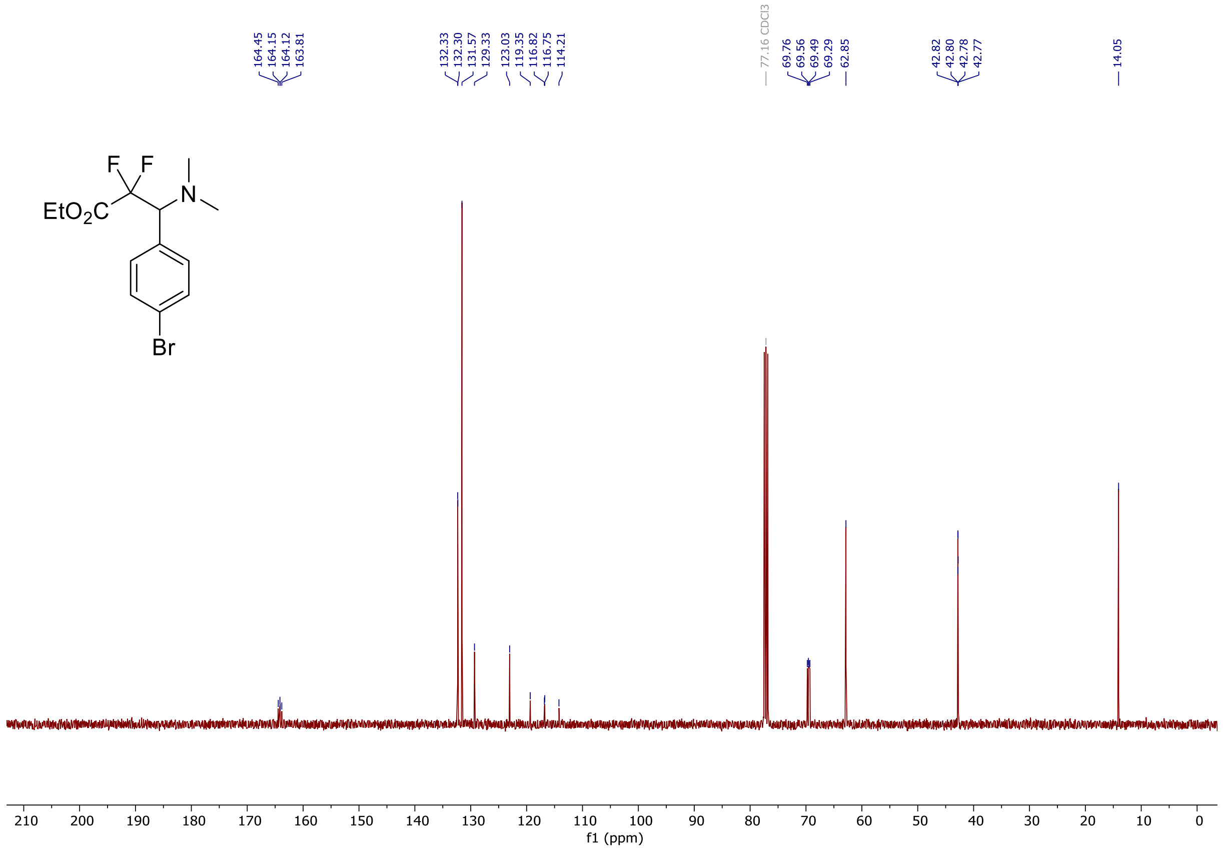

3d – ^{19}F NMR (377 MHz, $CDCl_3$)

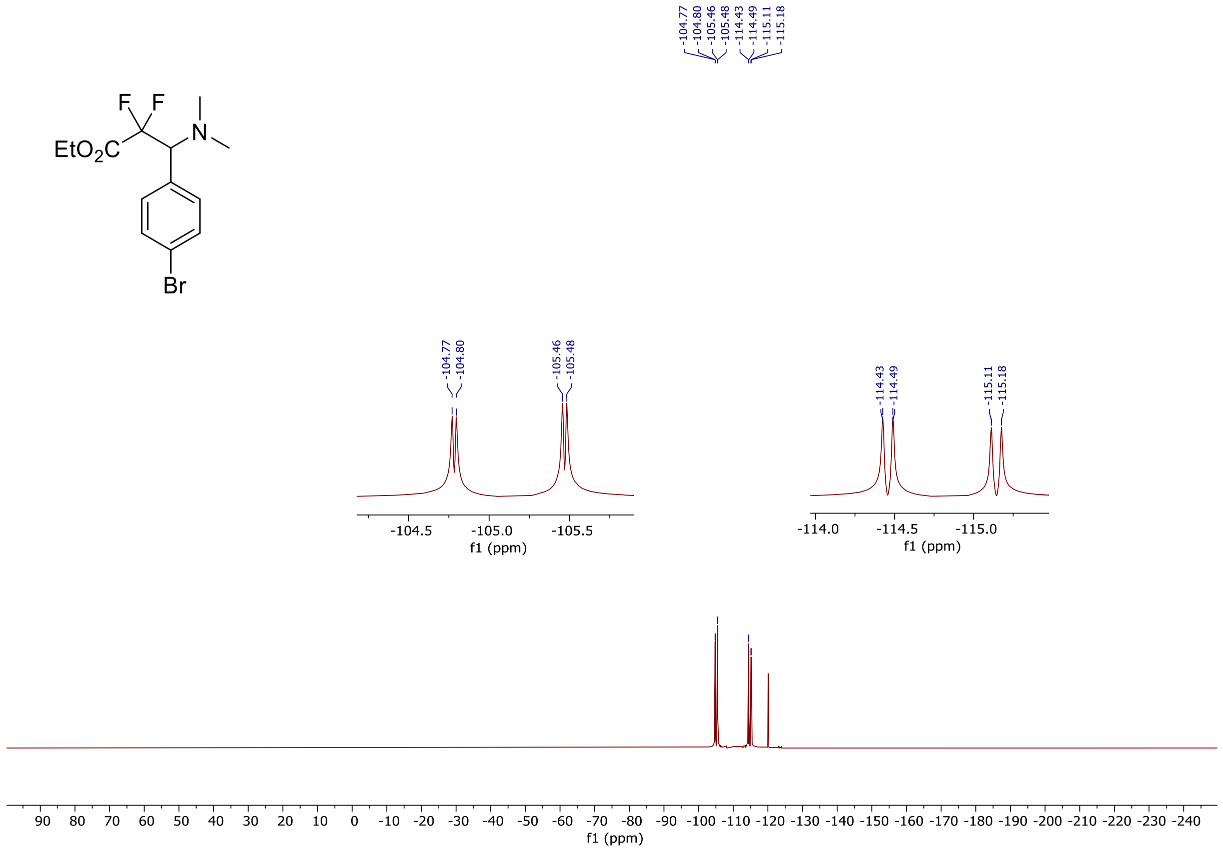

3e – ^{1}H NMR (400 MHz, $CDCl_3$)

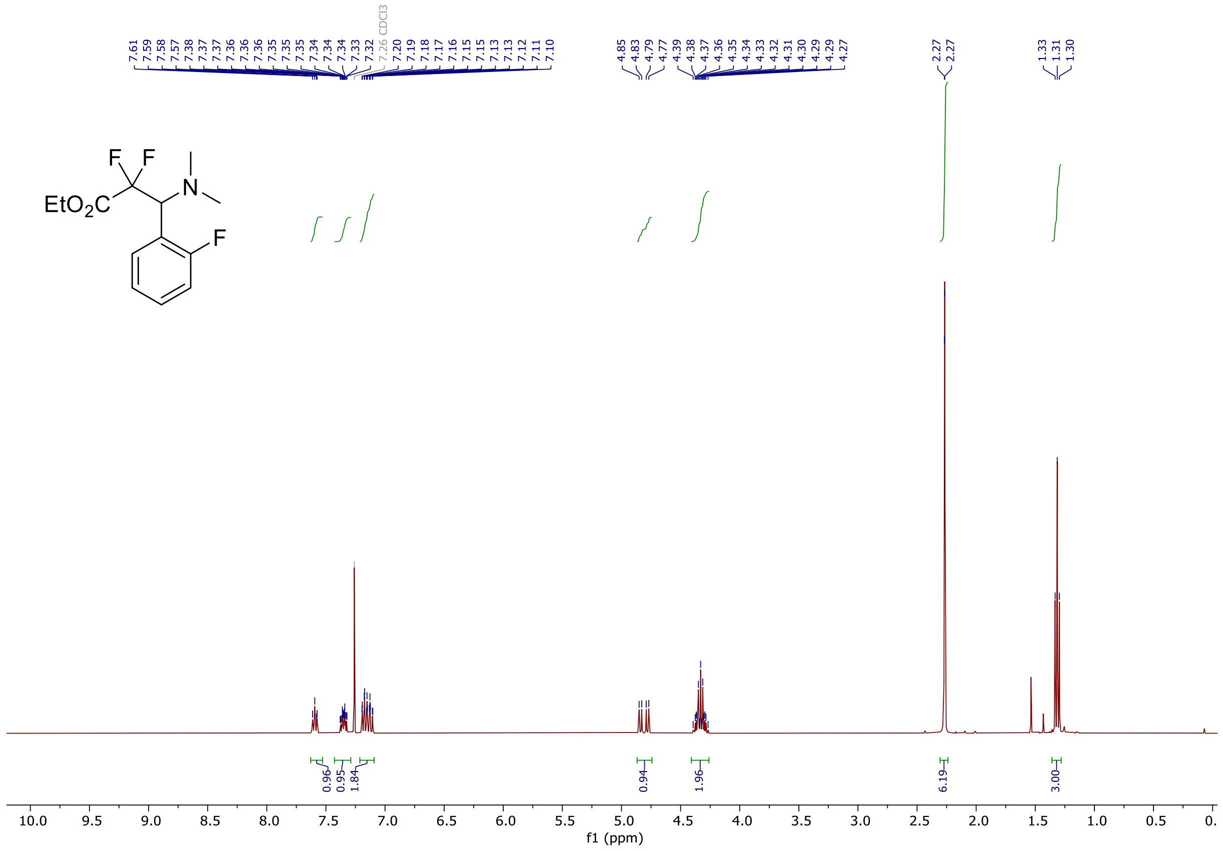

3e – ^{13}C NMR (126 MHz, $CDCl_3$)

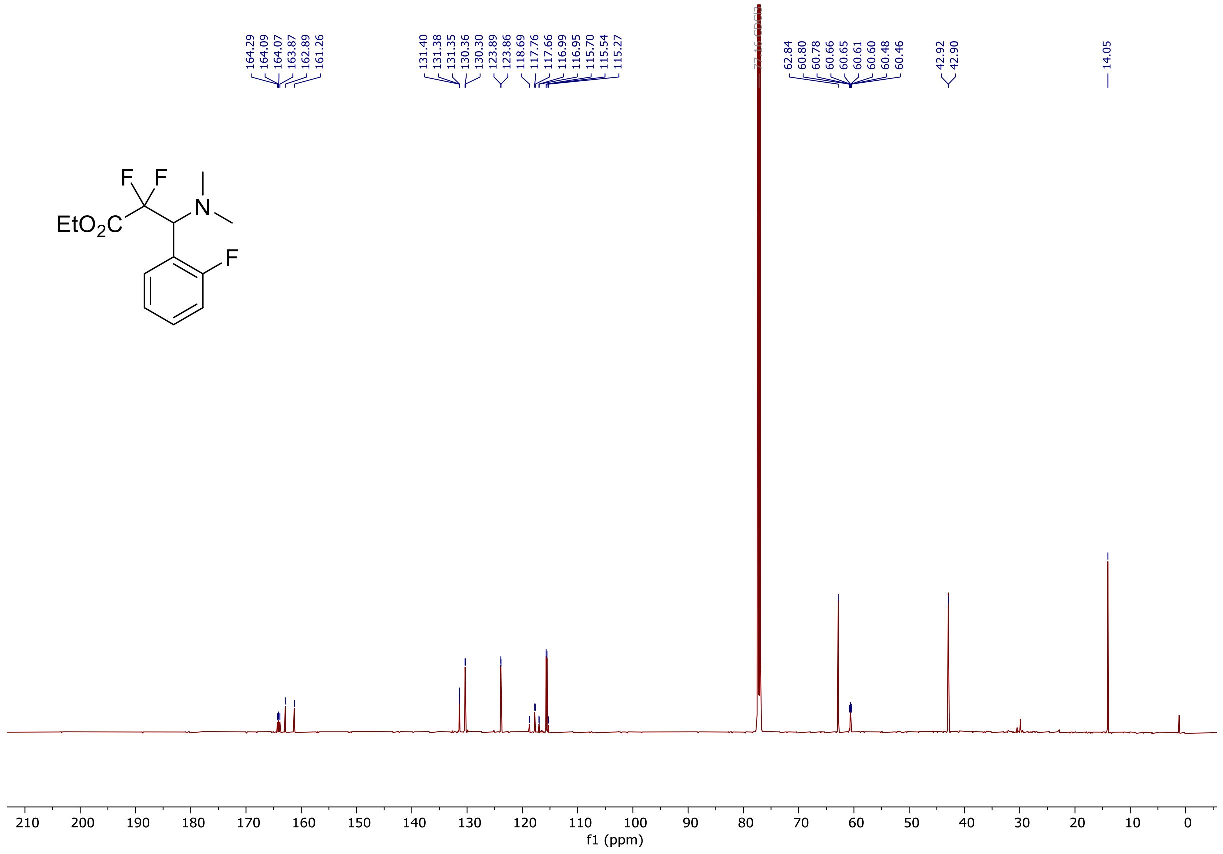

3e – ^{19}F NMR (377 MHz, $CDCl_3$)

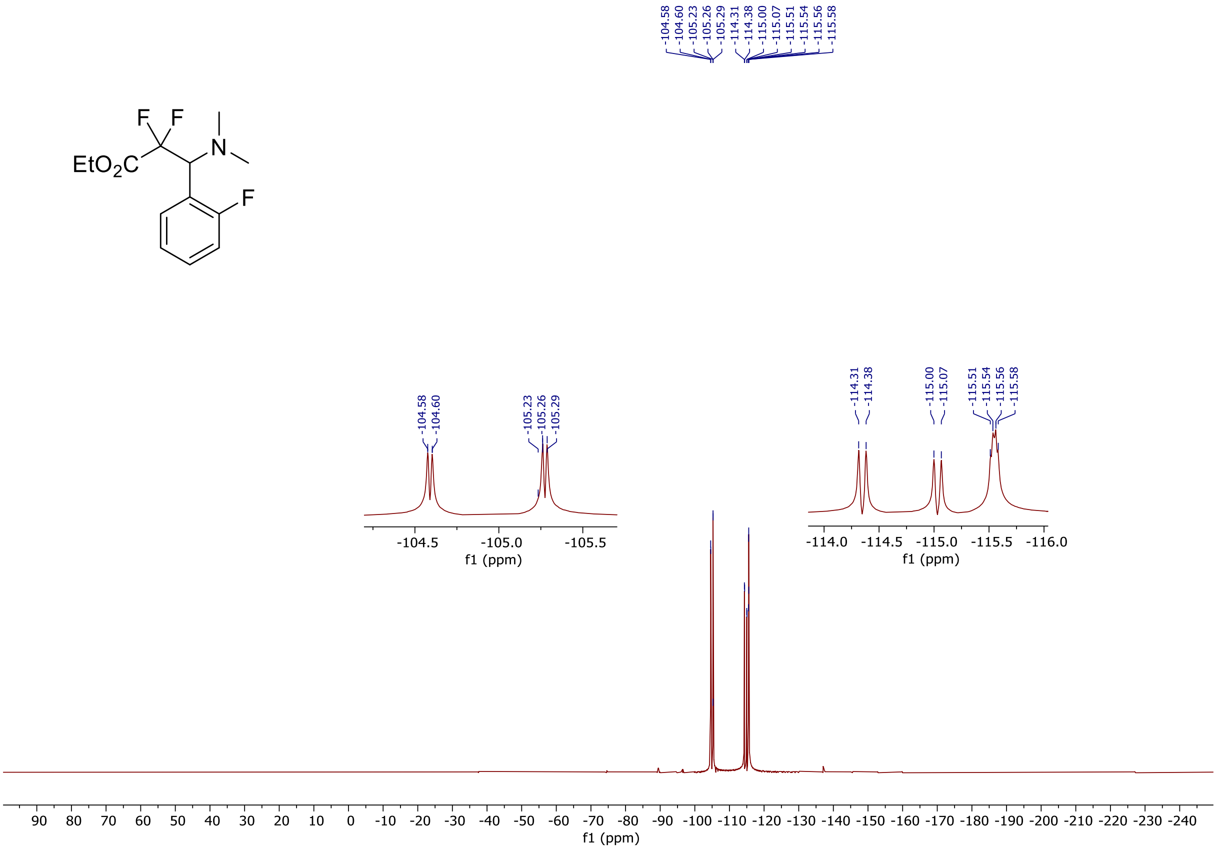

3f – ^{1}H NMR (400 MHz, $CDCl_3$)

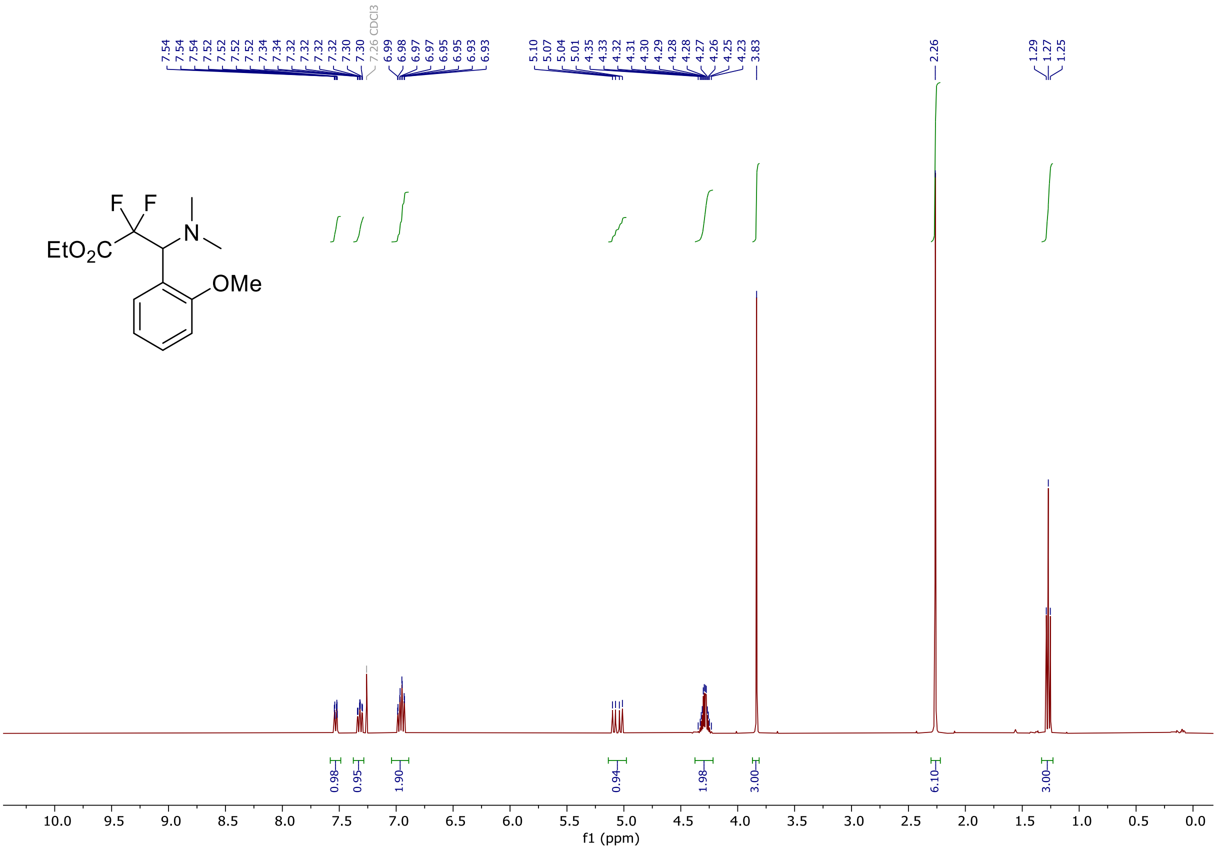

3f – ^{13}C NMR (101 MHz, $CDCl_3$)

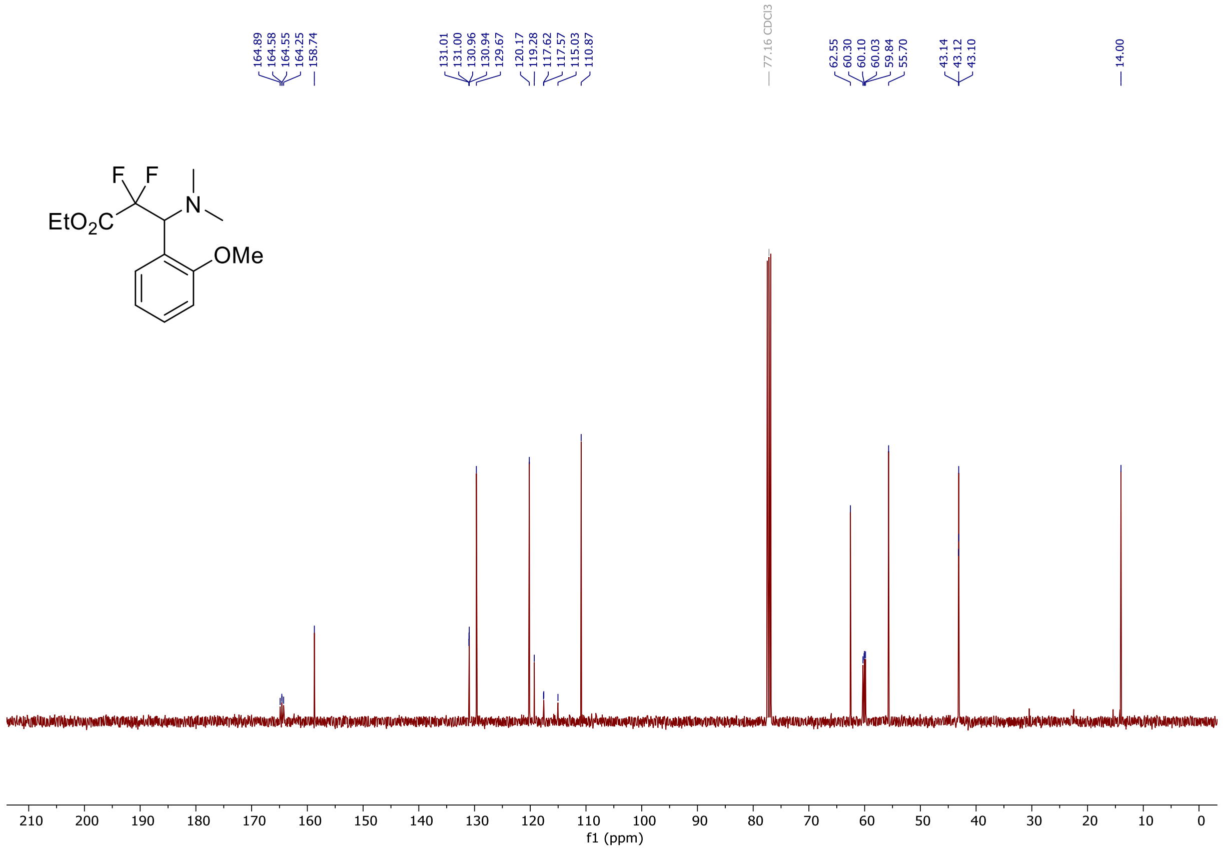

3f – ^{19}F NMR (377 MHz, $CDCl_3$)

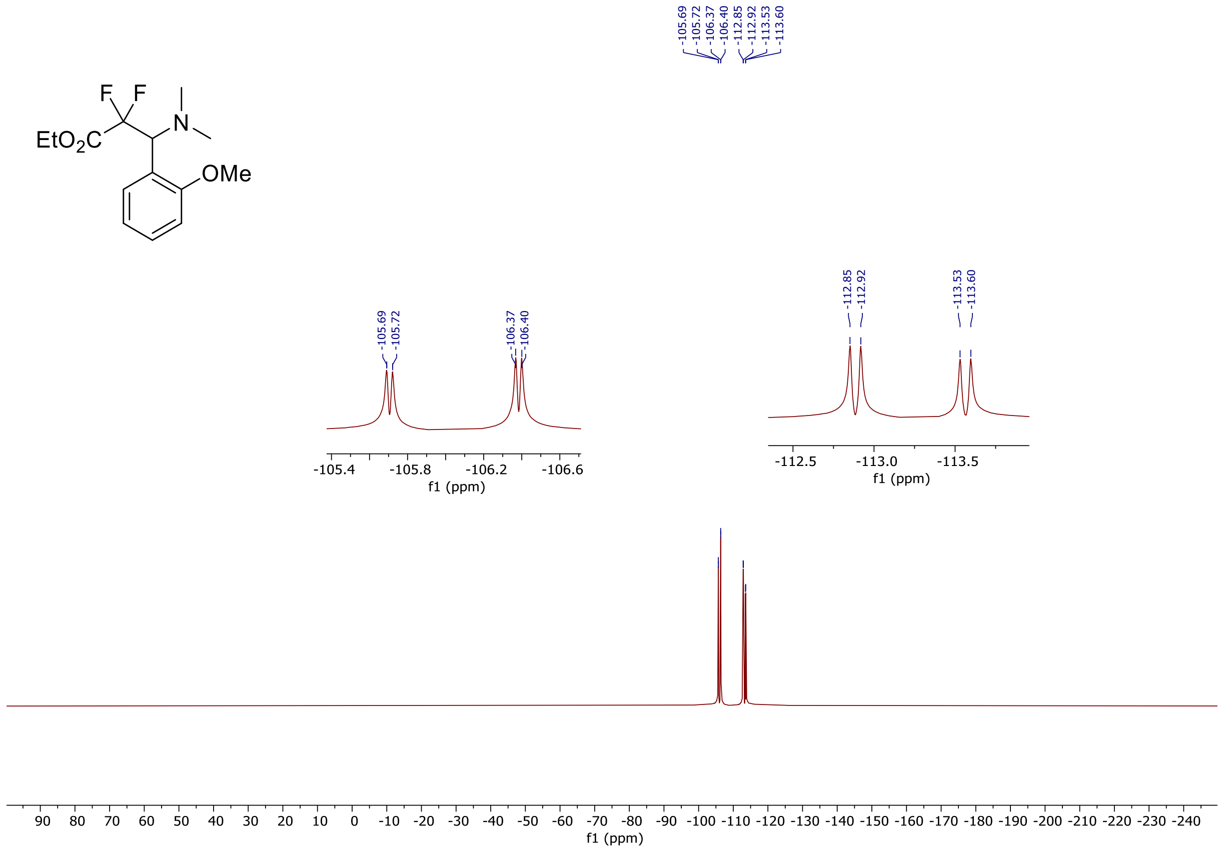

3g – ^{1}H NMR (400 MHz, $CDCl_3$)

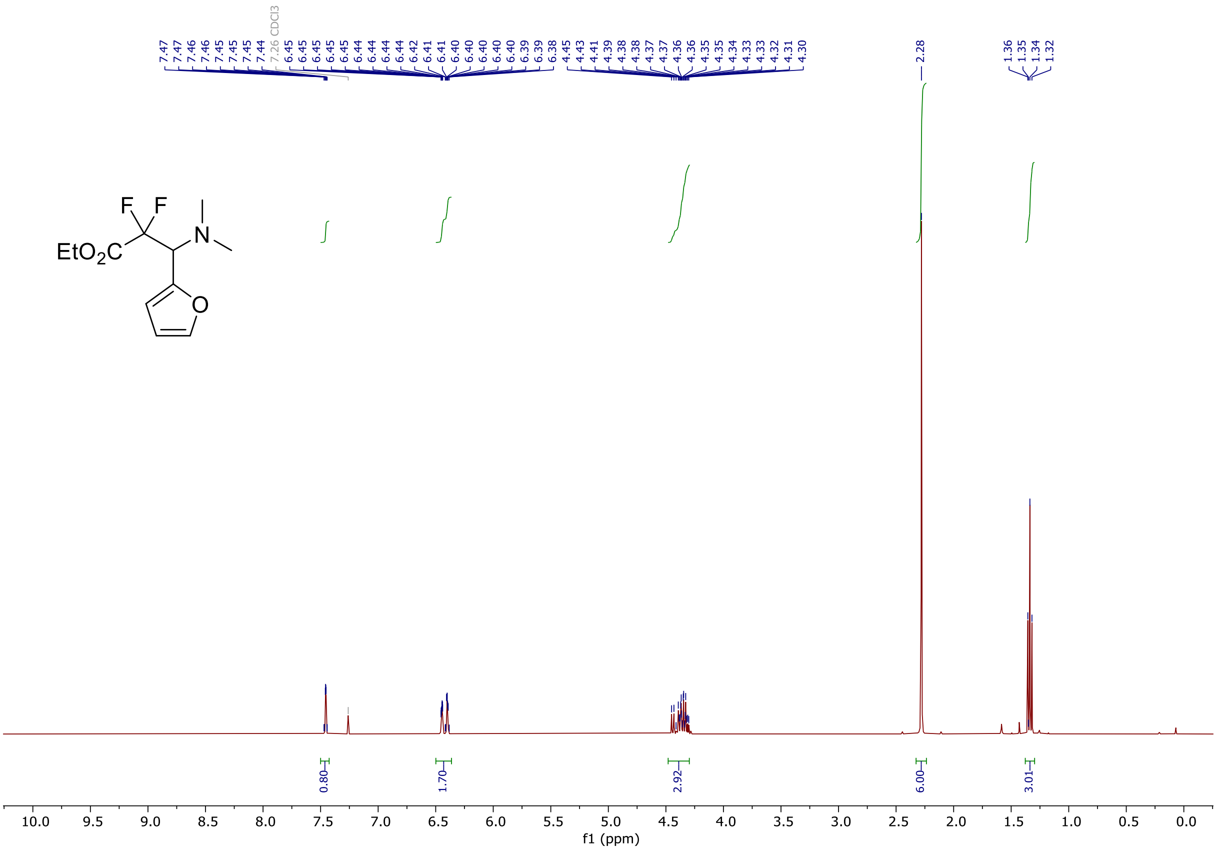

3g – ^{13}C NMR (101 MHz, $CDCl_3$)

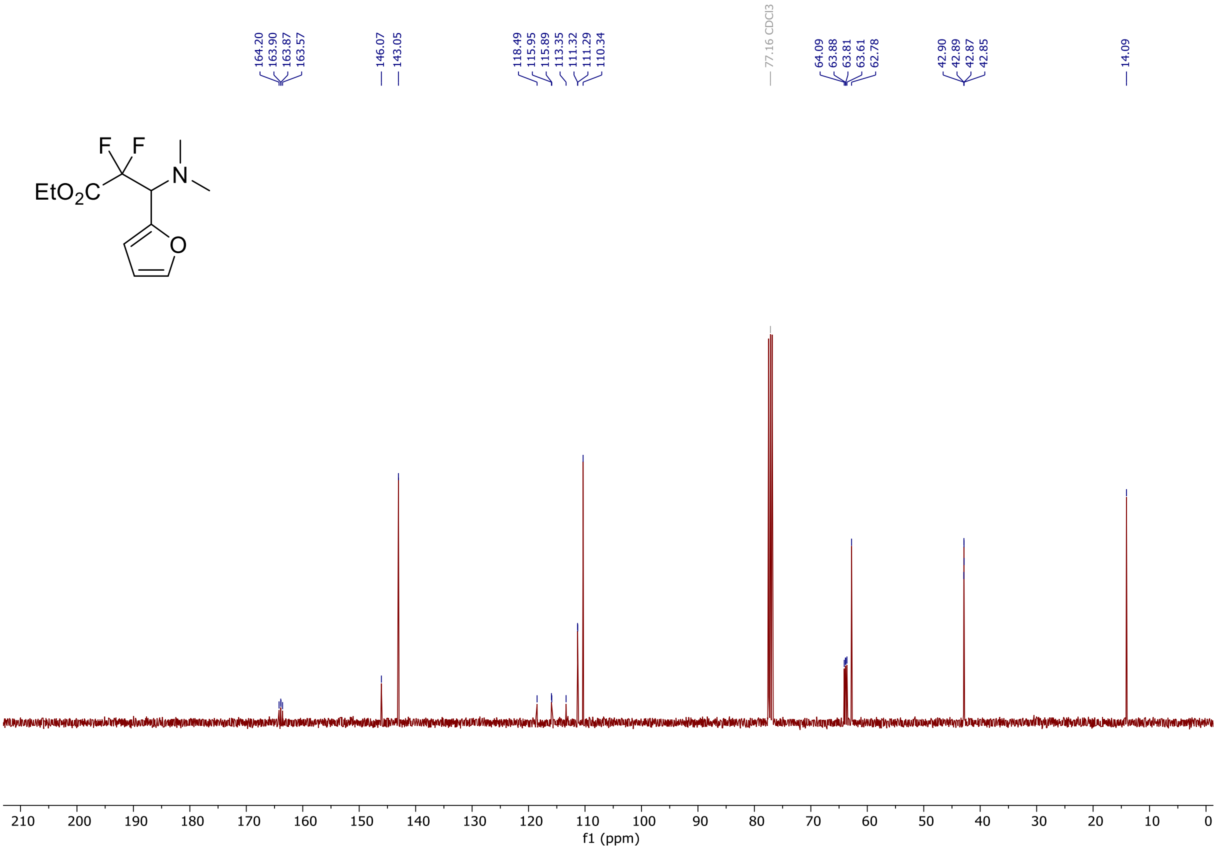

3g – ^{19}F NMR (377 MHz, $CDCl_3$)

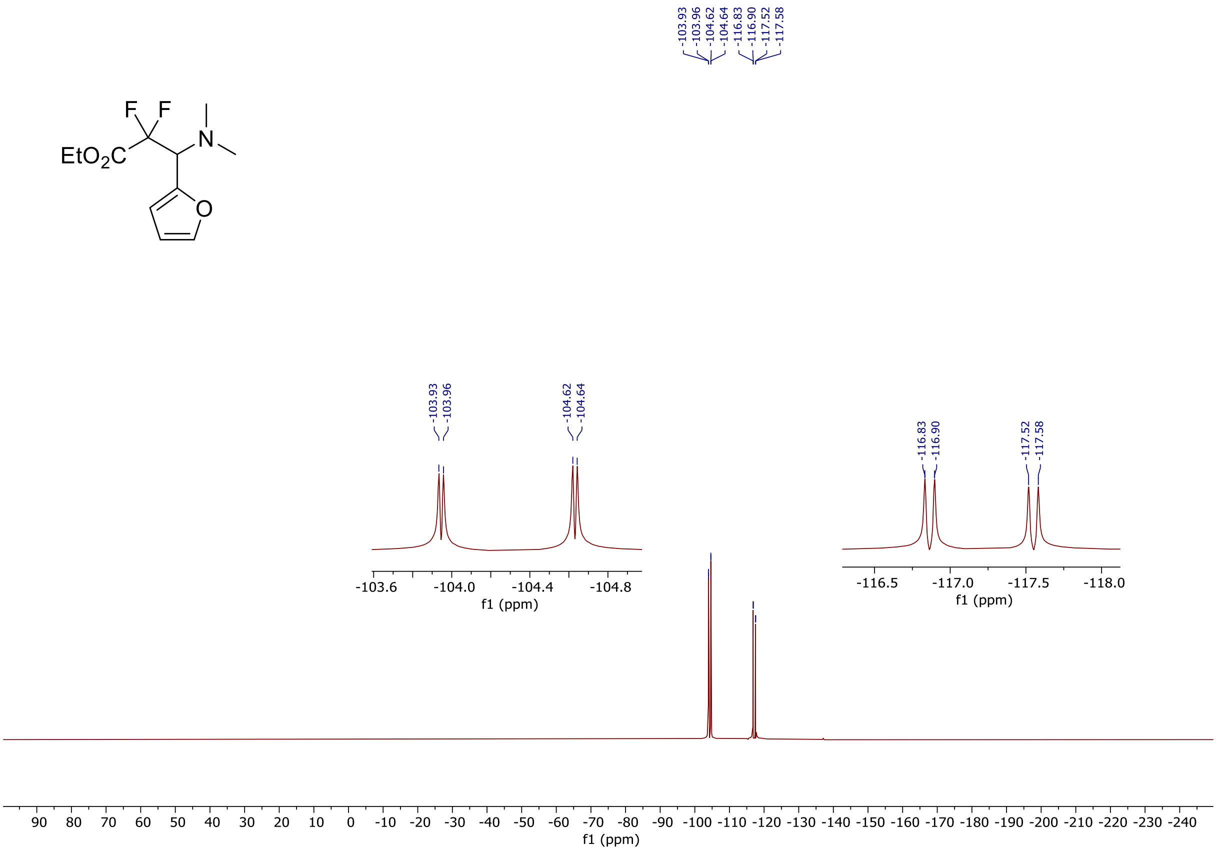

3h – ^{1}H NMR (400 MHz, $CDCl_3$)

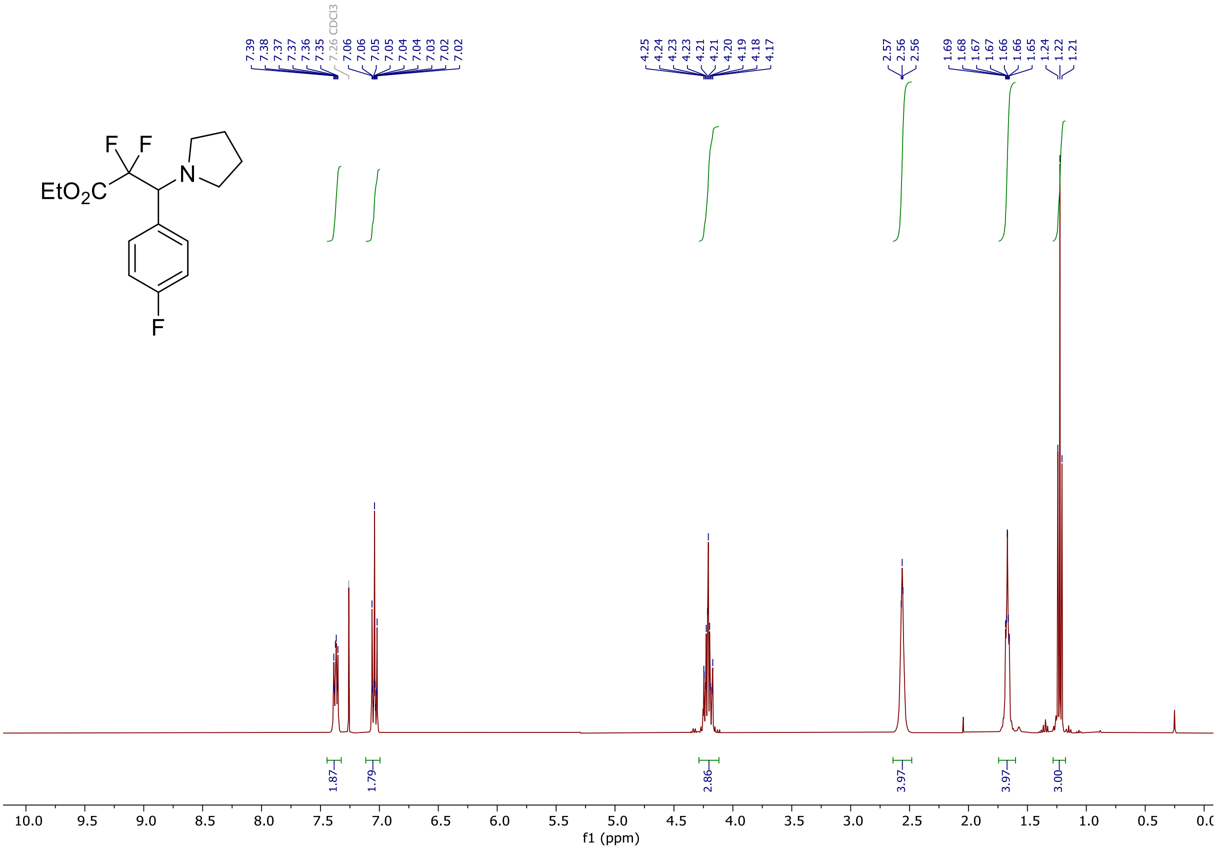

3h – ^{13}C NMR (126 MHz, $CDCl_3$)

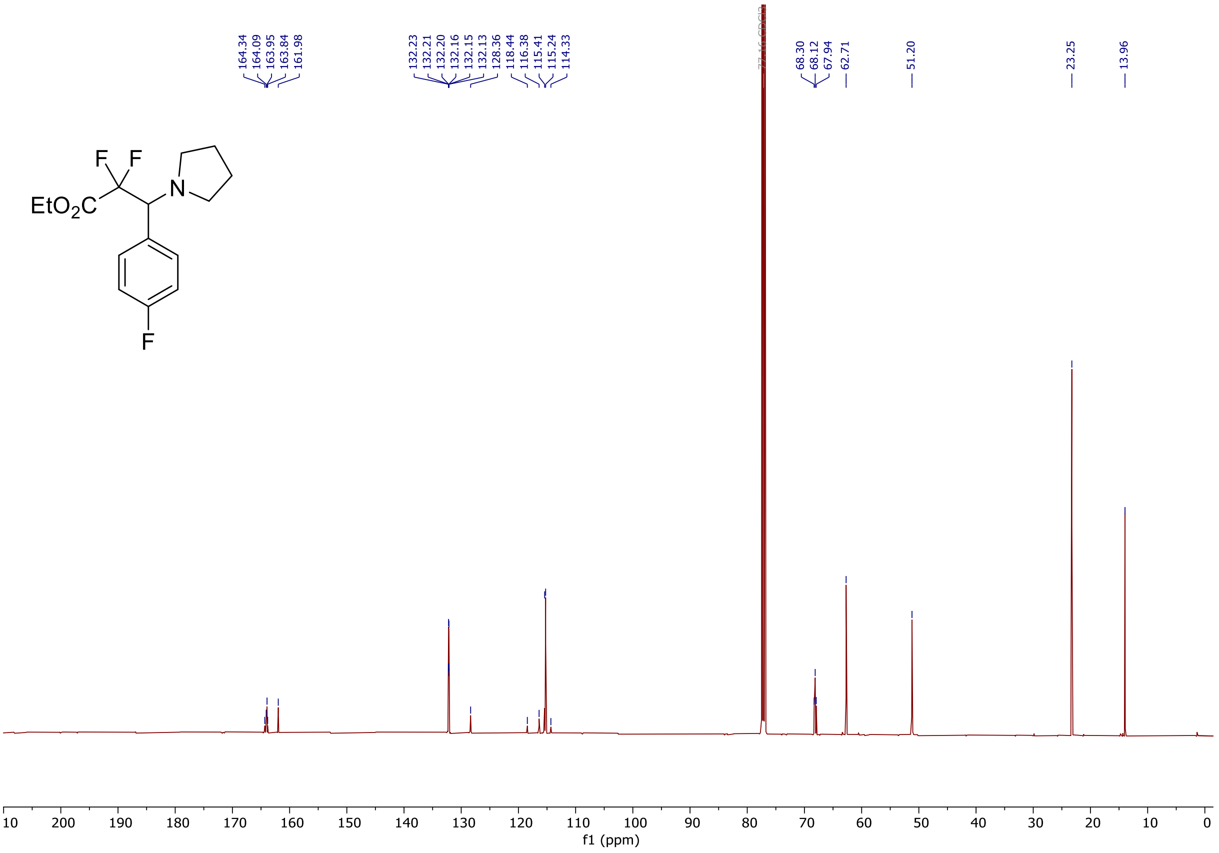

3h – ^{19}F NMR (377 MHz, $CDCl_3$)

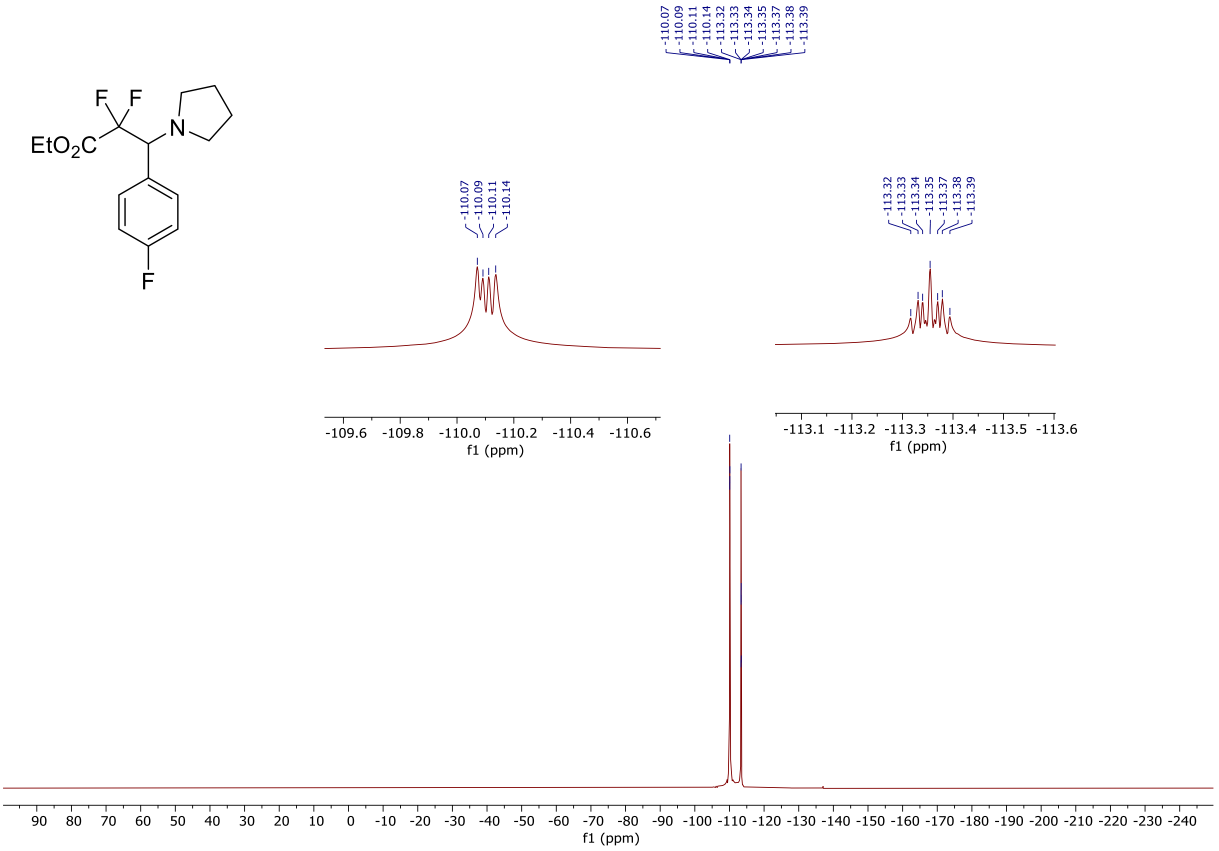

3i – ^{1}H NMR (400 MHz, $CDCl_3$)

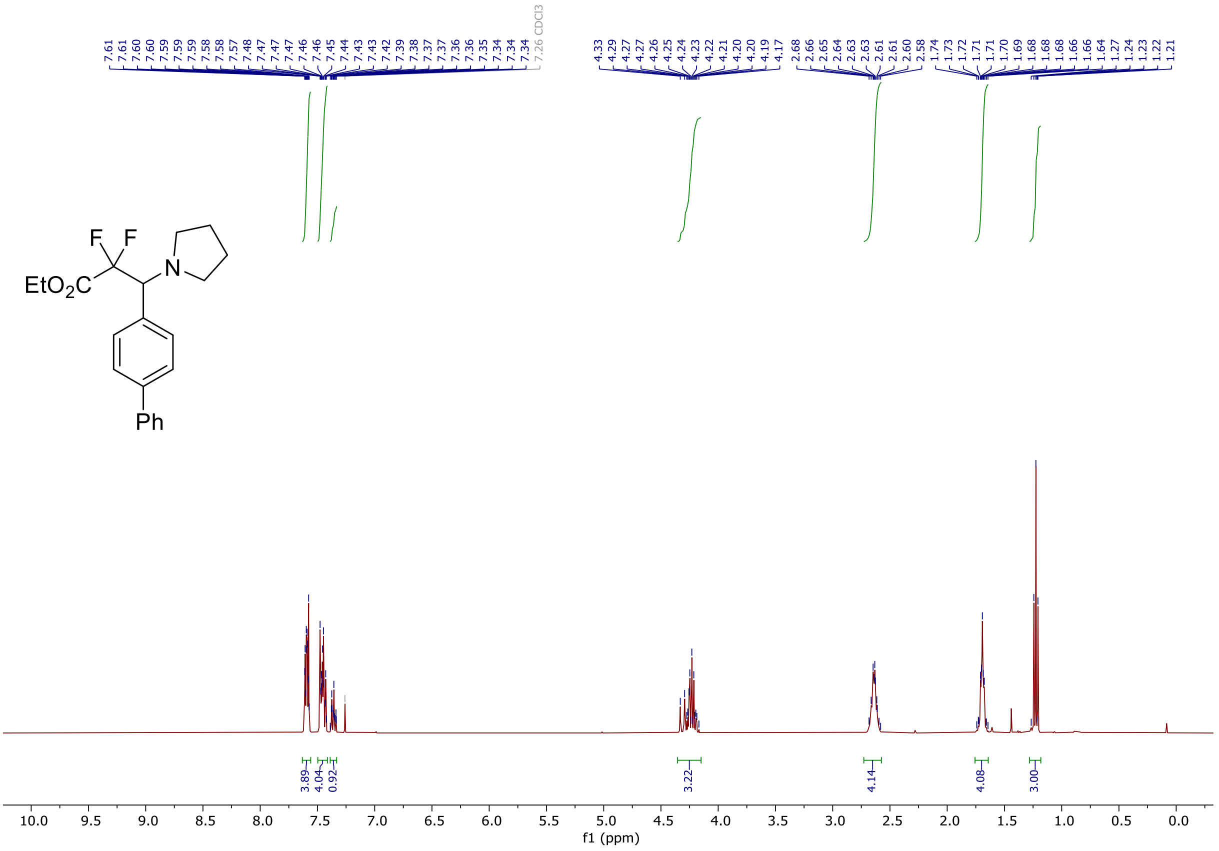

3i – ^{13}C NMR (126 MHz, $CDCl_3$)

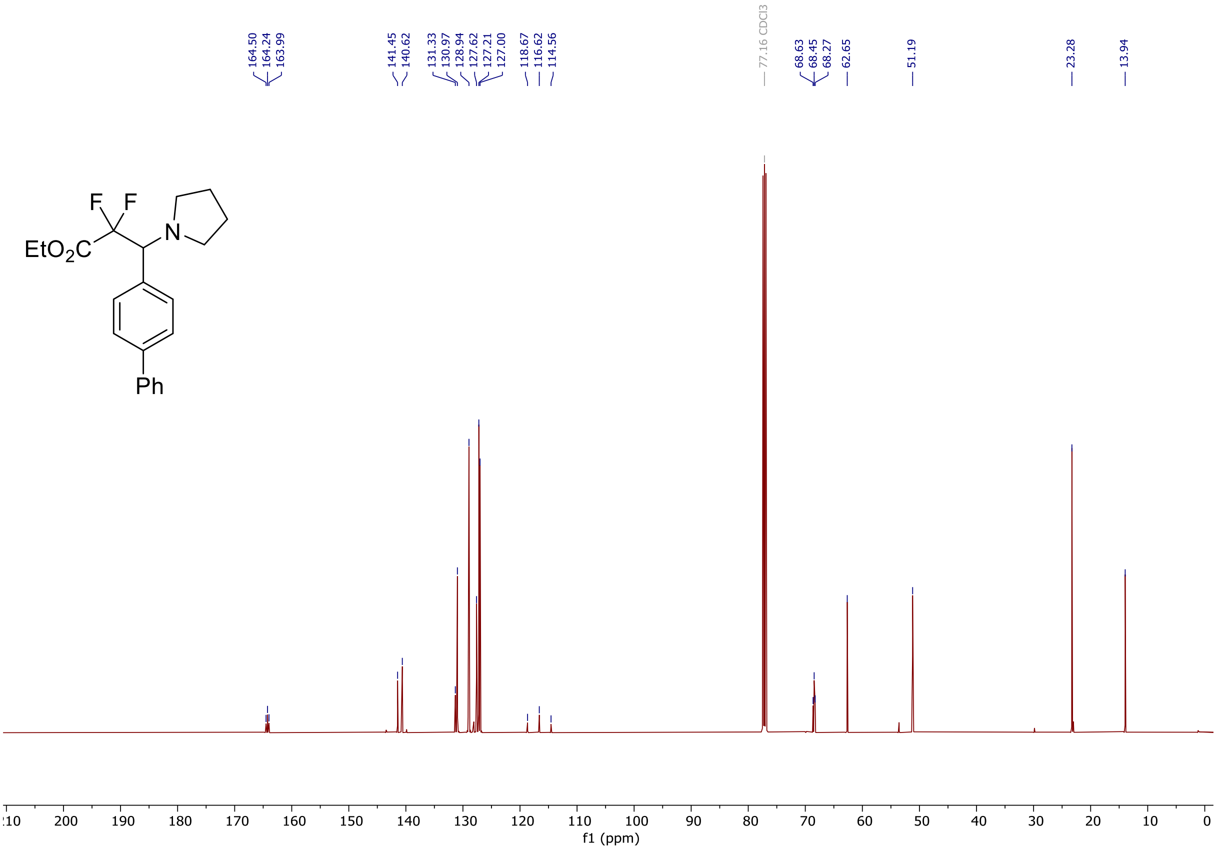

3i – ^{19}F NMR (377 MHz, $CDCl_3$)

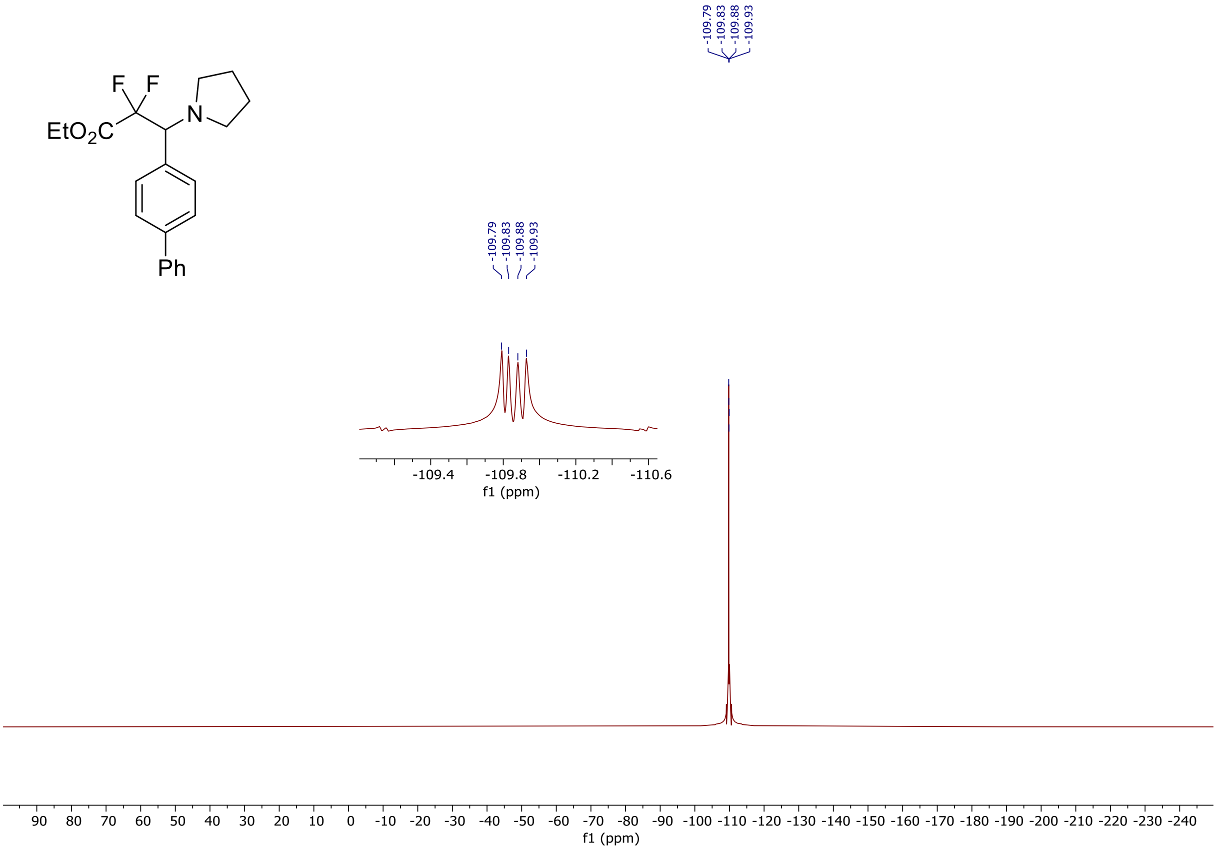

3j – ^{1}H NMR (400 MHz, $CDCl_3$)

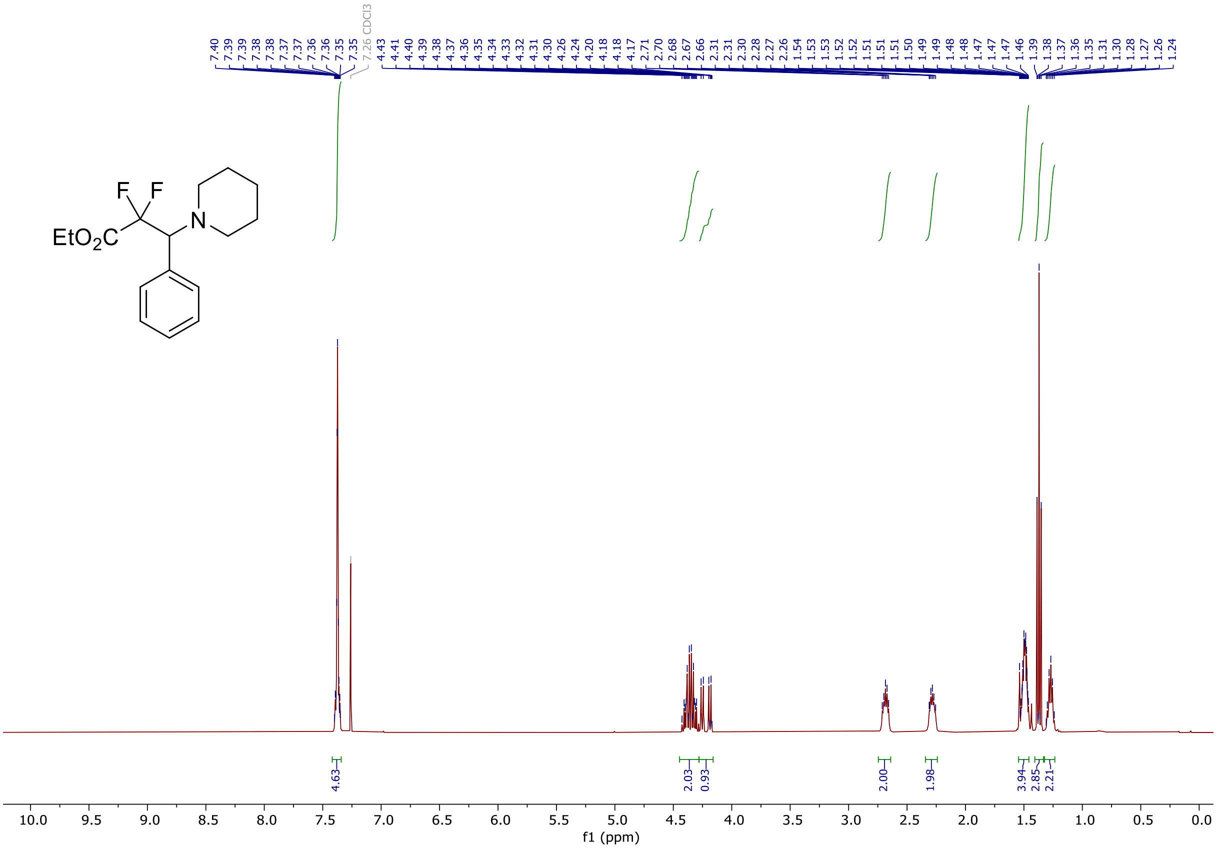

3j – ^{13}C NMR (126 MHz, $CDCl_3$)

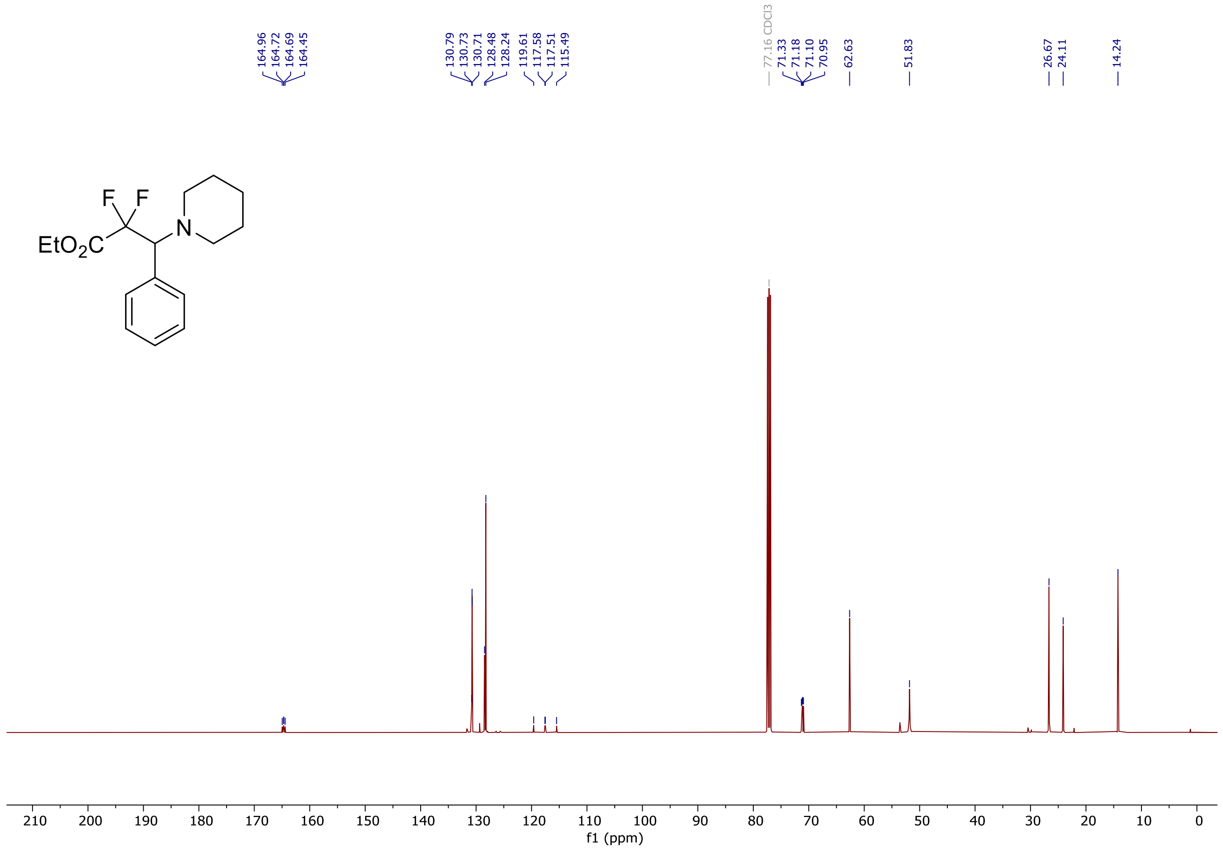

3j – ^{19}F NMR (377 MHz, $CDCl_3$)

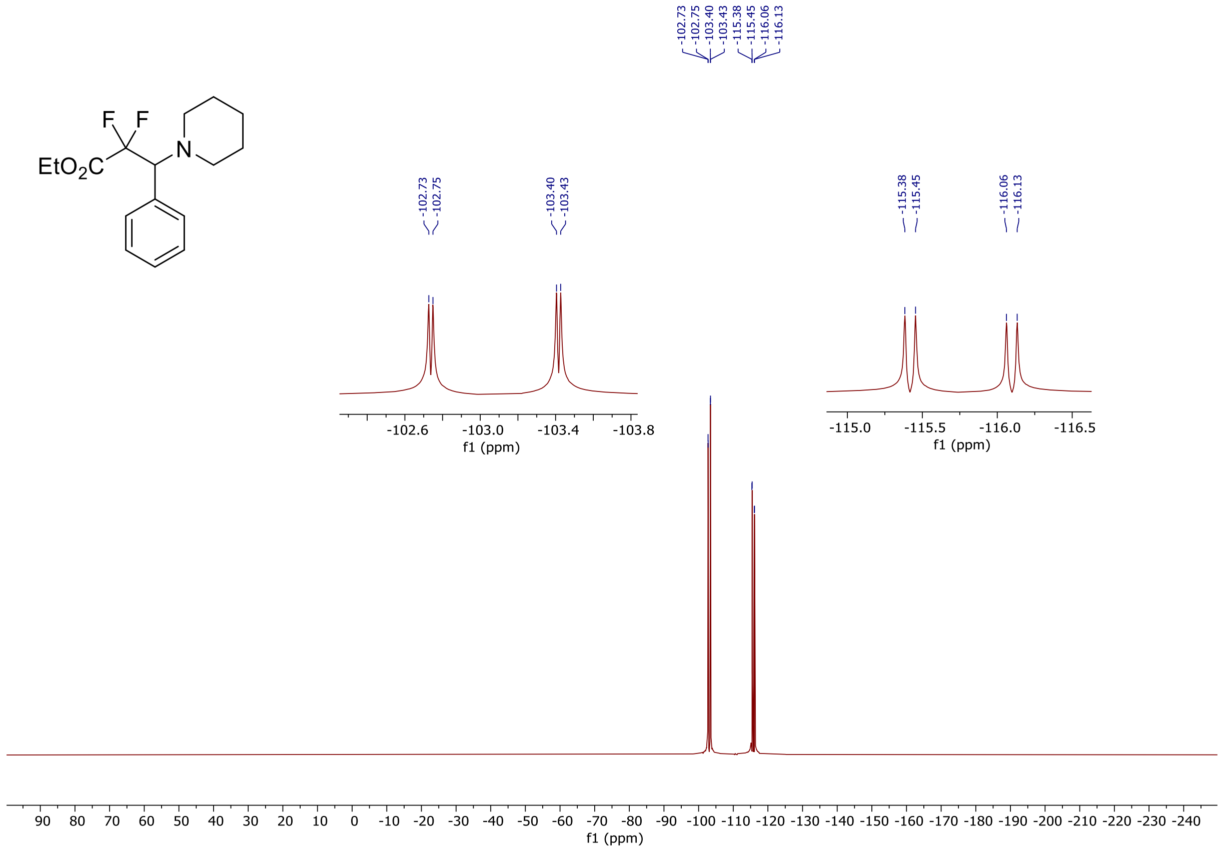

3k – ^{1}H NMR (400 MHz, $CDCl_3$)

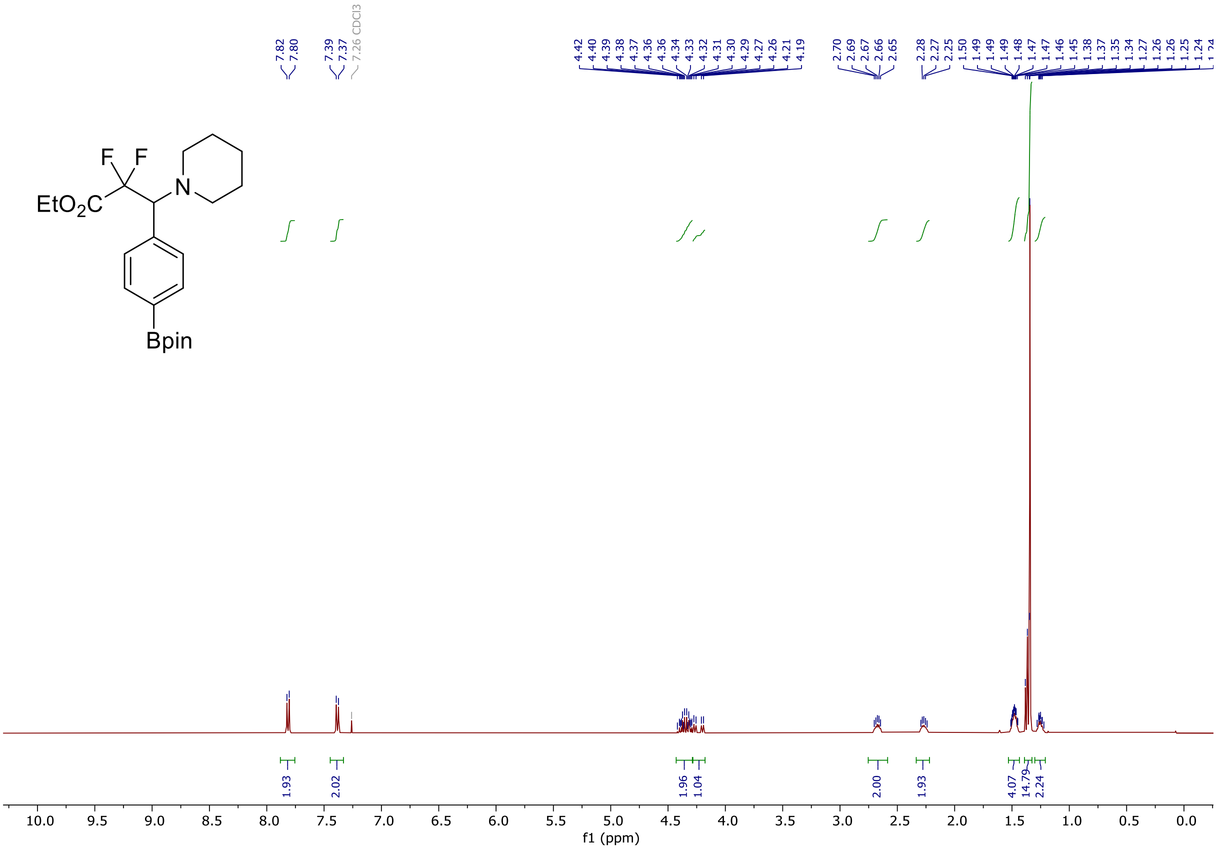

3k – ^{13}C NMR (101 MHz, $CDCl_3$)

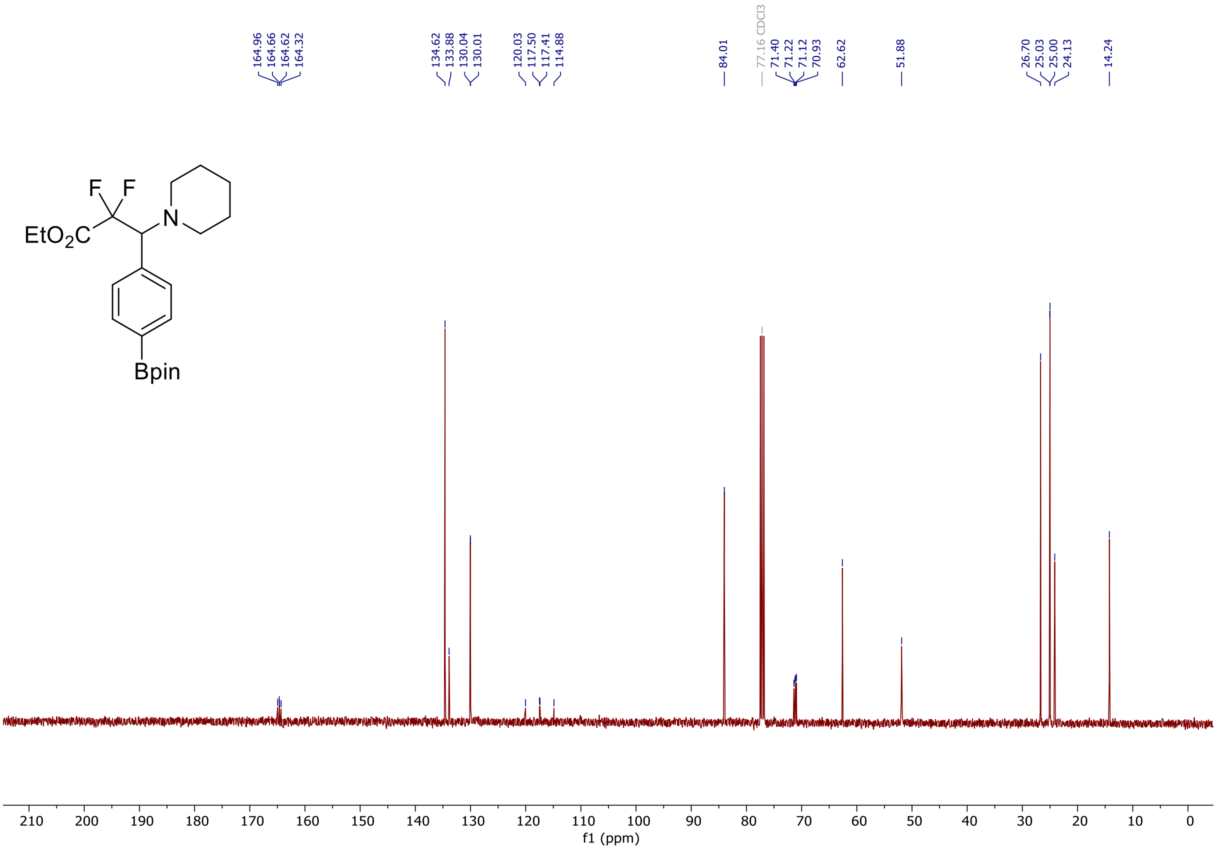

3k – ^{19}F NMR (377 MHz, $CDCl_3$)

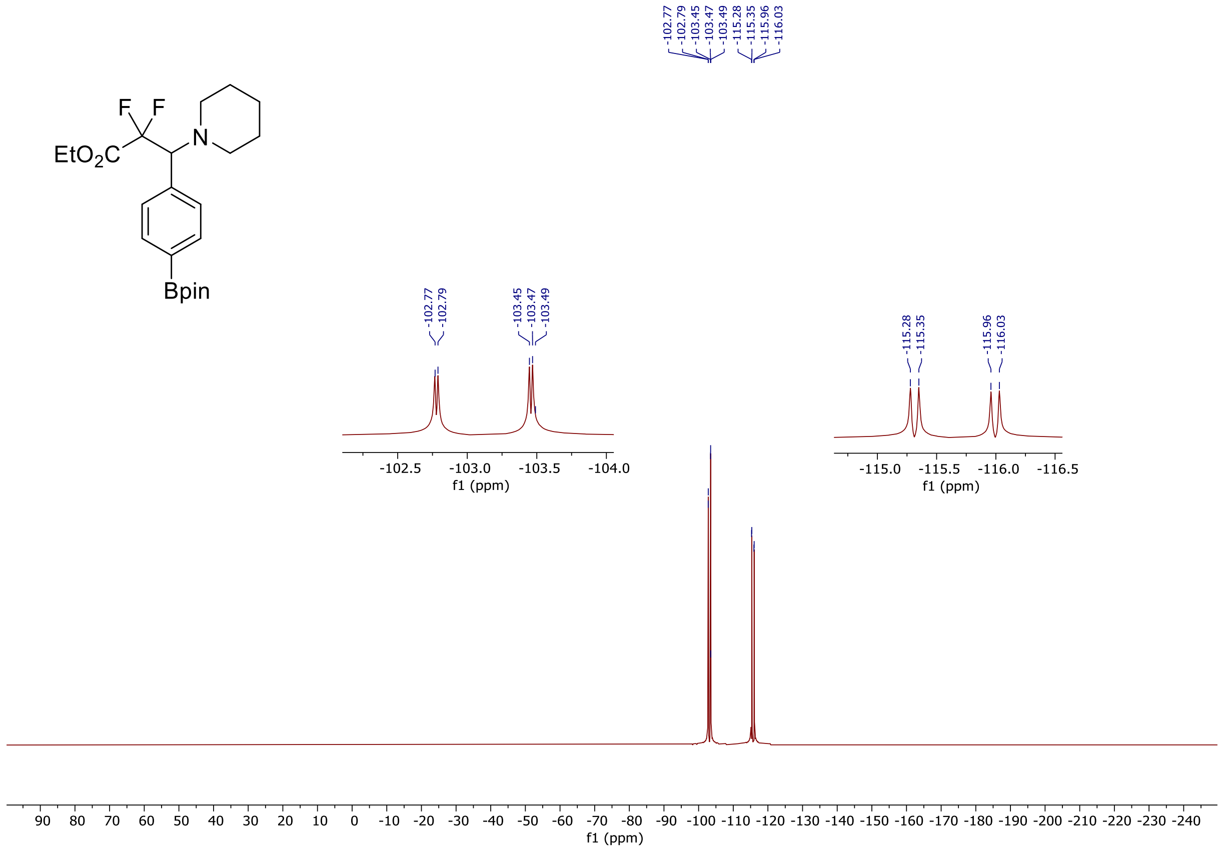

3l – ^{1}H NMR (400 MHz, $CDCl_3$)

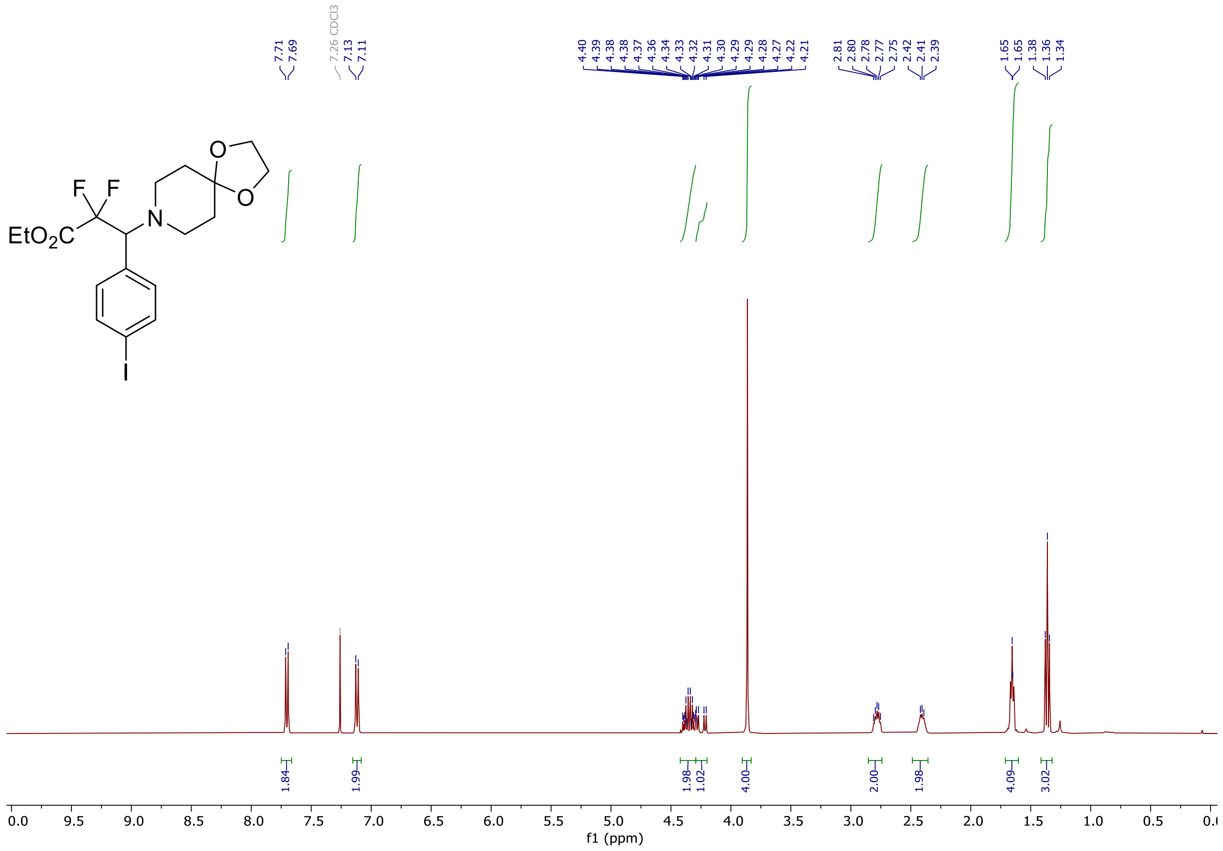

3l – ^{13}C NMR (126 MHz, $CDCl_3$)

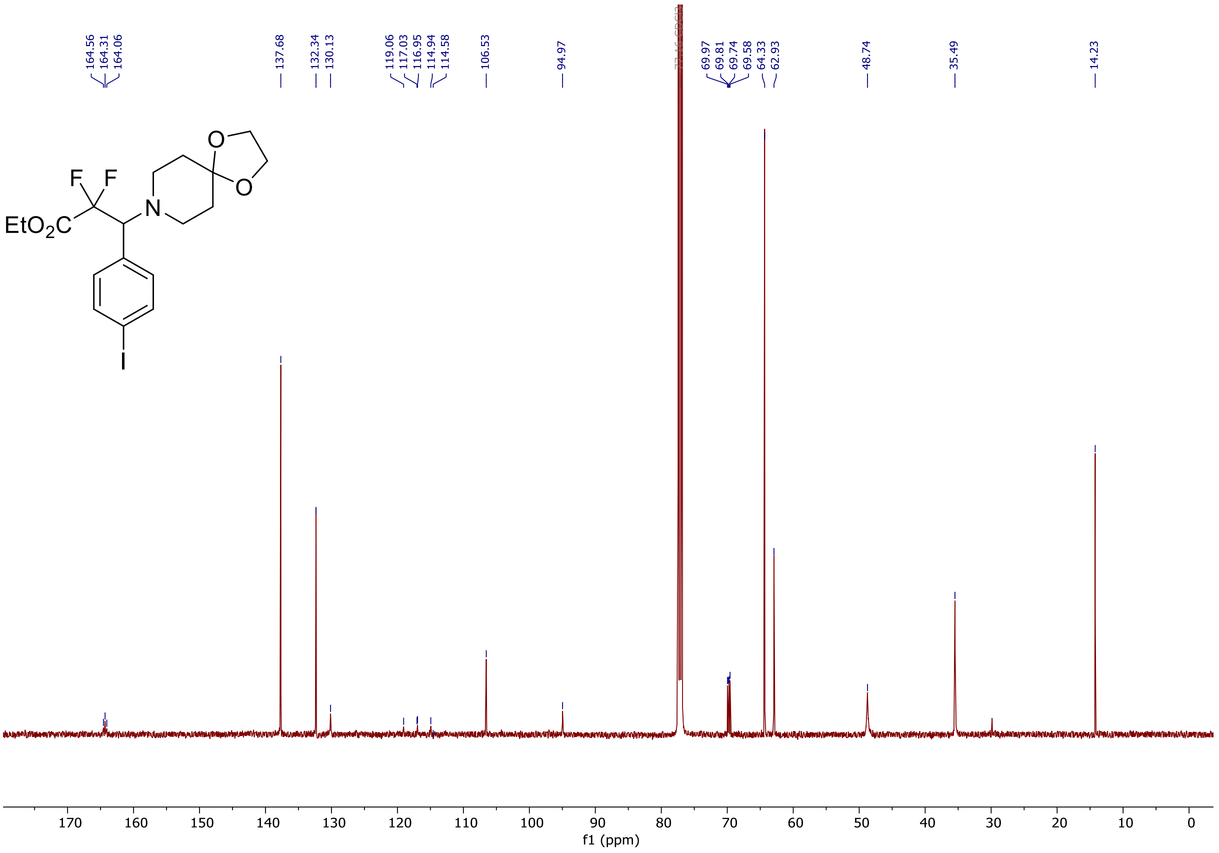

3l – ^{19}F NMR (377 MHz, $CDCl_3$)

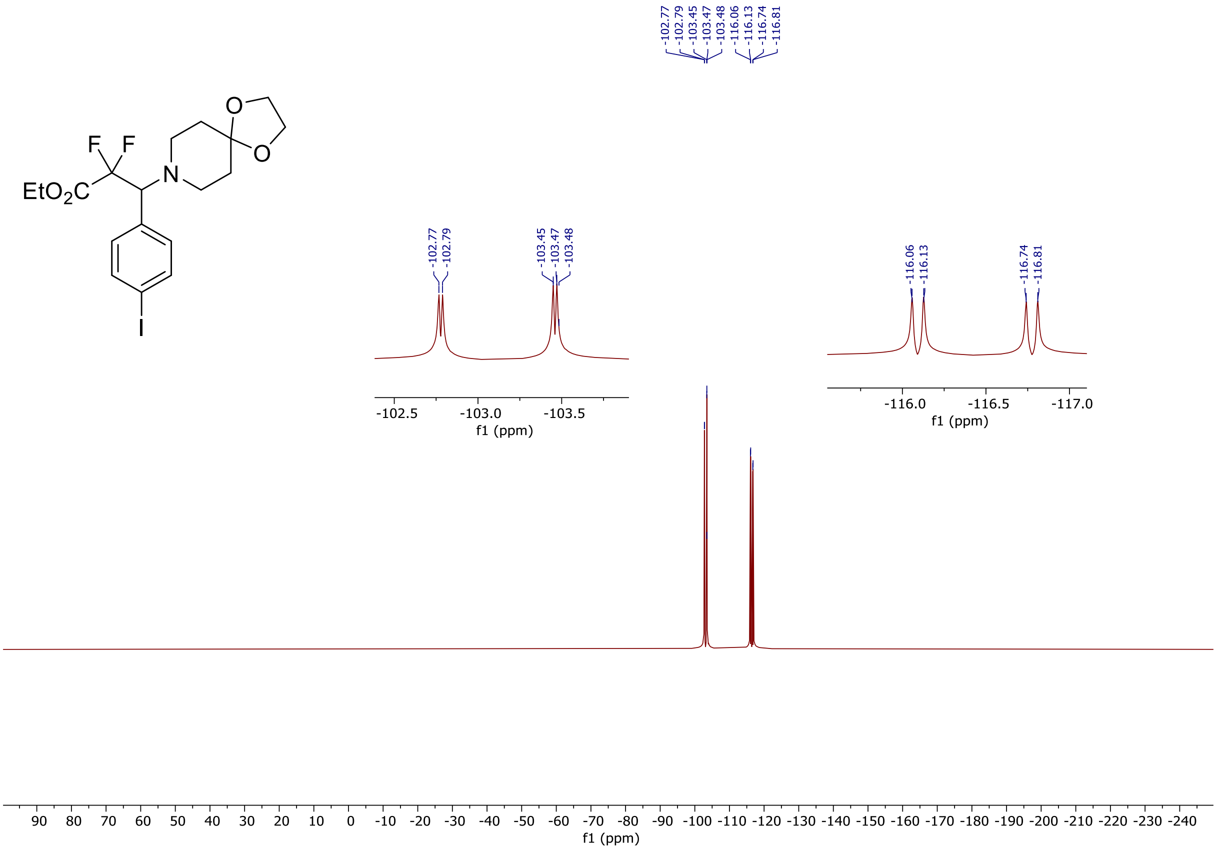

3m – ^{1}H NMR (400 MHz, $CDCl_3$)

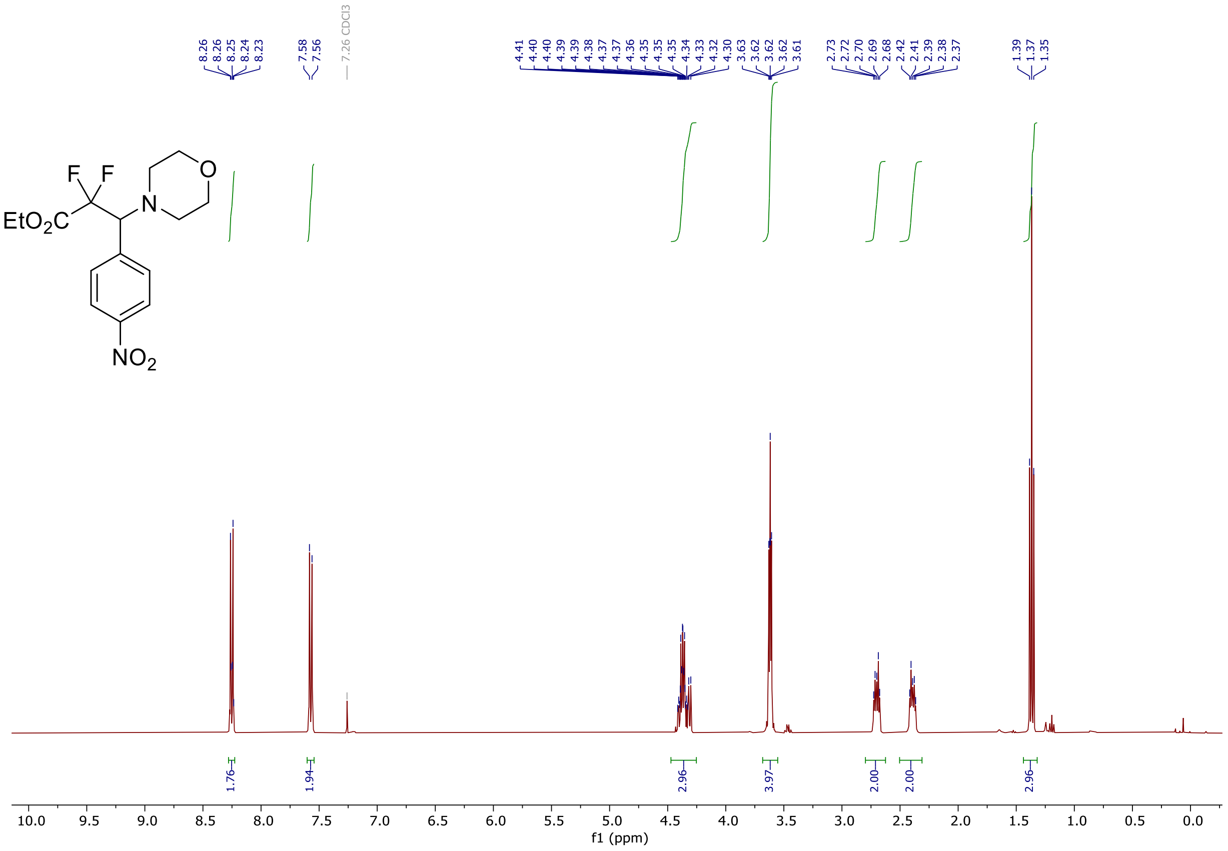

3m – ^{13}C NMR (101 MHz, $CDCl_3$)

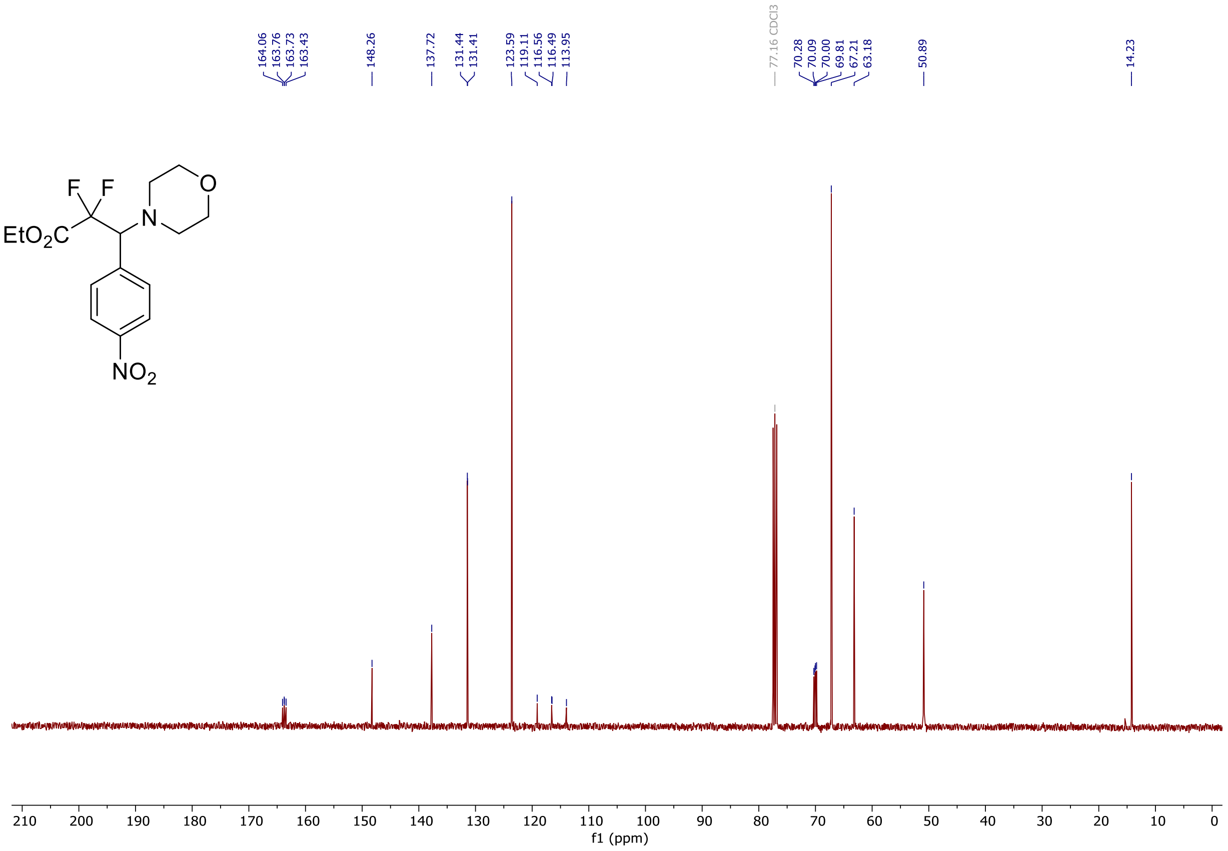

3m – ^{19}F NMR (377 MHz, $CDCl_3$)

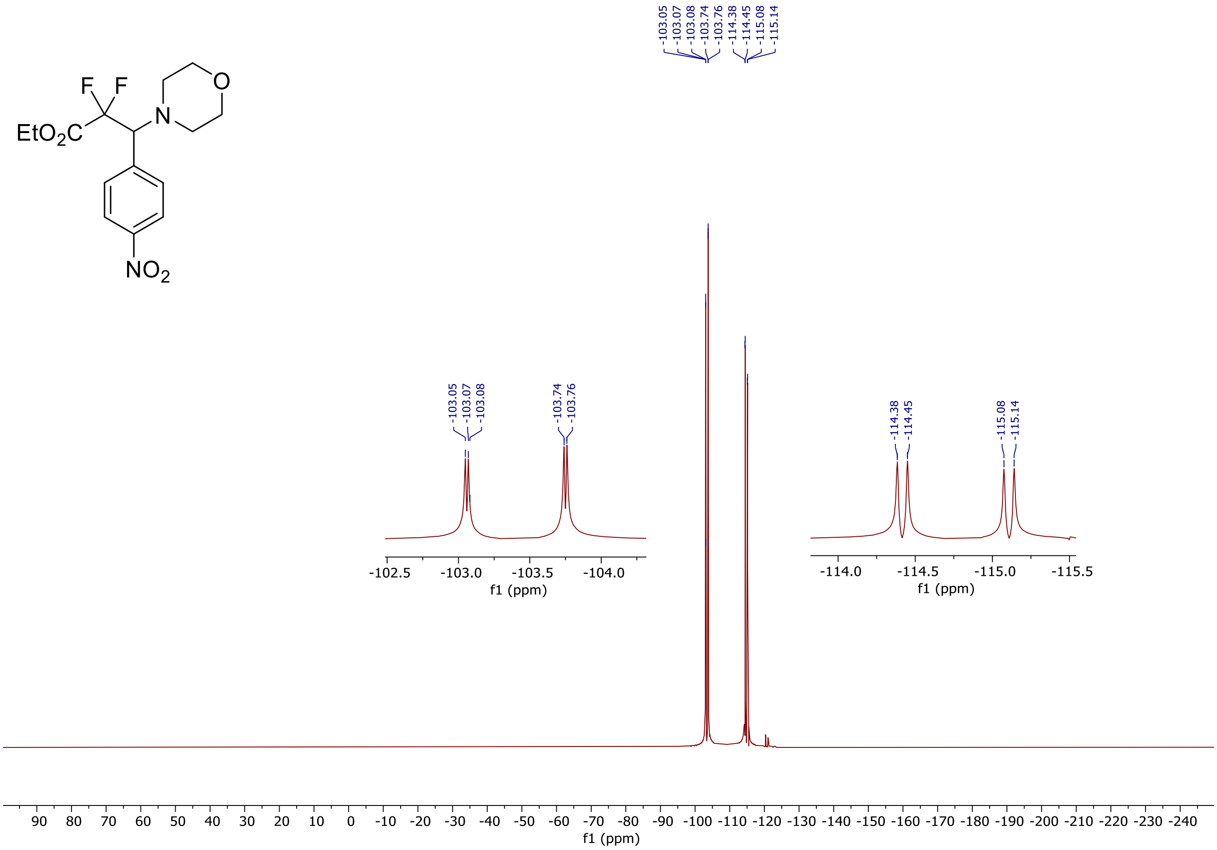

3n – ^{1}H NMR (400 MHz, $CDCl_3$)

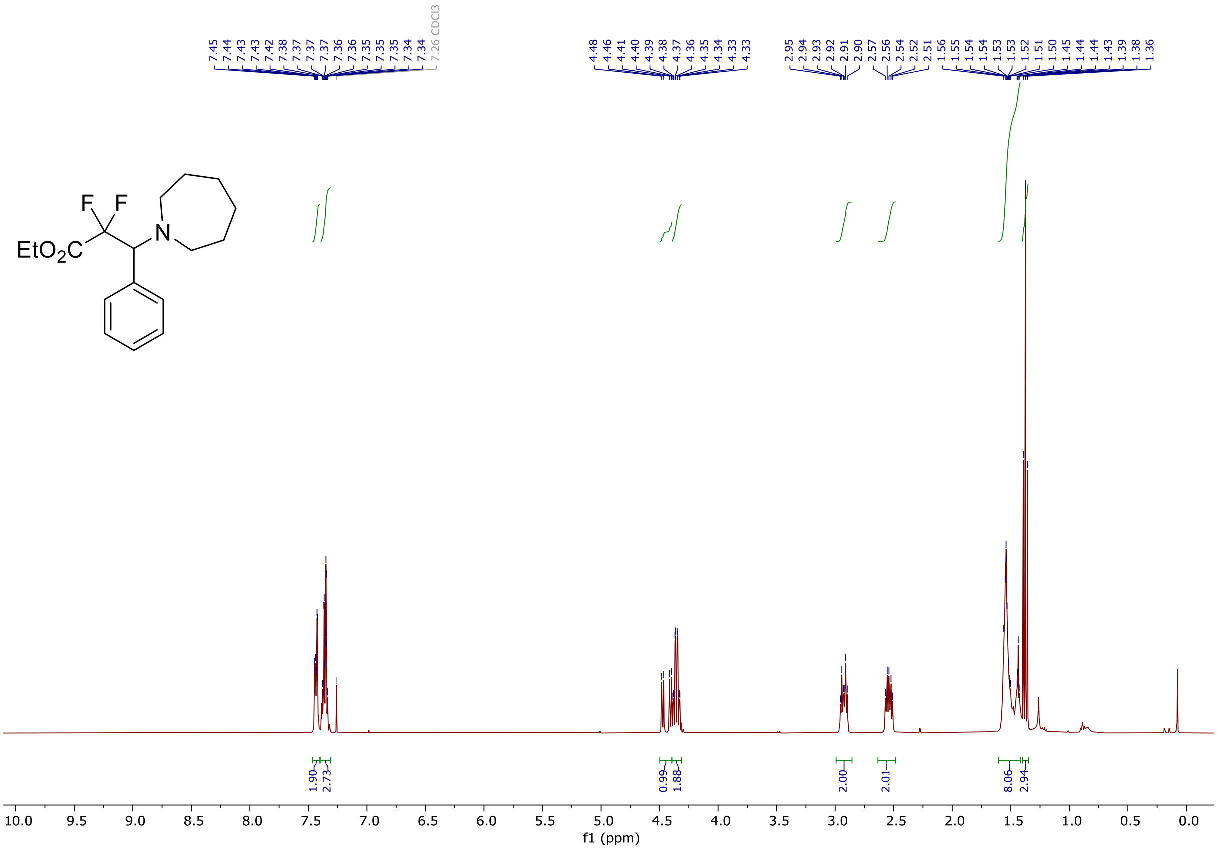

3n – ^{13}C NMR (101 MHz, CDCl$_3$)

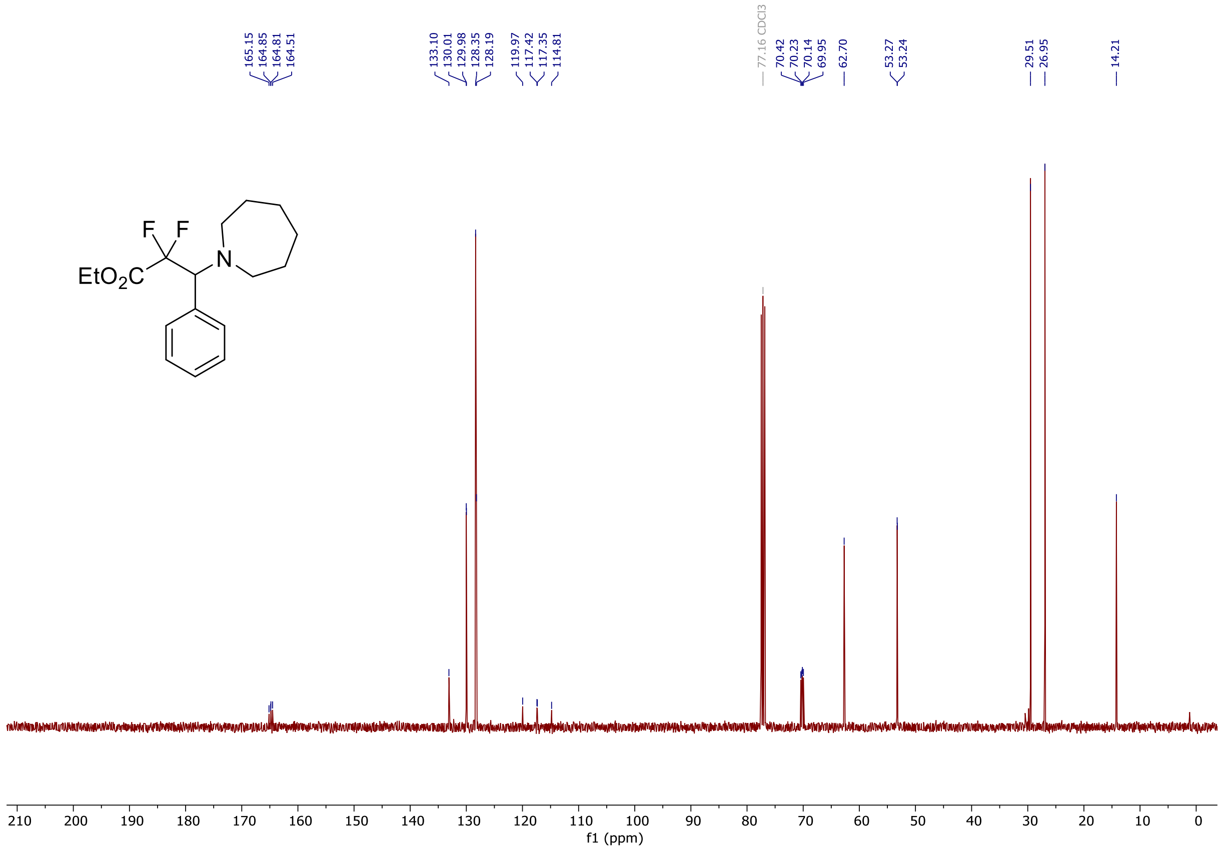

3n – ^{19}F NMR (377 MHz, CDCl$_3$)

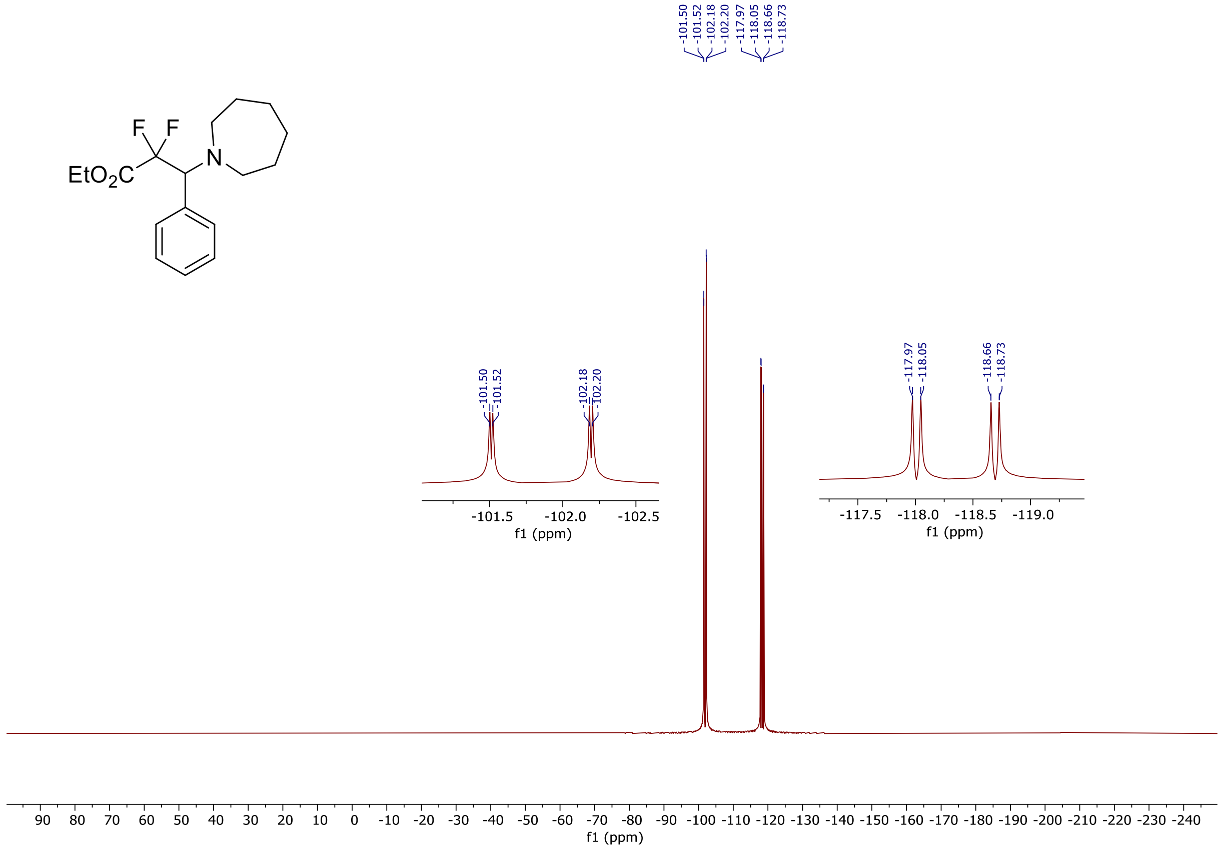

3o – ^{1}H NMR (400 MHz, $CDCl_3$)

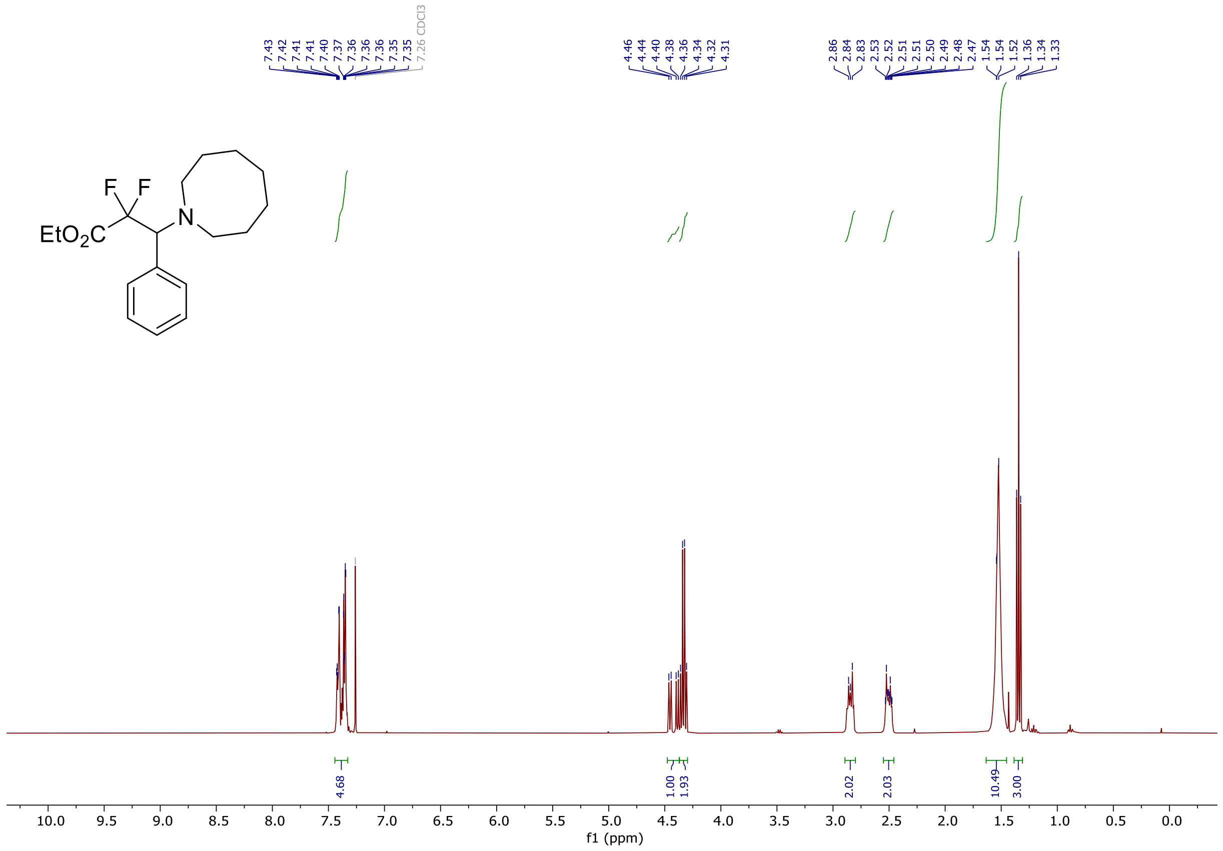

3o – ^{13}C NMR (126 MHz, $CDCl_3$)

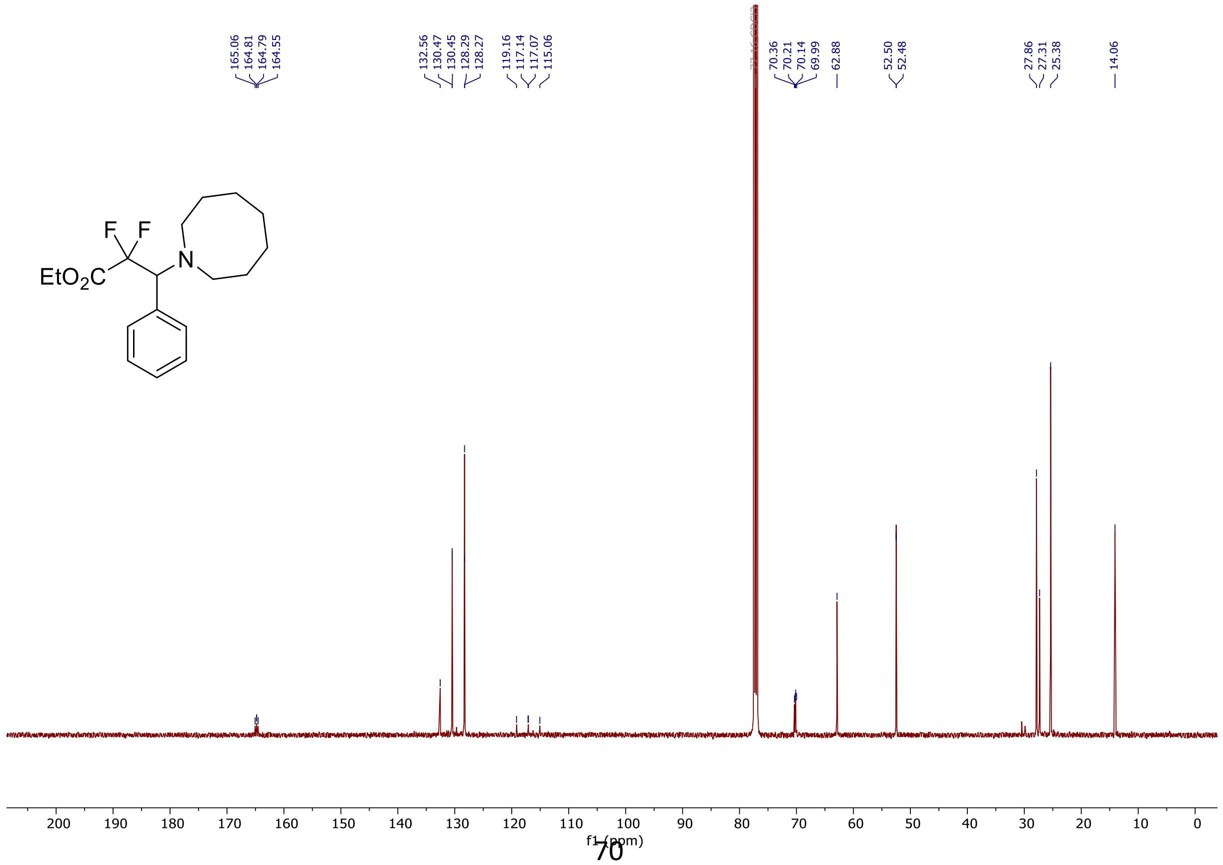

3o – ^{19}F NMR (377 MHz, CDCl$_3$)

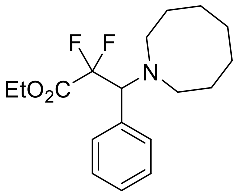

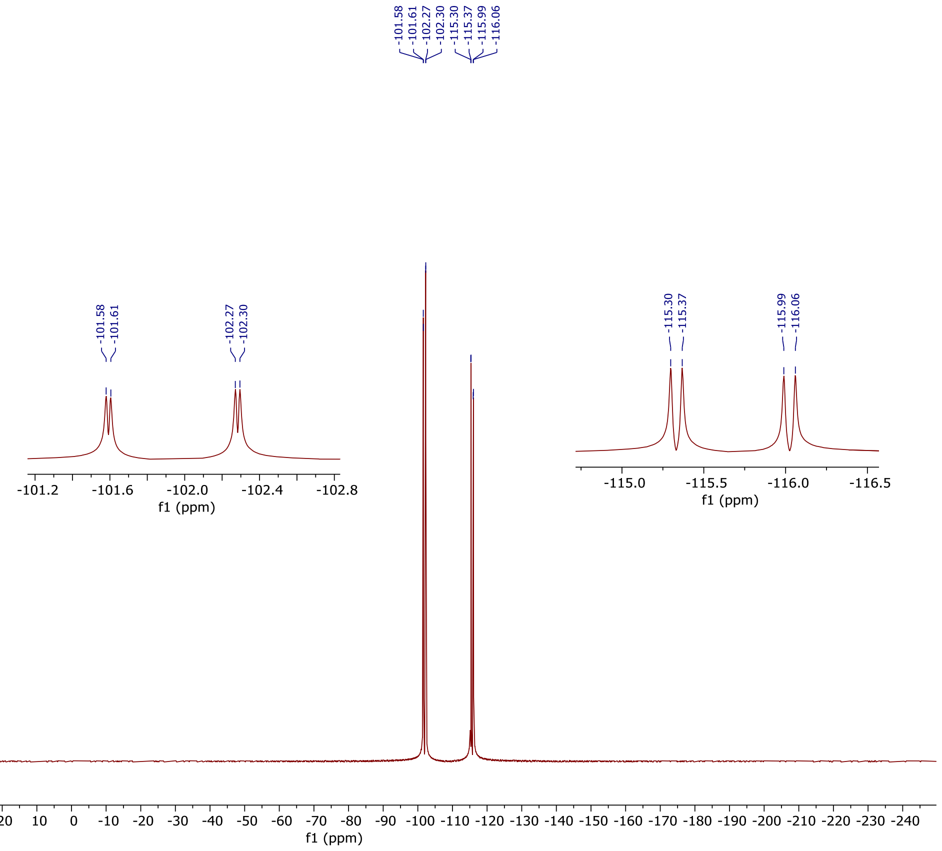

3p – ^{1}H NMR (400 MHz, CDCl$_3$)

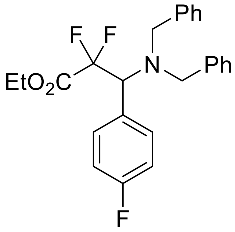

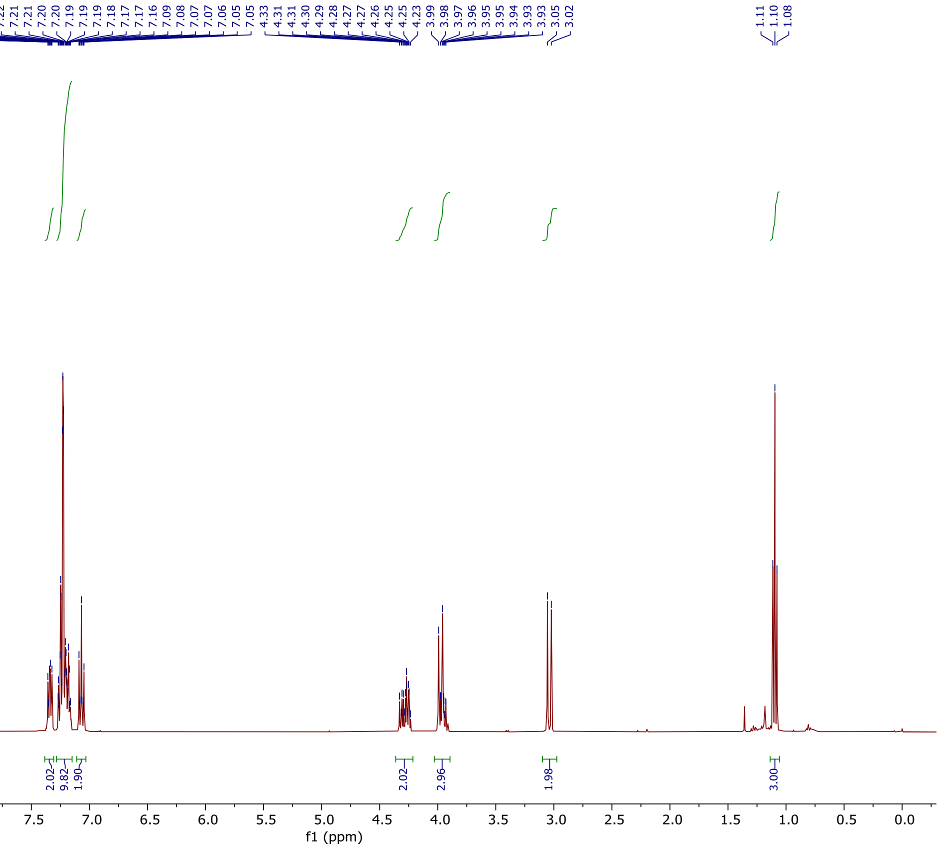

3p – ^{13}C NMR (126 MHz, $CDCl_3$)

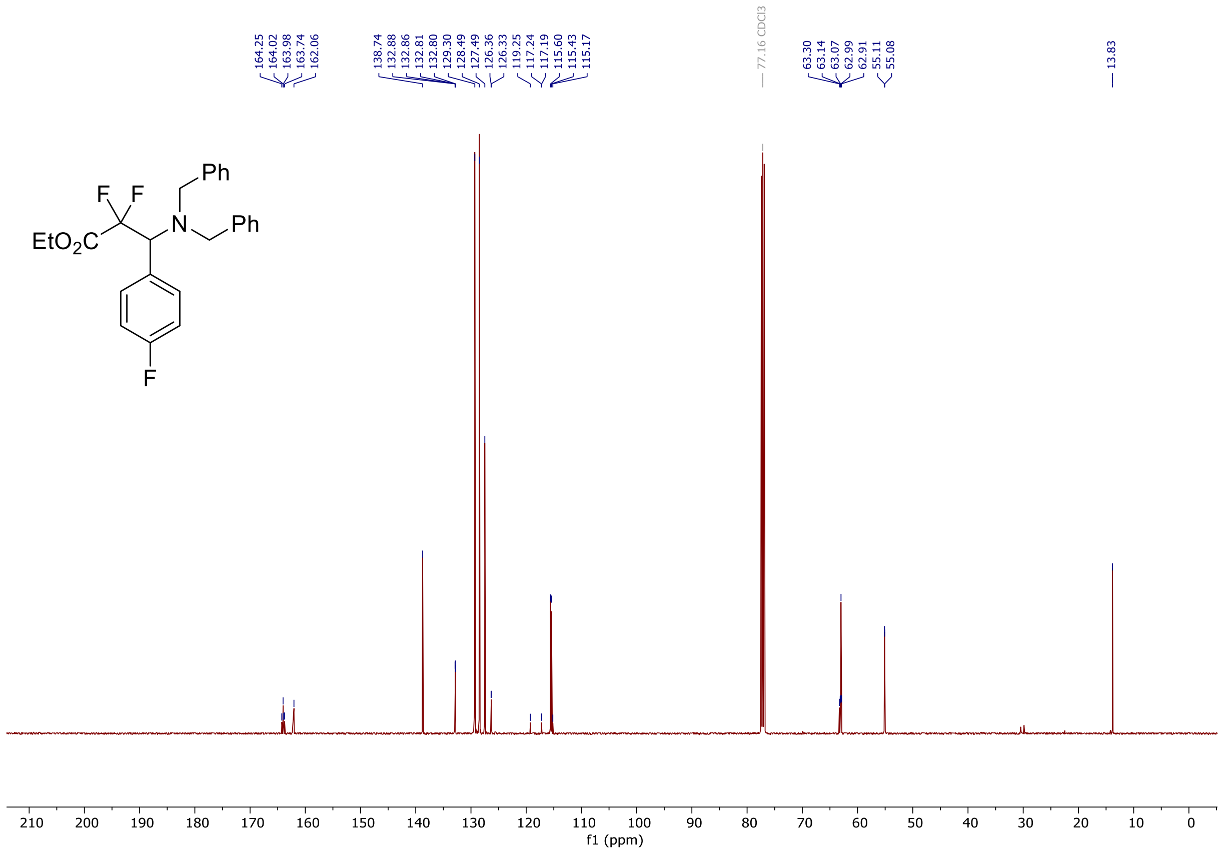

3p – ^{19}F NMR (377 MHz, $CDCl_3$)

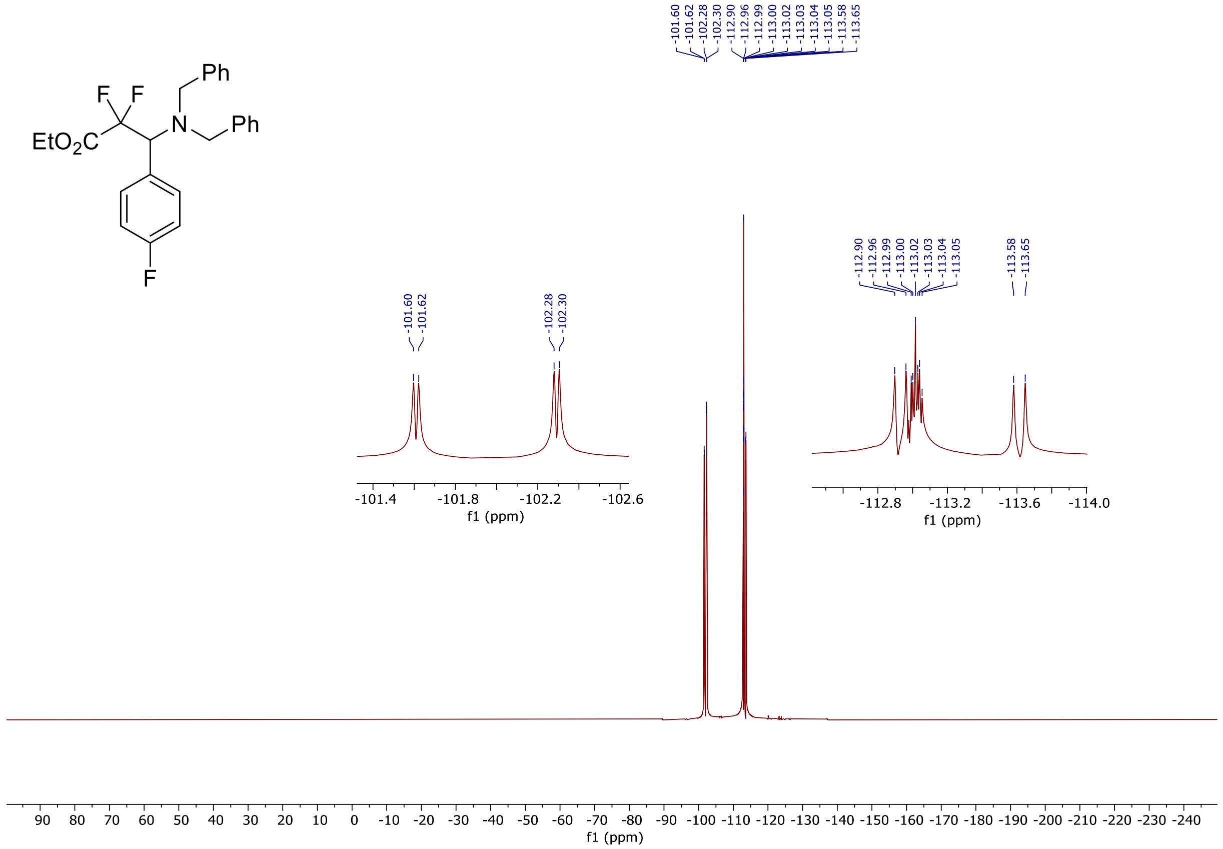

3q – ^{1}H NMR (400 MHz, $CDCl_3$)

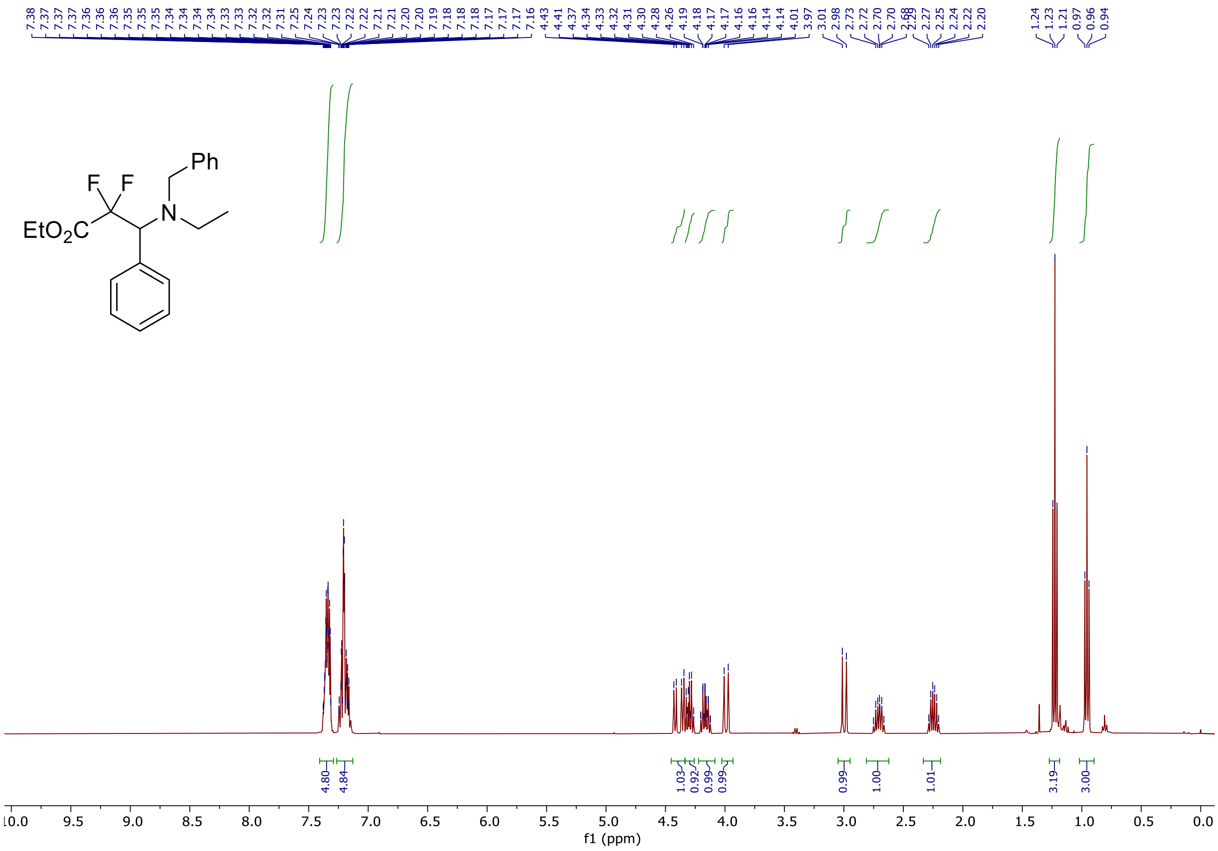

3q – ^{13}C NMR (126 MHz, $CDCl_3$)

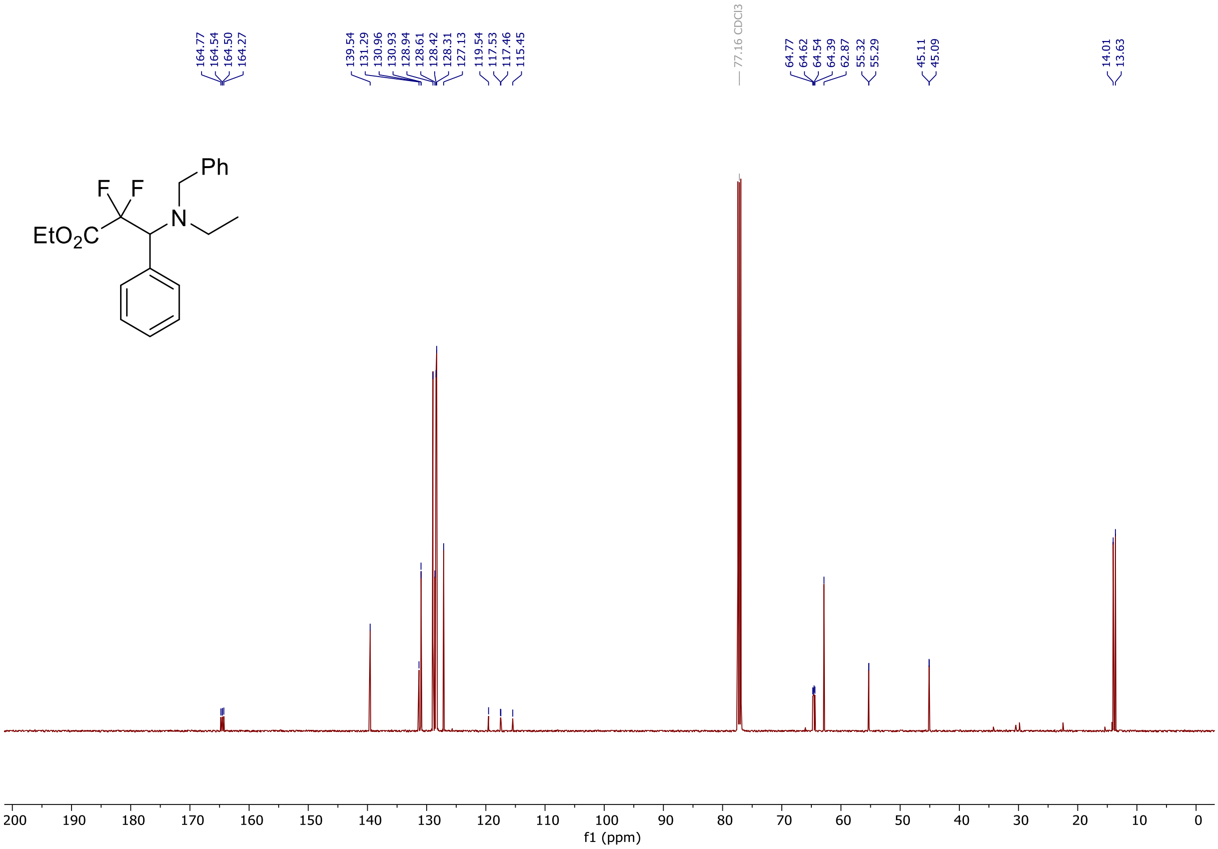

3q – ^{19}F NMR (377 MHz, CDCl$_3$)

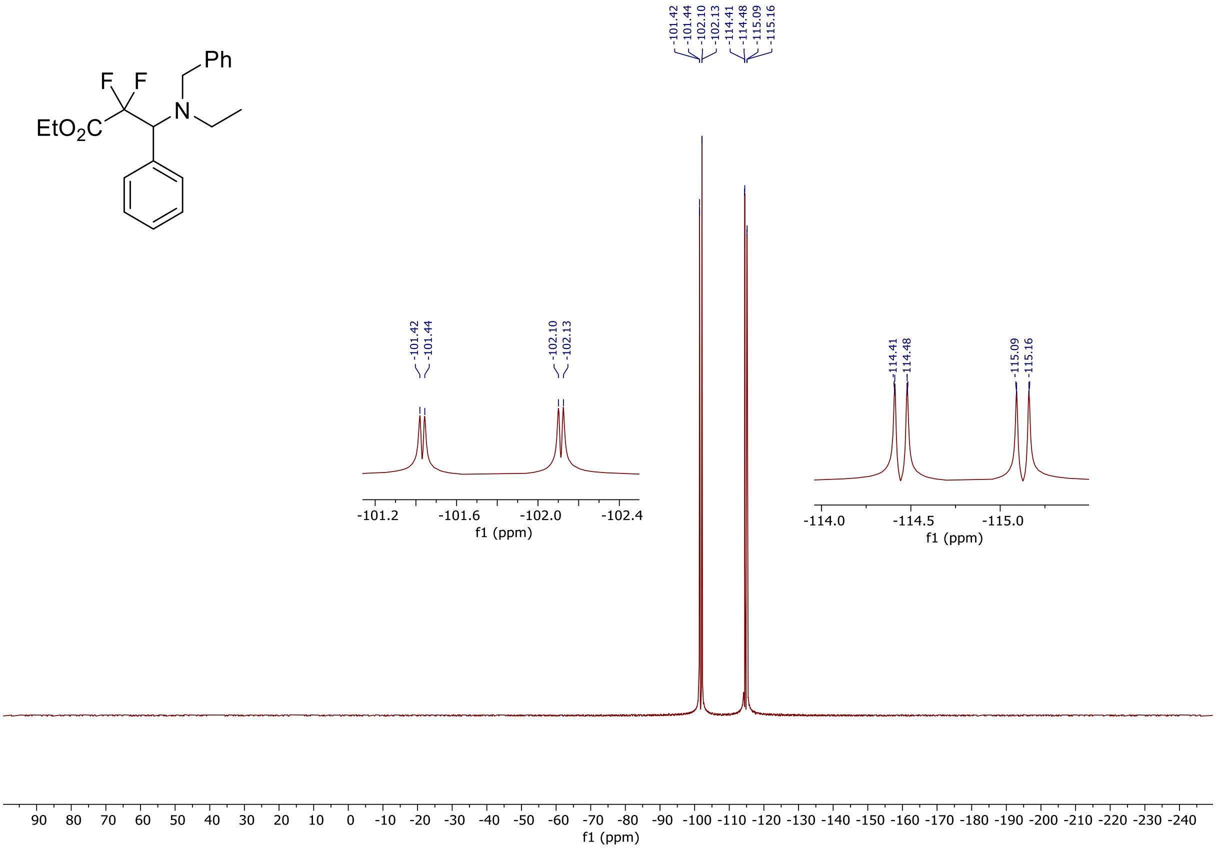

3r – ^{1}H NMR (400 MHz, CDCl$_3$)

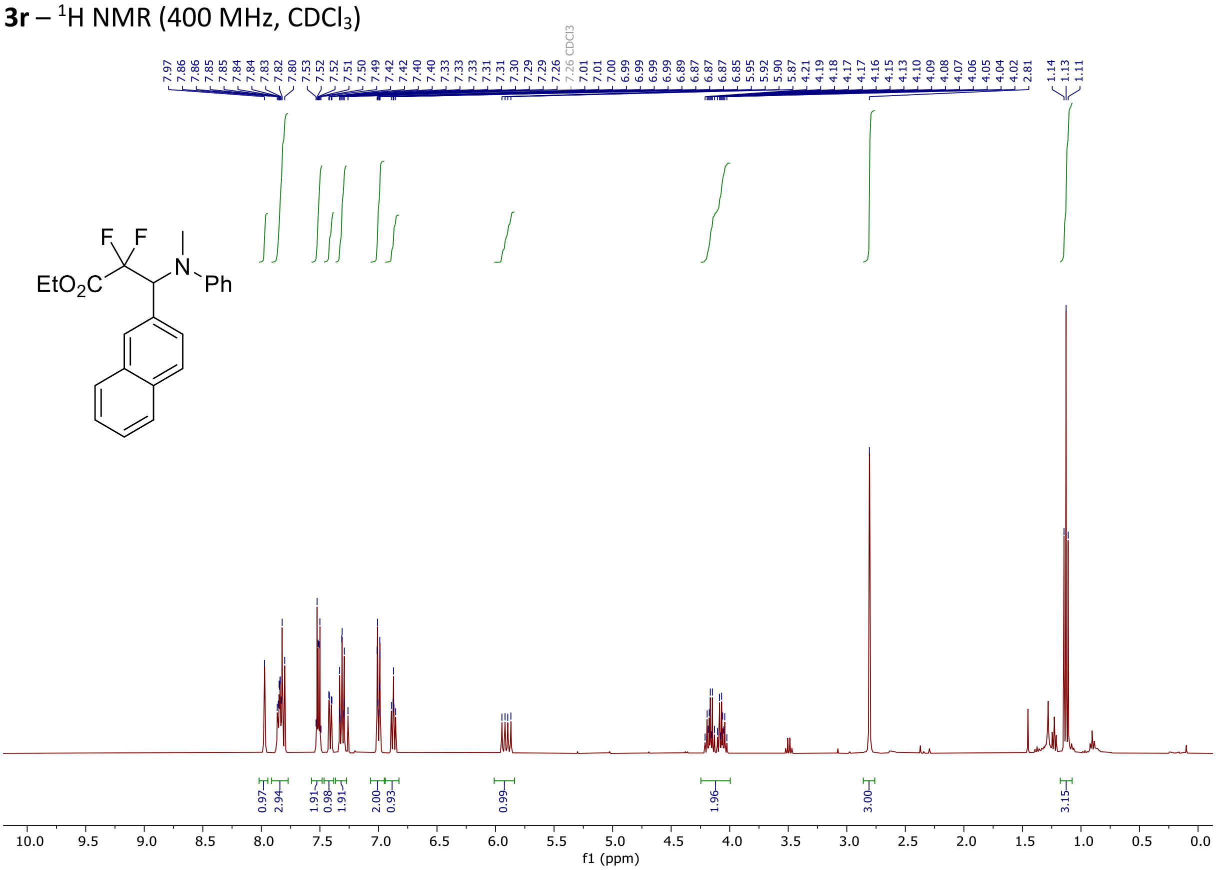

3r – ^{13}C NMR (126 MHz, $CDCl_3$)

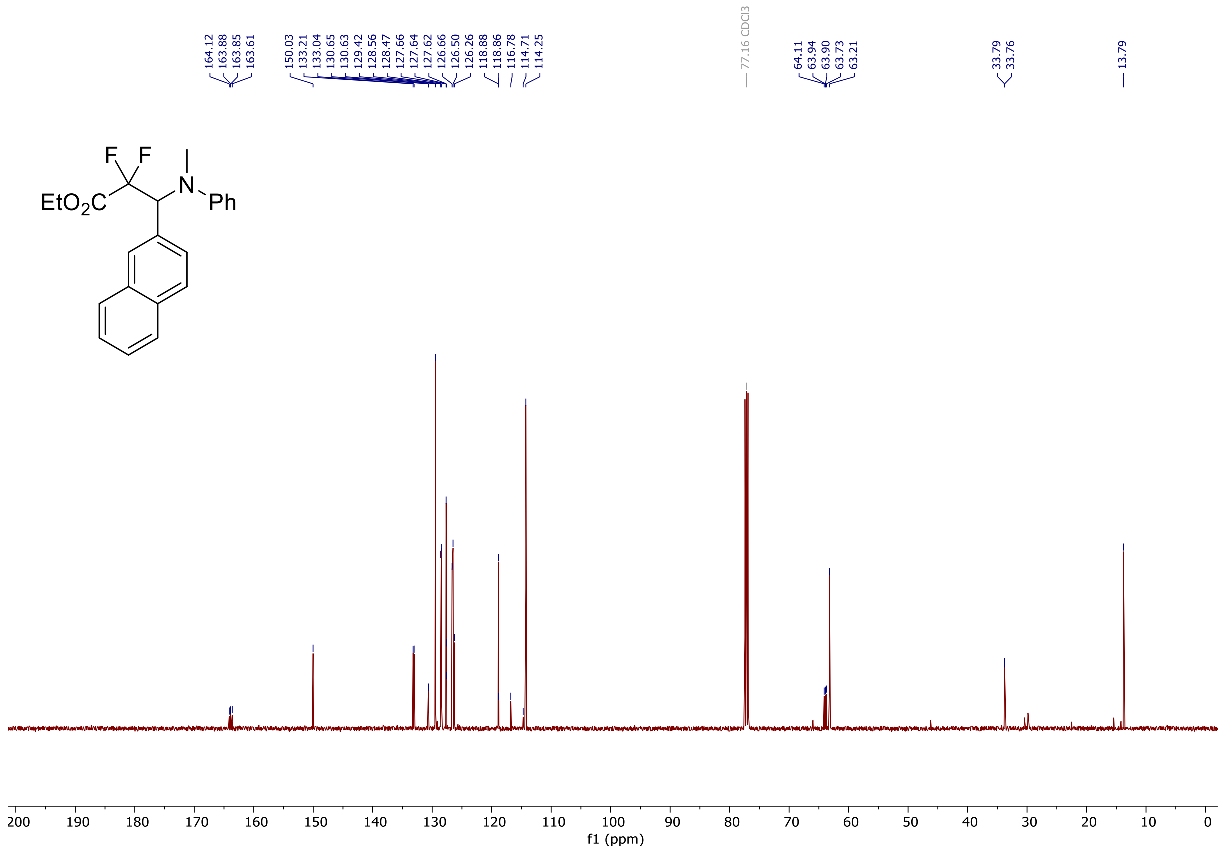

3r – ^{19}F NMR (377 MHz, $CDCl_3$)

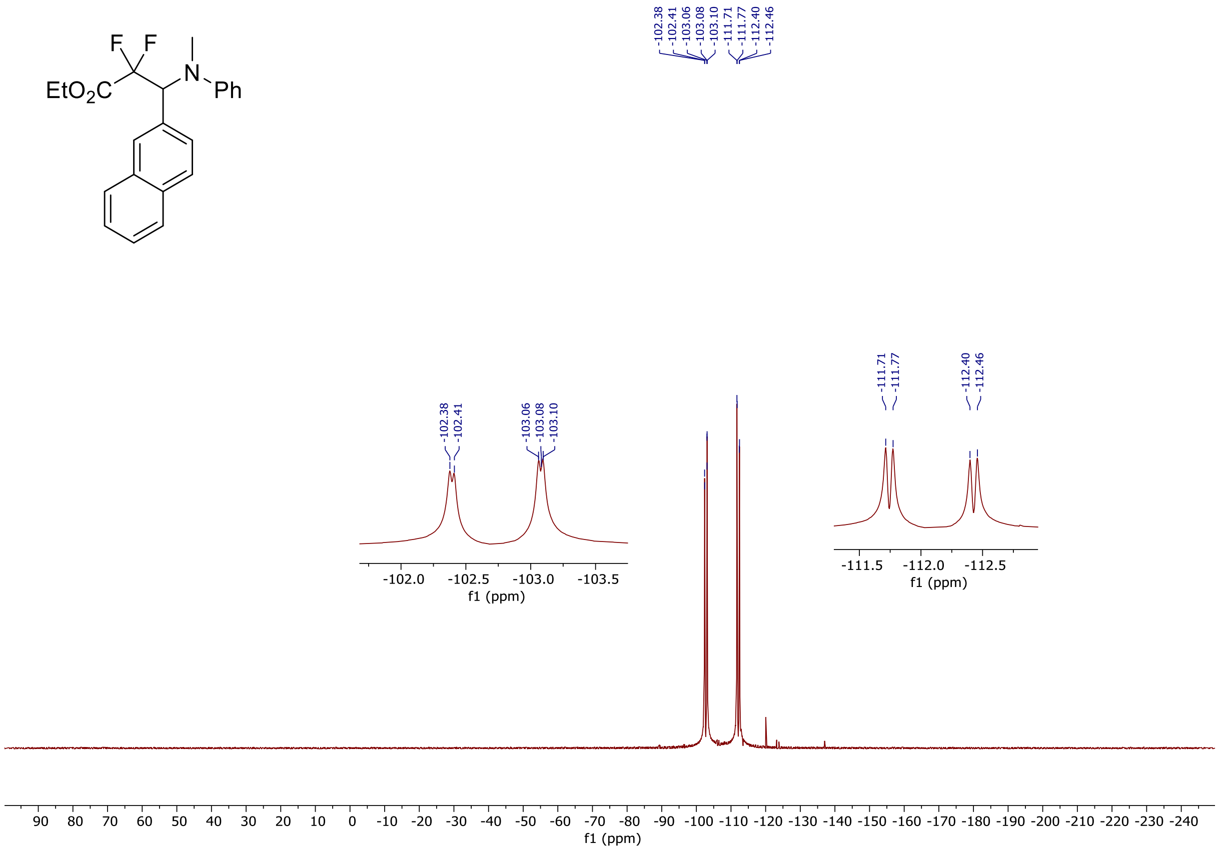

3s – ^{1}H NMR (400 MHz, $CDCl_3$)

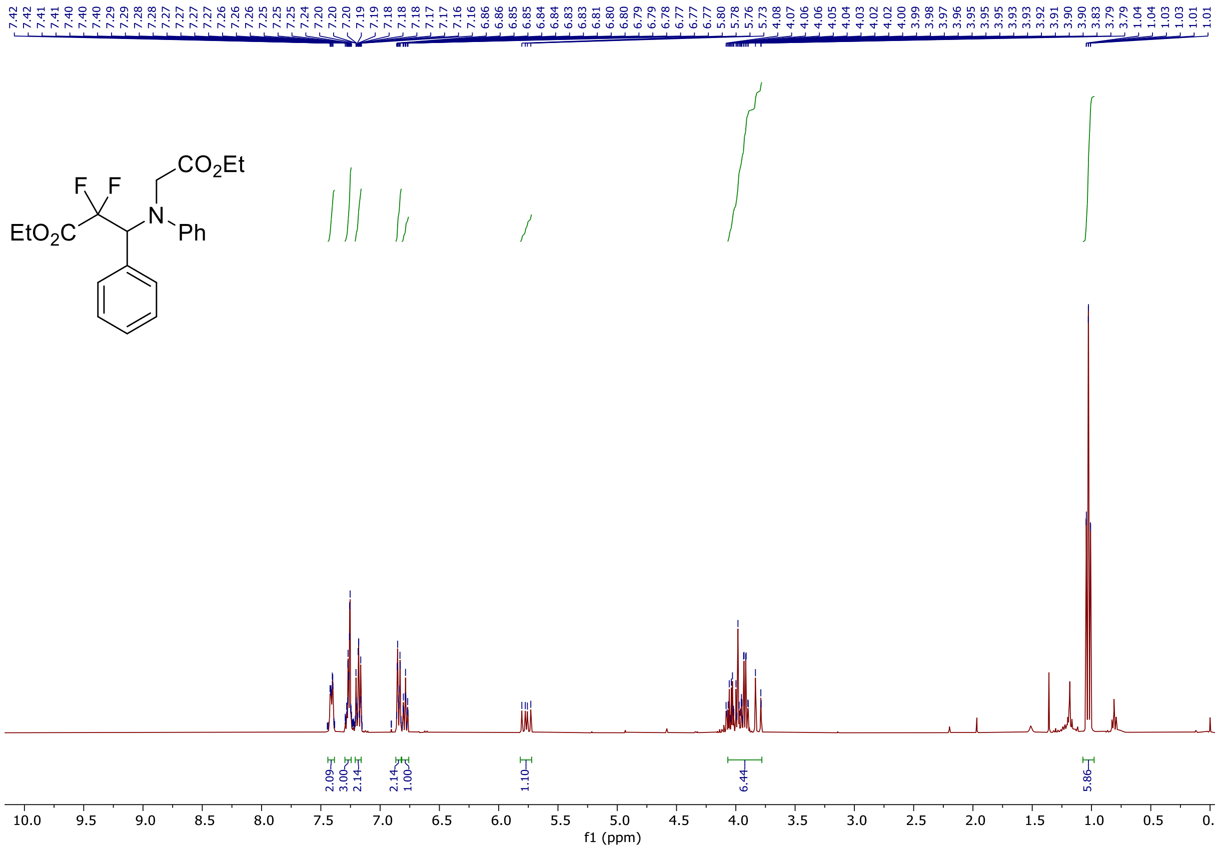

3s – ^{13}C NMR (101 MHz, $CDCl_3$)

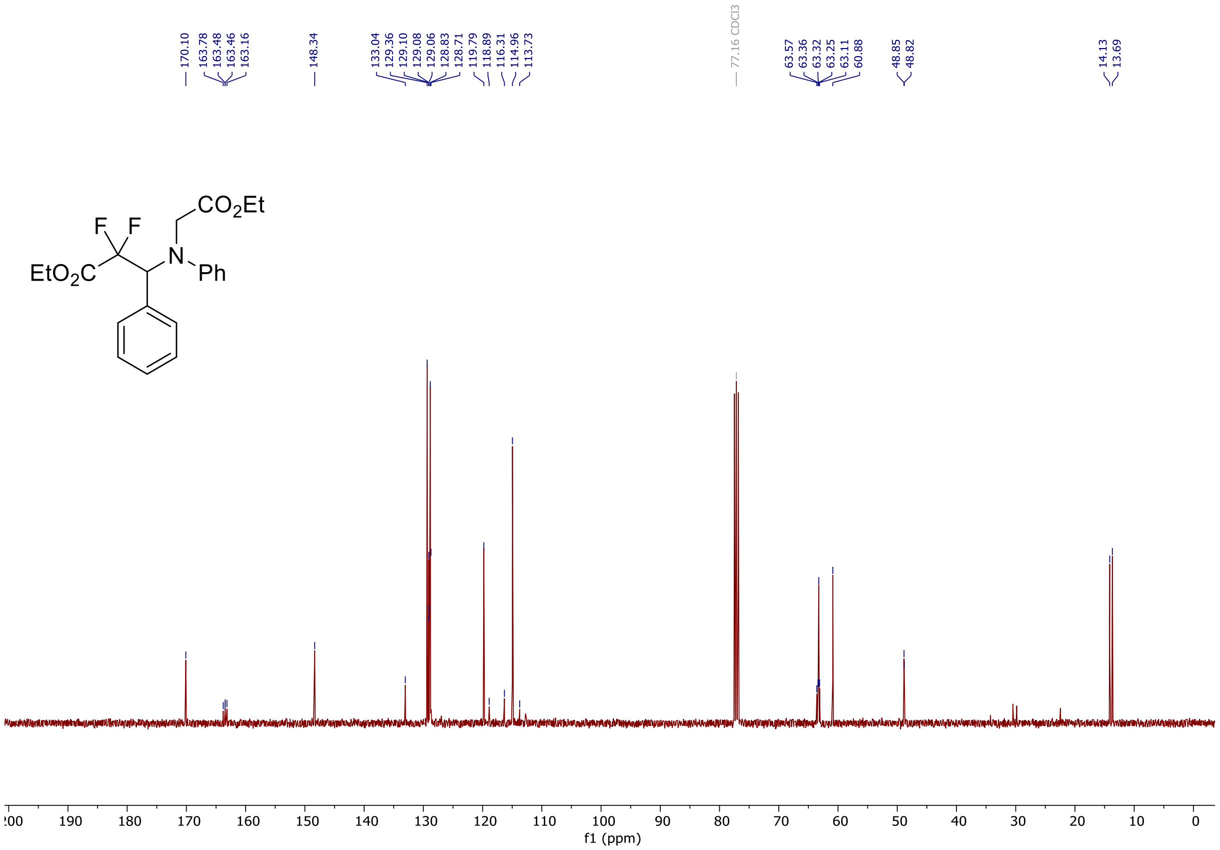

3s – ^{19}F NMR (377 MHz, $CDCl_3$)

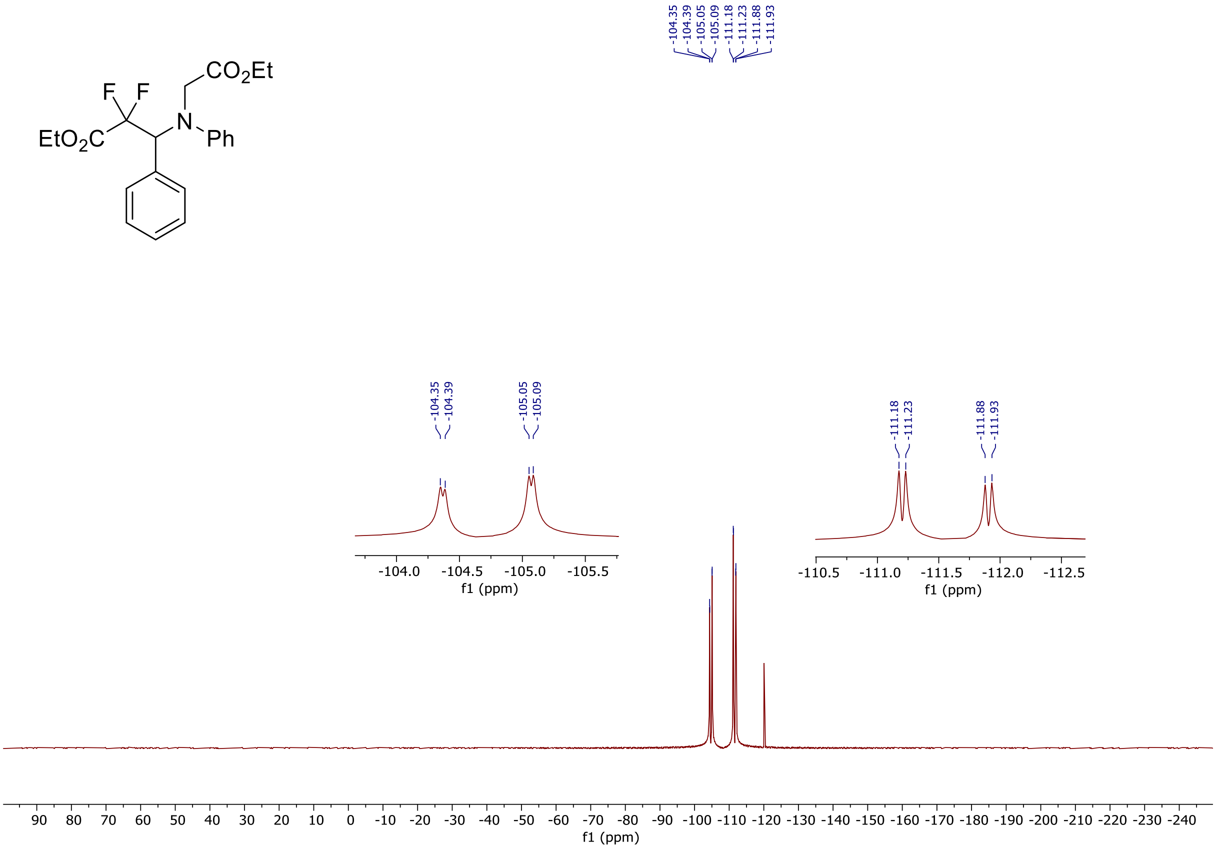

3t – ^{1}H NMR (400 MHz, $CDCl_3$)

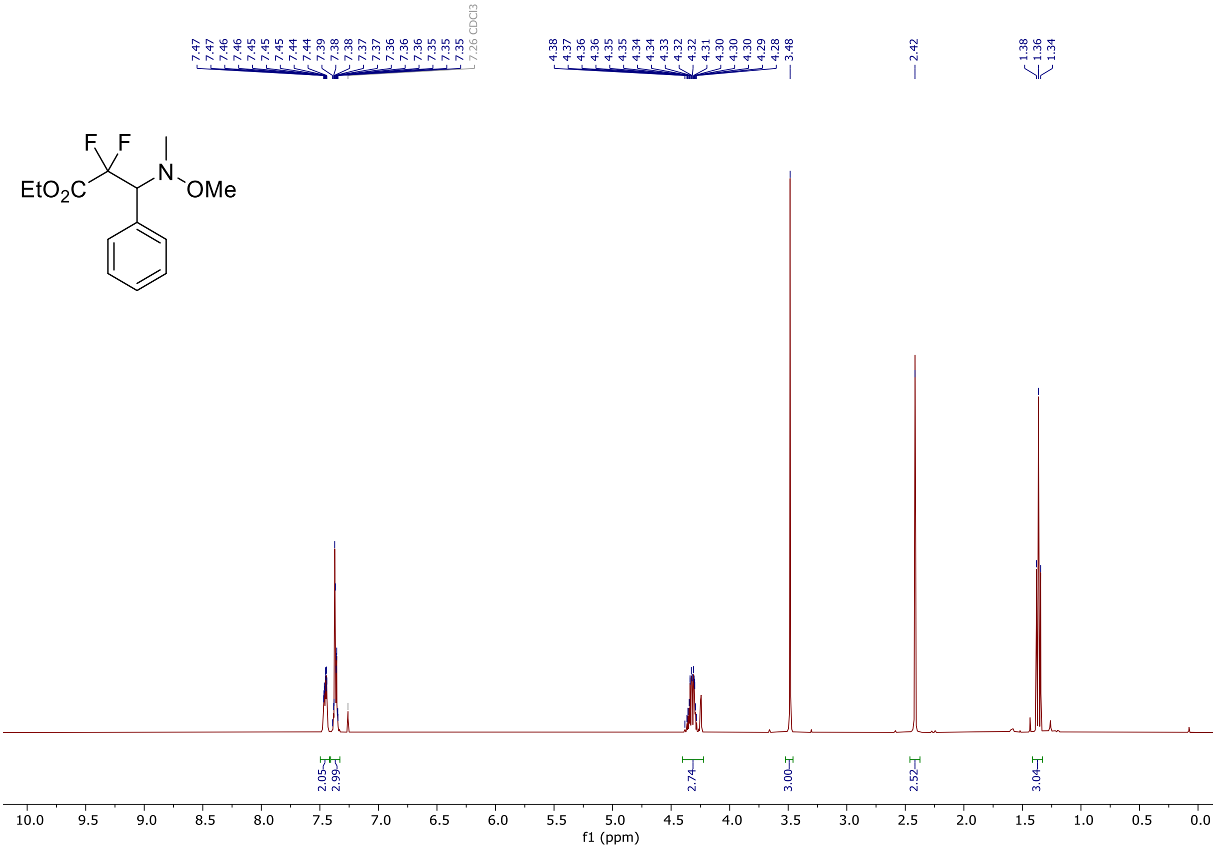

3t – ^{13}C NMR (101 MHz, CDCl$_3$)

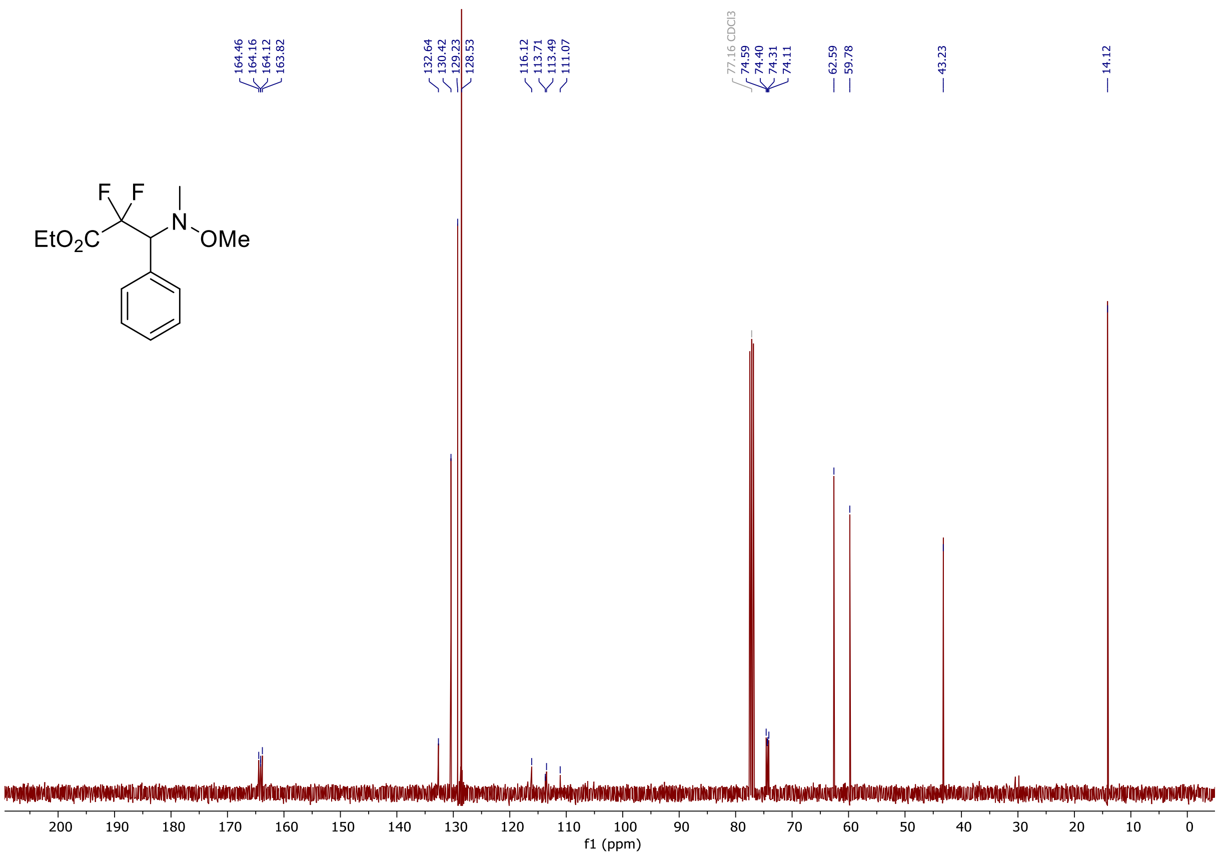

3t – ^{19}F NMR (377 MHz, CDCl$_3$)

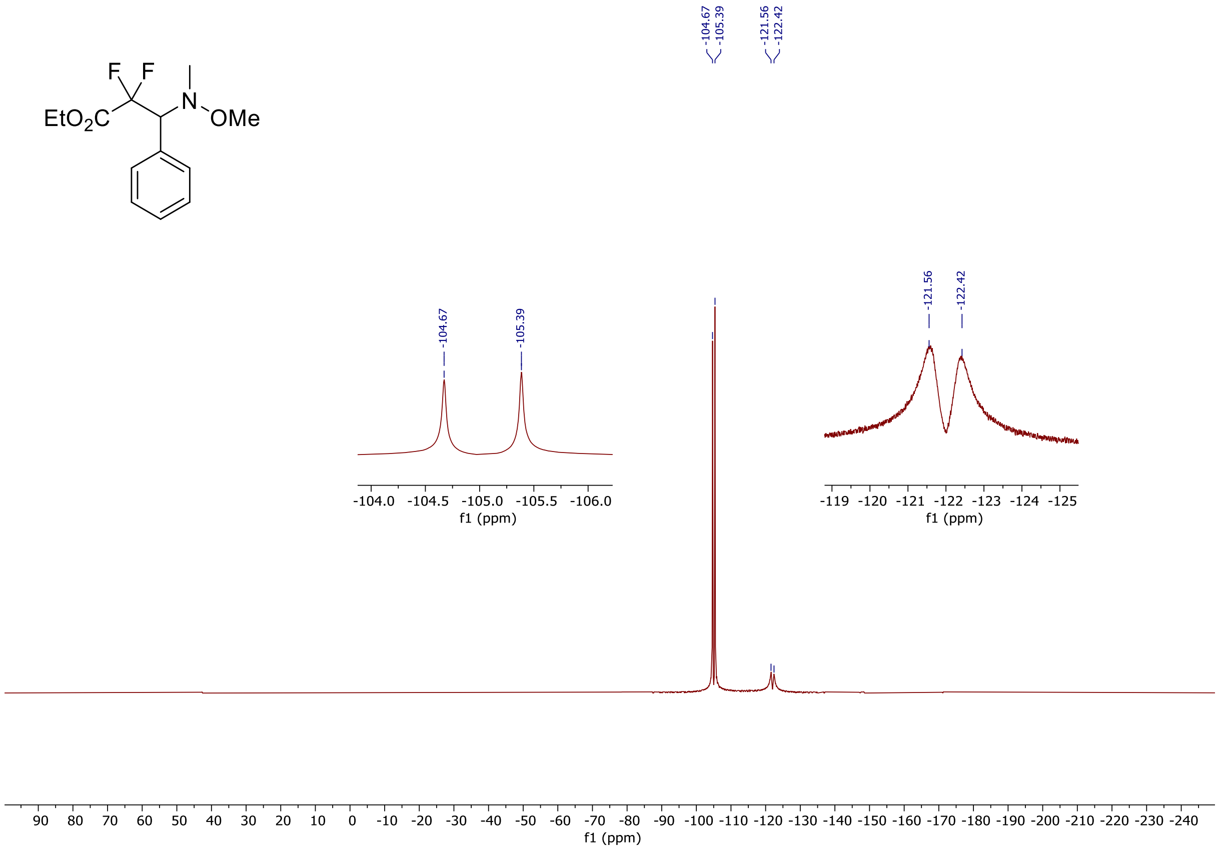

3u – ^{1}H NMR (400 MHz, $CDCl_3$)

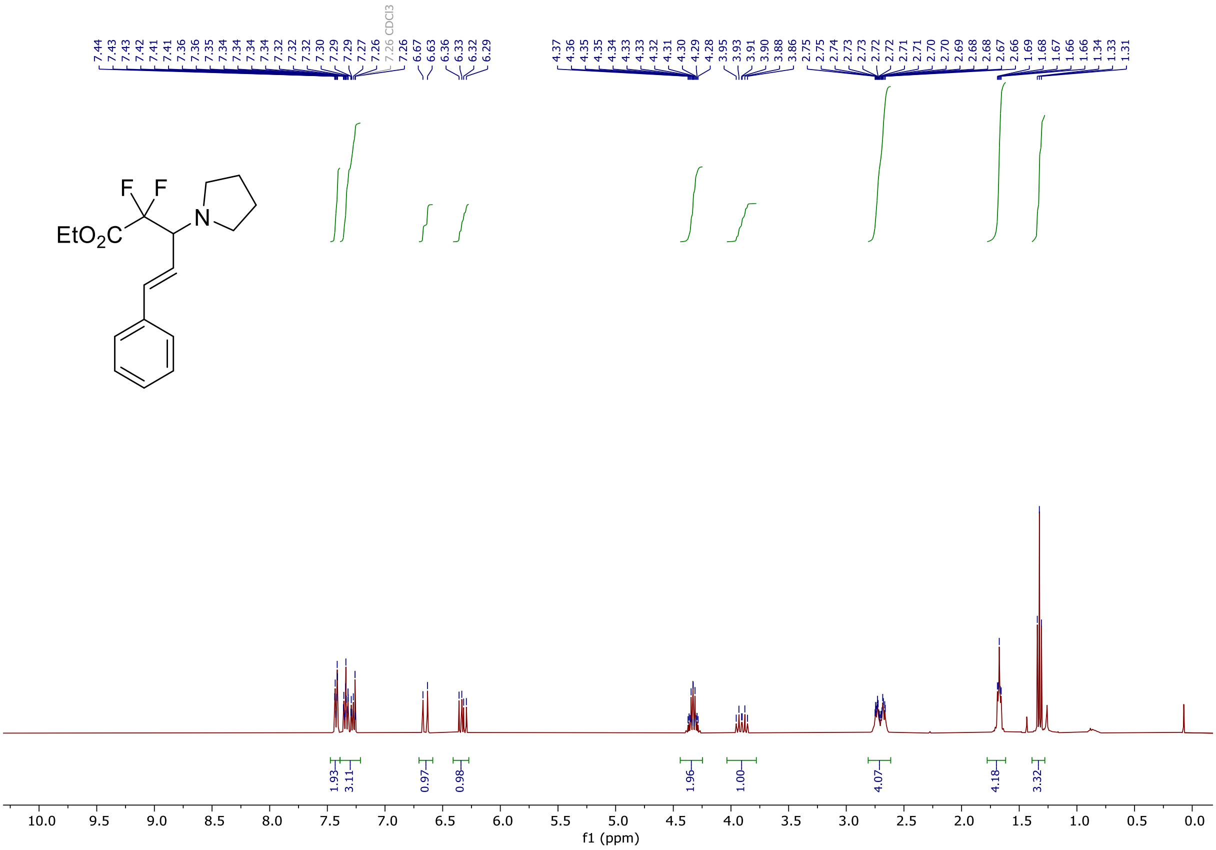

3u – ^{13}C NMR (101 MHz, $CDCl_3$)

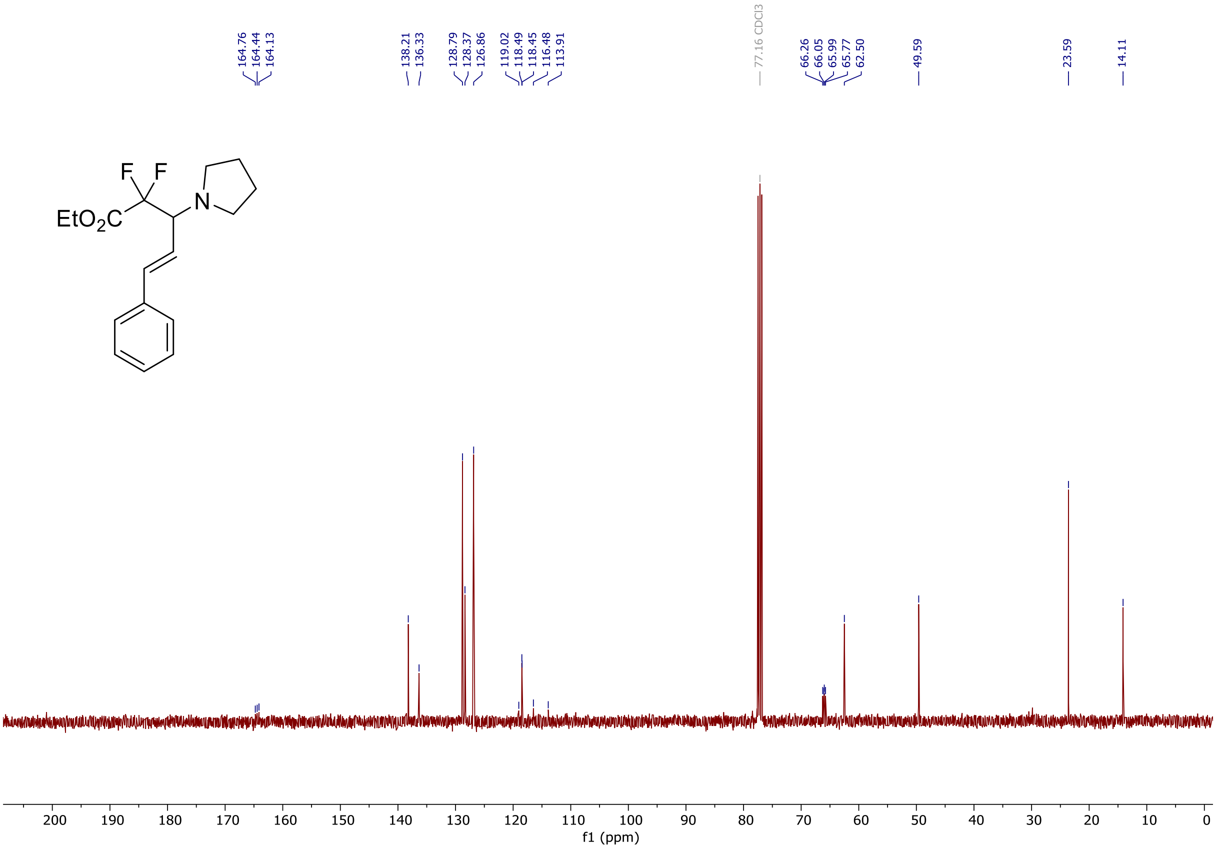

3u – ^{19}F NMR (377 MHz, CDCl$_3$)

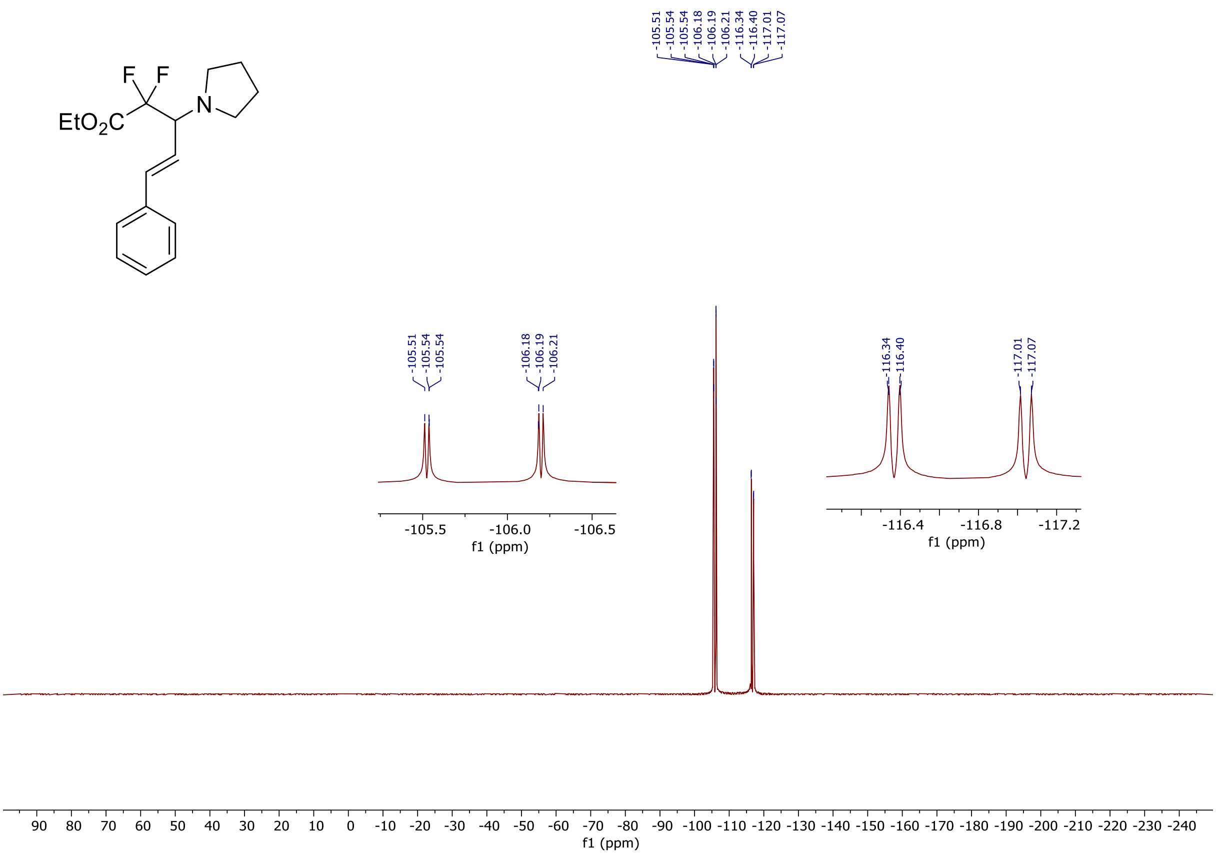

3v – ^{1}H NMR (400 MHz, CDCl$_3$)

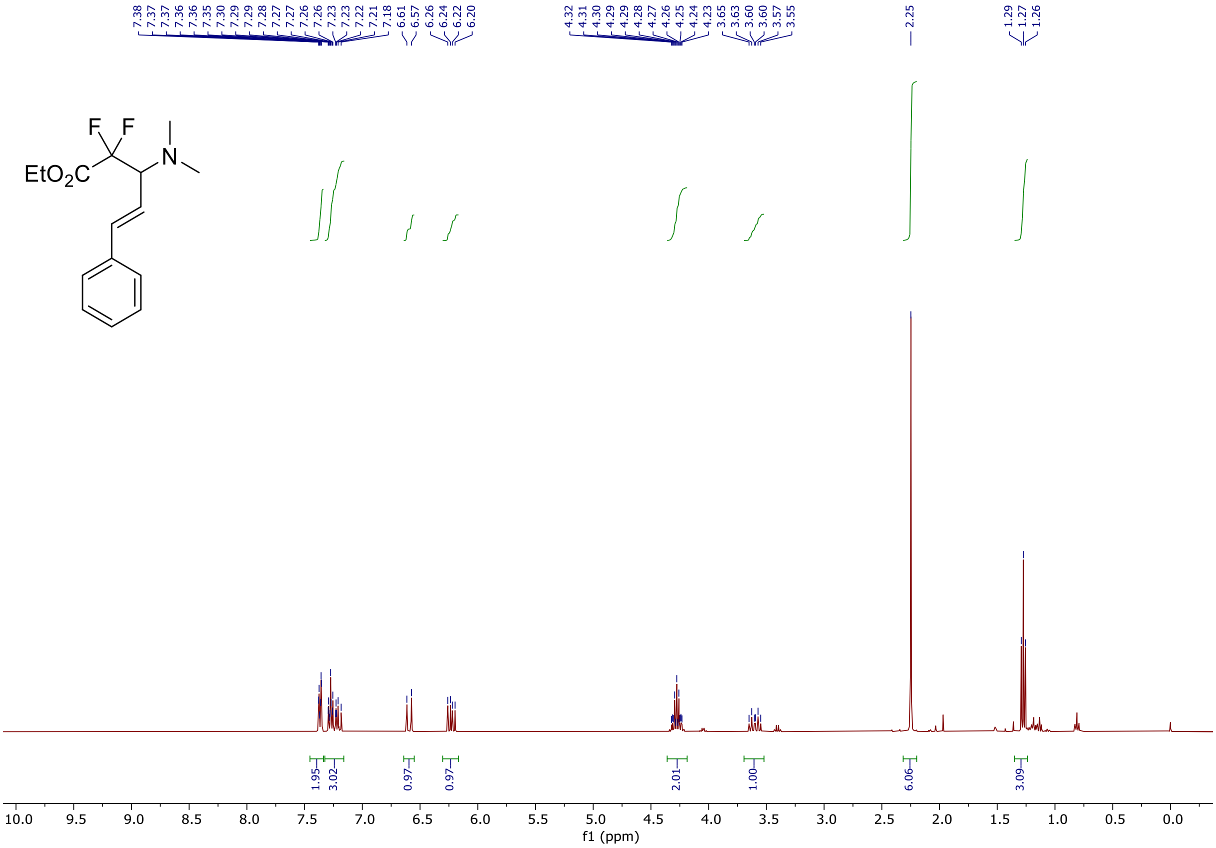

3v – ^{13}C NMR (101 MHz, CDCl$_3$)

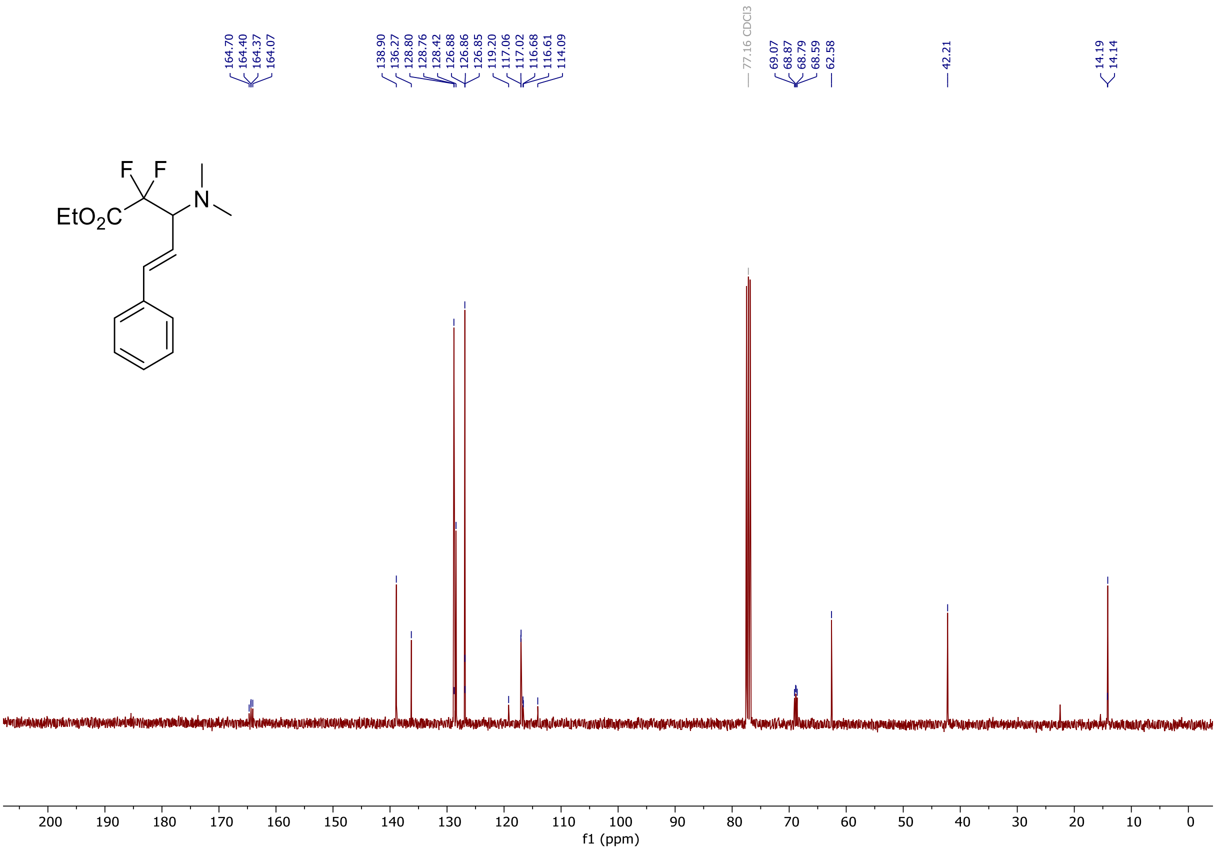

3v – ^{19}F NMR (377 MHz, CDCl$_3$)

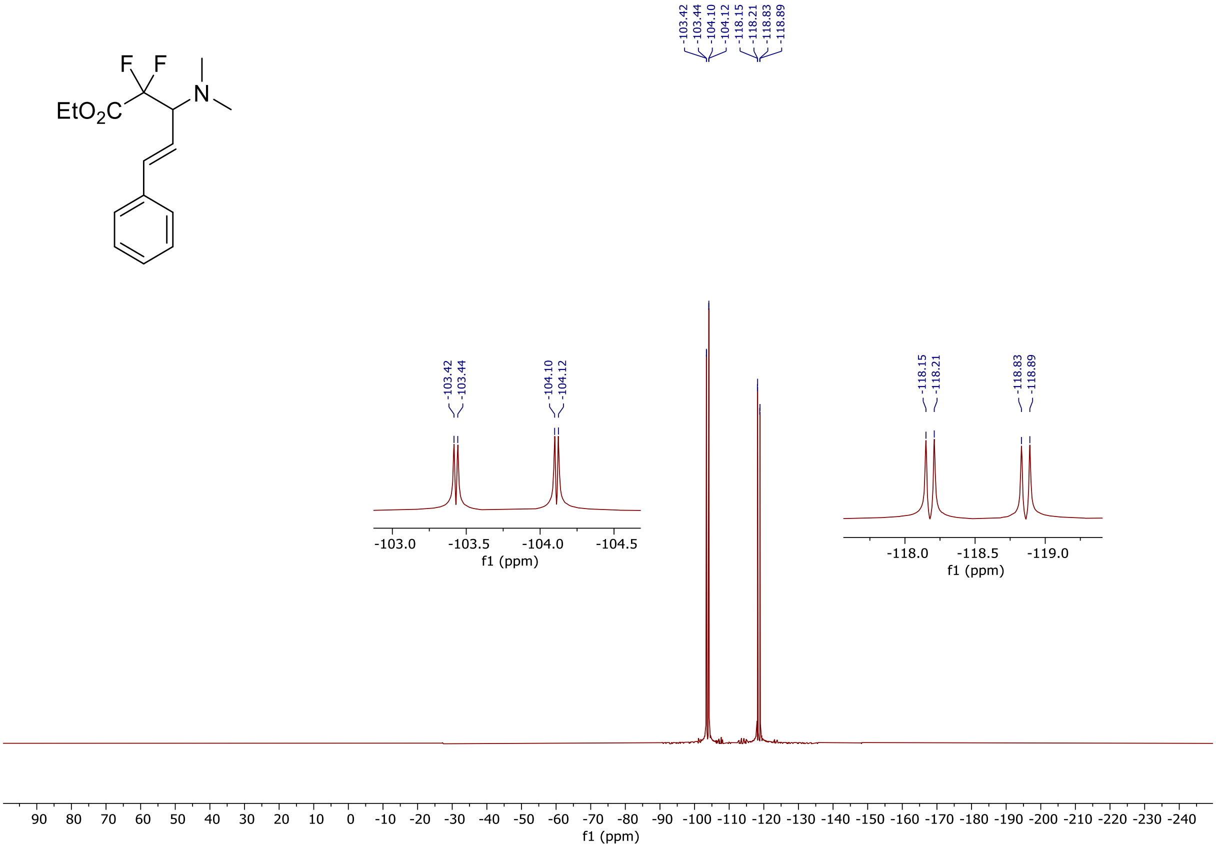

3w – ^{1}H NMR (400 MHz, $CDCl_3$)

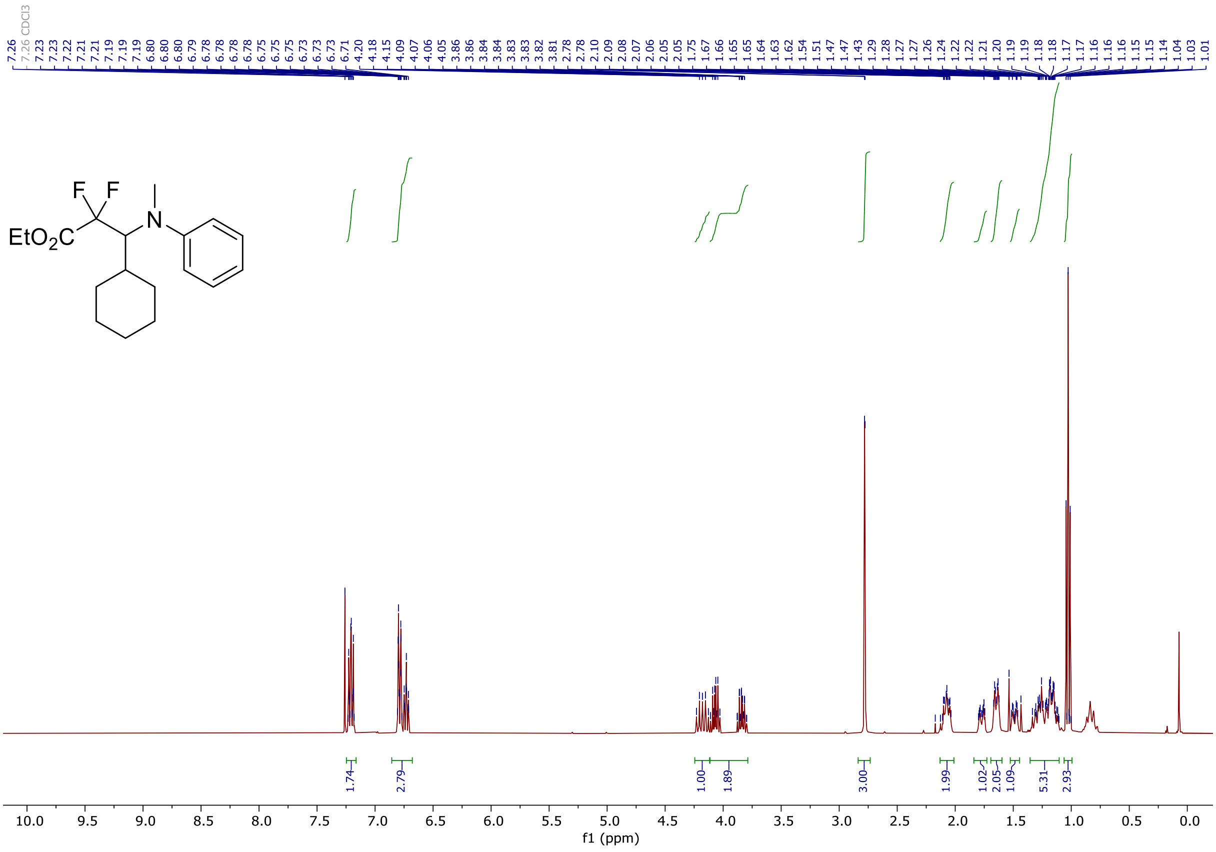

3w – ^{13}C NMR (126 MHz, $CDCl_3$)

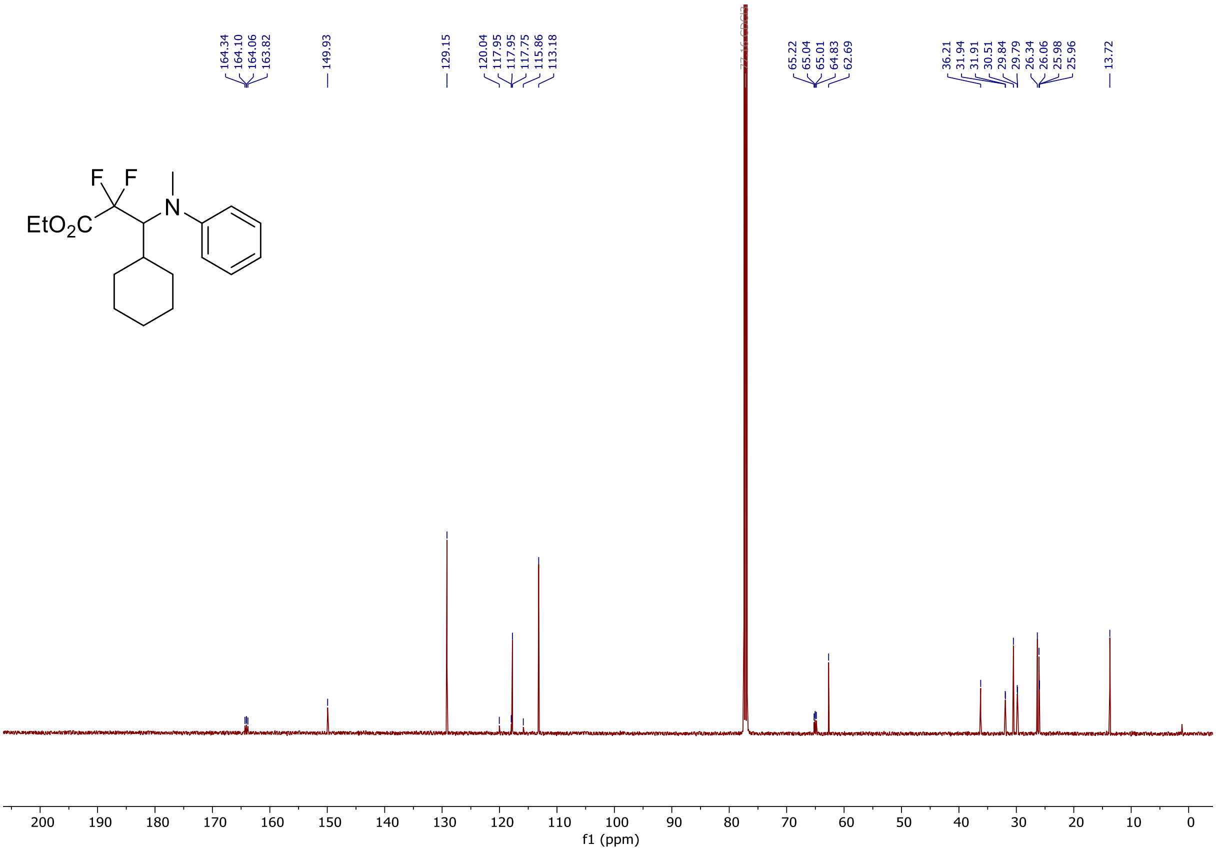

3w – ^{19}F NMR (377 MHz, $CDCl_3$)

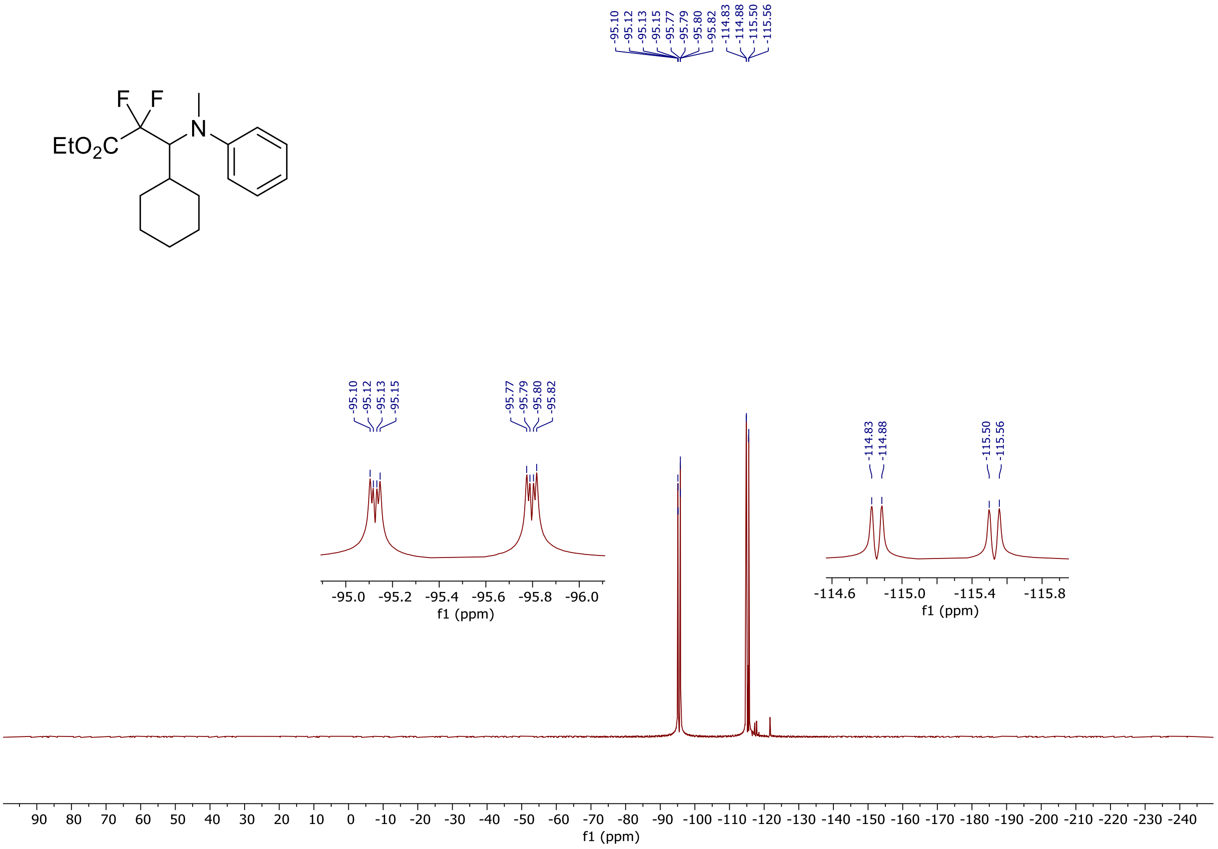

3x – ^{1}H NMR (400 MHz, $CDCl_3$)

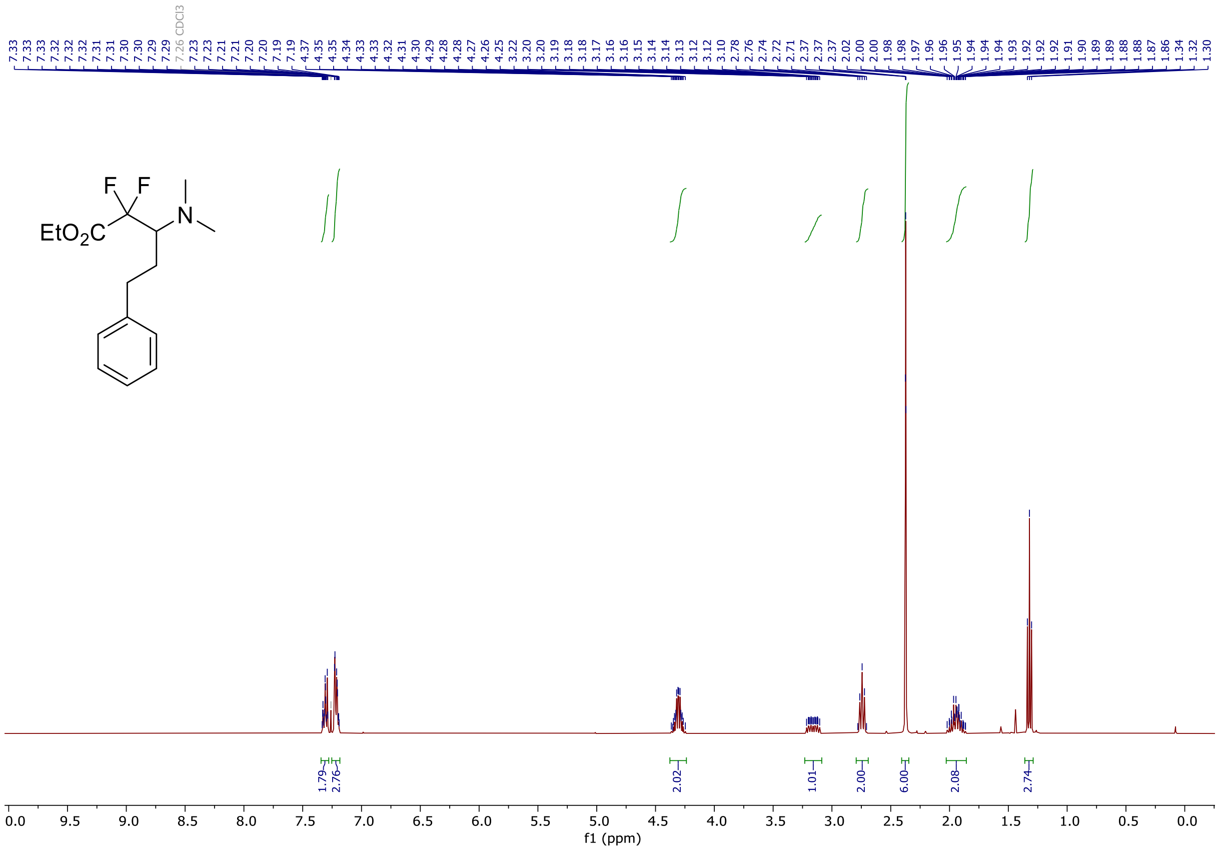

3x – ^{13}C NMR (126 MHz, $CDCl_3$)

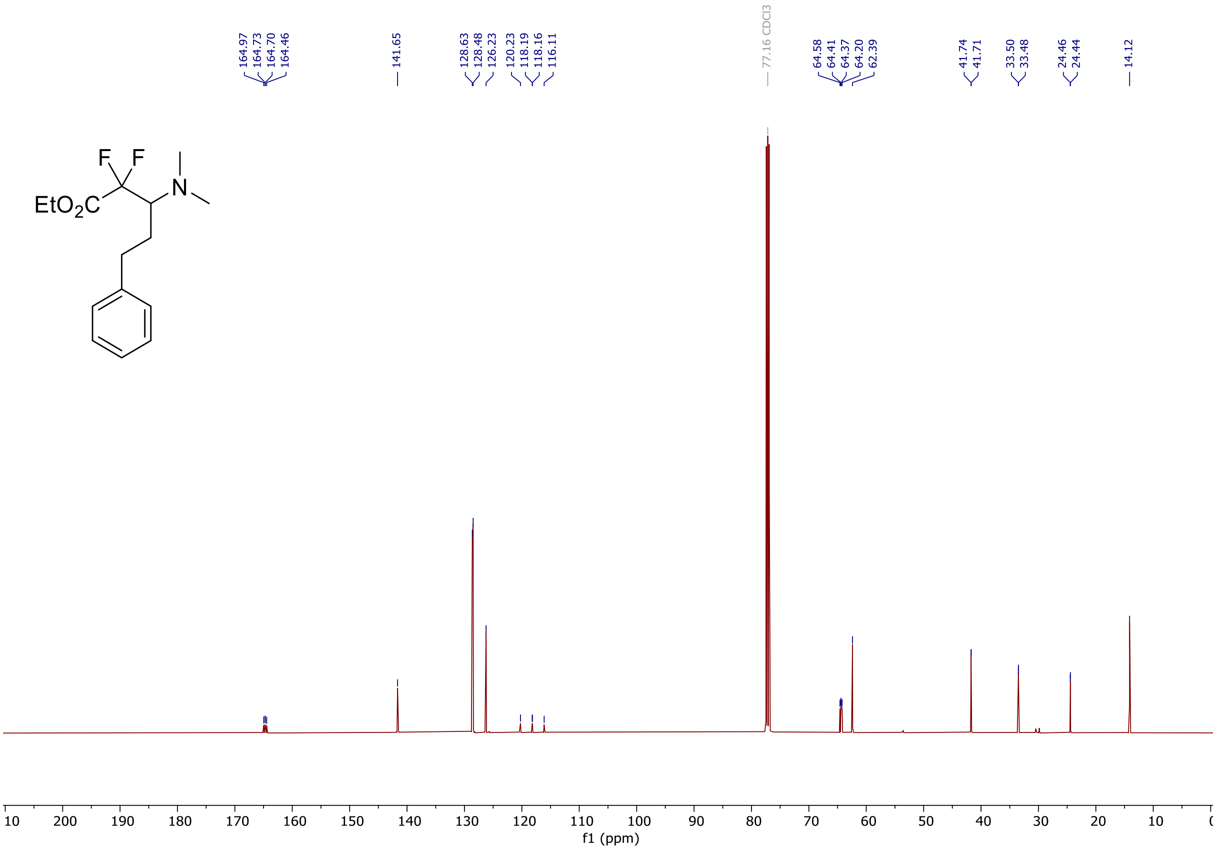

3x – ^{19}F NMR (377 MHz, $CDCl_3$)

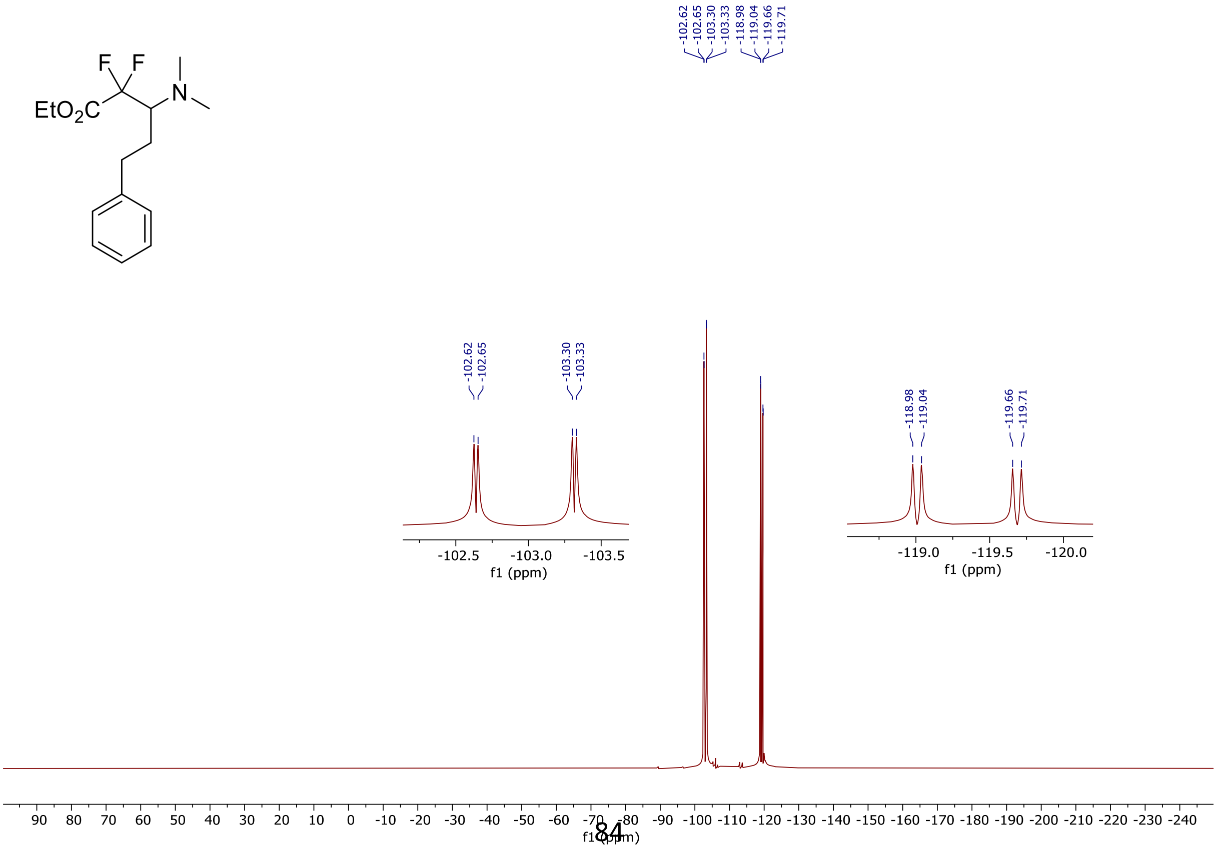

3y – ^{1}H NMR (400 MHz, $CDCl_3$)

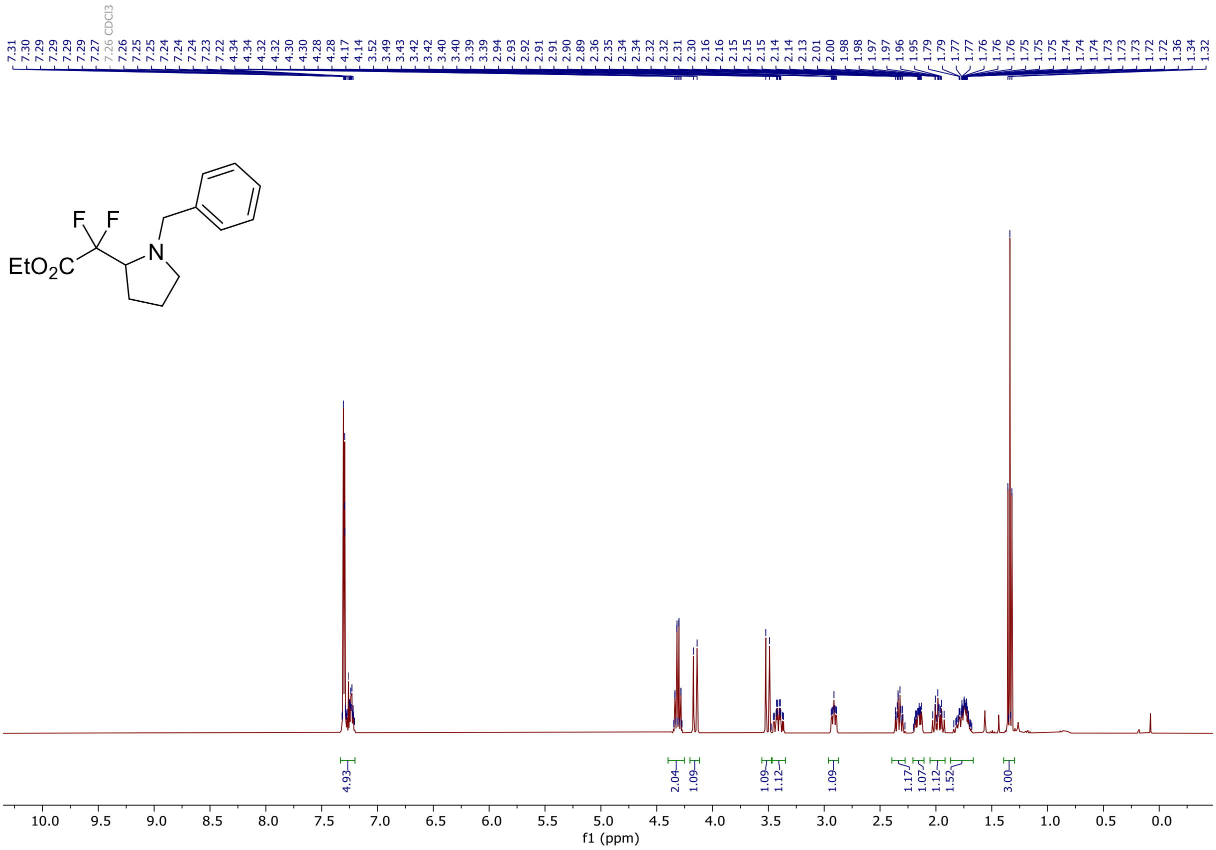

3y – ^{13}C NMR (101 MHz, $CDCl_3$)

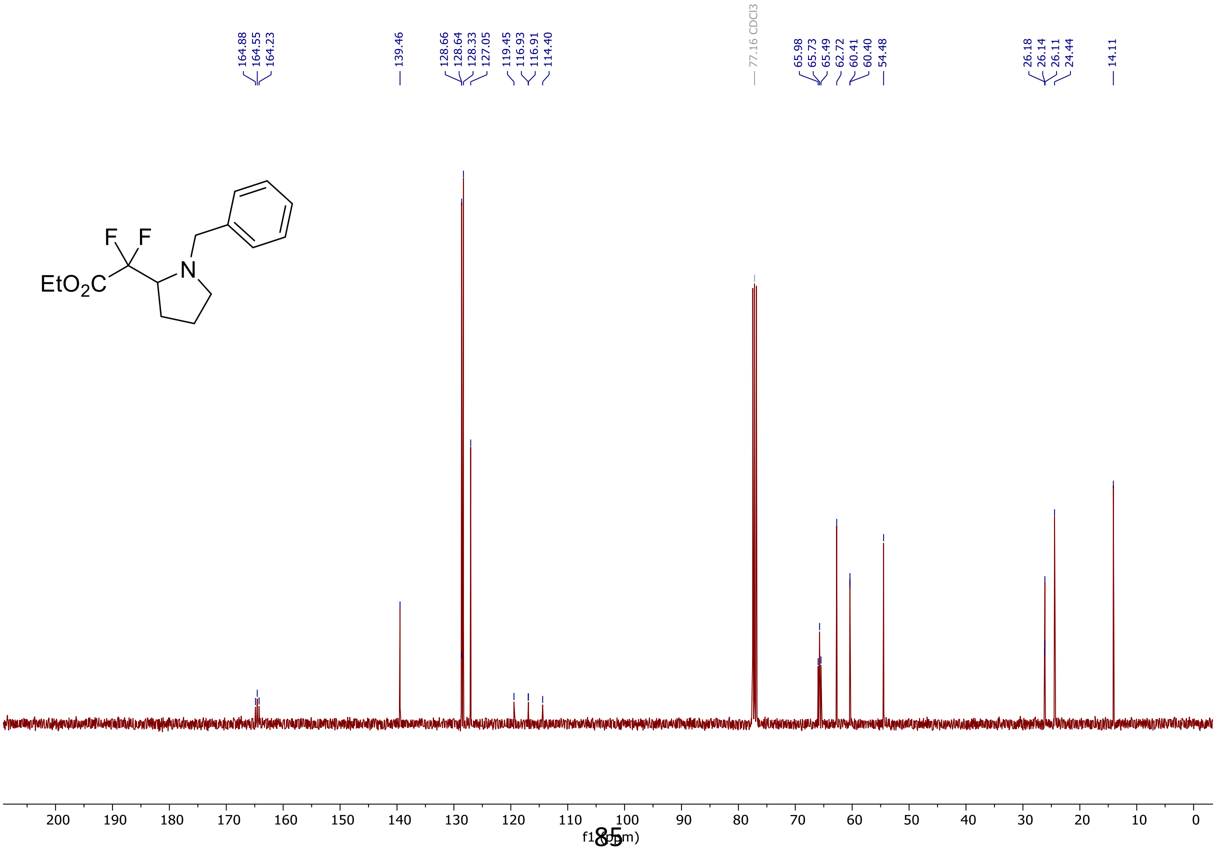

3y – ^{19}F NMR (377 MHz, $CDCl_3$)

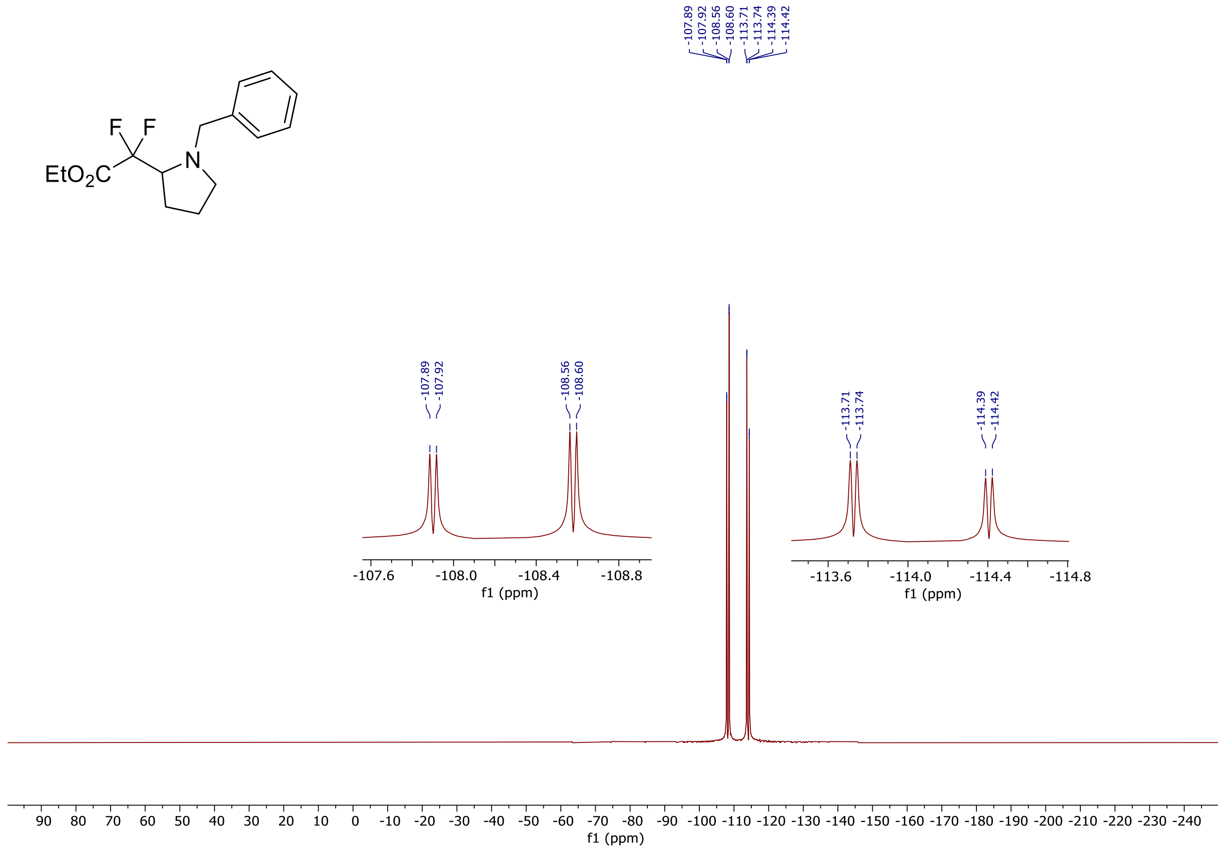

3z – ^{1}H NMR (400 MHz, $CDCl_3$)

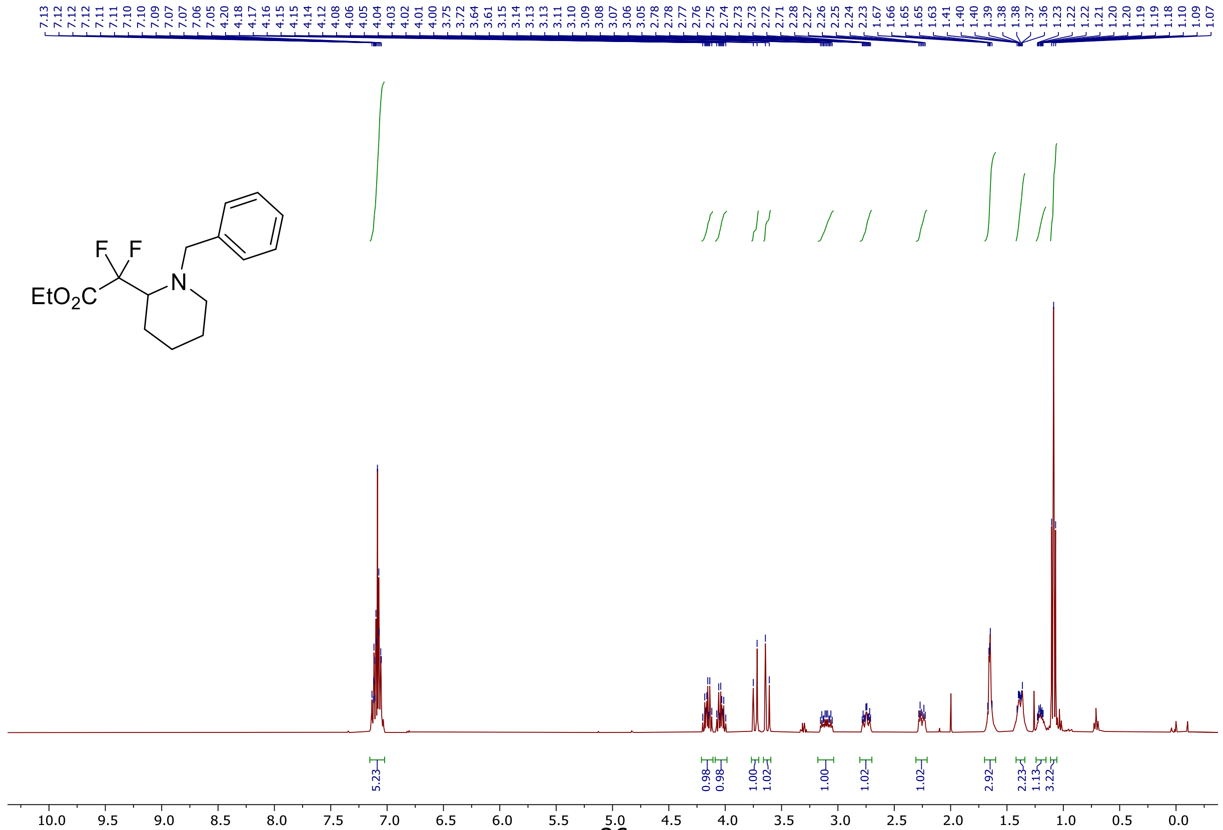

3z – ^{13}C NMR (101 MHz, $CDCl_3$)

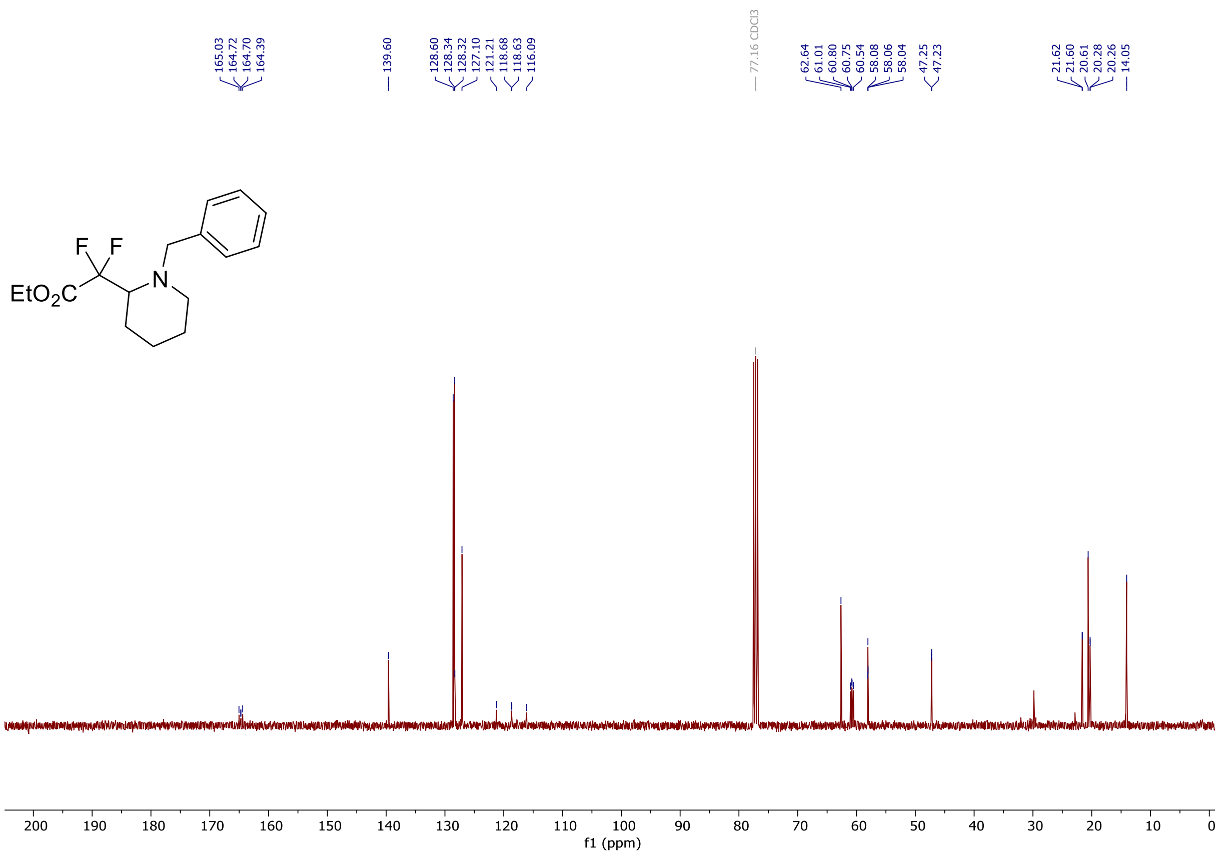

3z – ^{19}F NMR (377 MHz, $CDCl_3$)

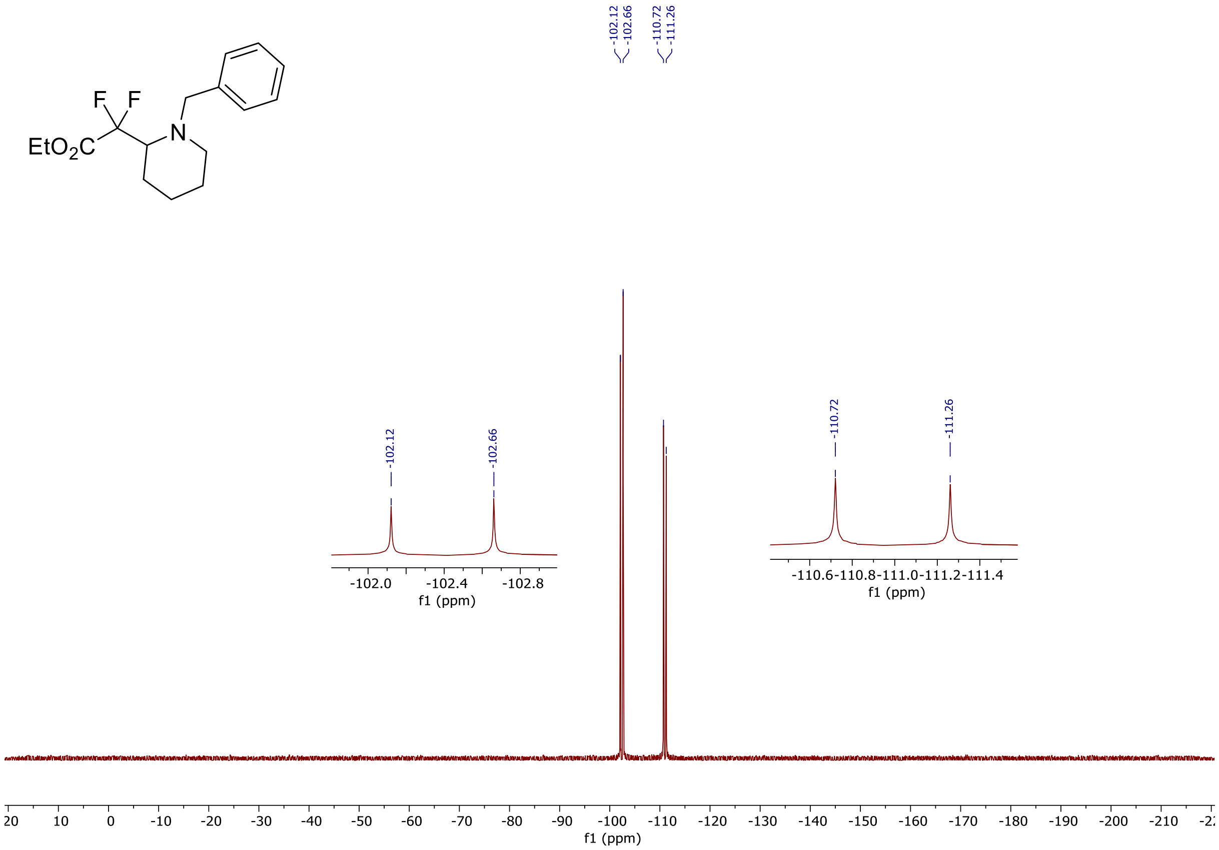

3aa – ^{1}H NMR (400 MHz, $CDCl_3$)

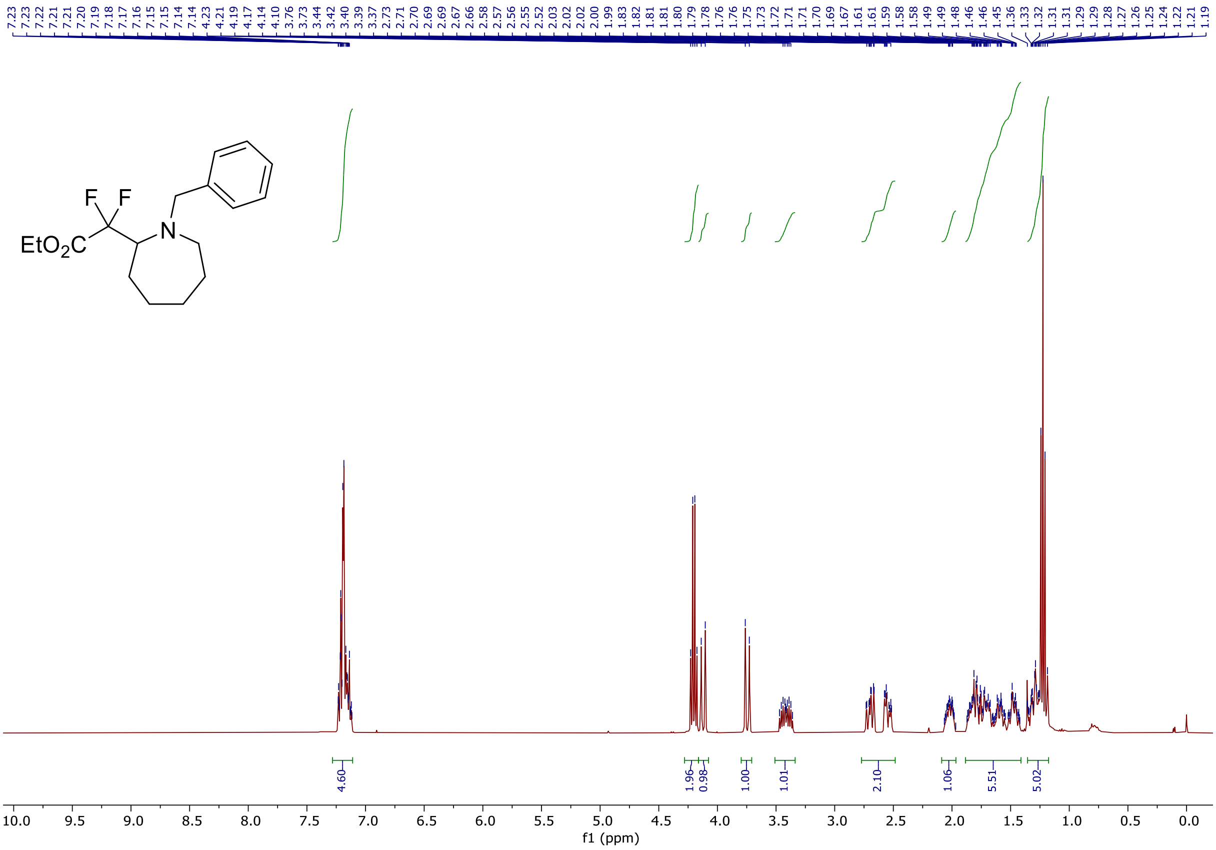

3aa – ^{13}C NMR (101 MHz, $CDCl_3$)

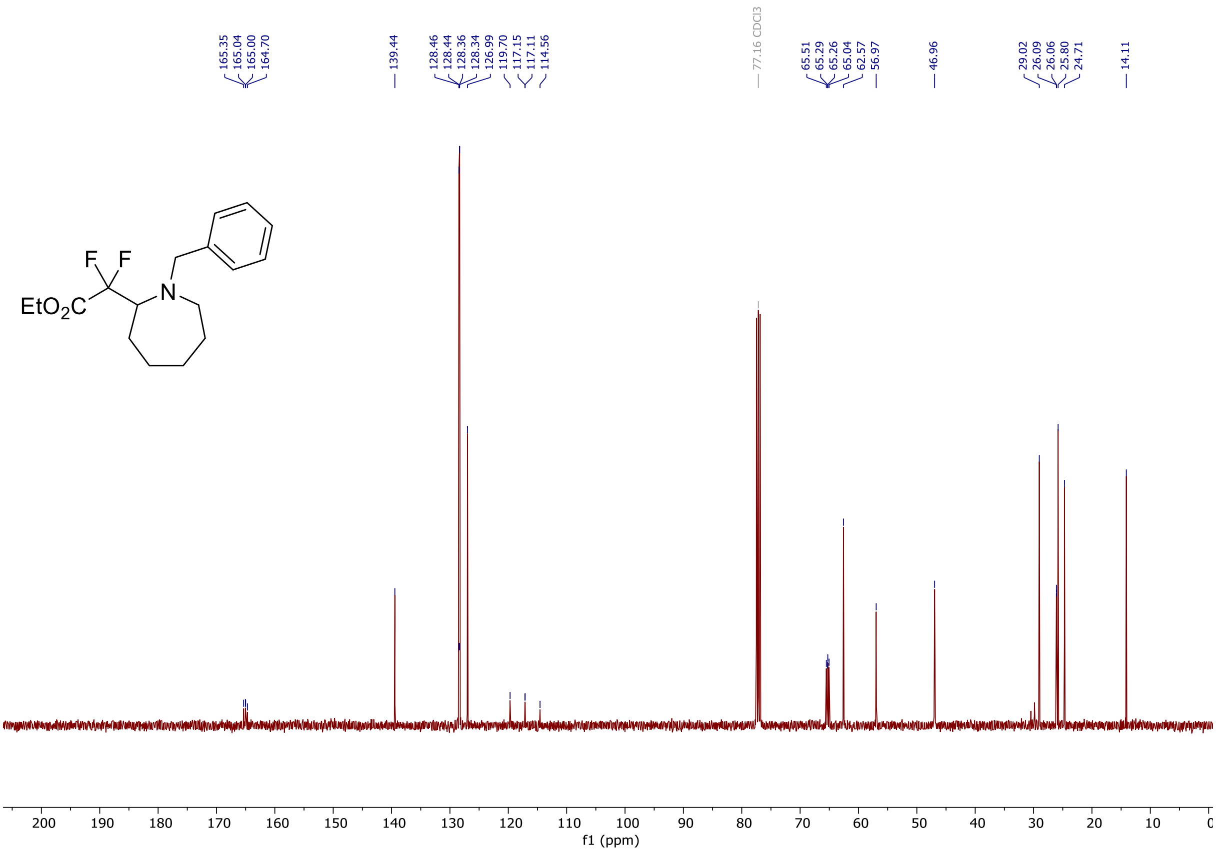

3aa – ^{19}F NMR (377 MHz, $CDCl_3$)

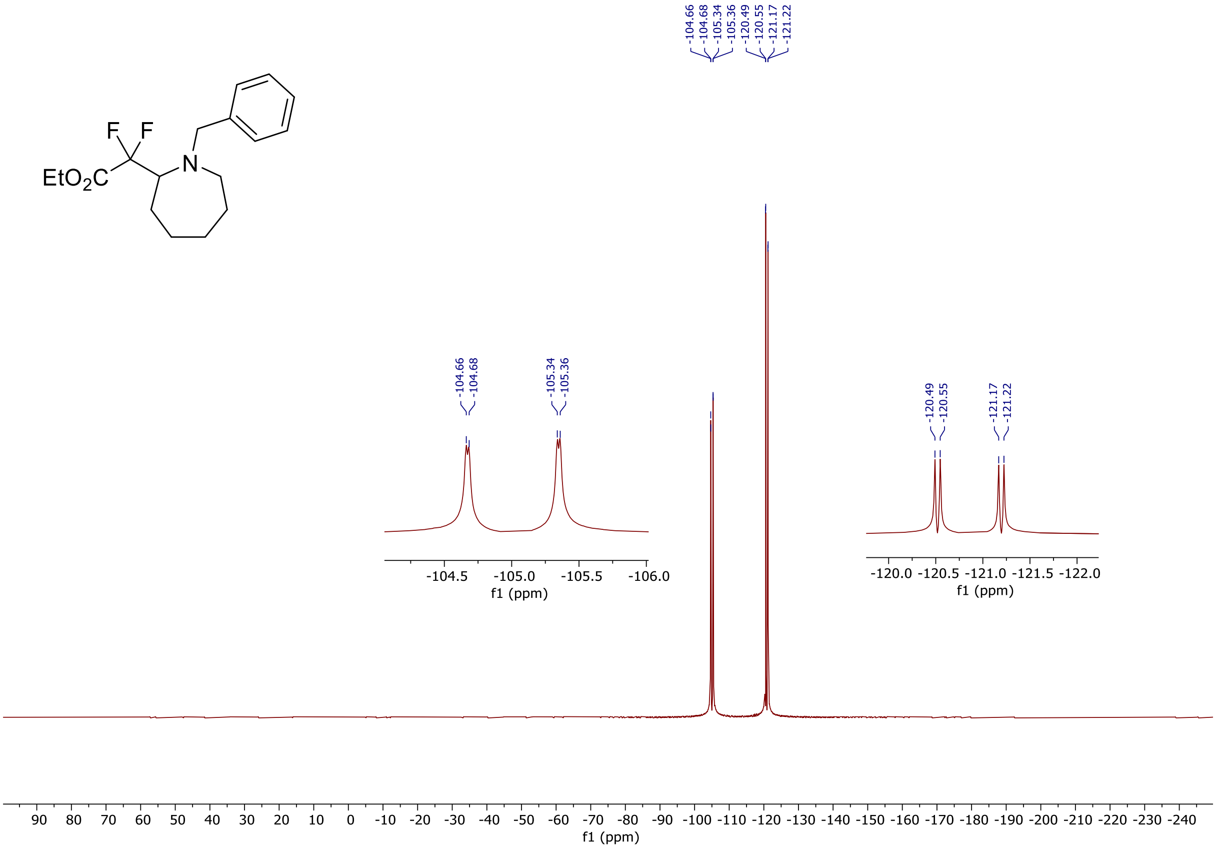

3ab – ^{1}H NMR (400 MHz, $CDCl_3$)

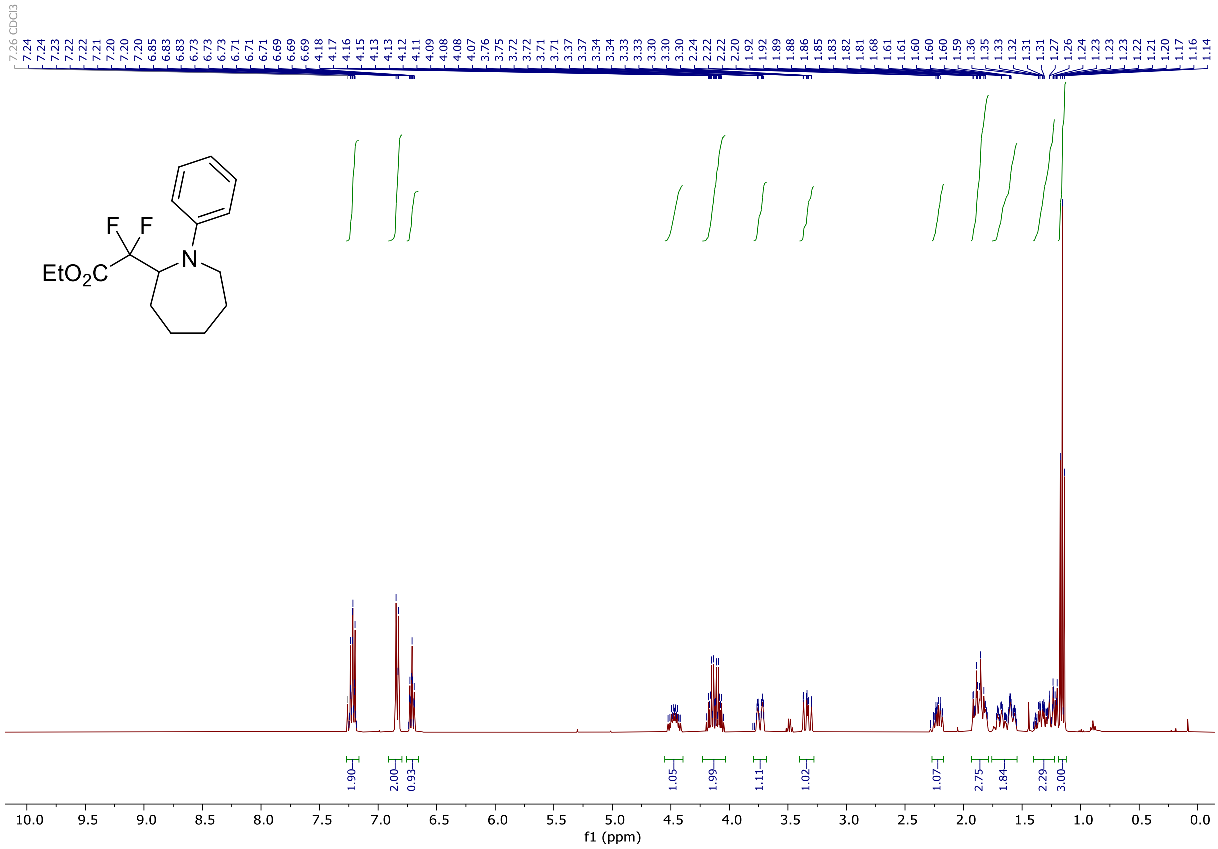

3ab – ^{13}C NMR (101 MHz, CDCl$_3$)

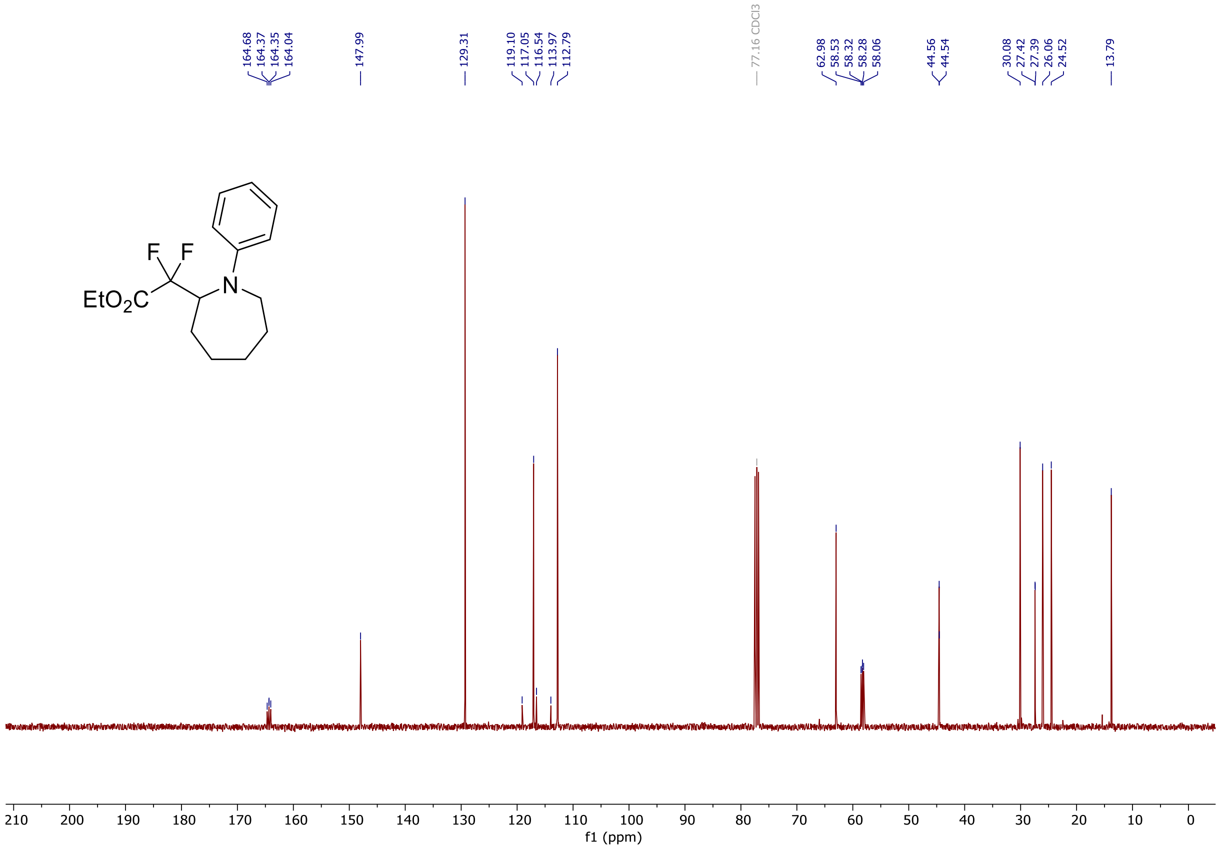

3ab – ^{19}F NMR (377 MHz, CDCl$_3$)

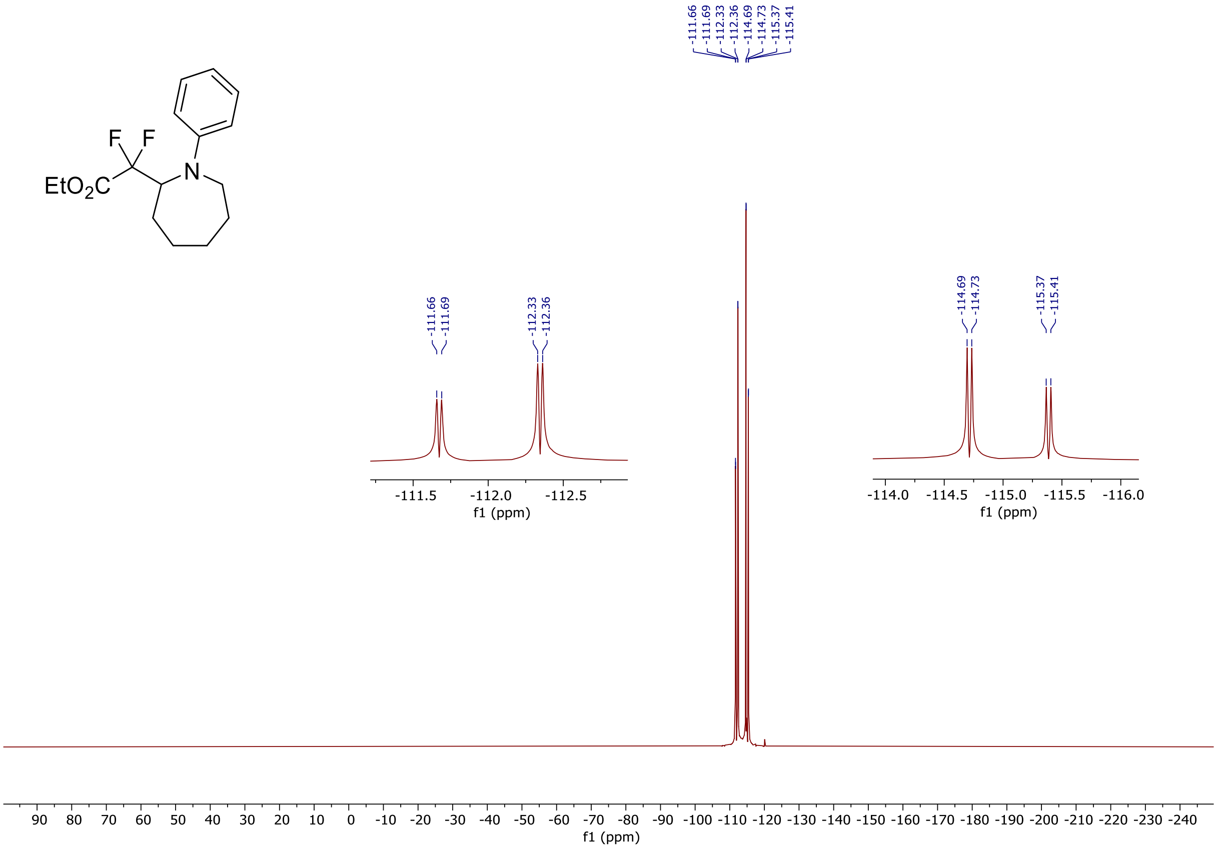

3ac – ^{1}H NMR (400 MHz, $CDCl_3$)

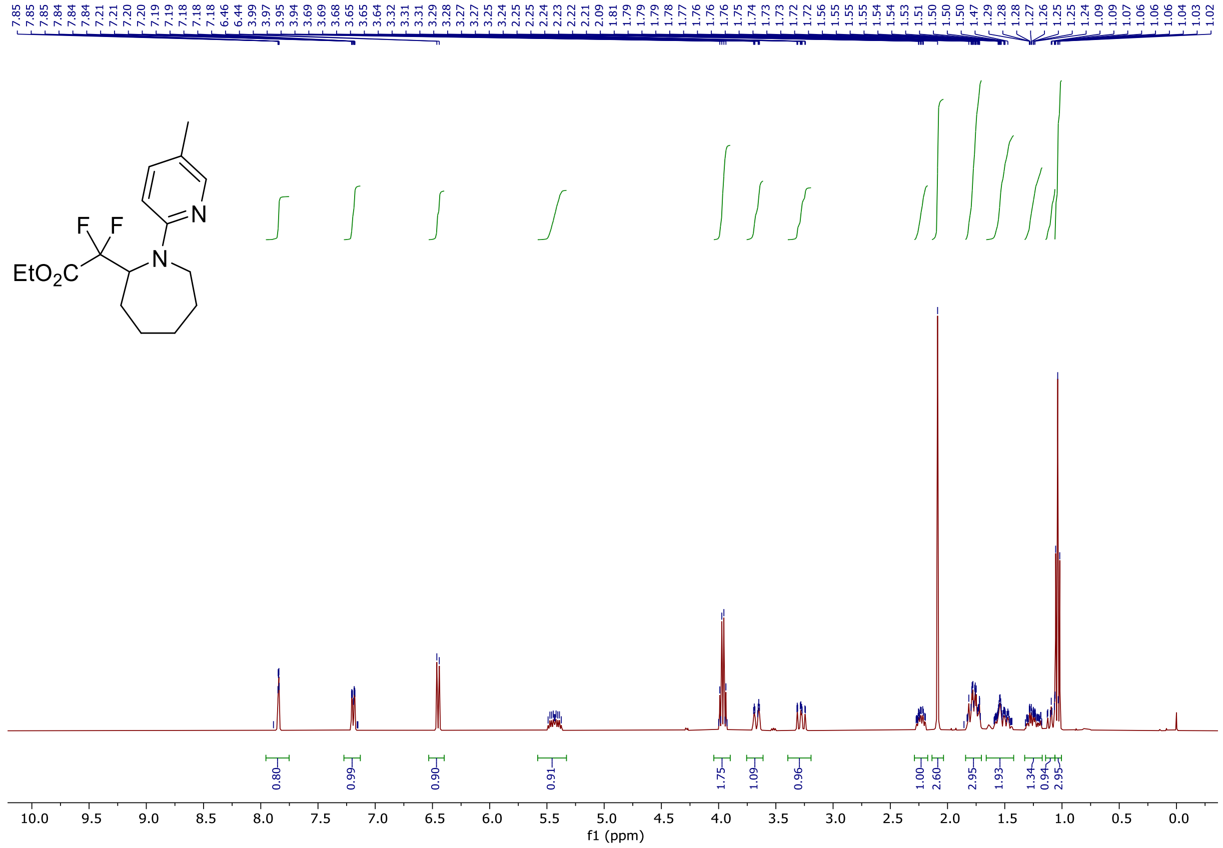

3ac – ^{13}C NMR (101 MHz, $CDCl_3$)

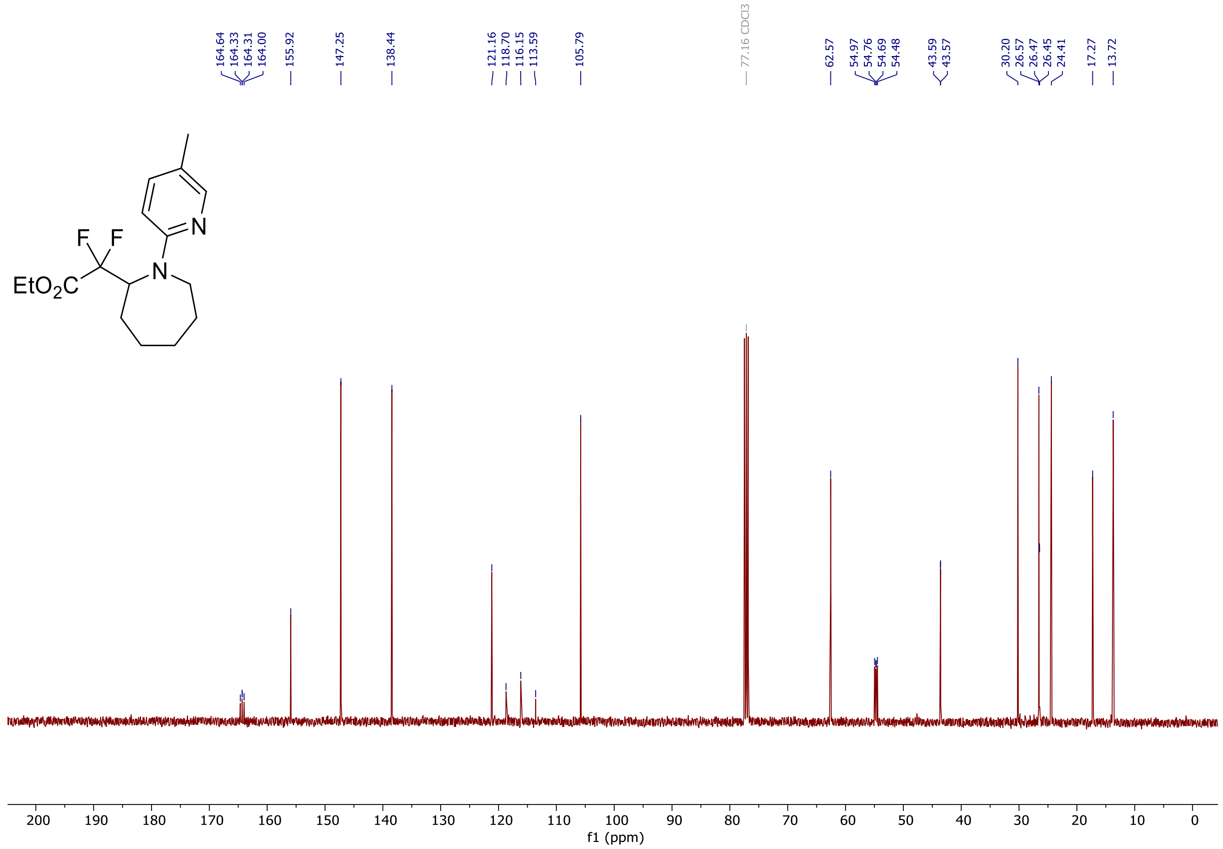

3ac – ^{19}F NMR (377 MHz, $CDCl_3$)

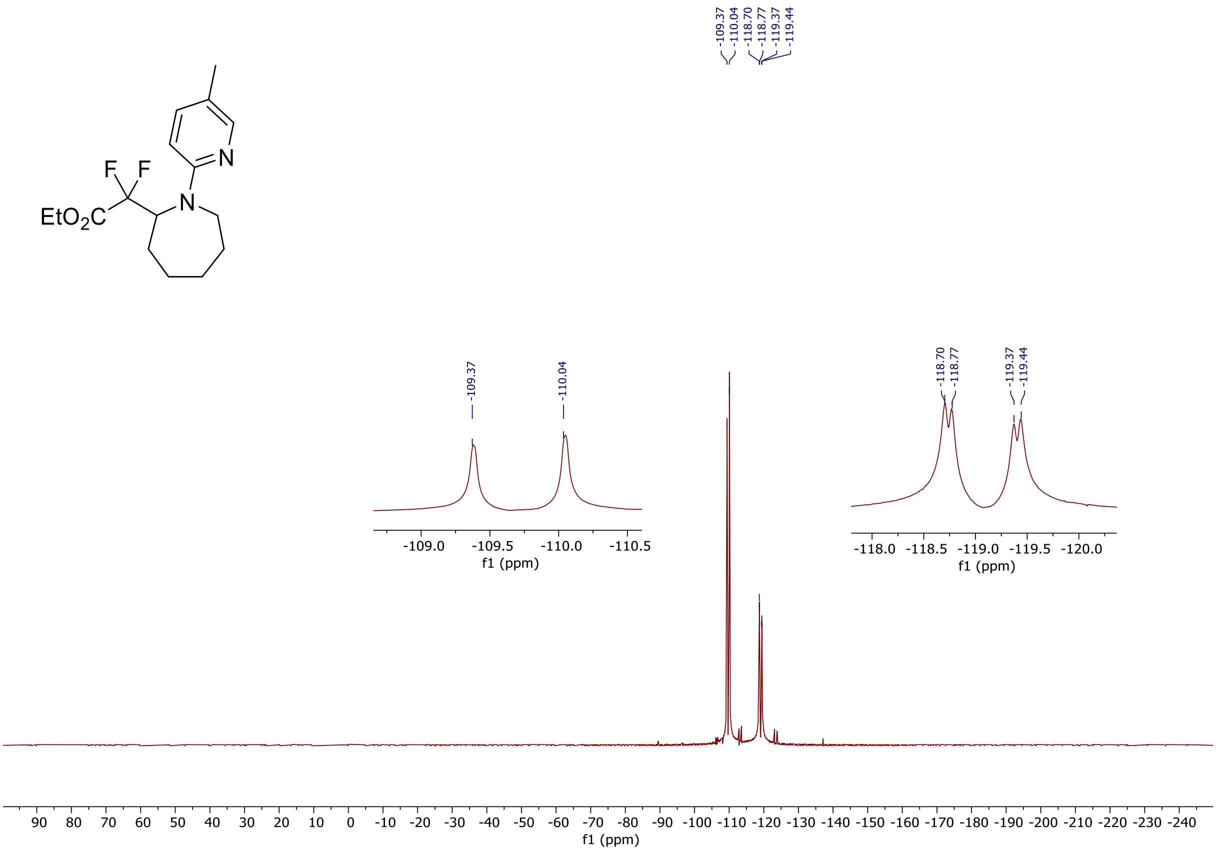

3ad – ^{1}H NMR (400 MHz, $CDCl_3$)

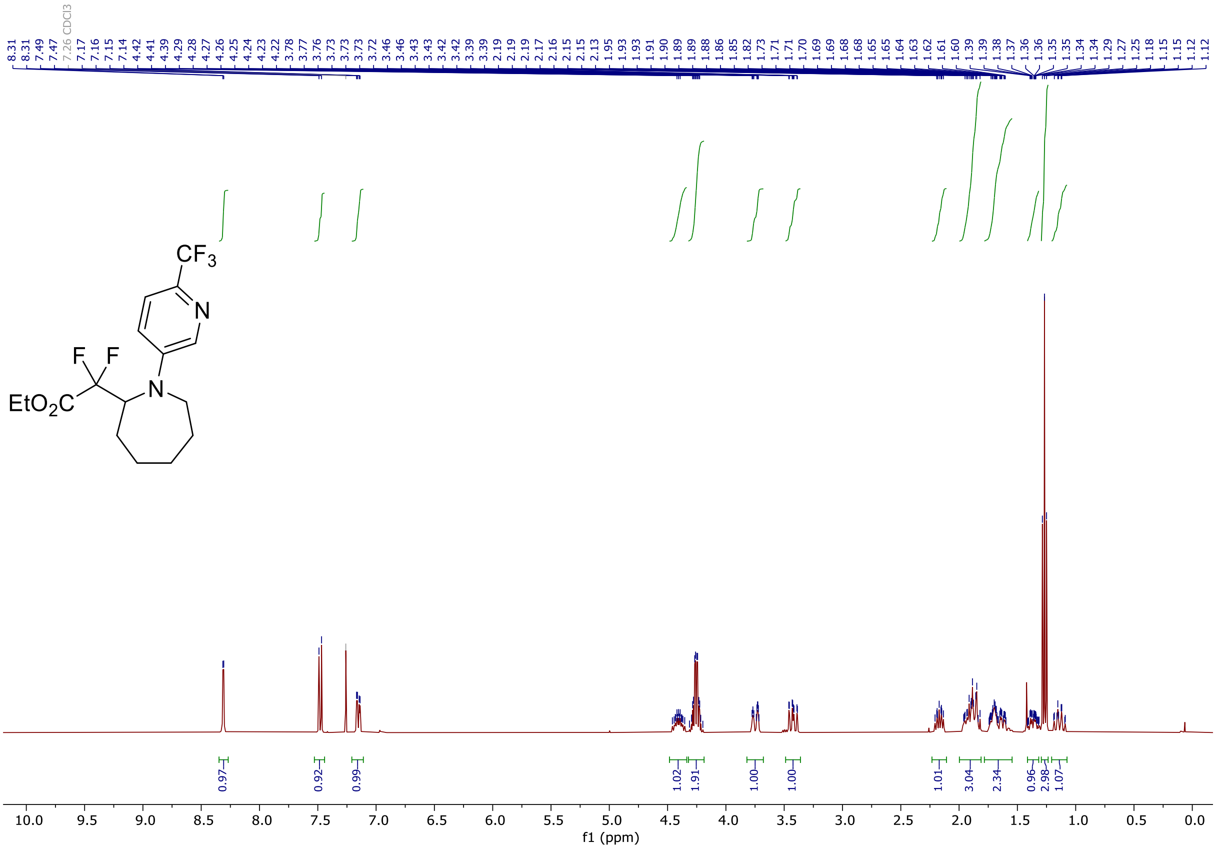

3ad – ^{13}C NMR (101 MHz, $CDCl_3$)

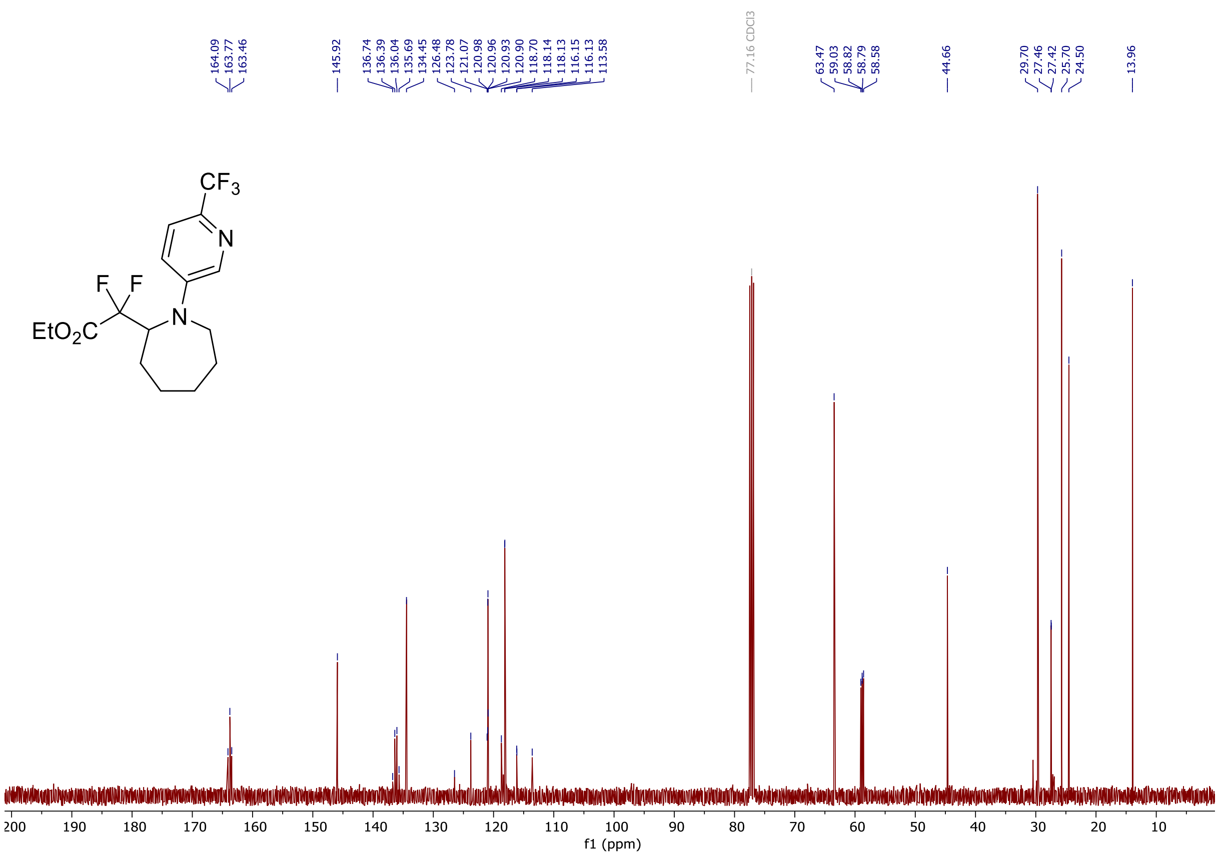

3ad – ^{19}F NMR (377 MHz, $CDCl_3$)

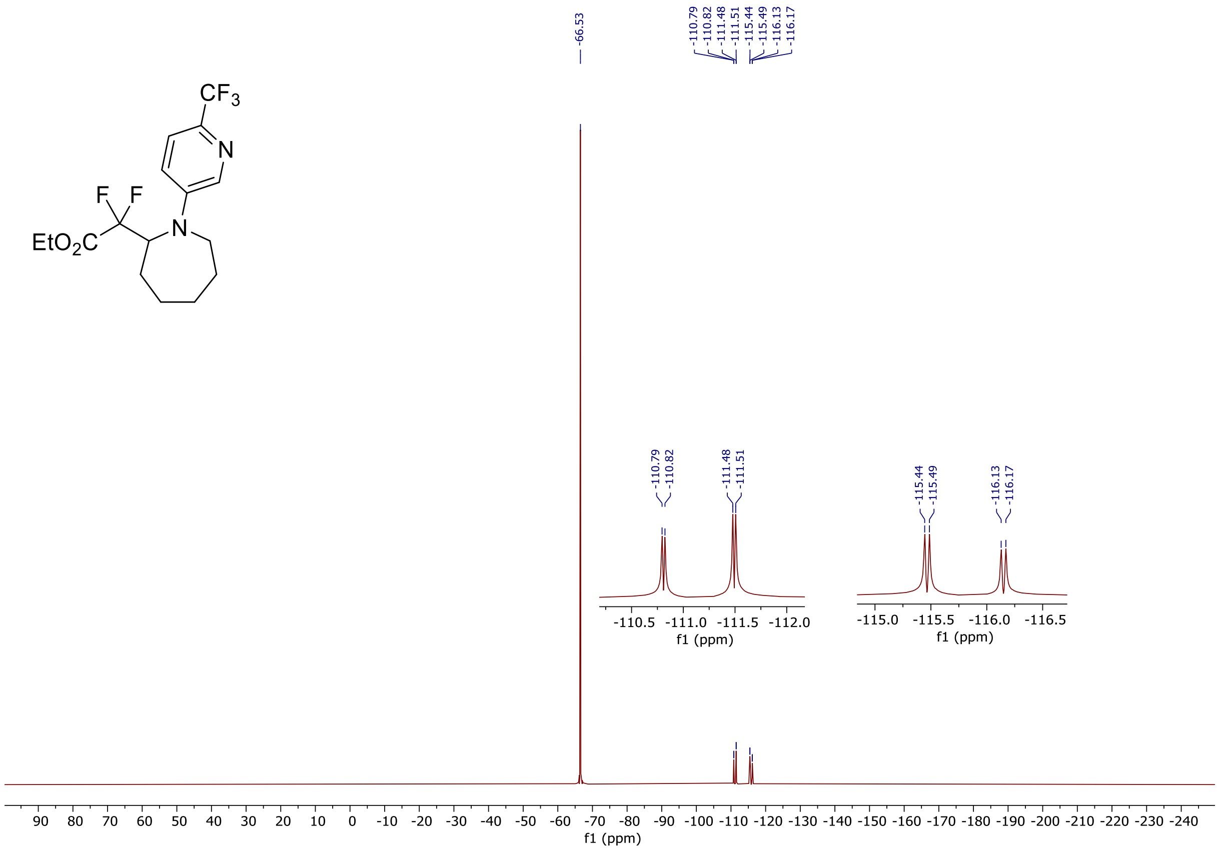

3ae – ^{1}H NMR (400 MHz, $CDCl_3$)

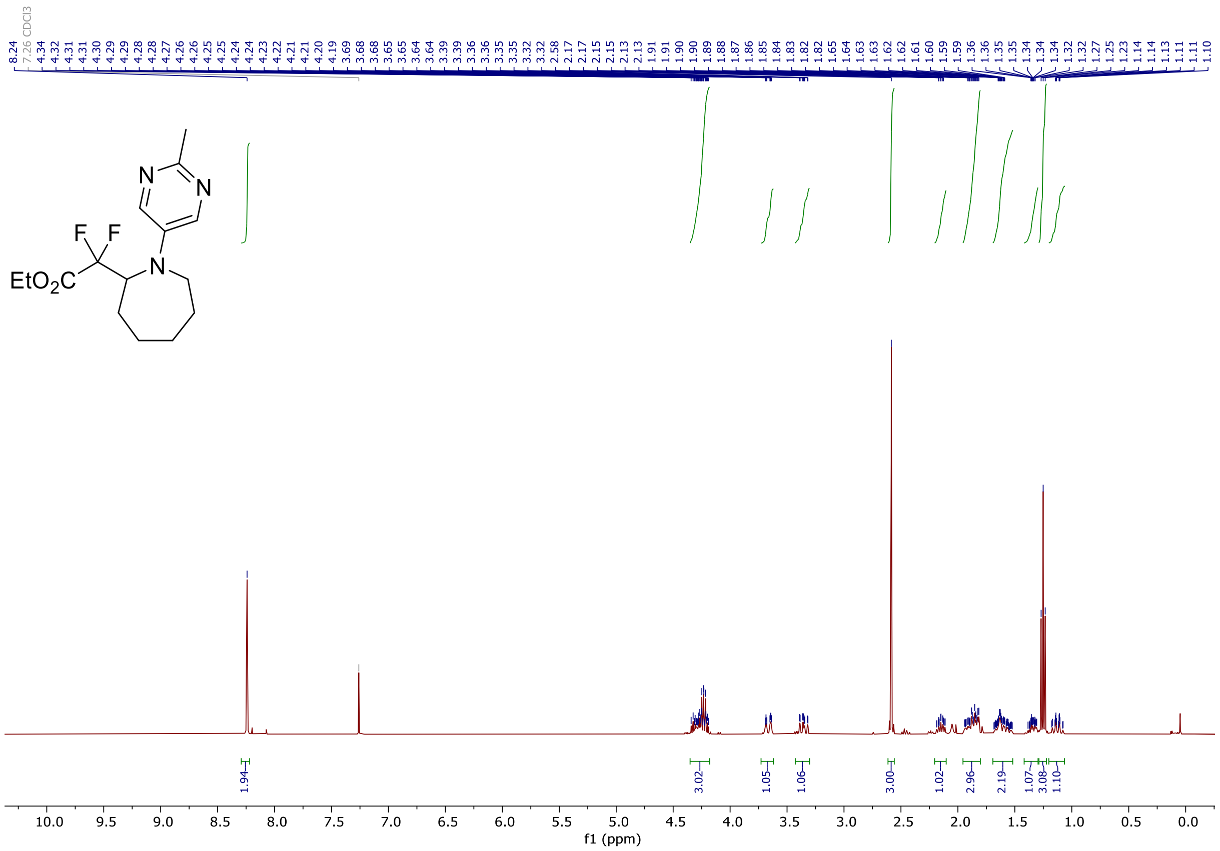

3ae – ^{13}C NMR (101 MHz, $CDCl_3$)

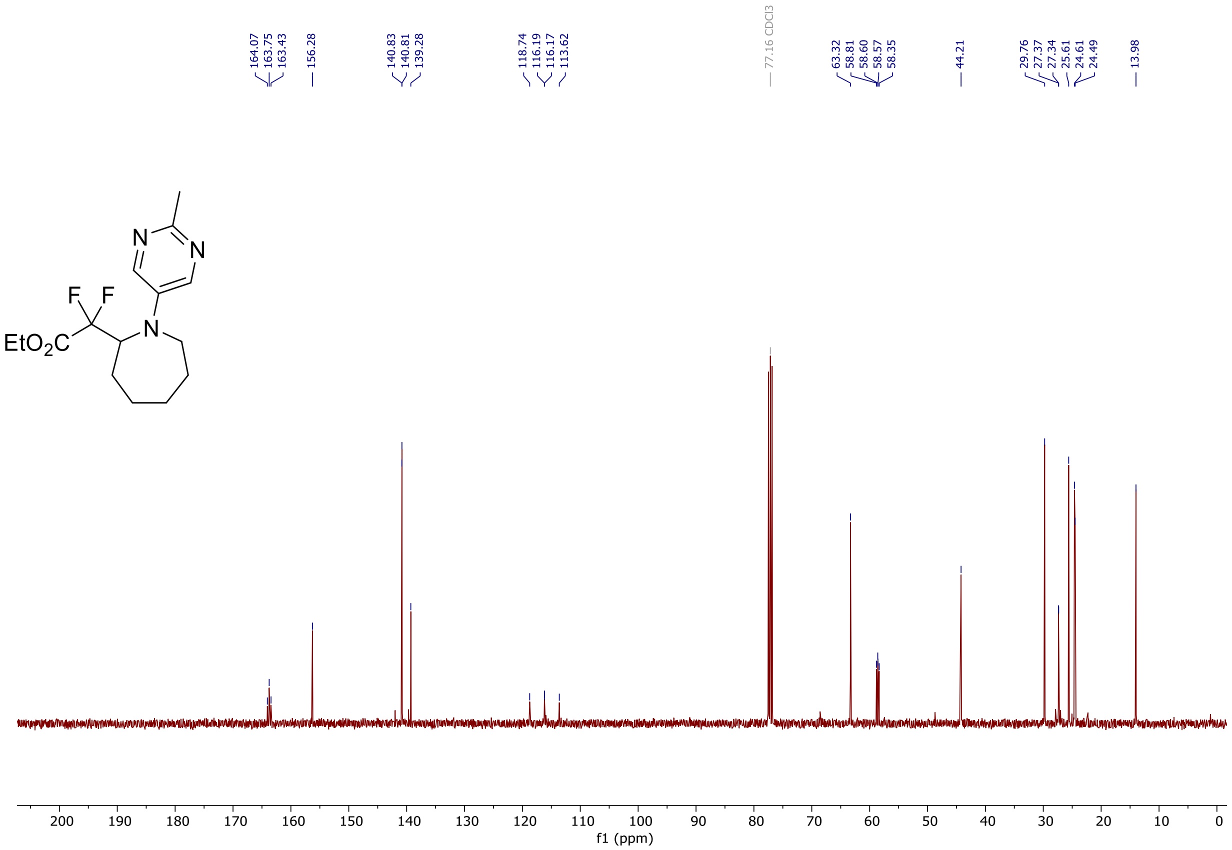

3ae – ^{19}F NMR (377 MHz, $CDCl_3$)

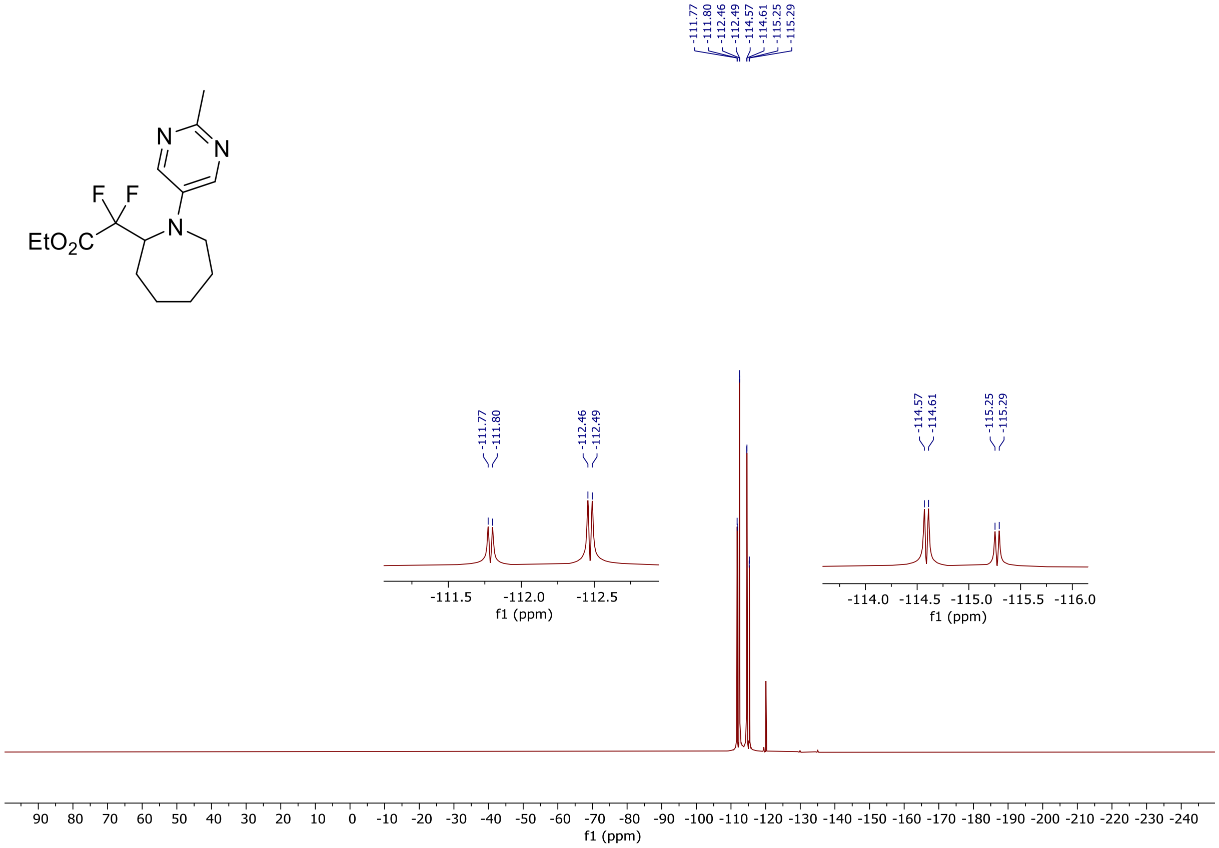

3af – ^{1}H NMR (400 MHz, $CDCl_3$)

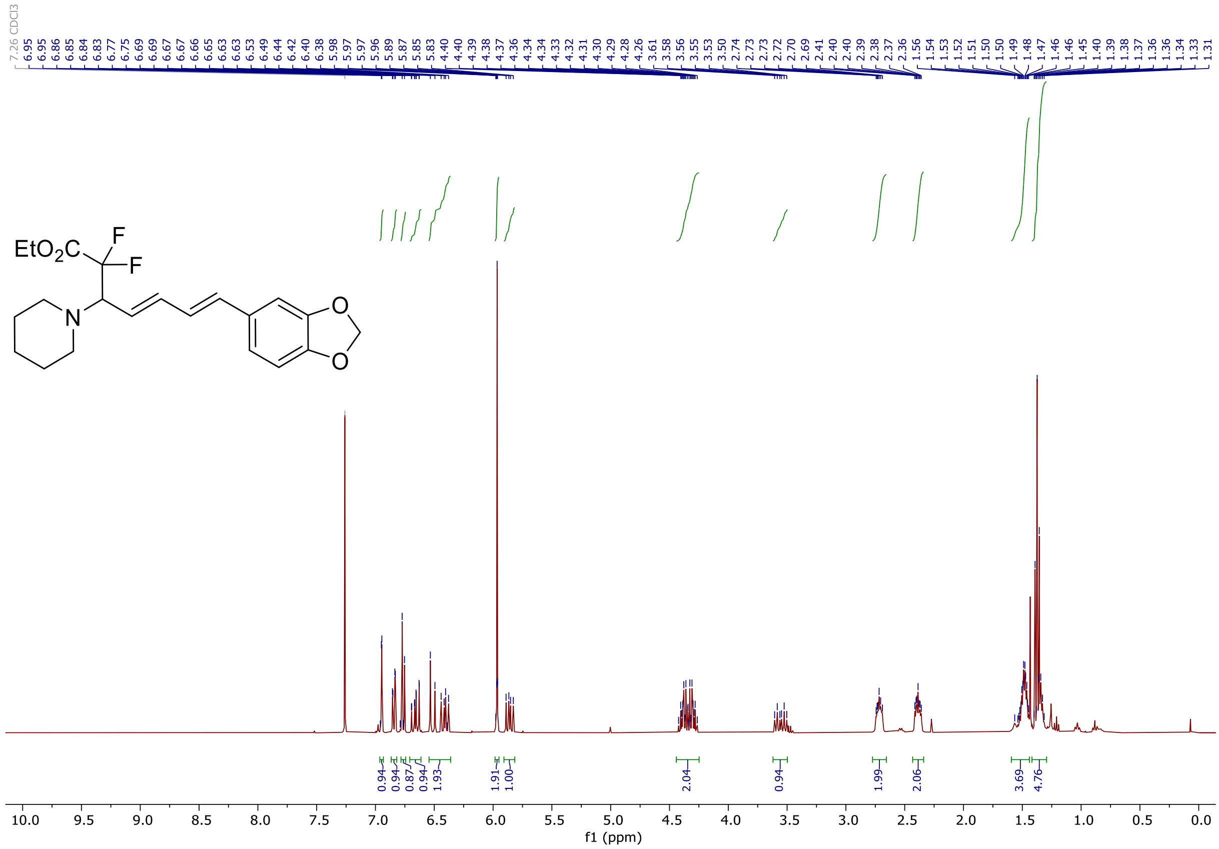

3af – ^{13}C NMR (126 MHz, CDCl$_3$)

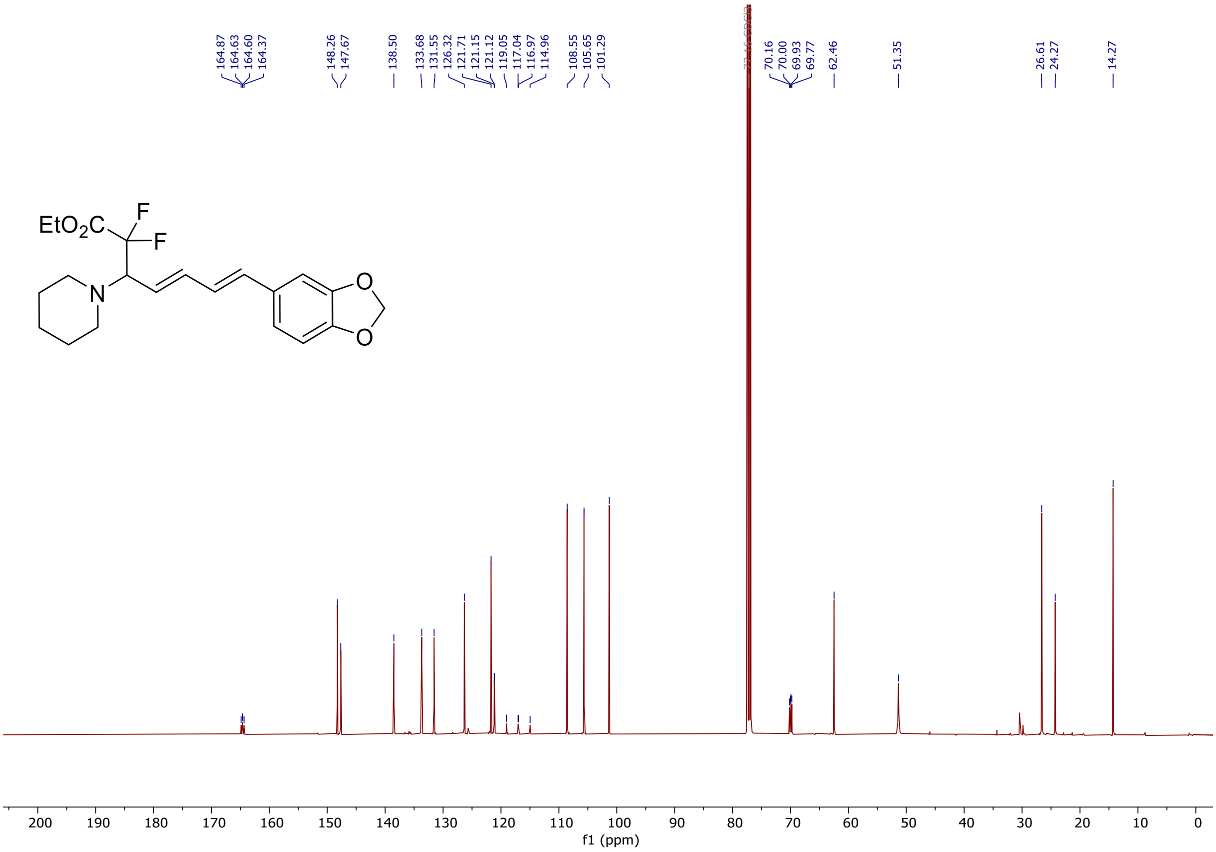

3af – ^{19}F NMR (377 MHz, CDCl$_3$)

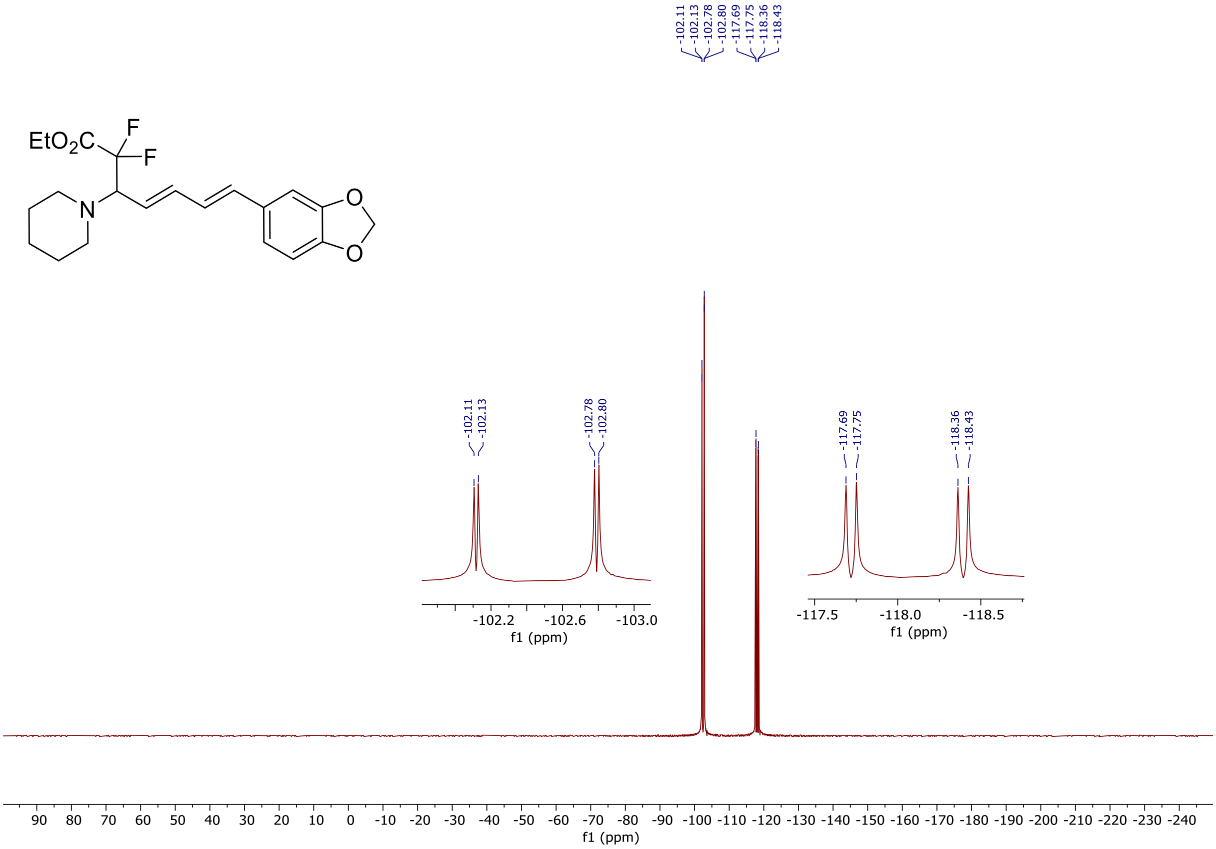

3ag – ^{1}H NMR (400 MHz, $CDCl_3$)

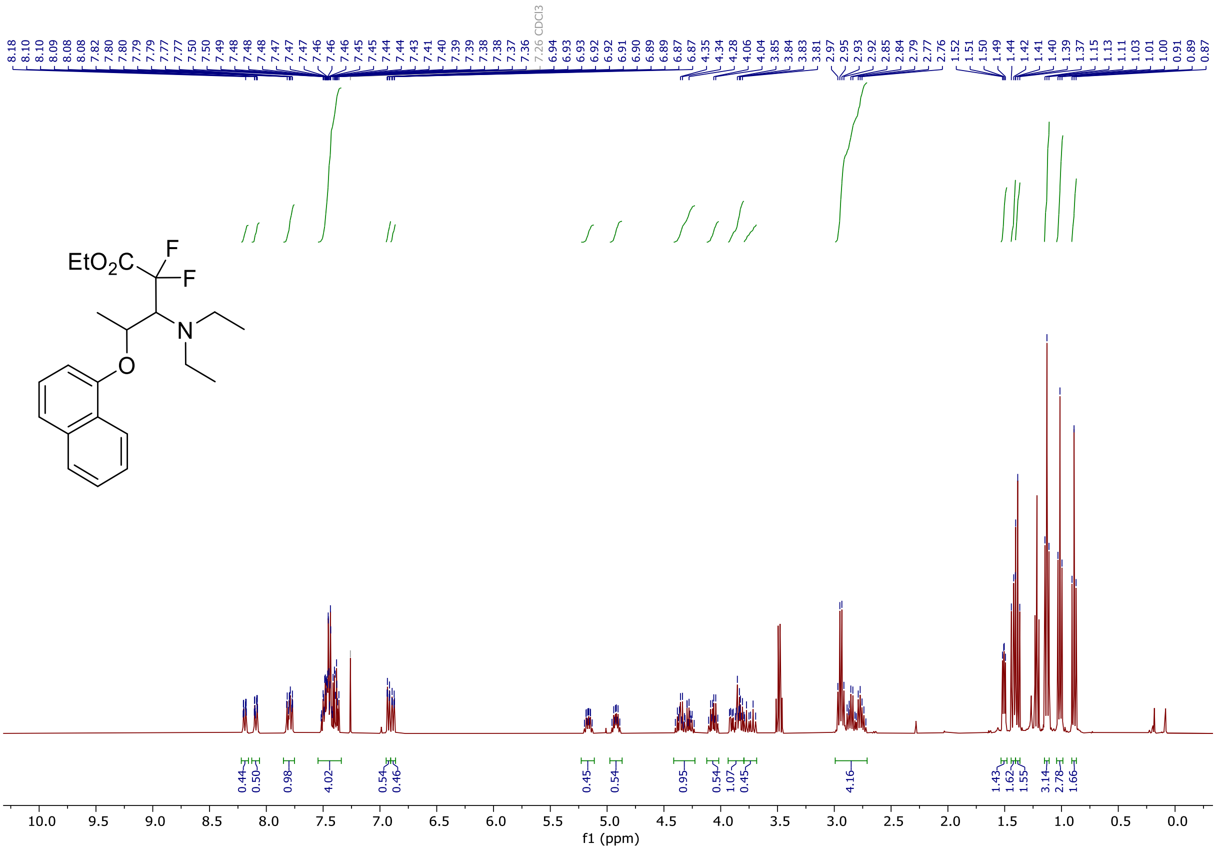

3ag – ^{13}C NMR (126 MHz, $CDCl_3$)

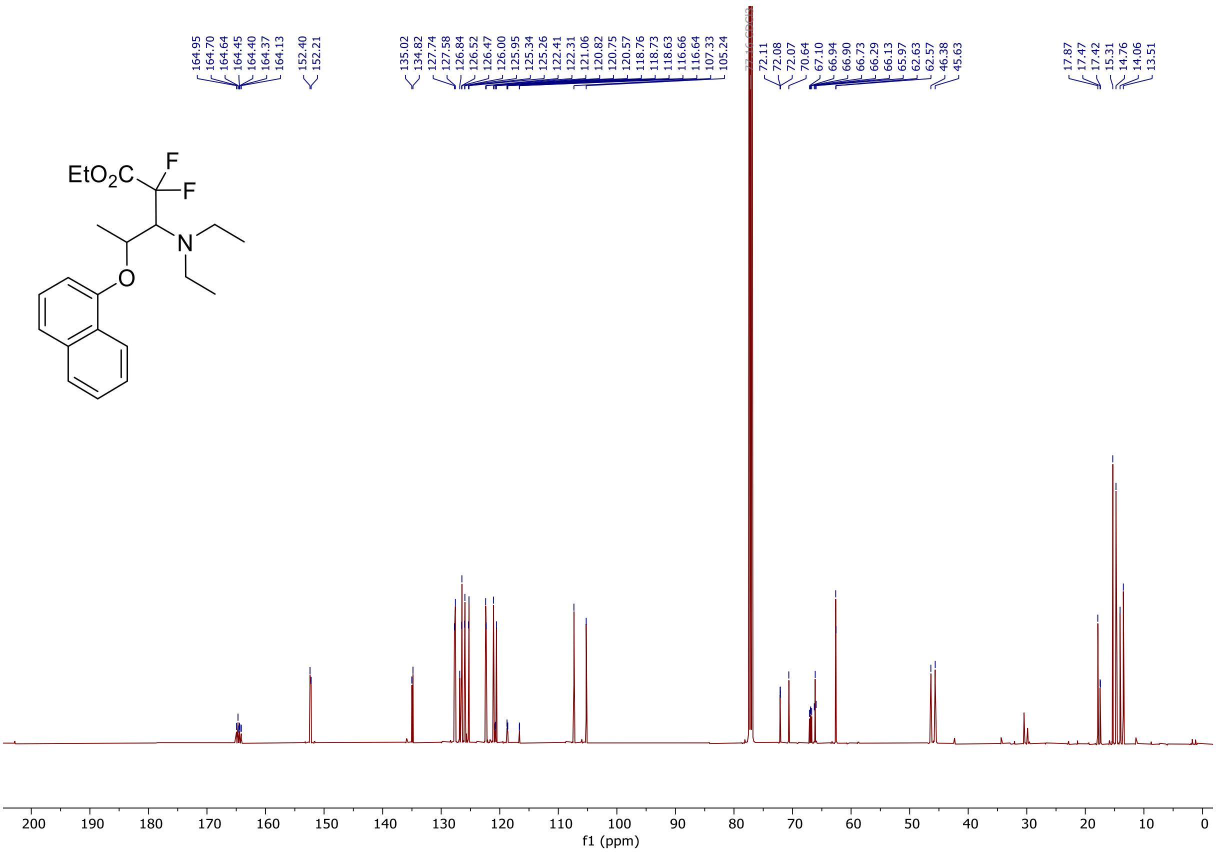

3ag – ^{19}F NMR (377 MHz, $CDCl_3$)

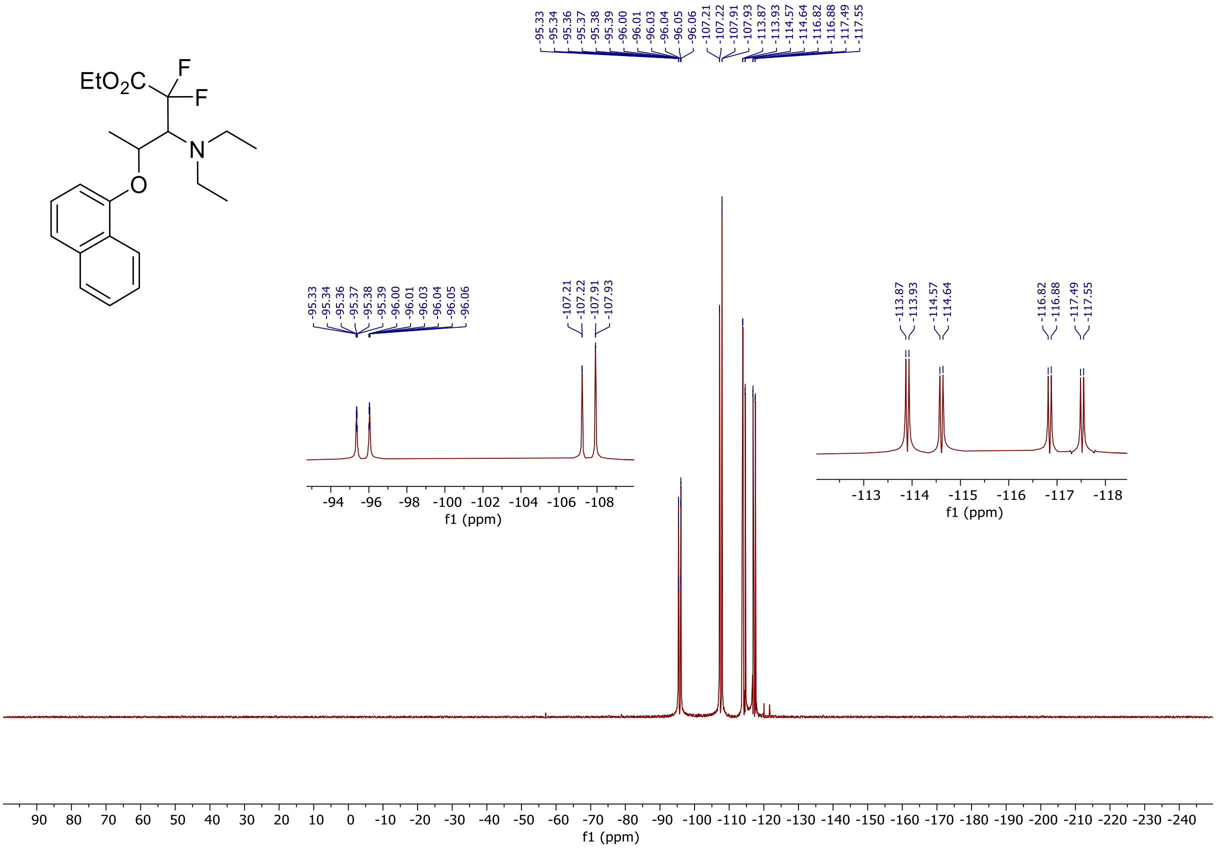

3ah – ^{1}H NMR (400 MHz, $CDCl_3$)

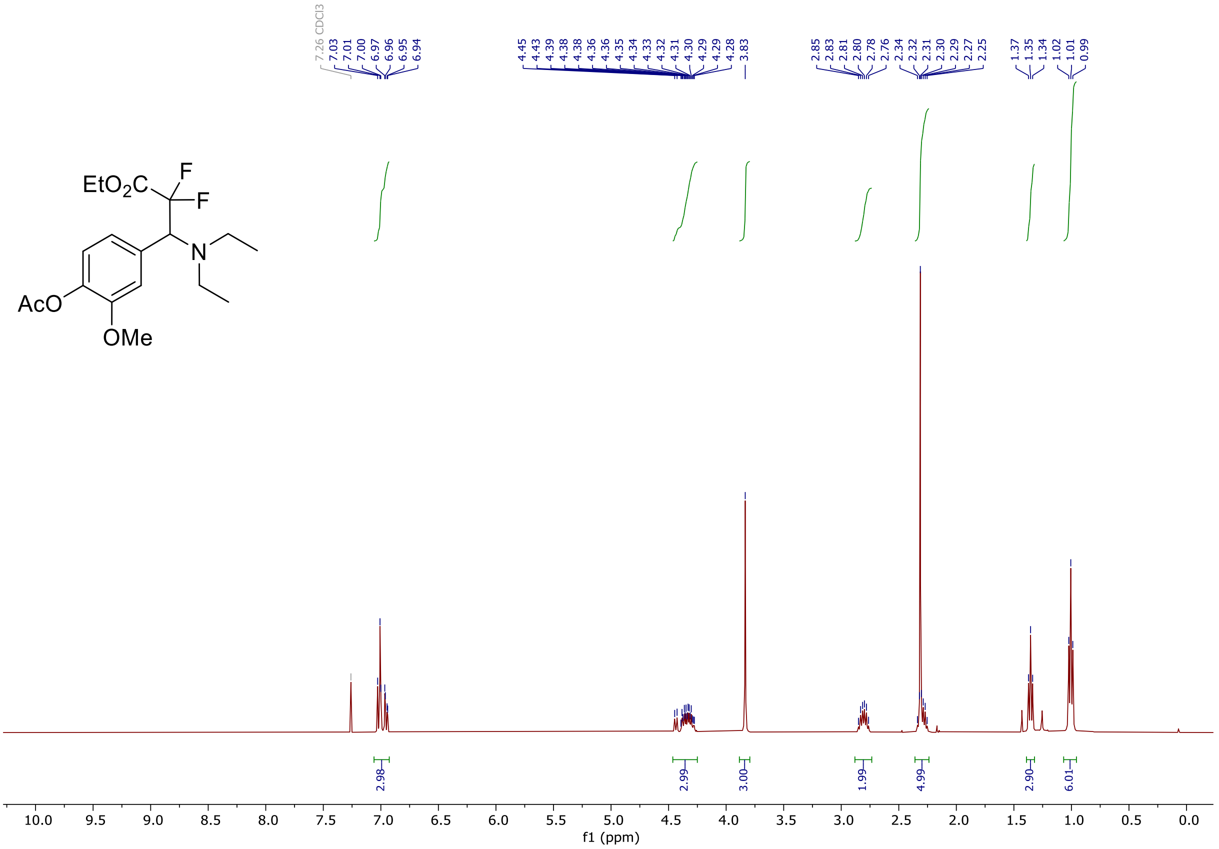

3ah – ^{13}C NMR (126 MHz, $CDCl_3$)

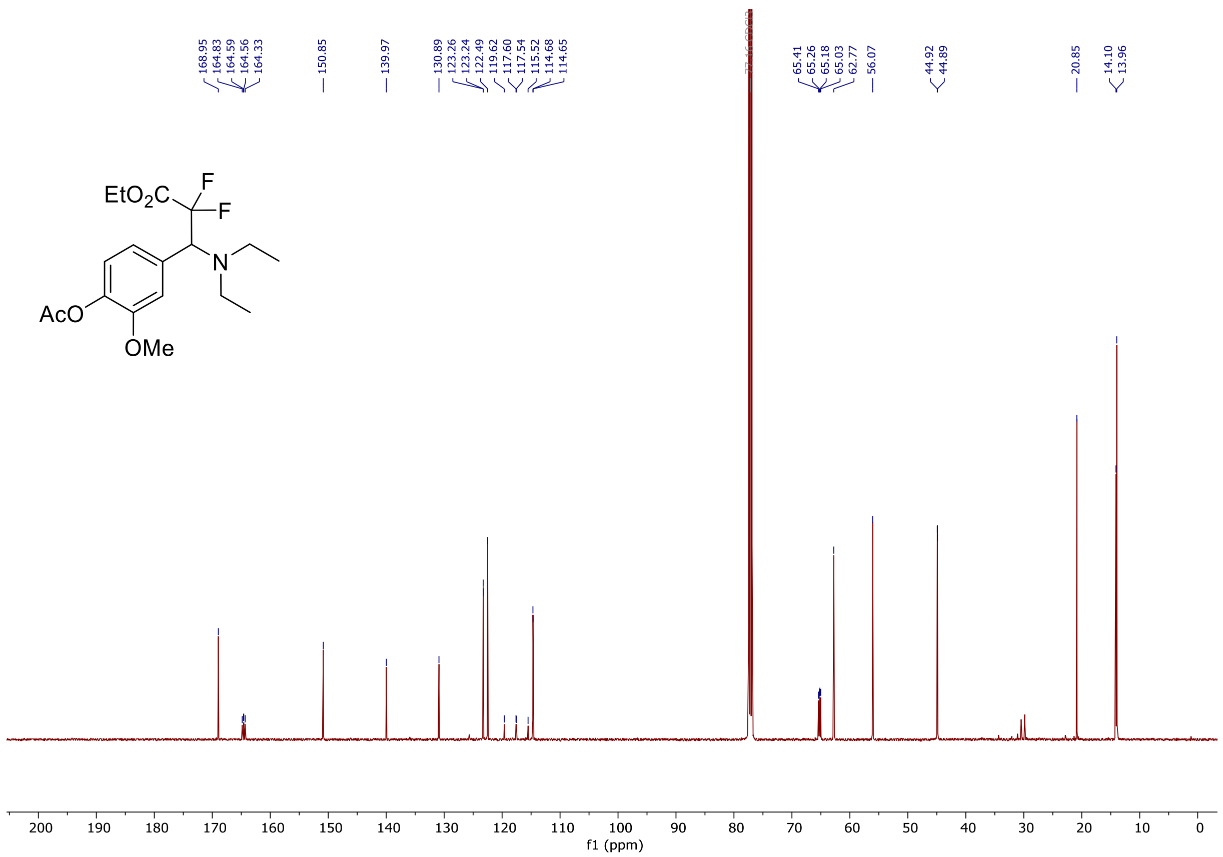

3ah – ^{19}F NMR (377 MHz, $CDCl_3$)

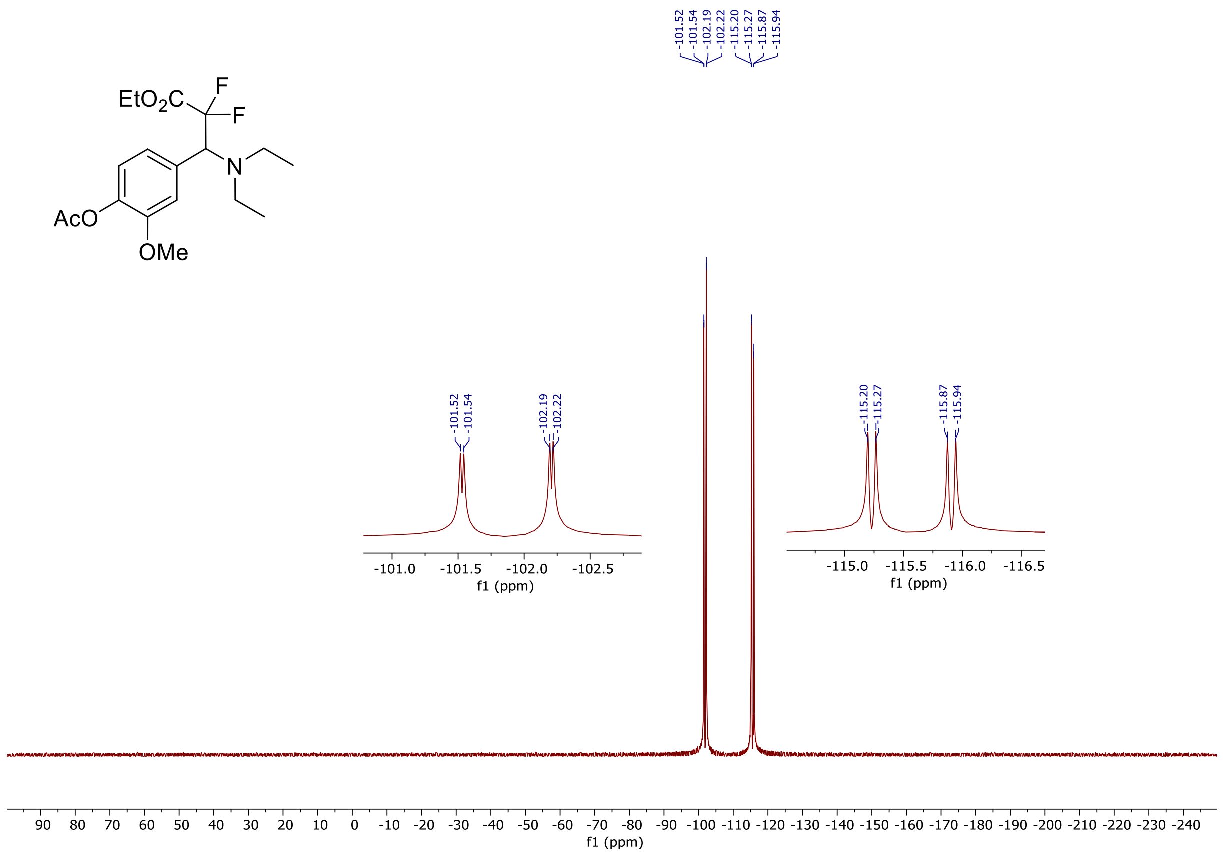

3ai – ^{1}H NMR (400 MHz, $CDCl_3$)

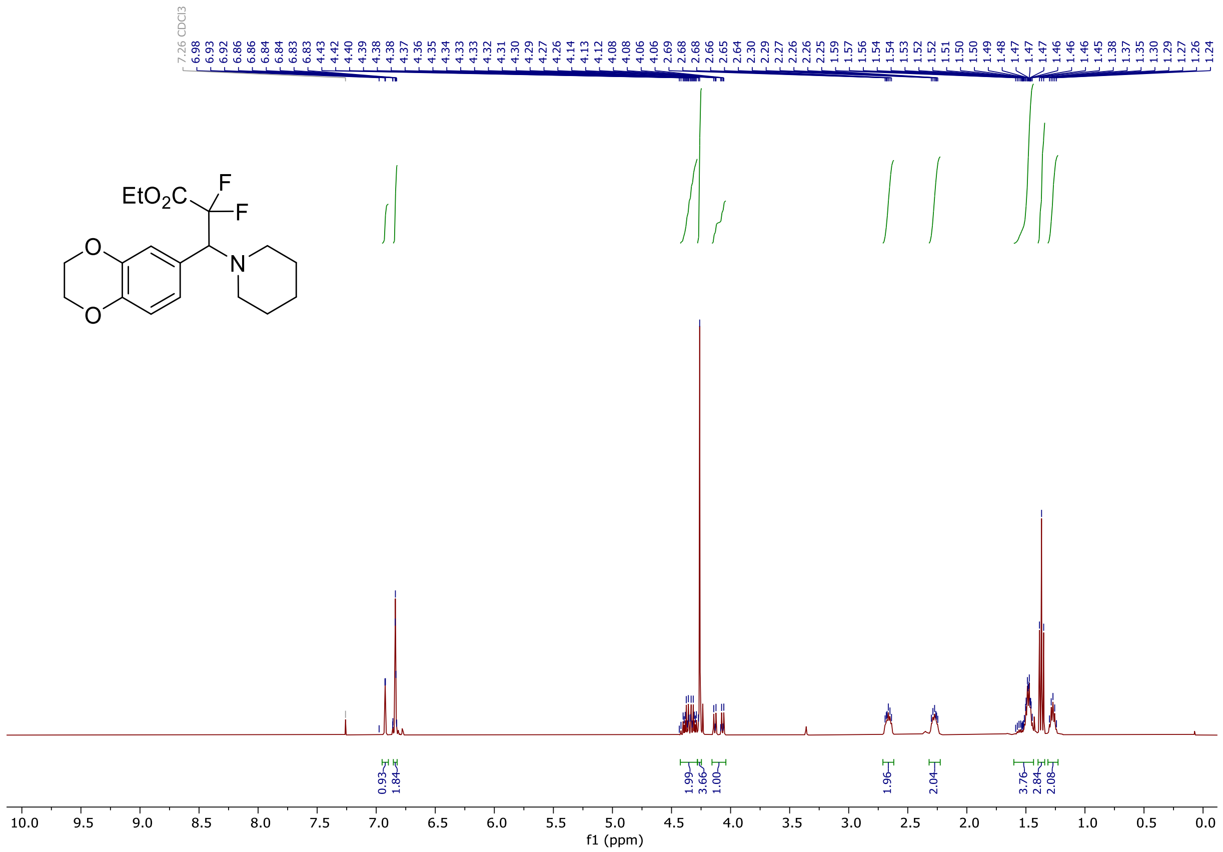

3ai – ^{13}C NMR (101 MHz, $CDCl_3$)

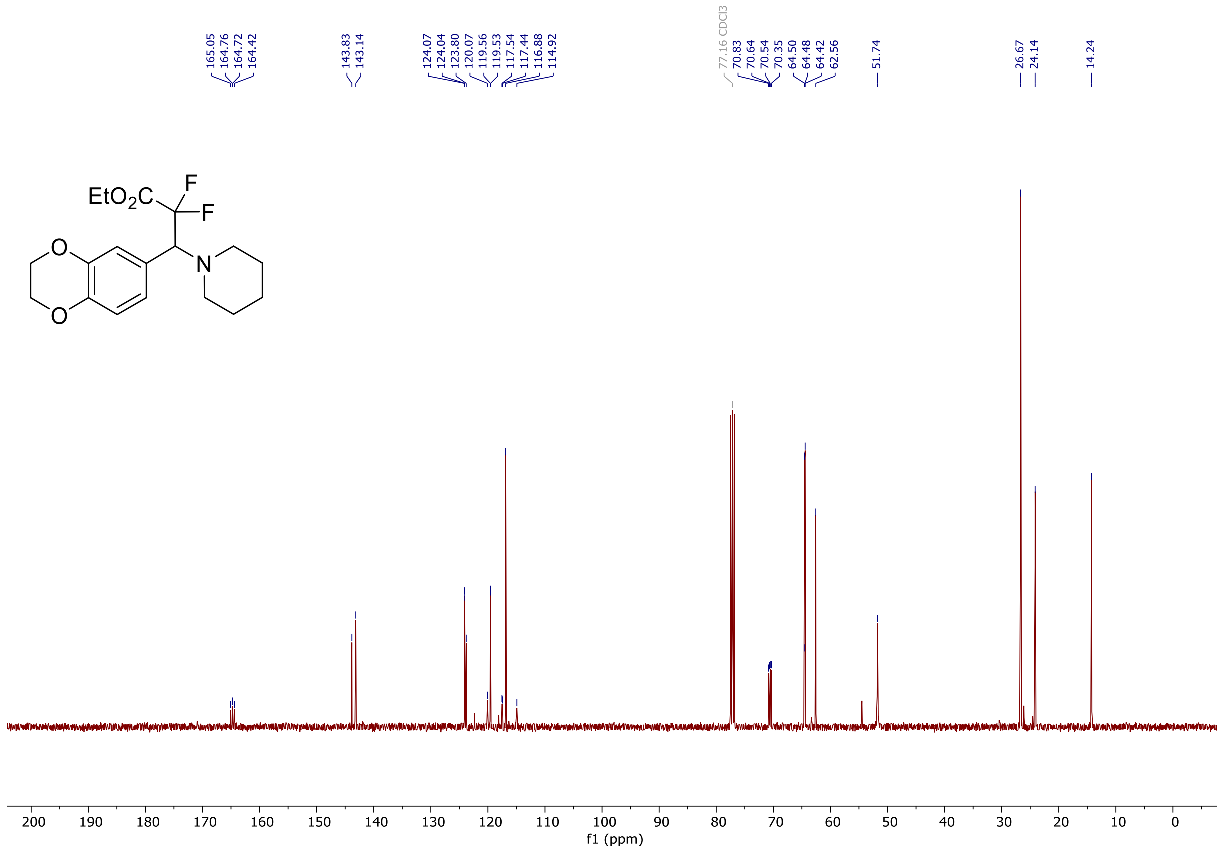

3ai – ^{19}F NMR (377 MHz, $CDCl_3$)

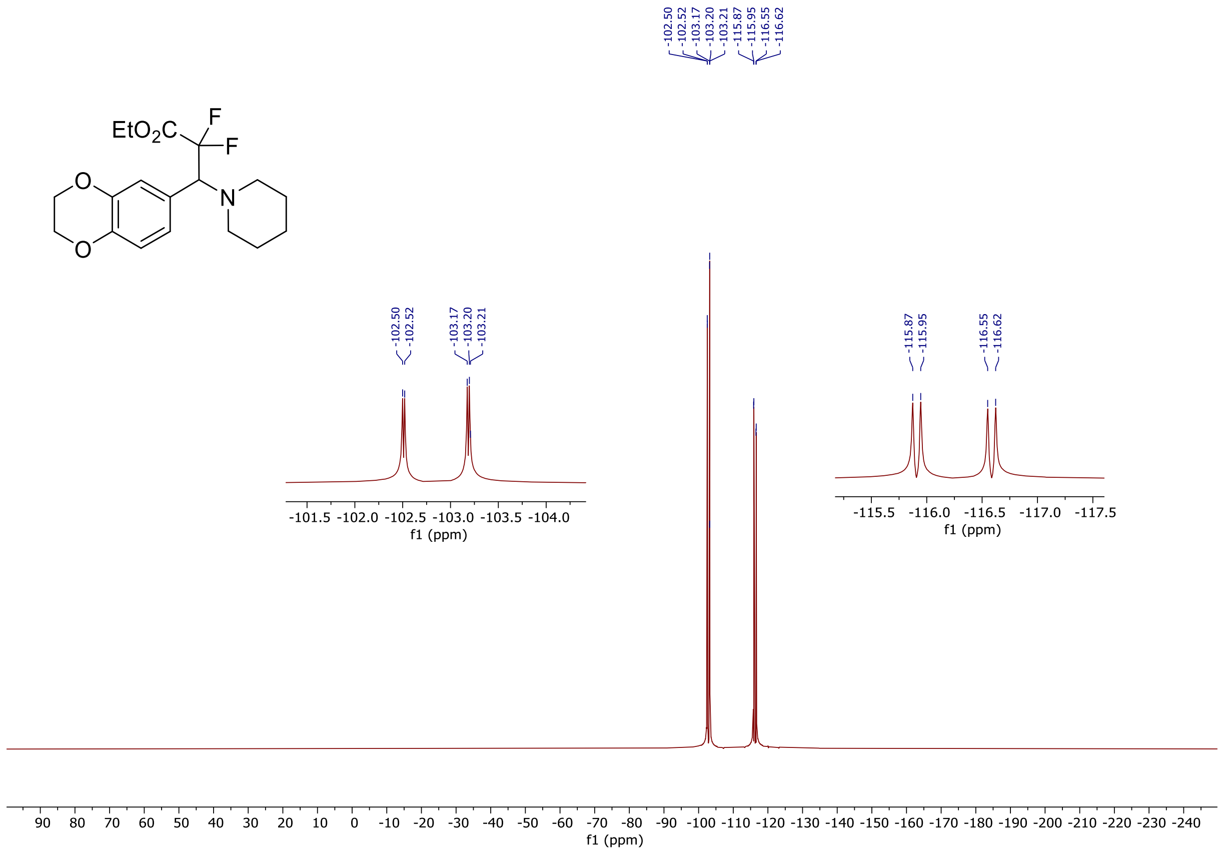

3aj – ^{1}H NMR (400 MHz, $CDCl_3$)

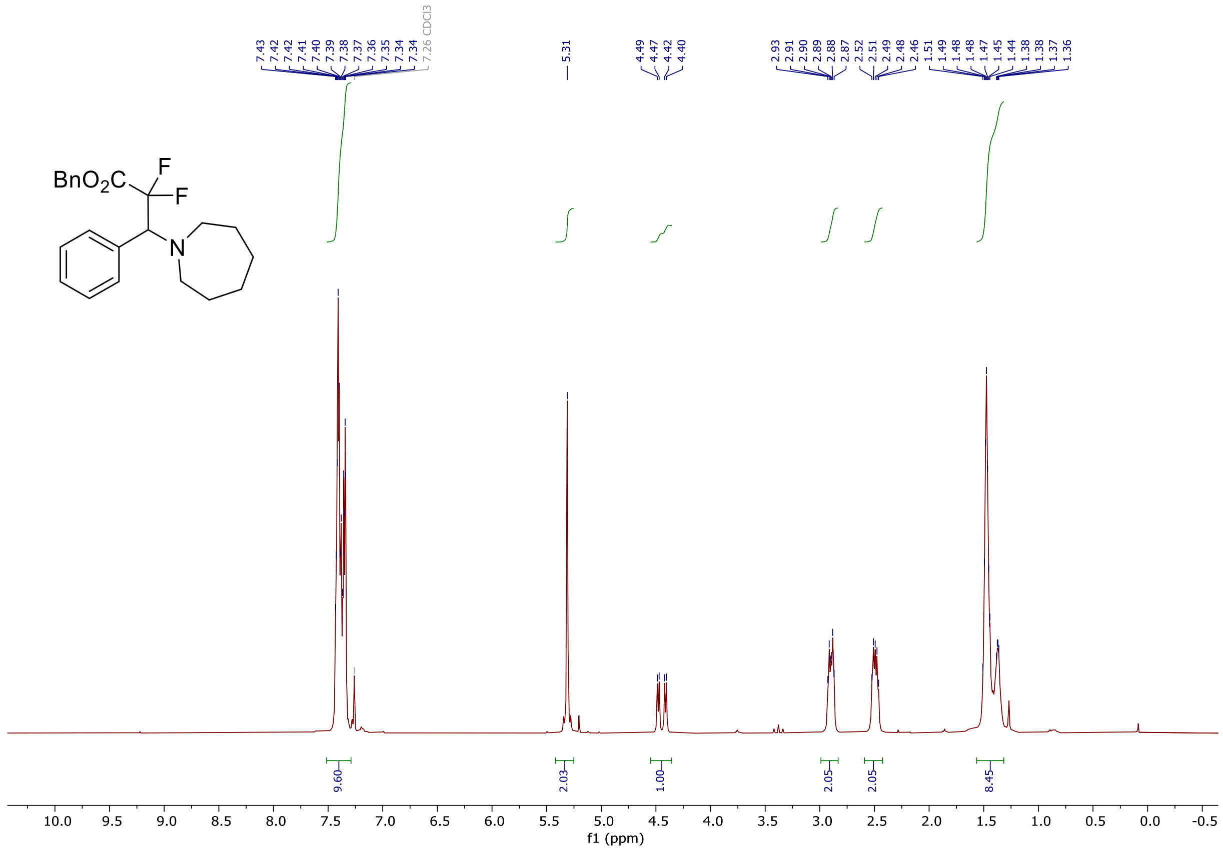

3aj – ^{13}C NMR (126 MHz, $CDCl_3$)

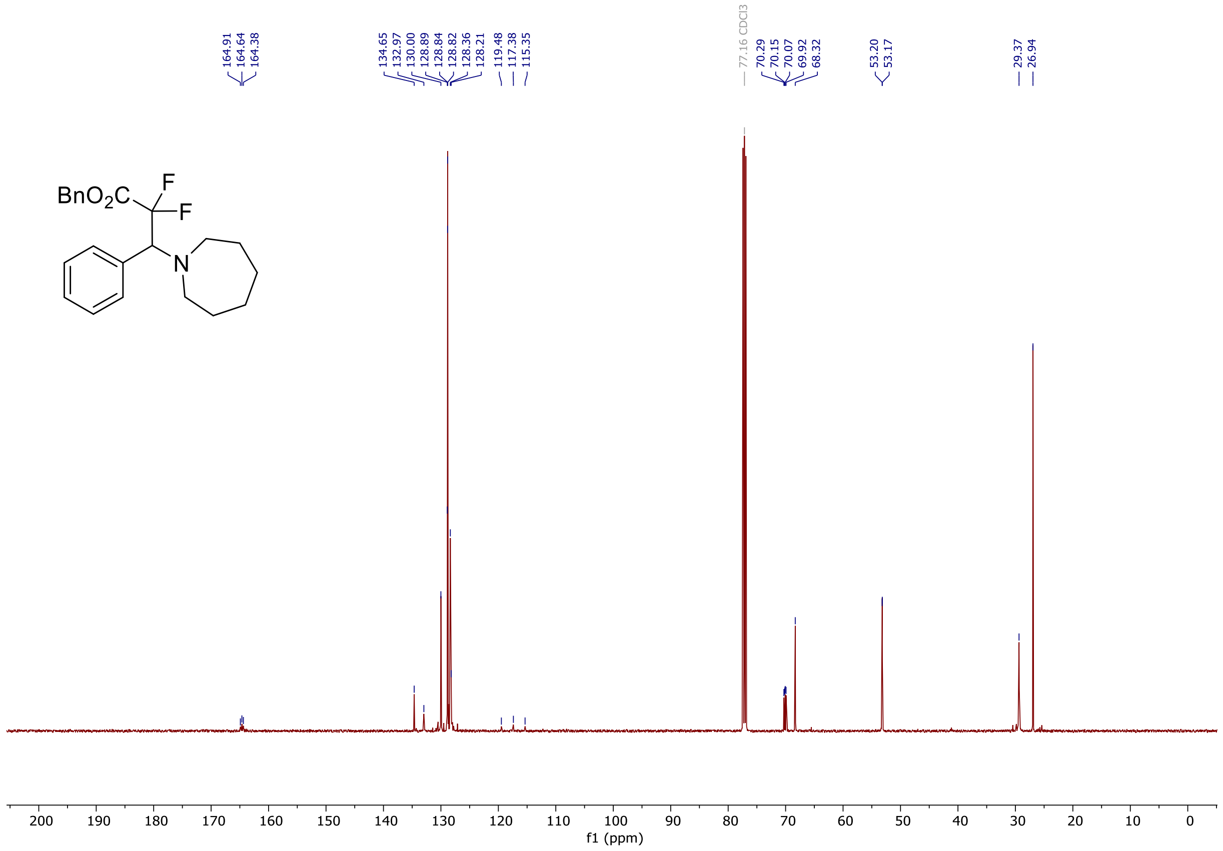

3aj – ^{19}F NMR (377 MHz, $CDCl_3$)

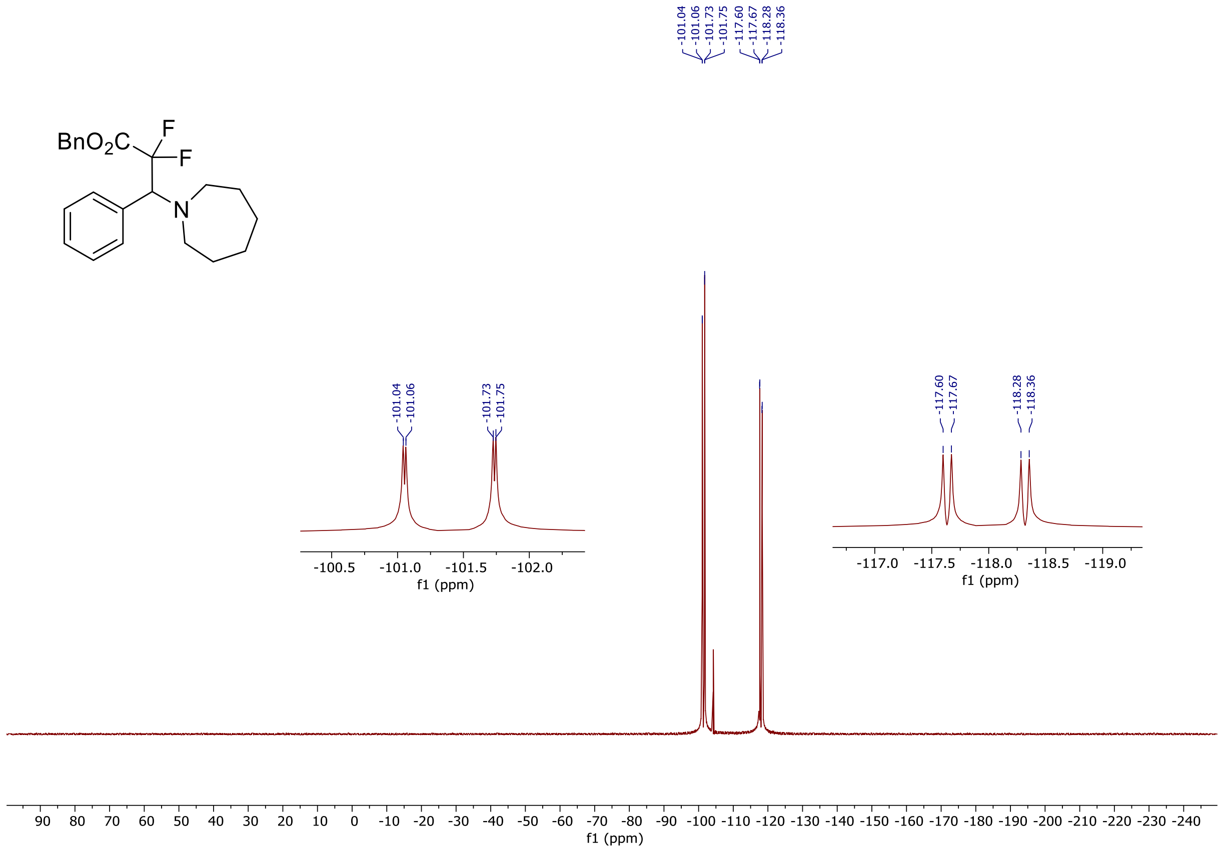

3ak – ^{1}H NMR (400 MHz, $CDCl_3$)

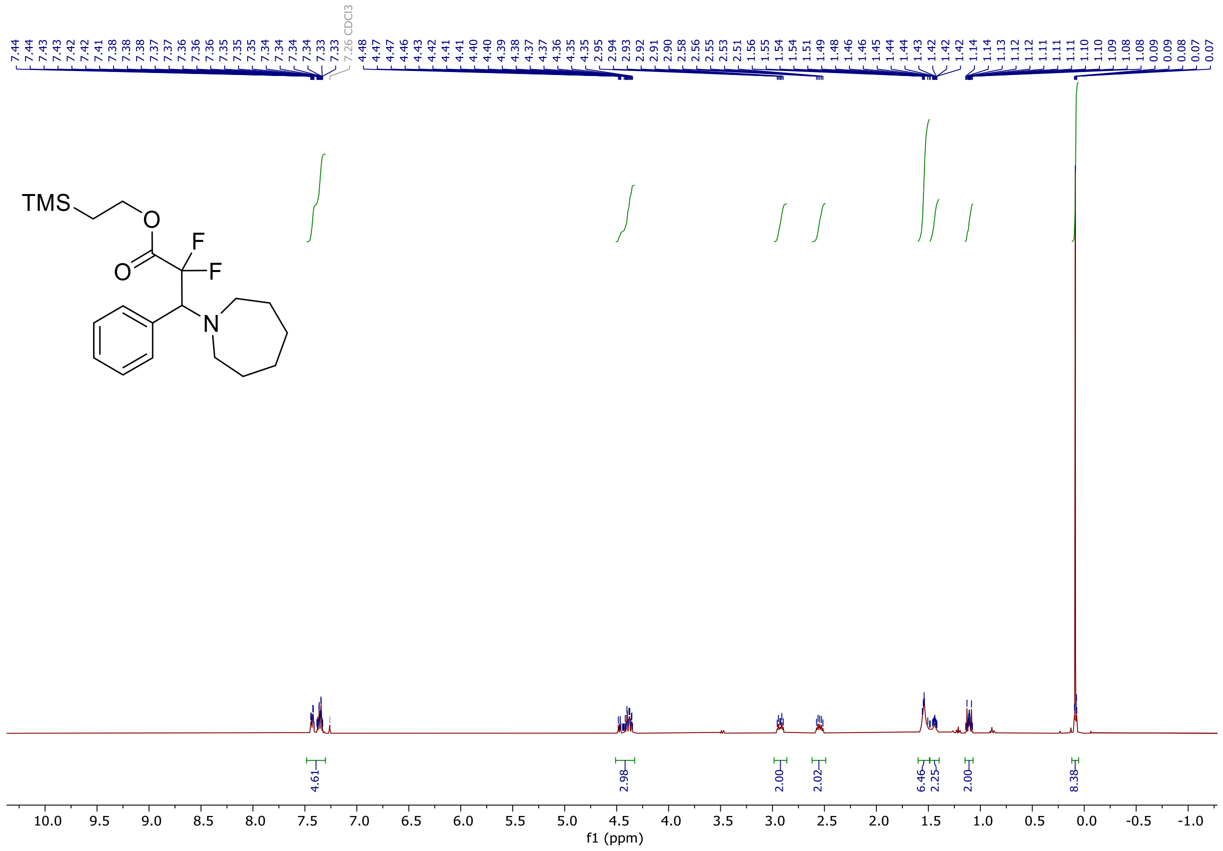

3ak – ^{13}C NMR (101 MHz, $CDCl_3$)

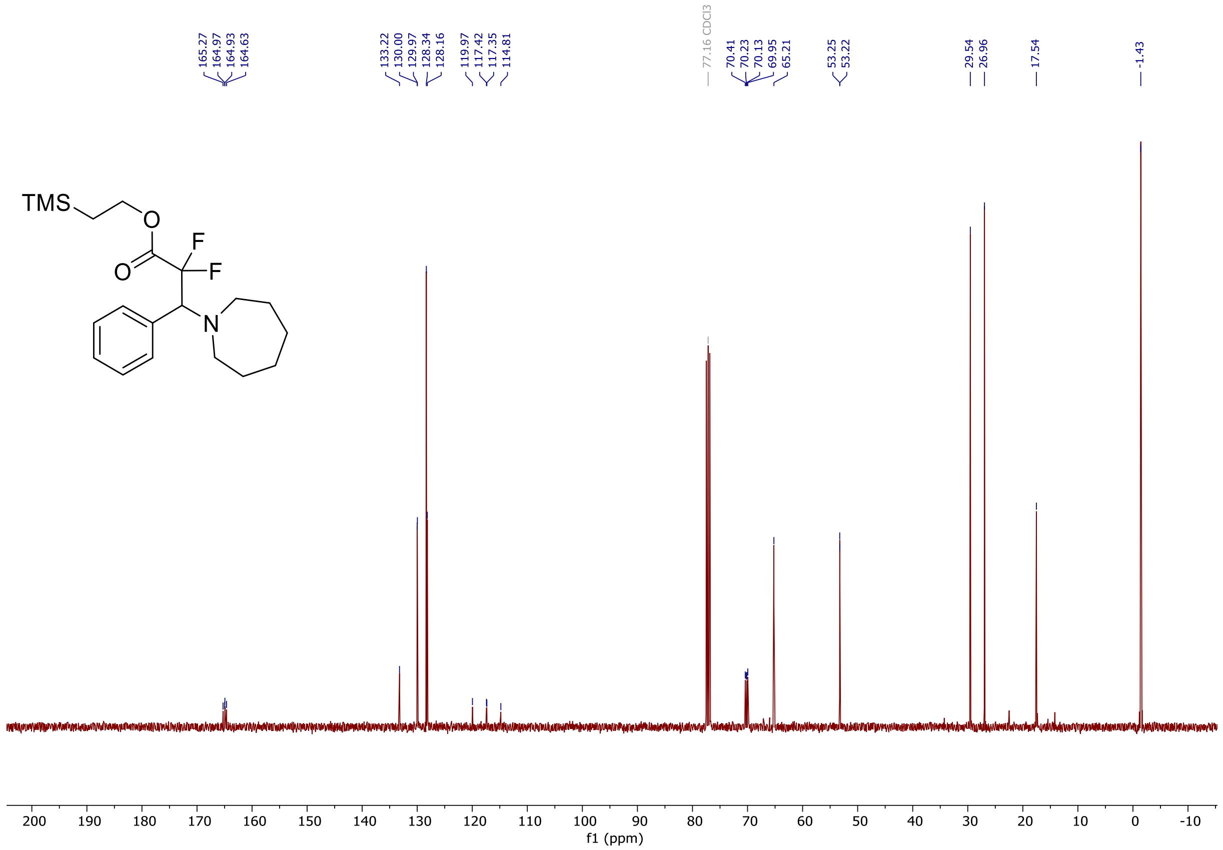

3ak – ^{19}F NMR (377 MHz, $CDCl_3$)

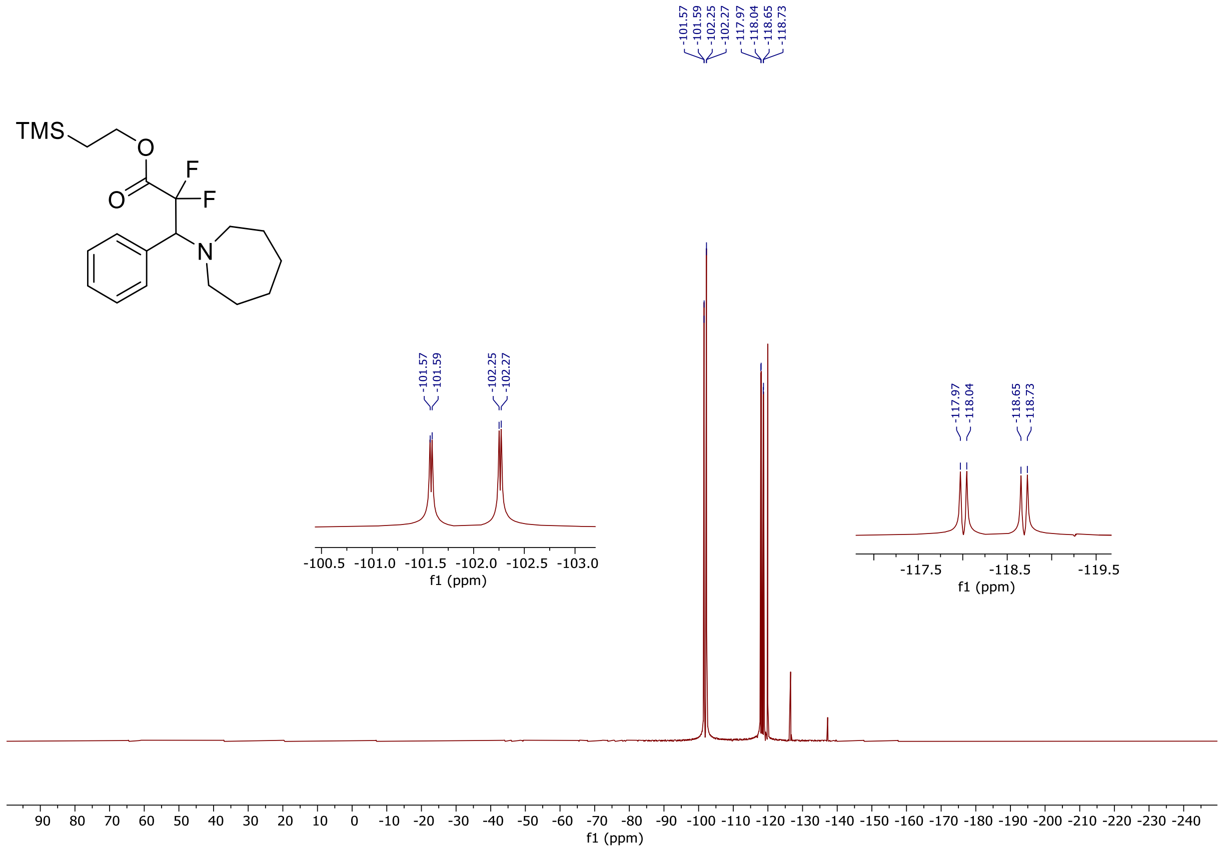

3al – ^{1}H NMR (400 MHz, $CDCl_3$)

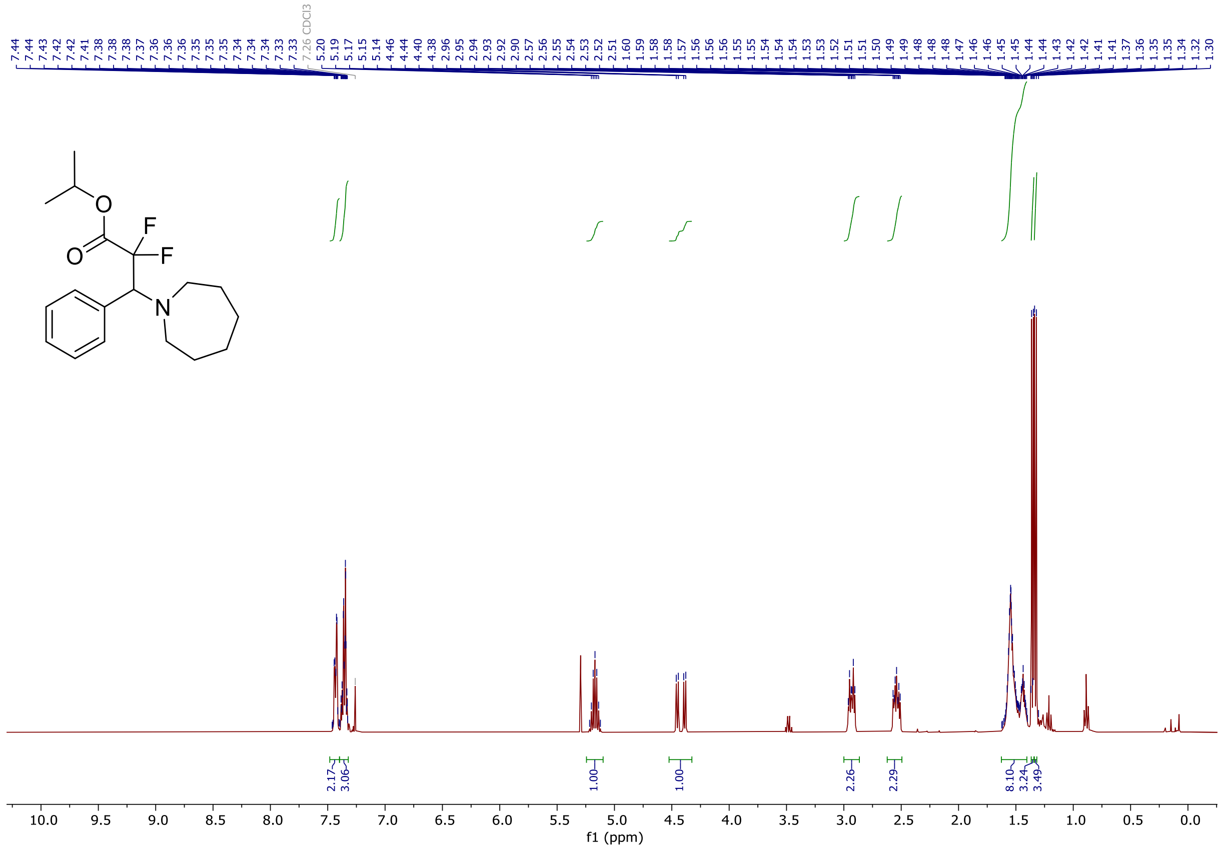

3al – ^{13}C NMR (101 MHz, CDCl$_3$)

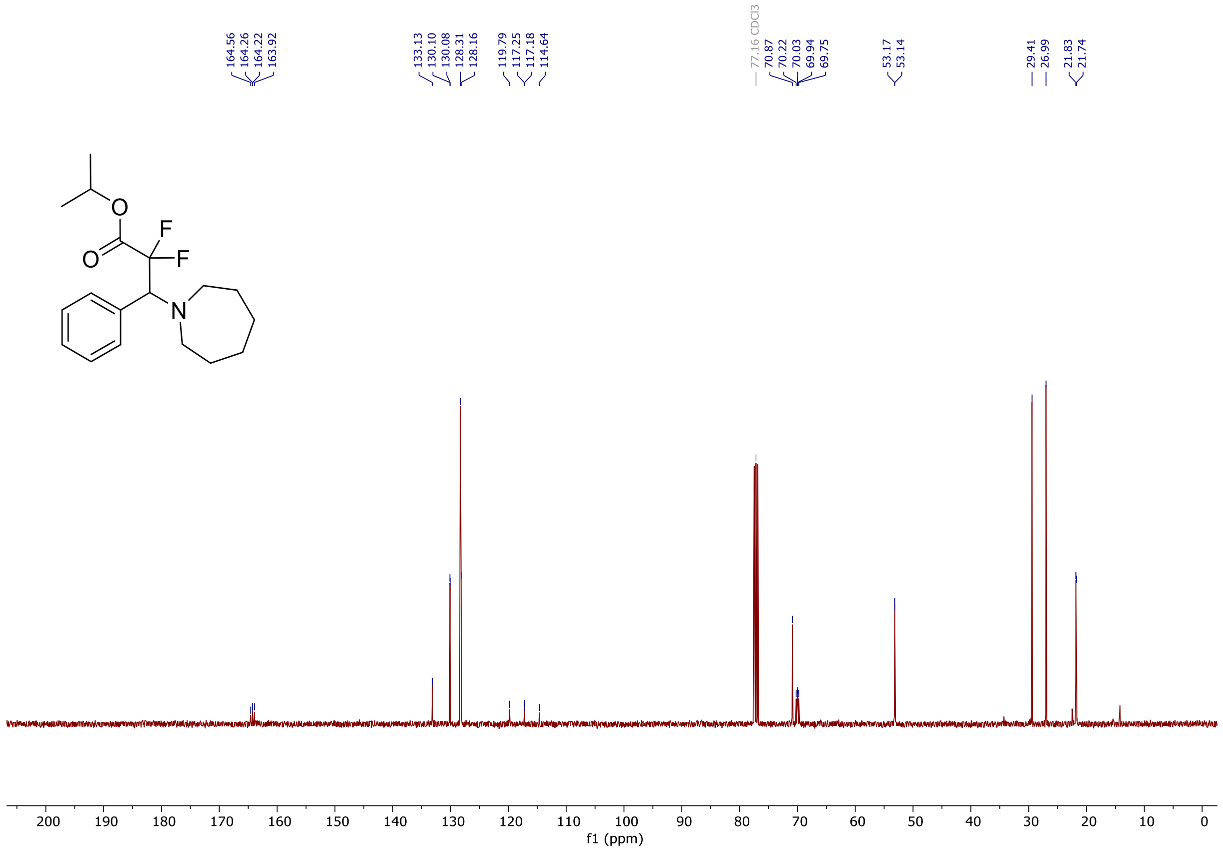

3al – ^{19}F NMR (377 MHz, CDCl$_3$)

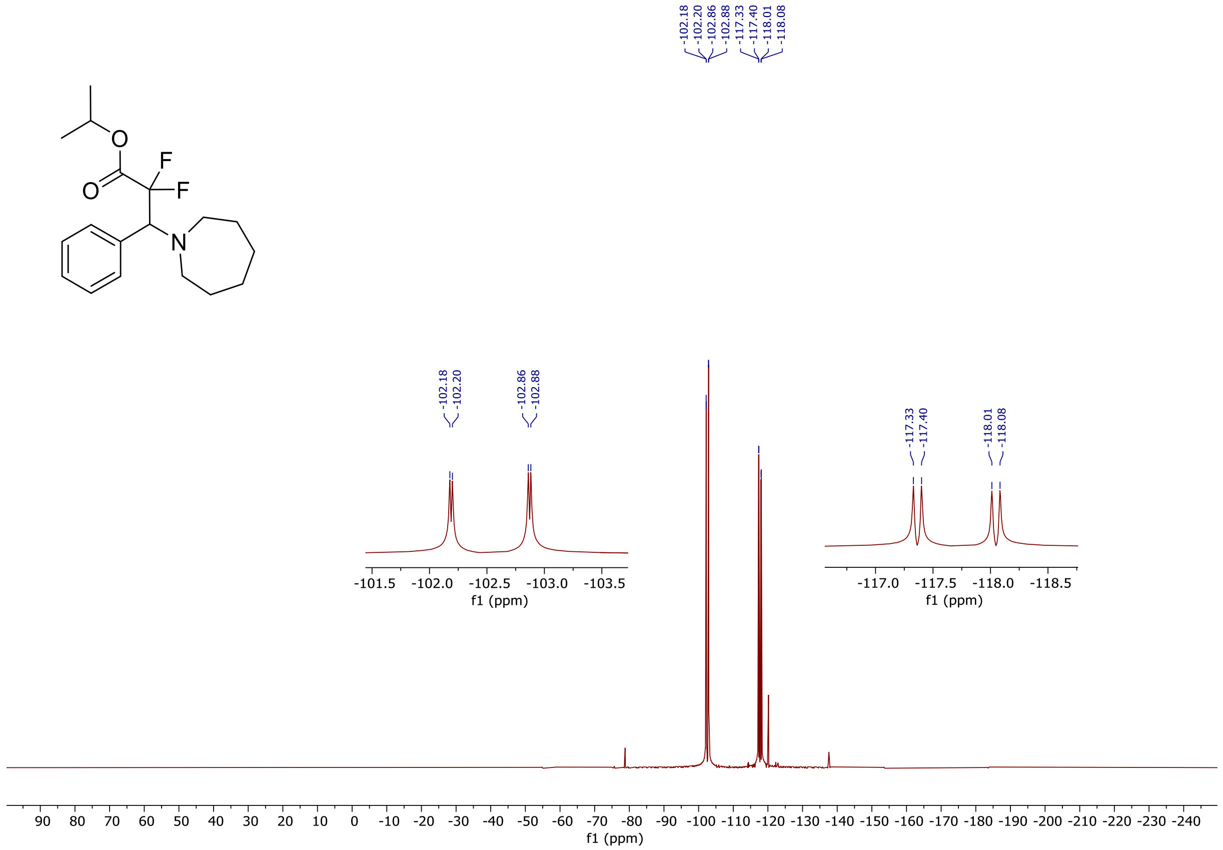

3am – ^{1}H NMR (400 MHz, CDCl$_3$)

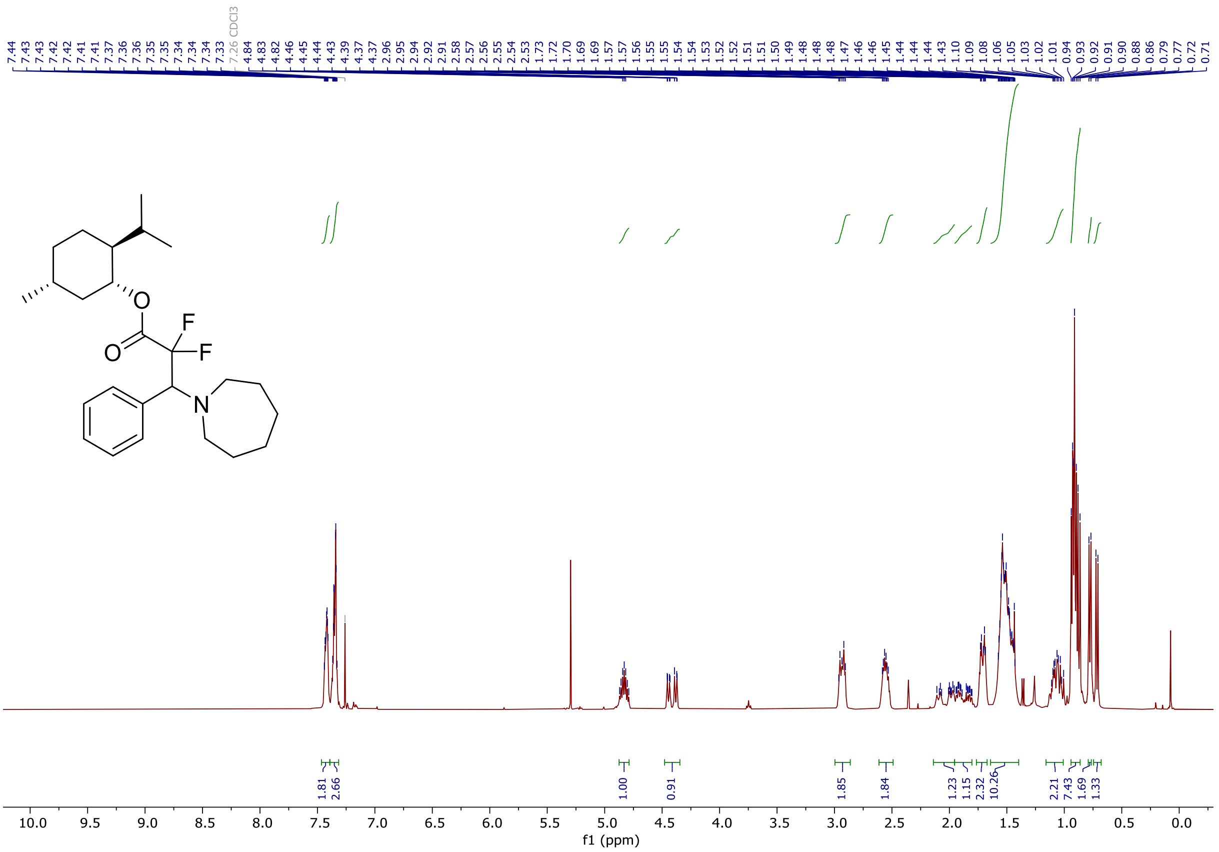

3am – ^{13}C NMR (126 MHz, CDCl$_3$)

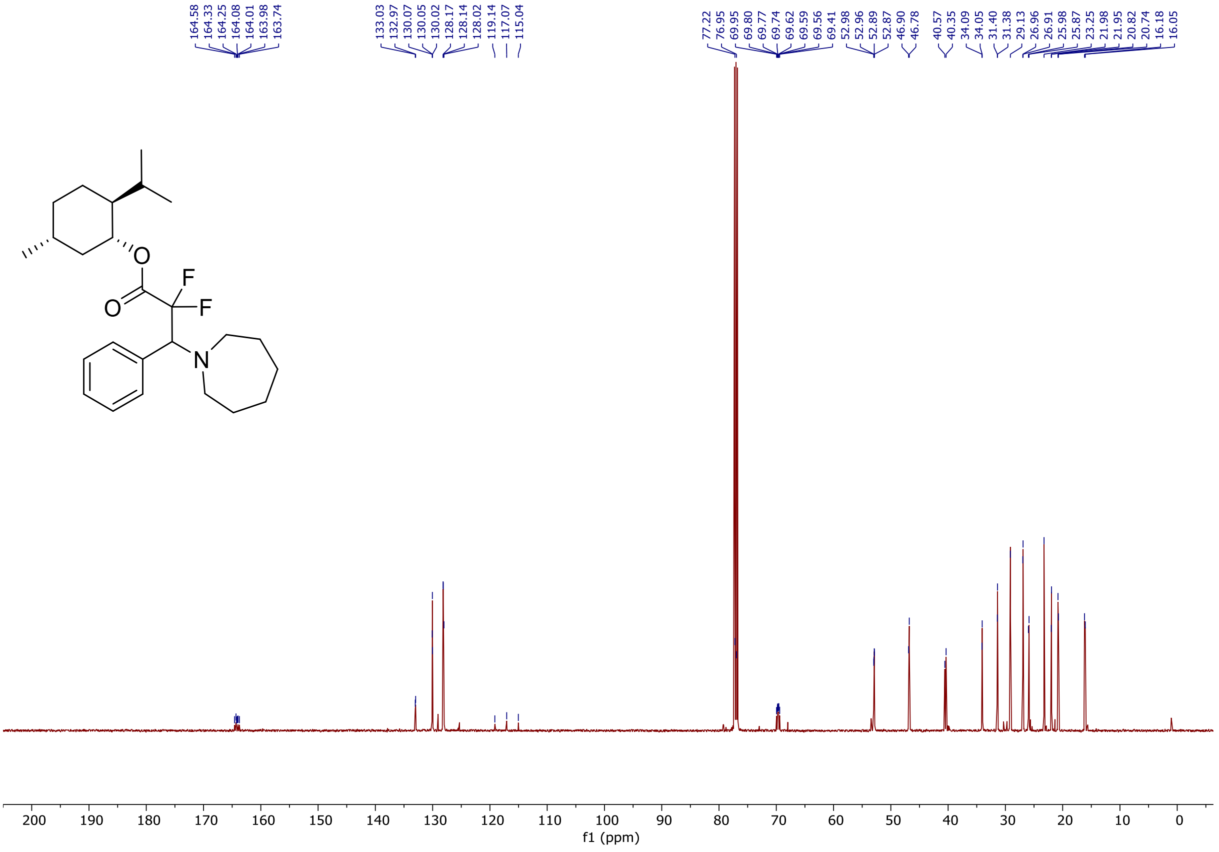

3am – ^{19}F NMR (377 MHz, CDCl$_3$)

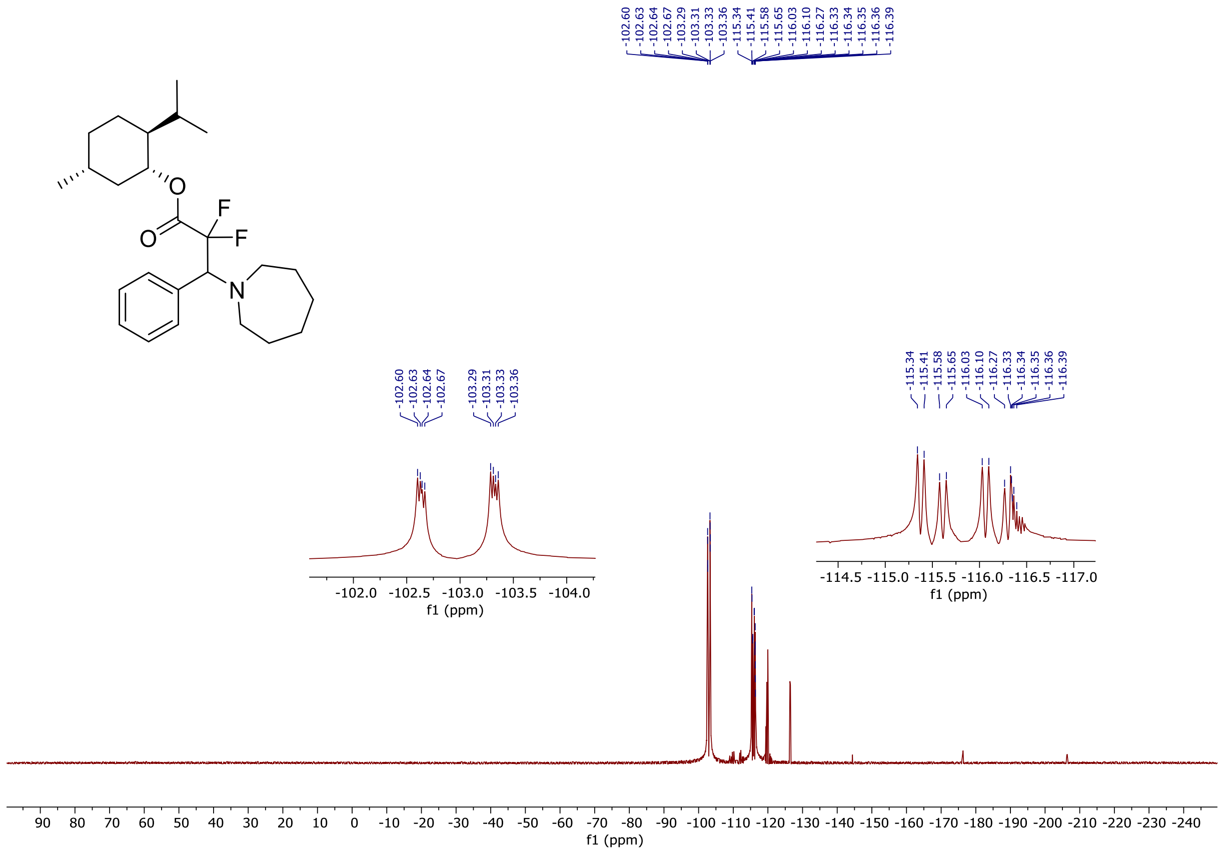

3an – ^{1}H NMR (400 MHz, CDCl$_3$)

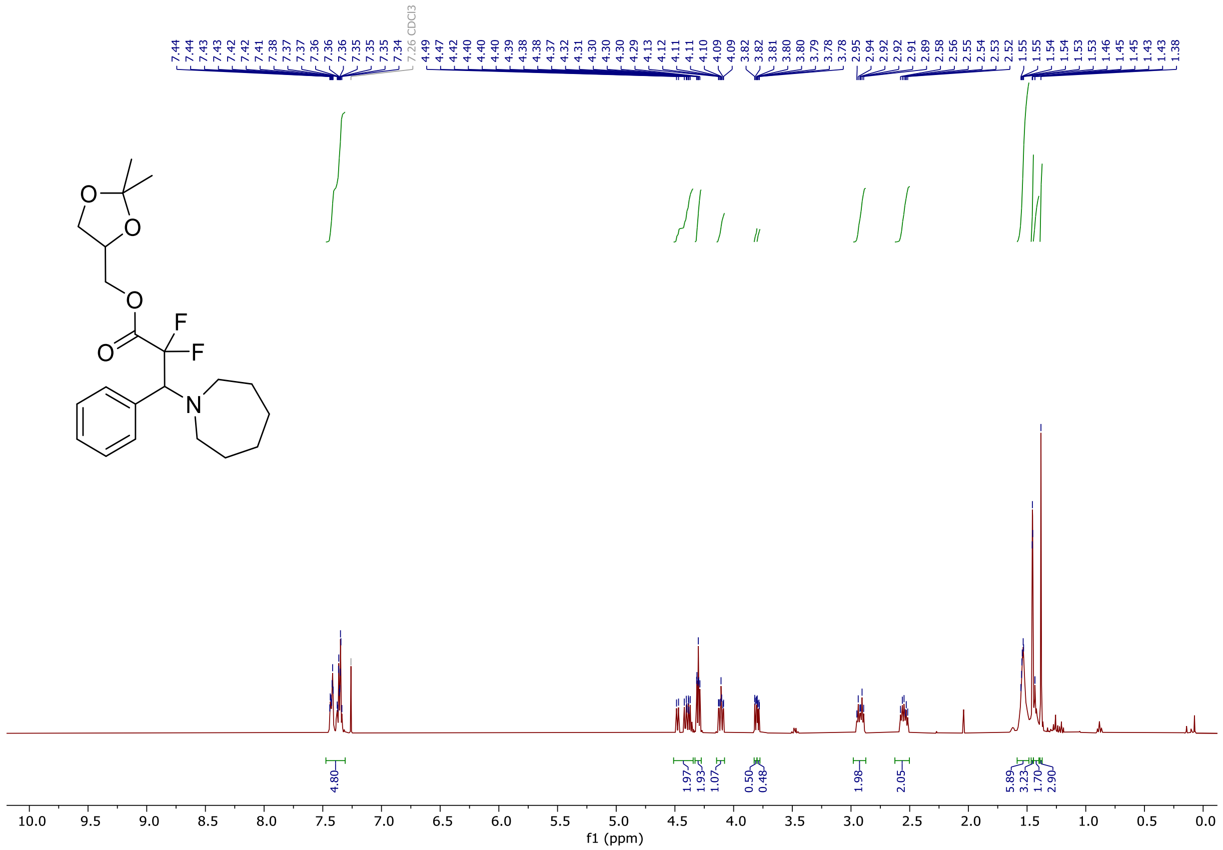

3an – ^{13}C NMR (101 MHz, $CDCl_3$)

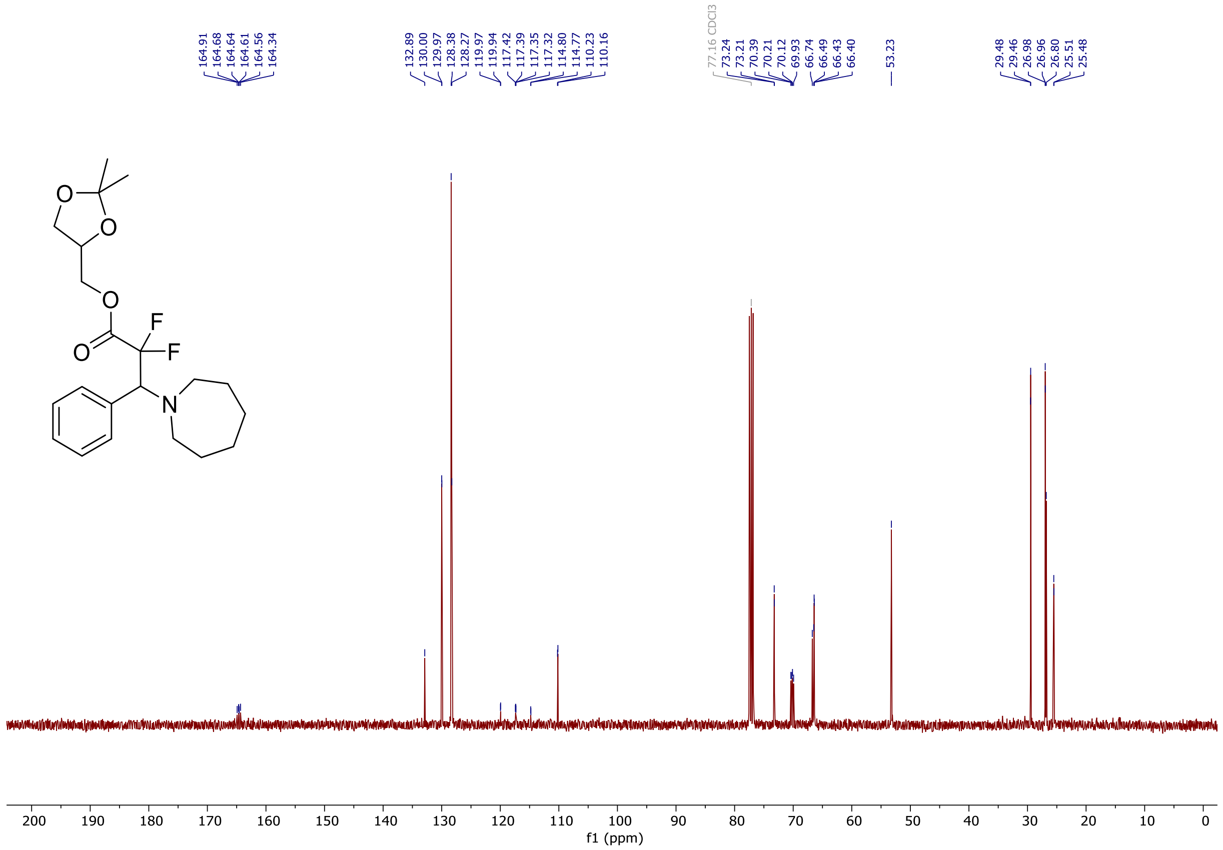

3an – ^{19}F NMR (377 MHz, $CDCl_3$)

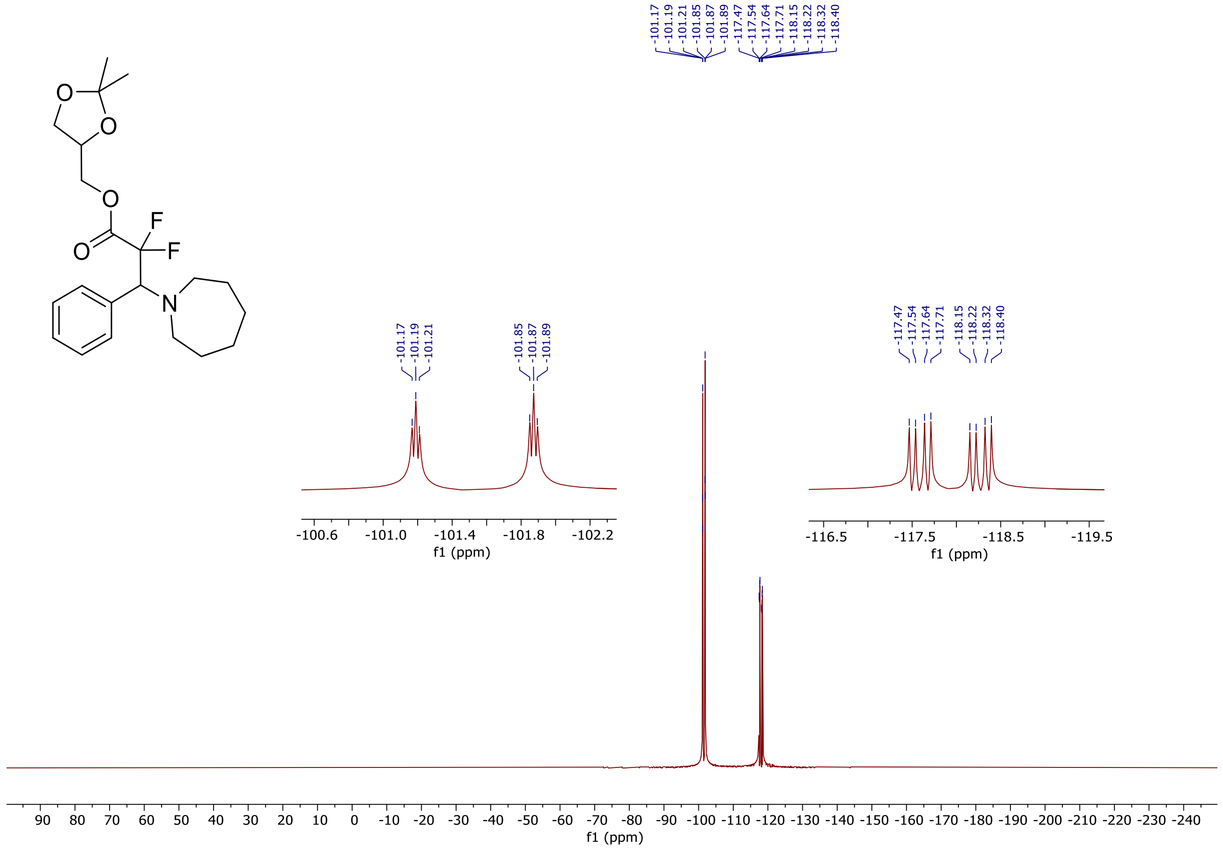

3ao – ^{1}H NMR (400 MHz, $CDCl_3$)

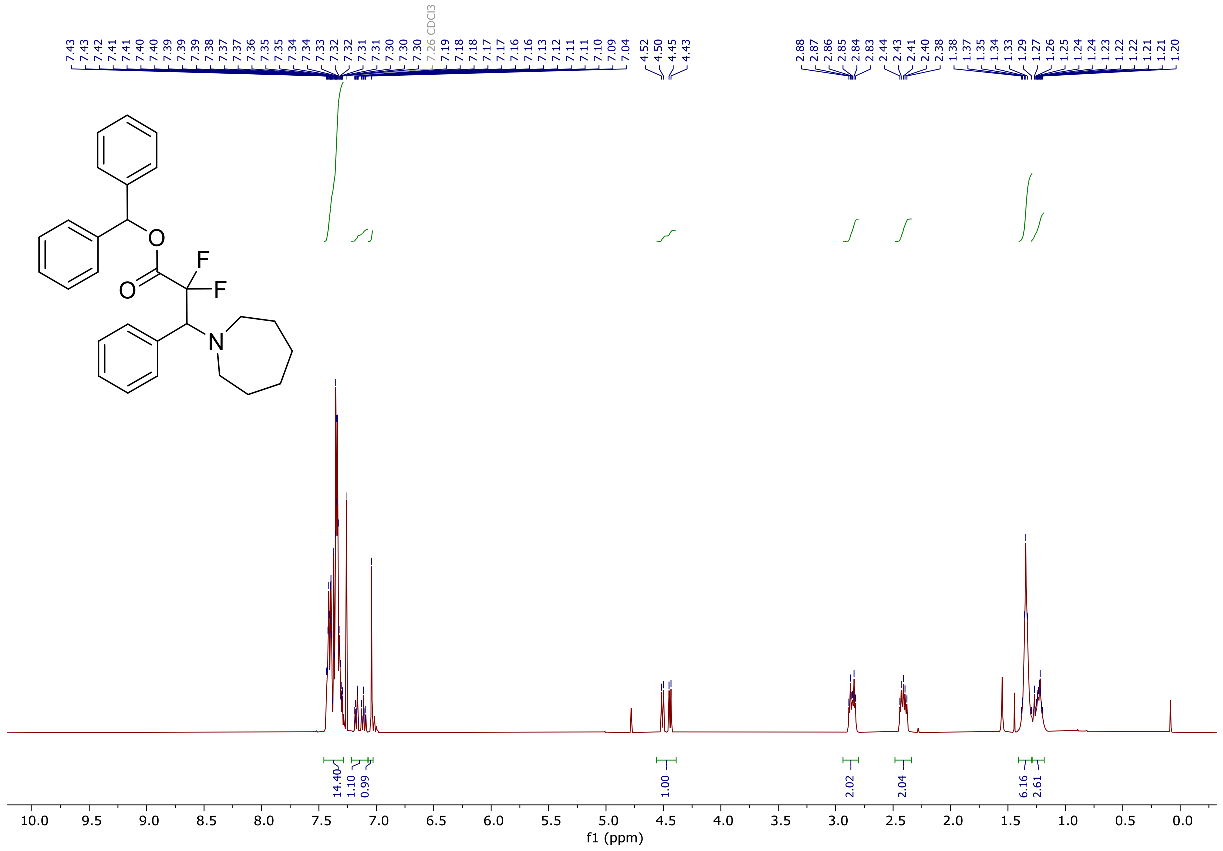

3ao – ^{13}C NMR (101 MHz, $CDCl_3$)

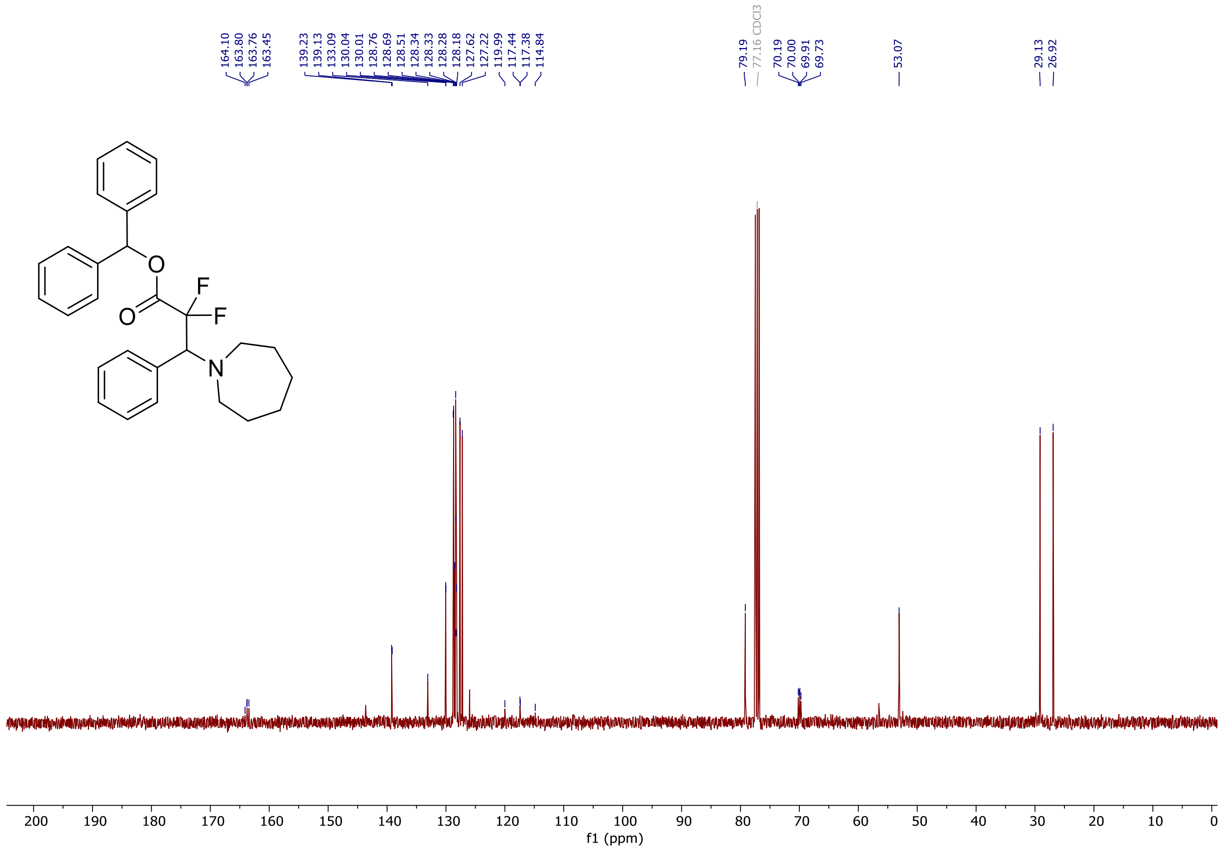

3ao – ^{19}F NMR (377 MHz, CDCl$_3$)

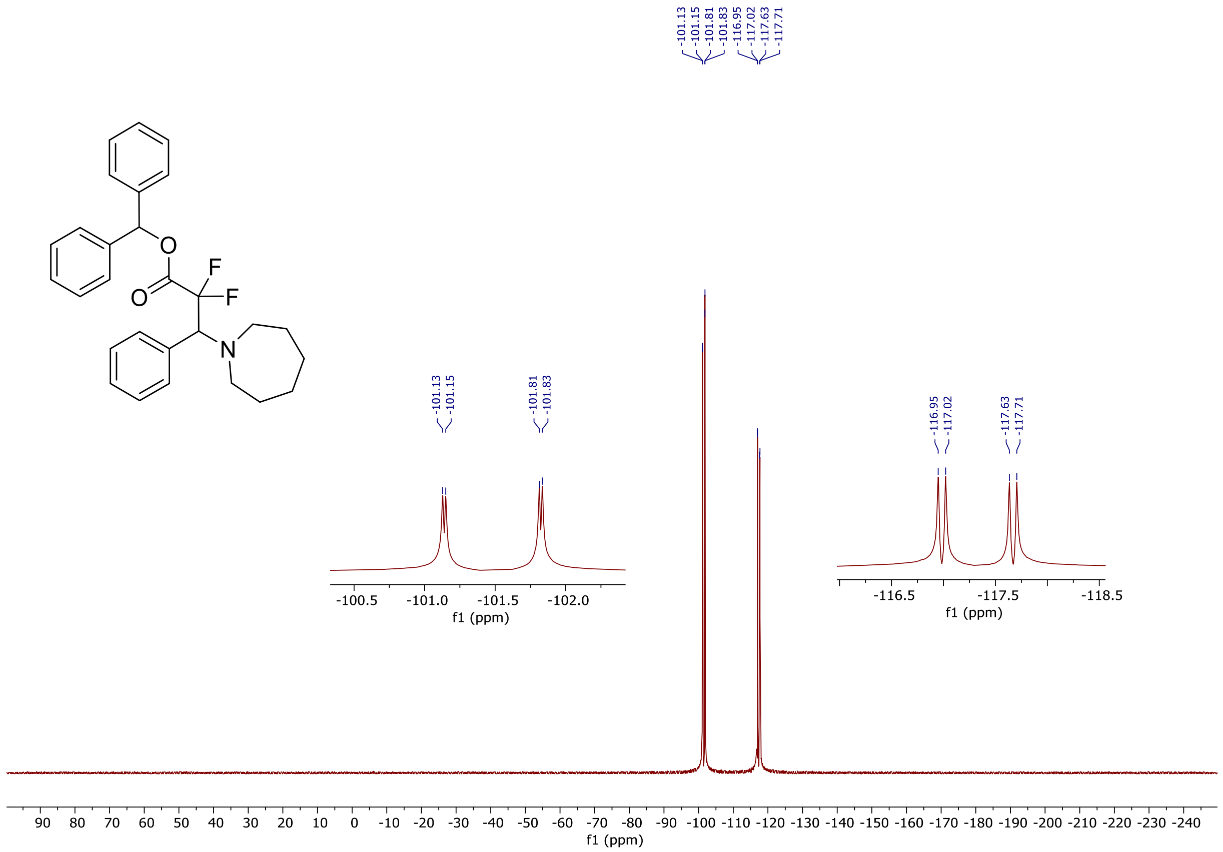

3ap – ^{1}H NMR (400 MHz, CDCl$_3$)

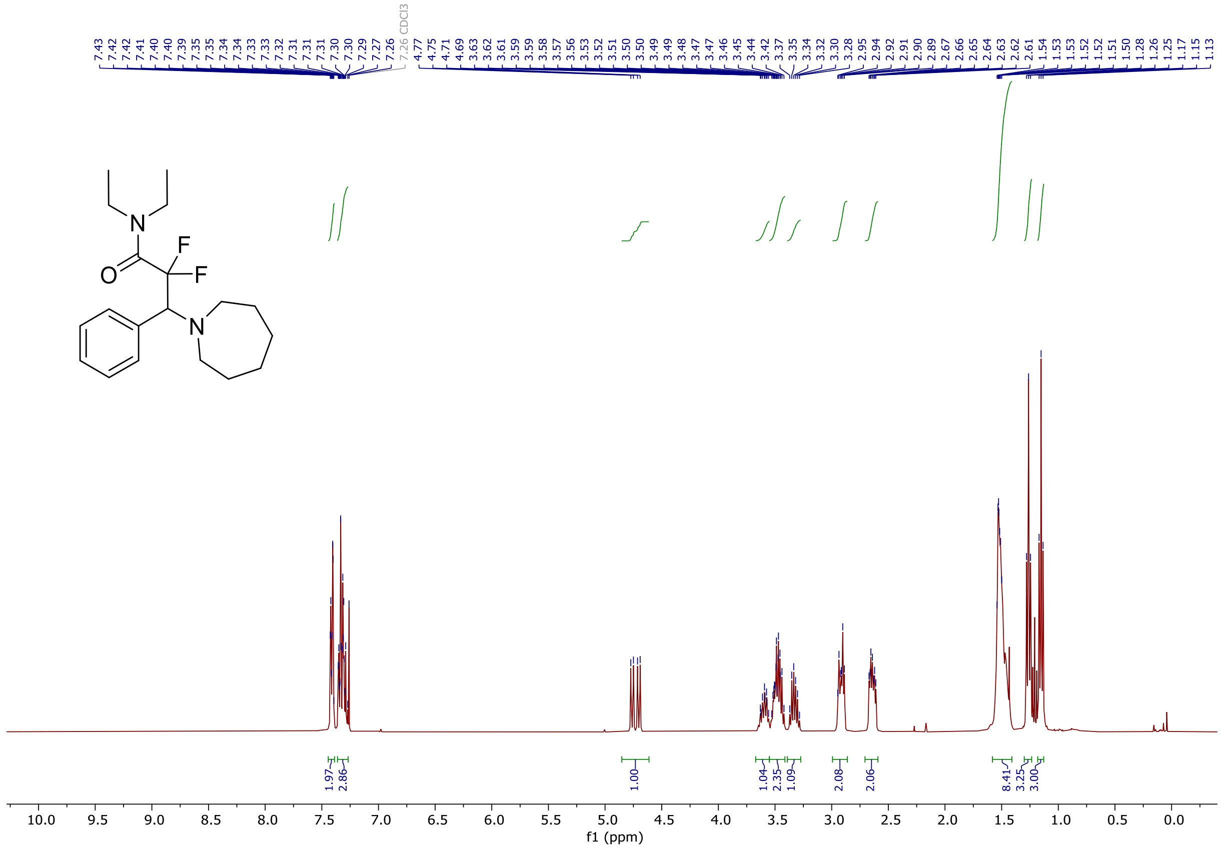

3ap – ^{13}C NMR (126 MHz, $CDCl_3$)

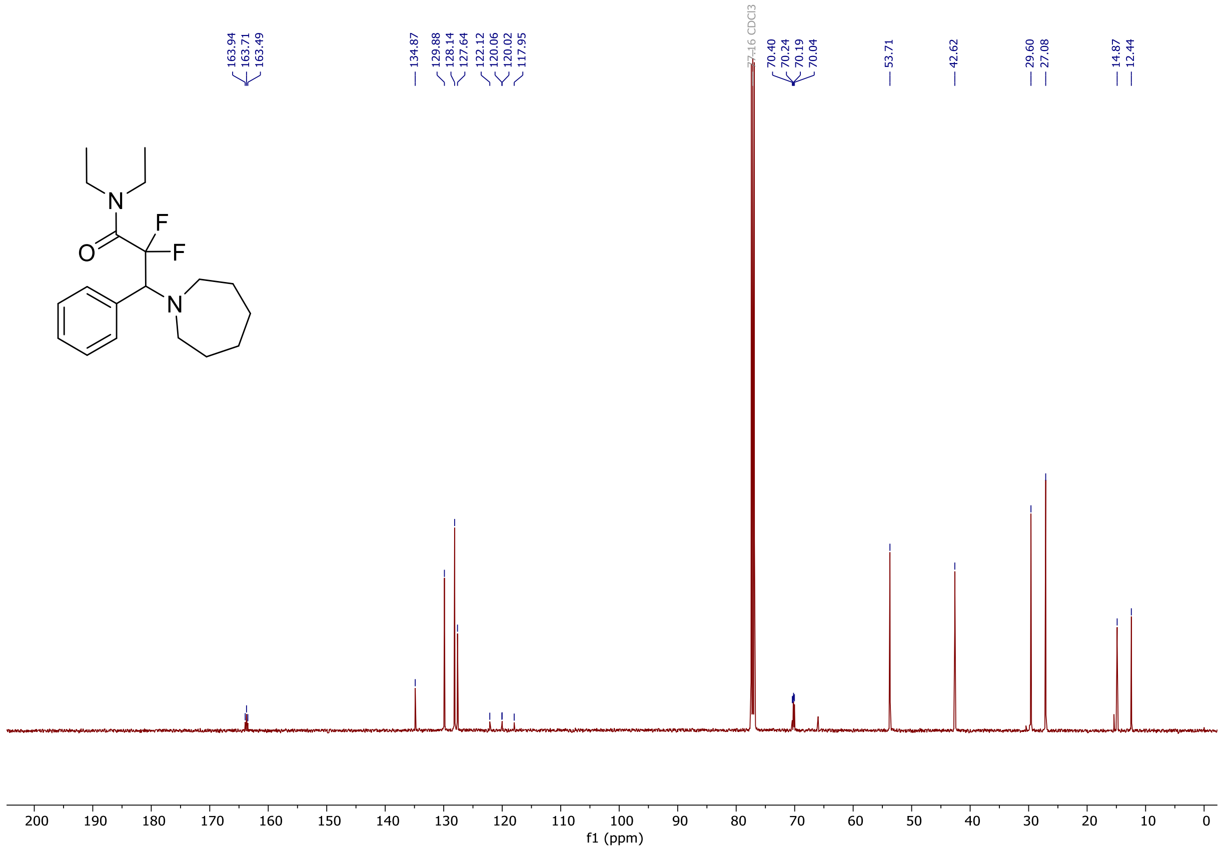

3ap – ^{19}F NMR (377 MHz, $CDCl_3$)

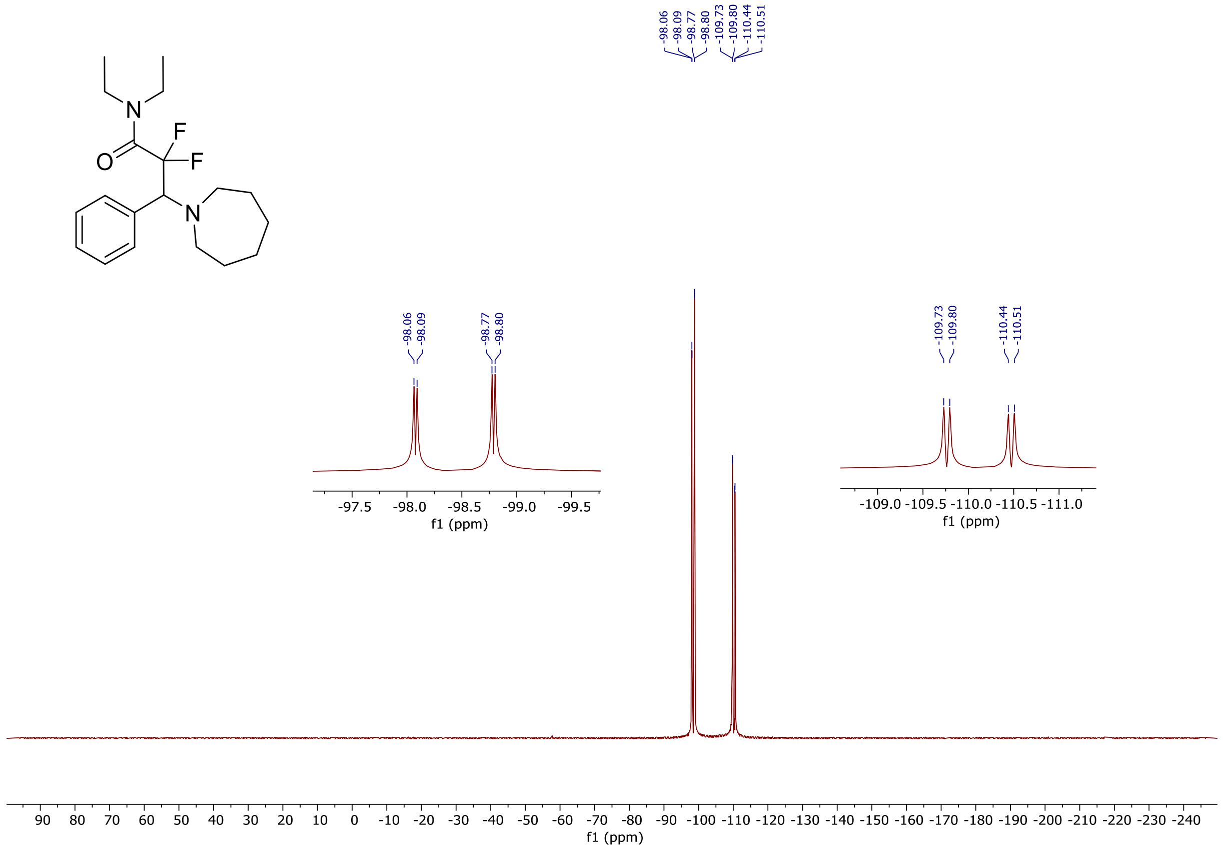

3aq – ^{1}H NMR (400 MHz, CDCl$_3$)

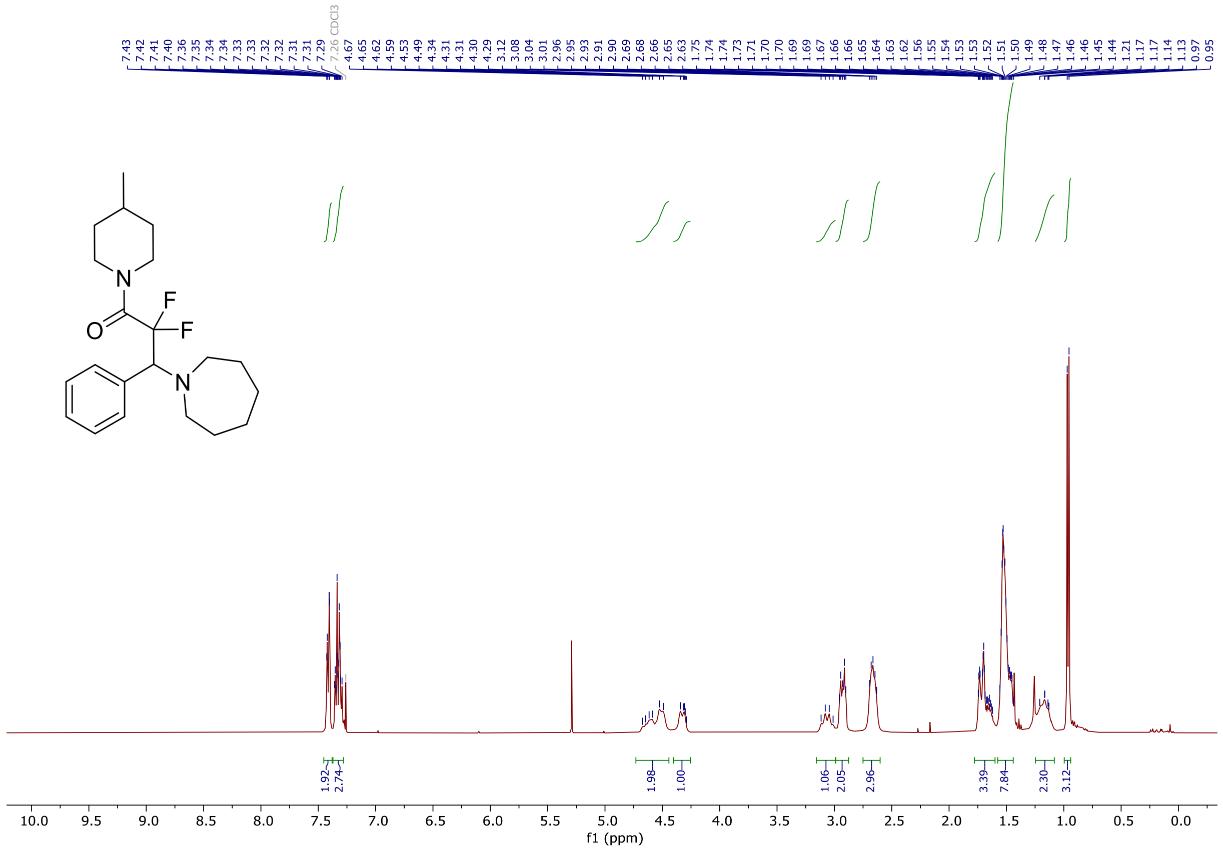

3aq – ^{13}C NMR (126 MHz, CDCl$_3$)

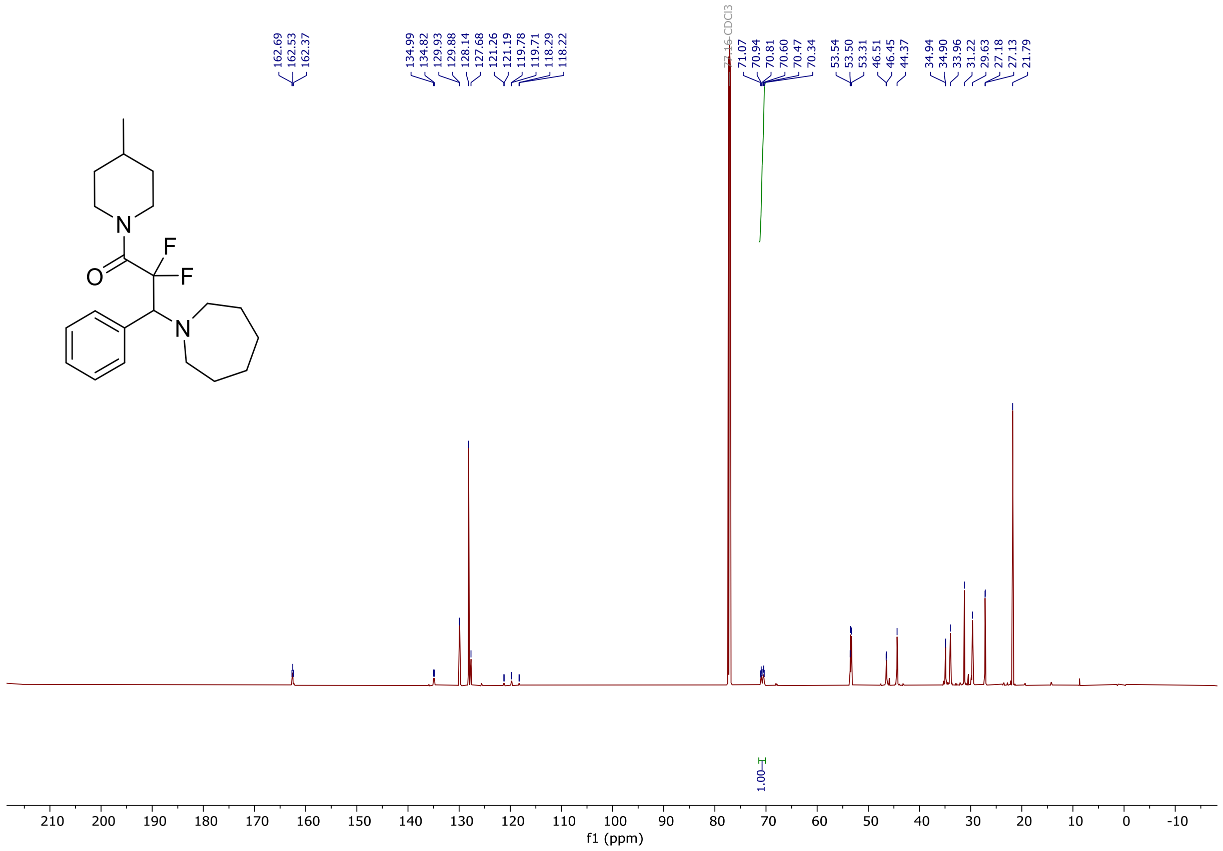

3aq – ^{19}F NMR (377 MHz, CDCl$_3$)

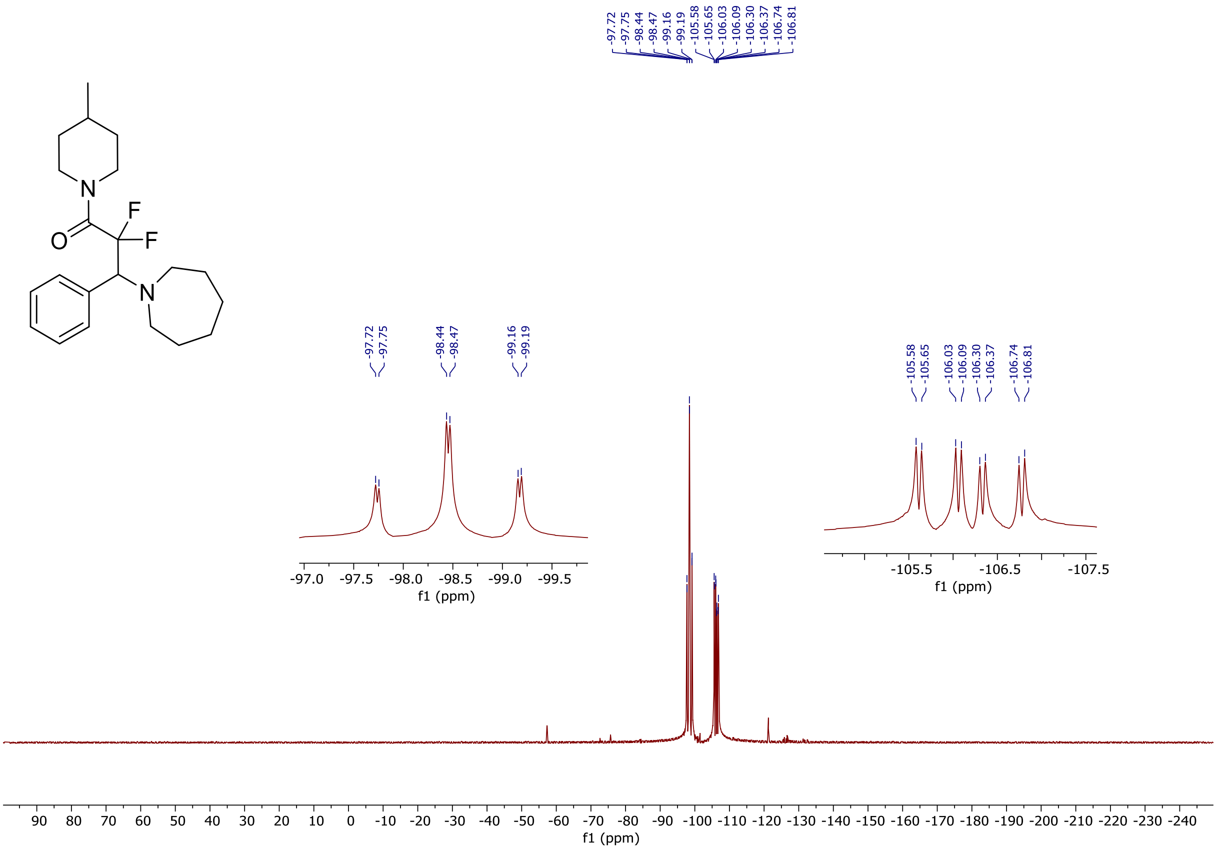

3ar – ^{1}H NMR (400 MHz, CDCl$_3$)

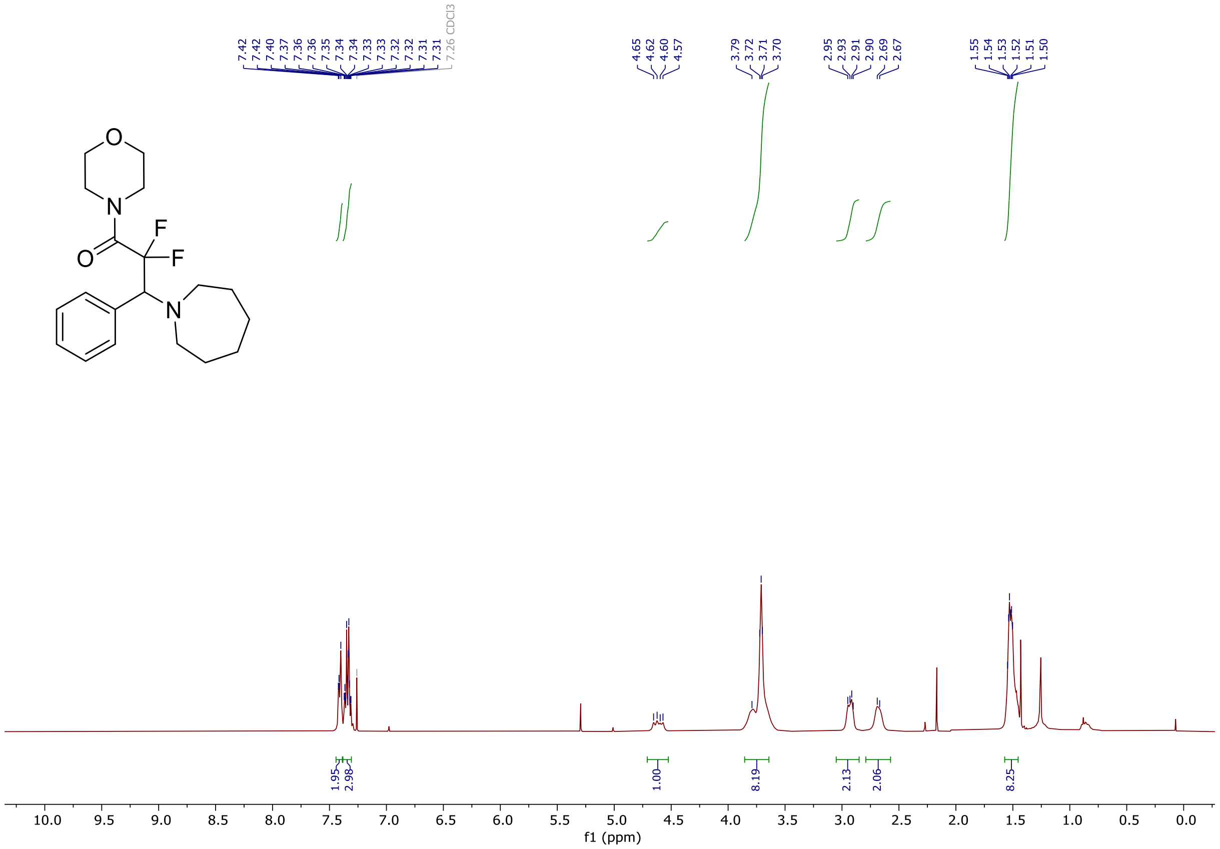

3ar – ^{13}C NMR (126 MHz, $CDCl_3$)

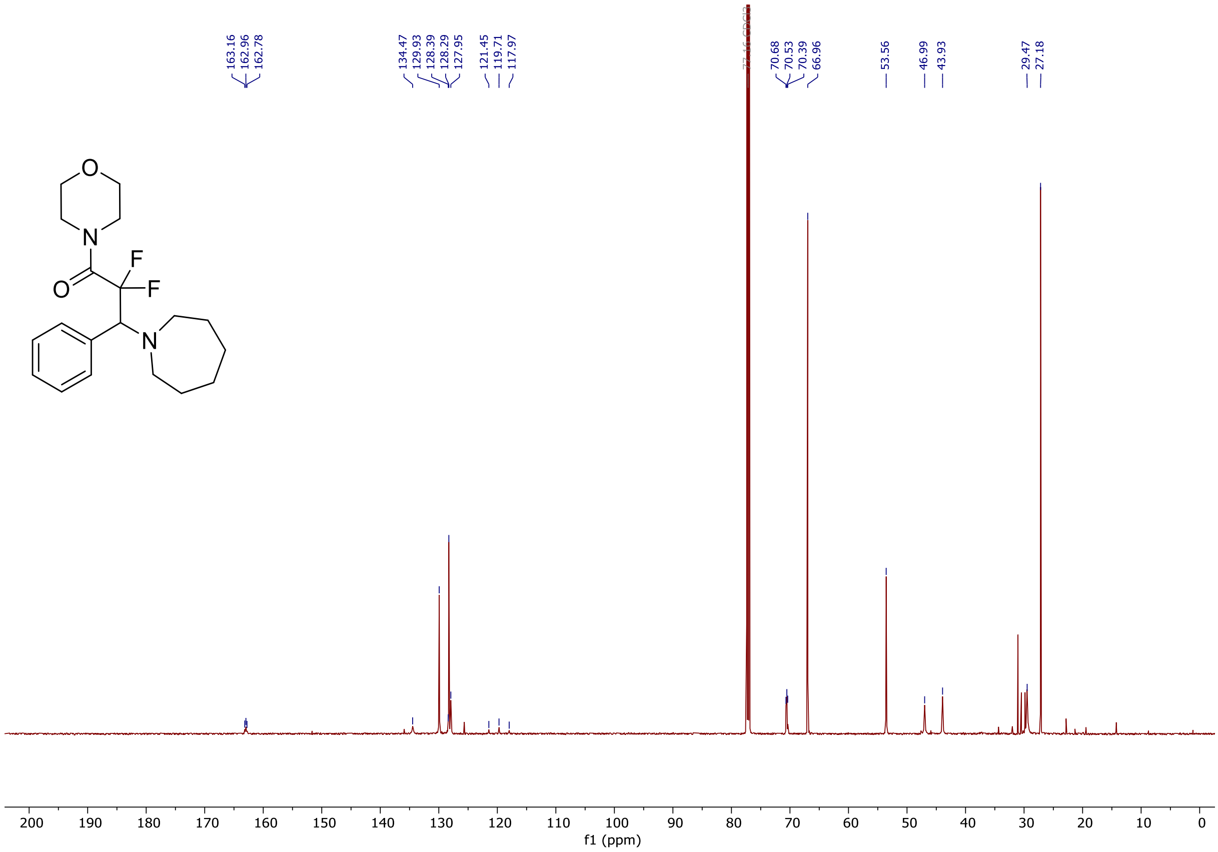

3ar – ^{19}F NMR (377 MHz, $CDCl_3$)

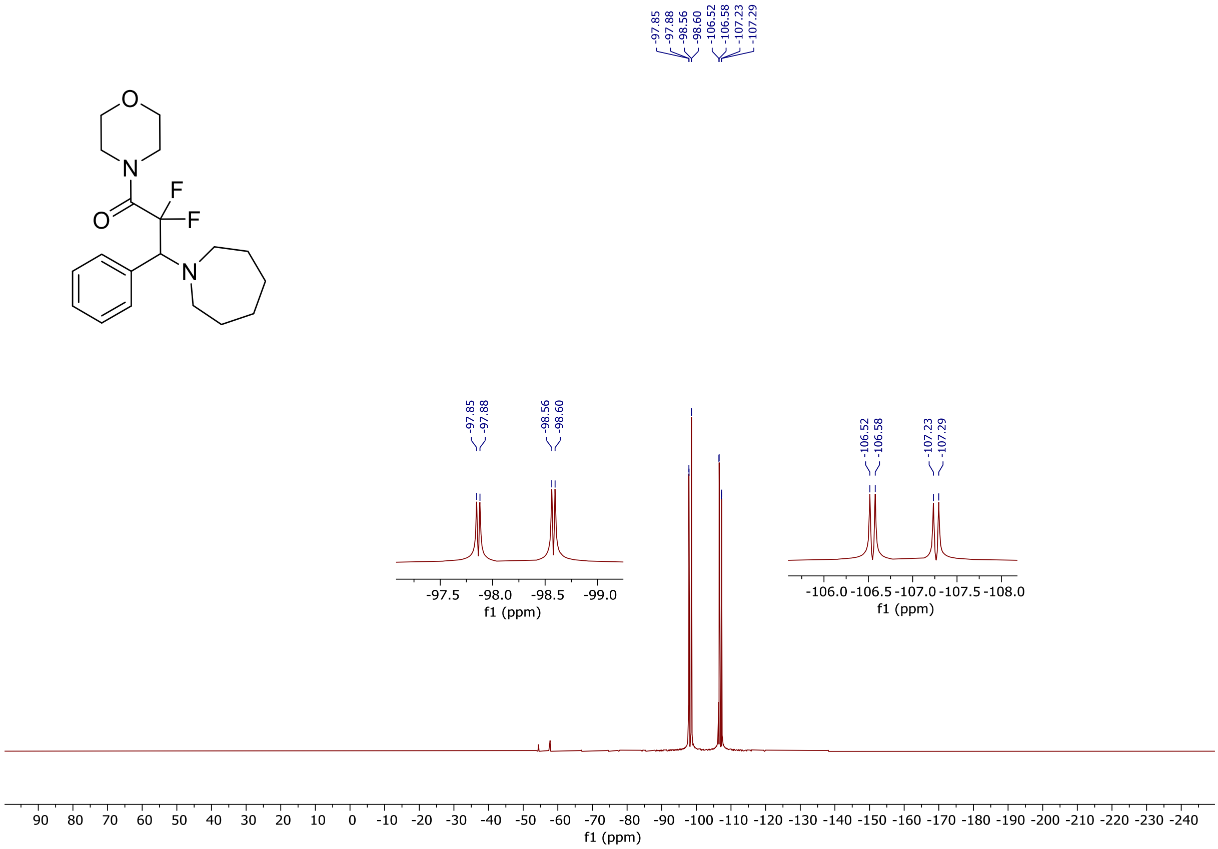

3as – ^{1}H NMR (400 MHz, CDCl$_3$)

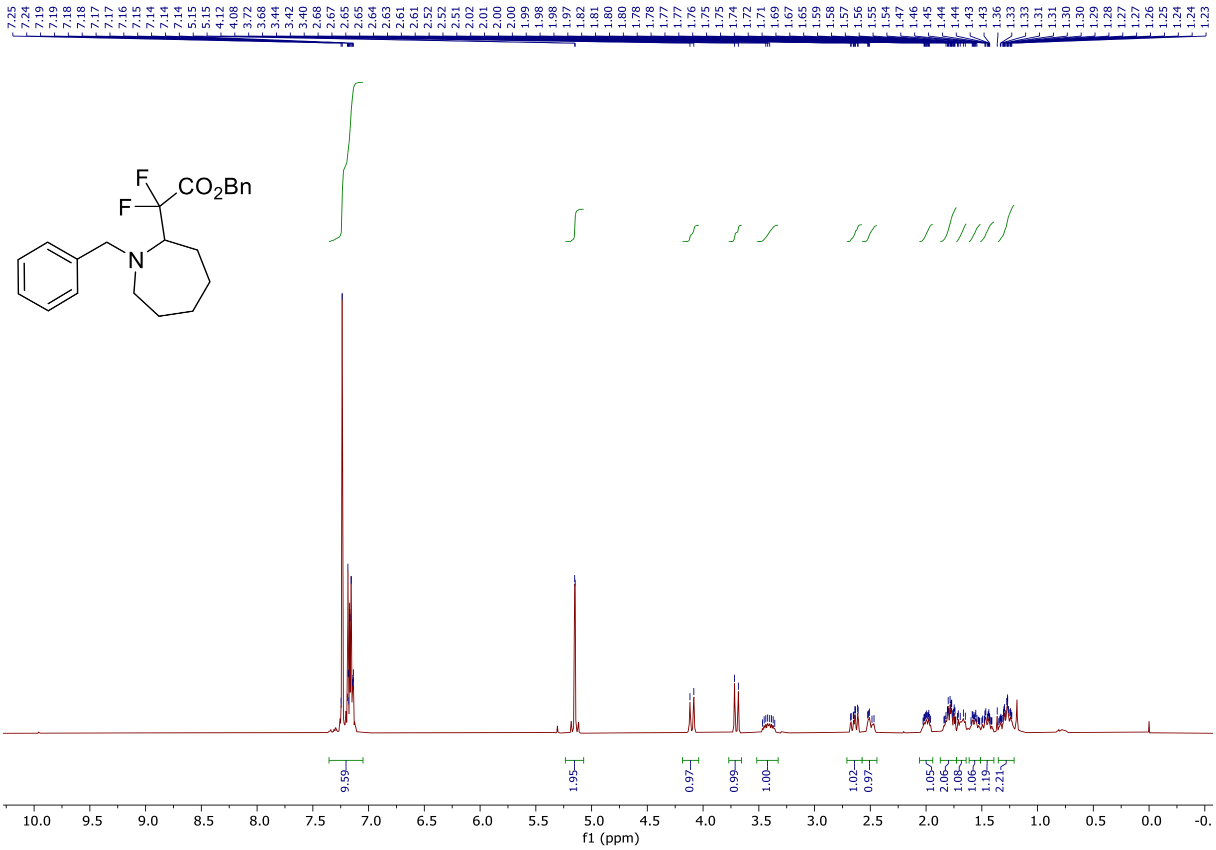

3as – ^{13}C NMR (126 MHz, CDCl$_3$)

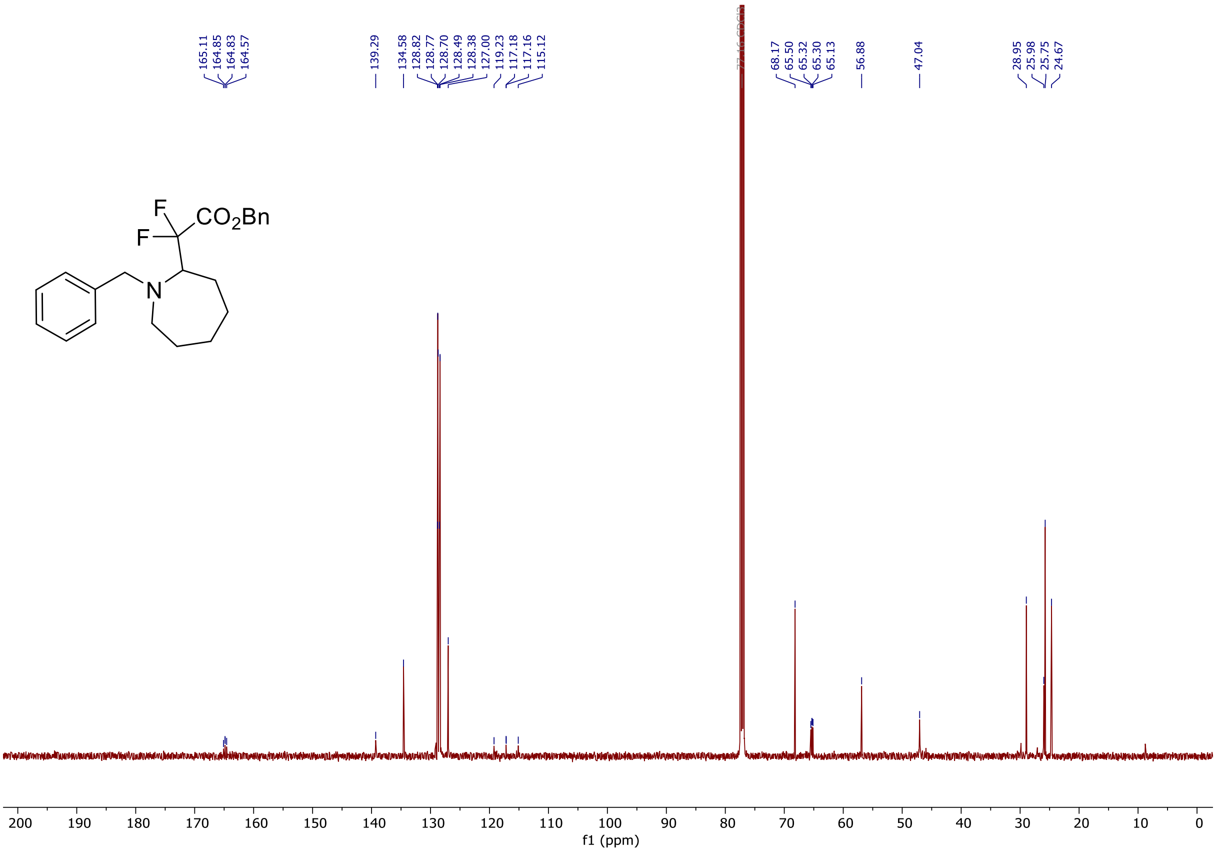

3as – ^{19}F NMR (377 MHz, $CDCl_3$)

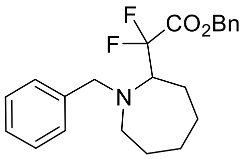

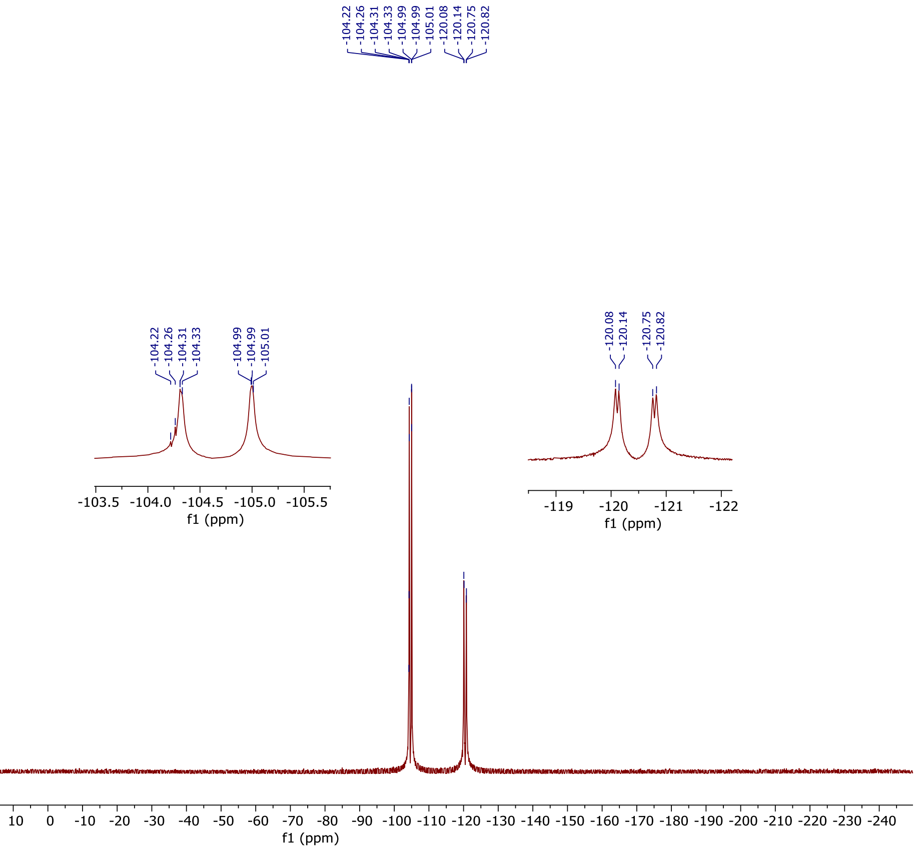

3at – ^{1}H NMR (400 MHz, $CDCl_3$)

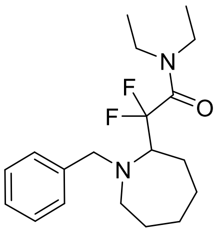

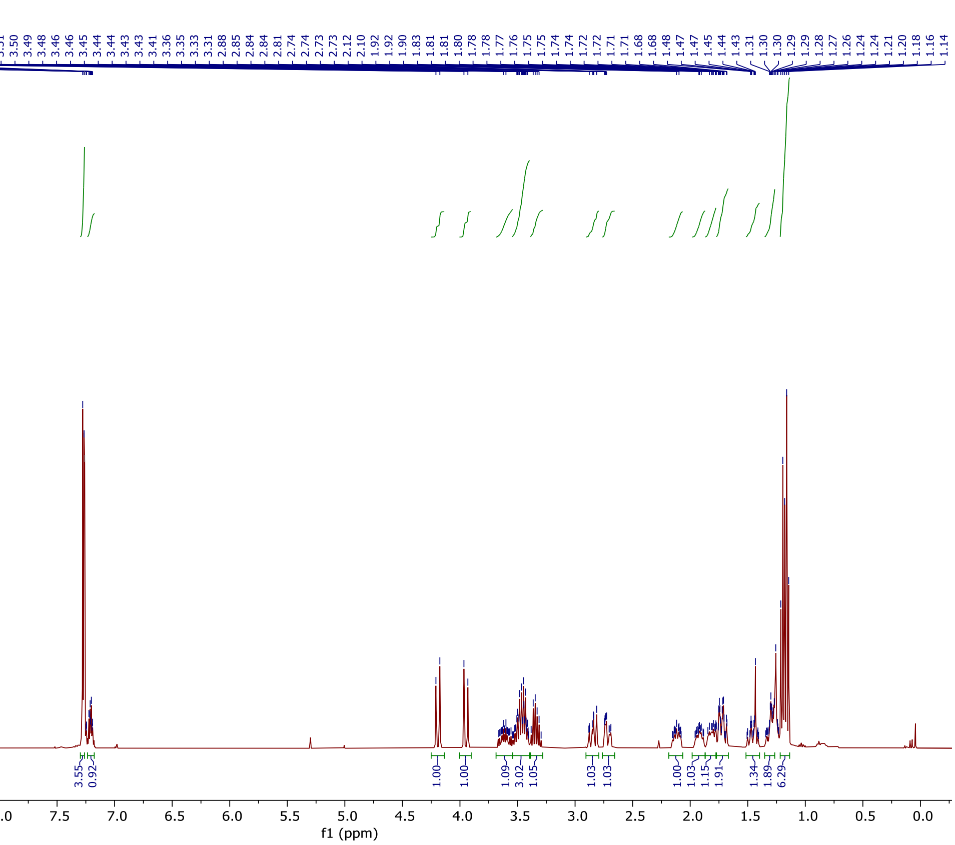

3at – ^{13}C NMR (126 MHz, $CDCl_3$)

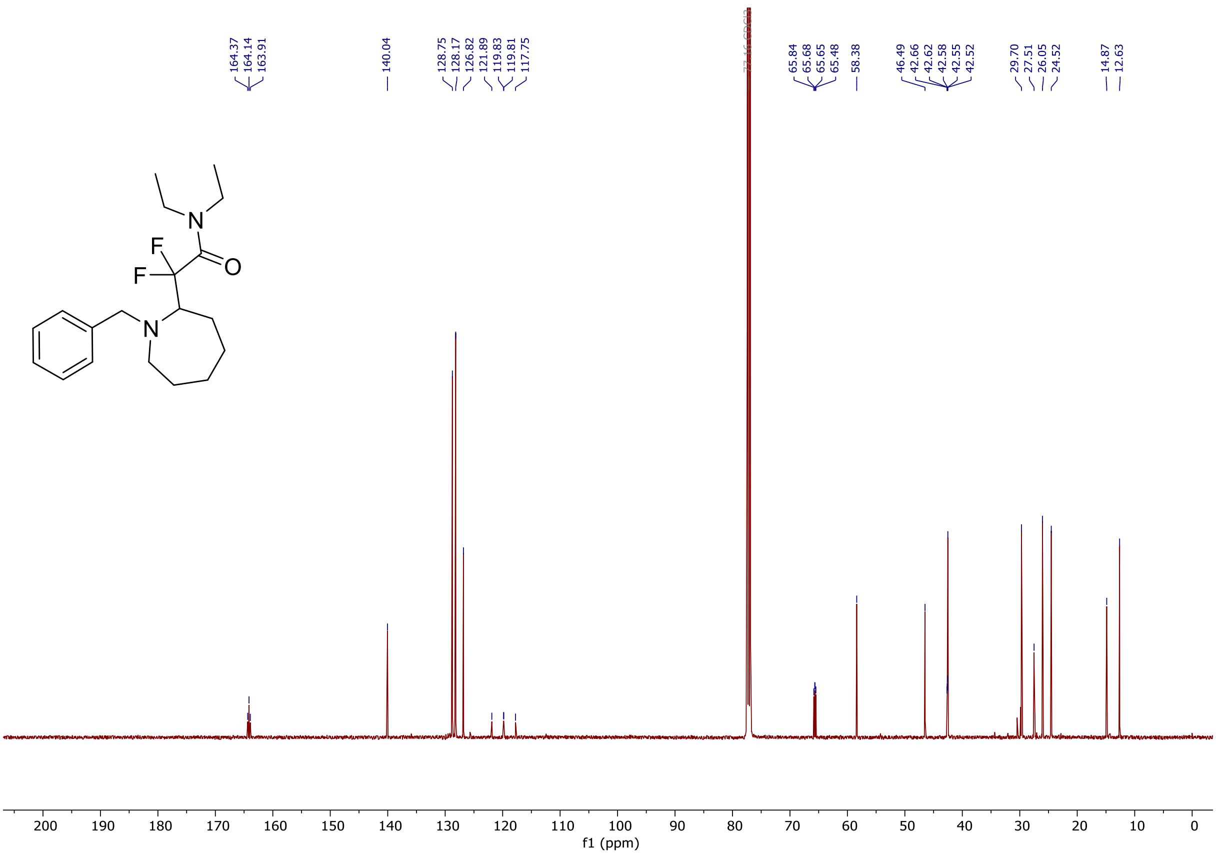

3at – ^{19}F NMR (377 MHz, $CDCl_3$)

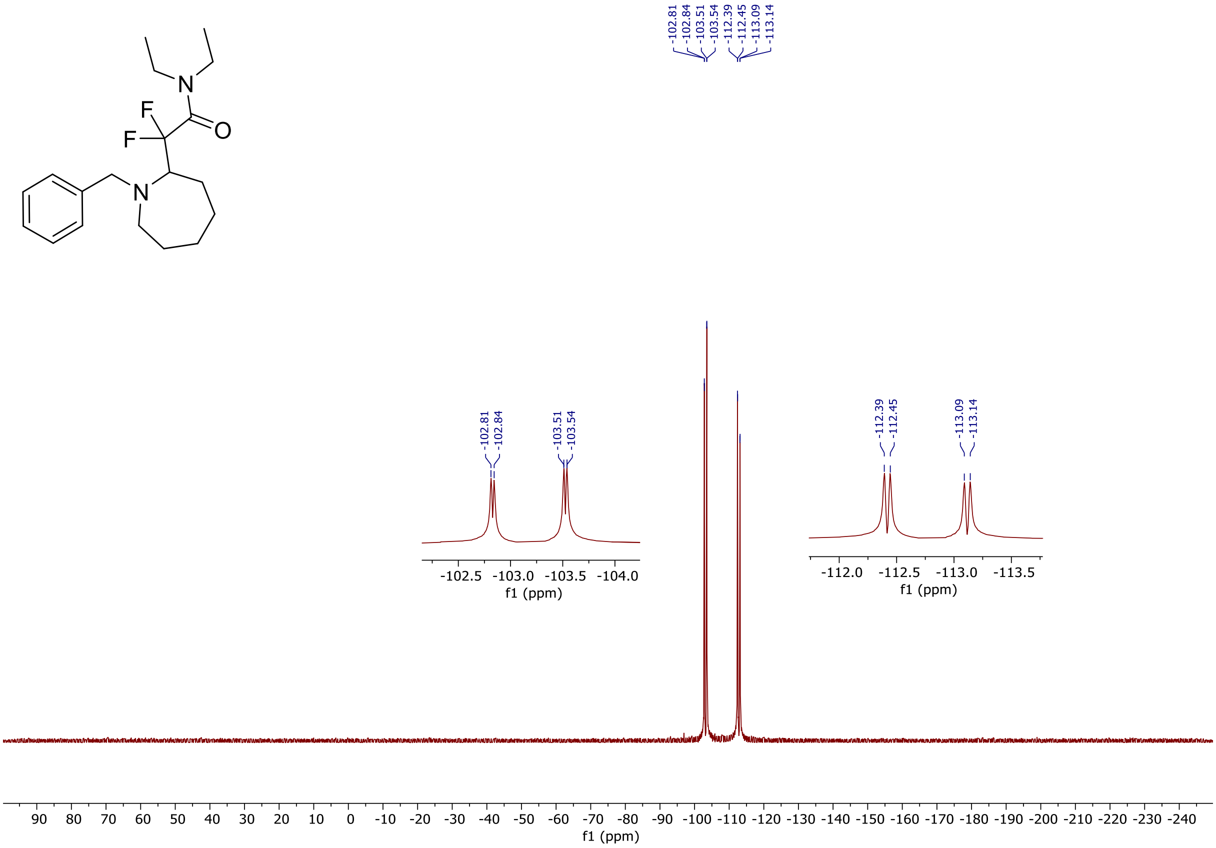

6 – ^{1}H NMR (400 MHz, $CDCl_3$)

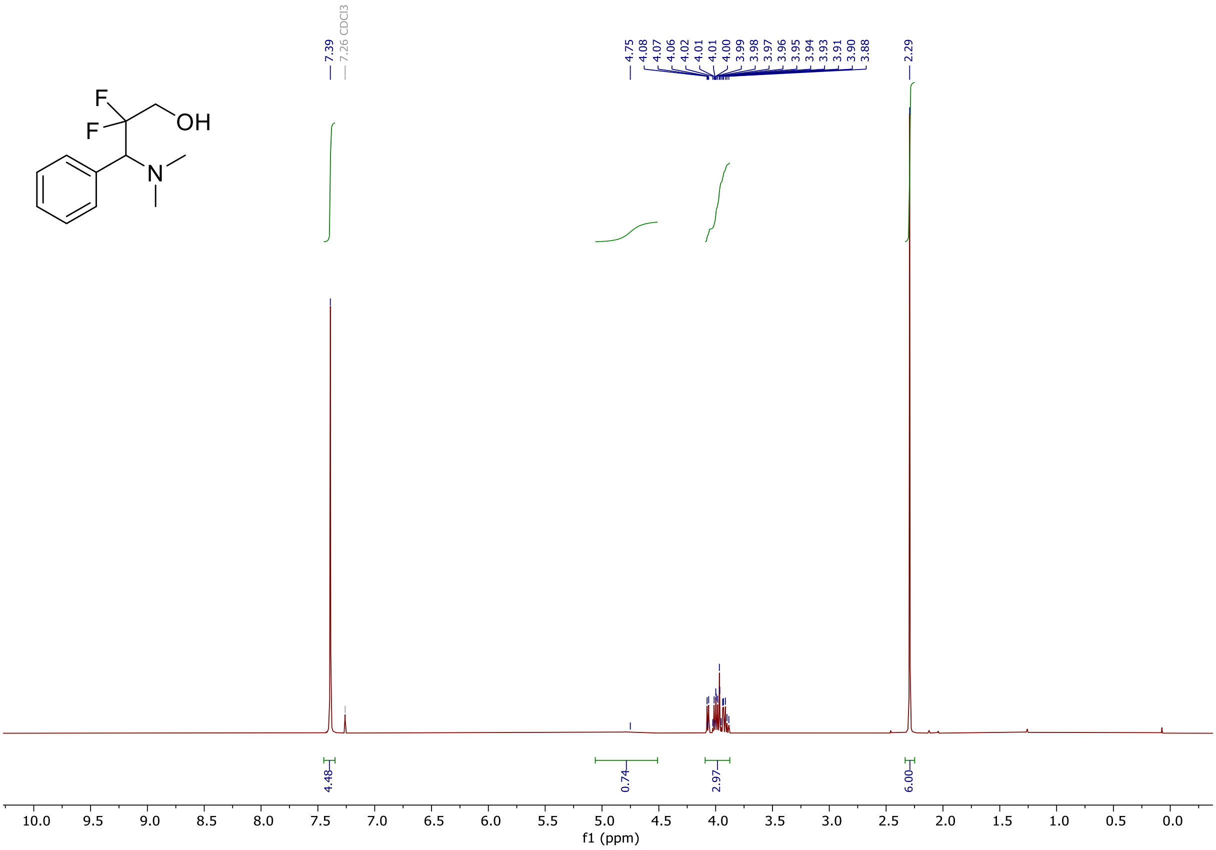

6 – ^{13}C NMR (126 MHz, $CDCl_3$)

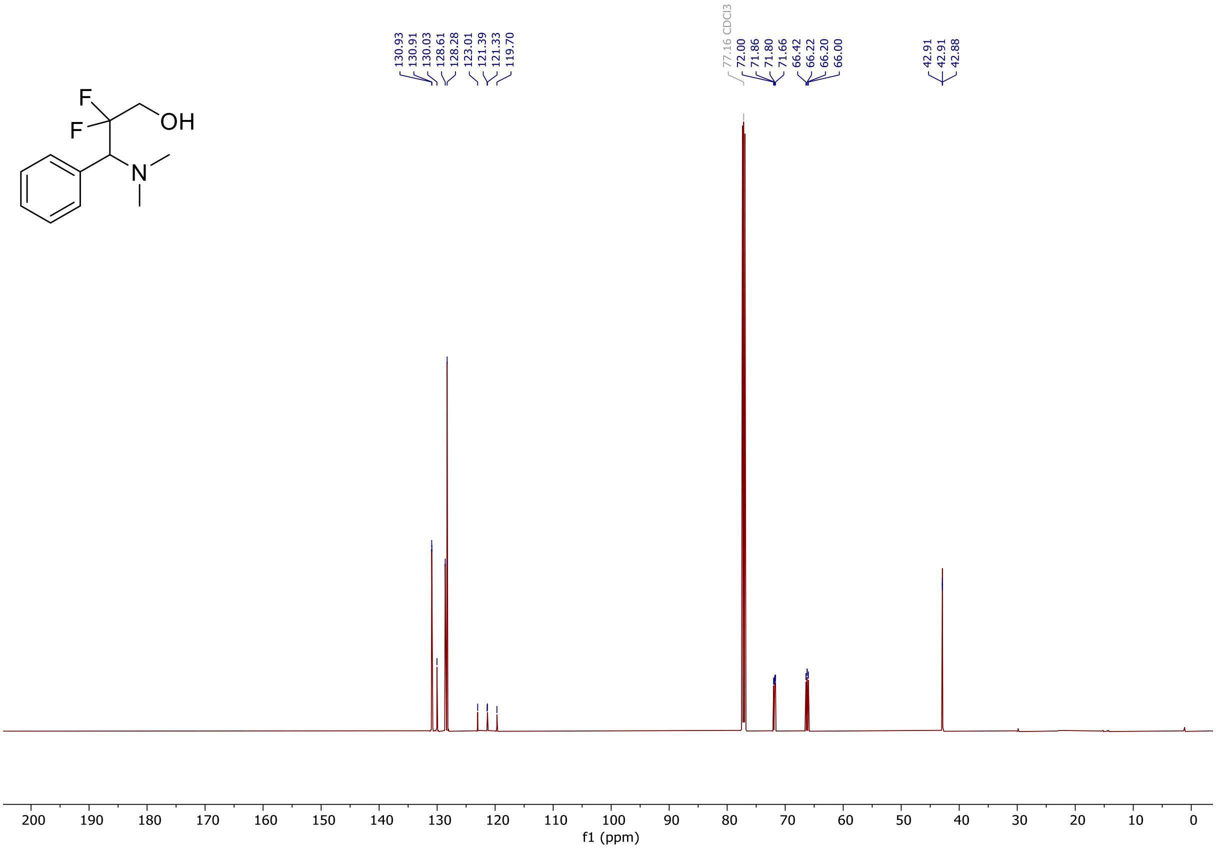

6 – ^{19}F NMR (377 MHz, $CDCl_3$)

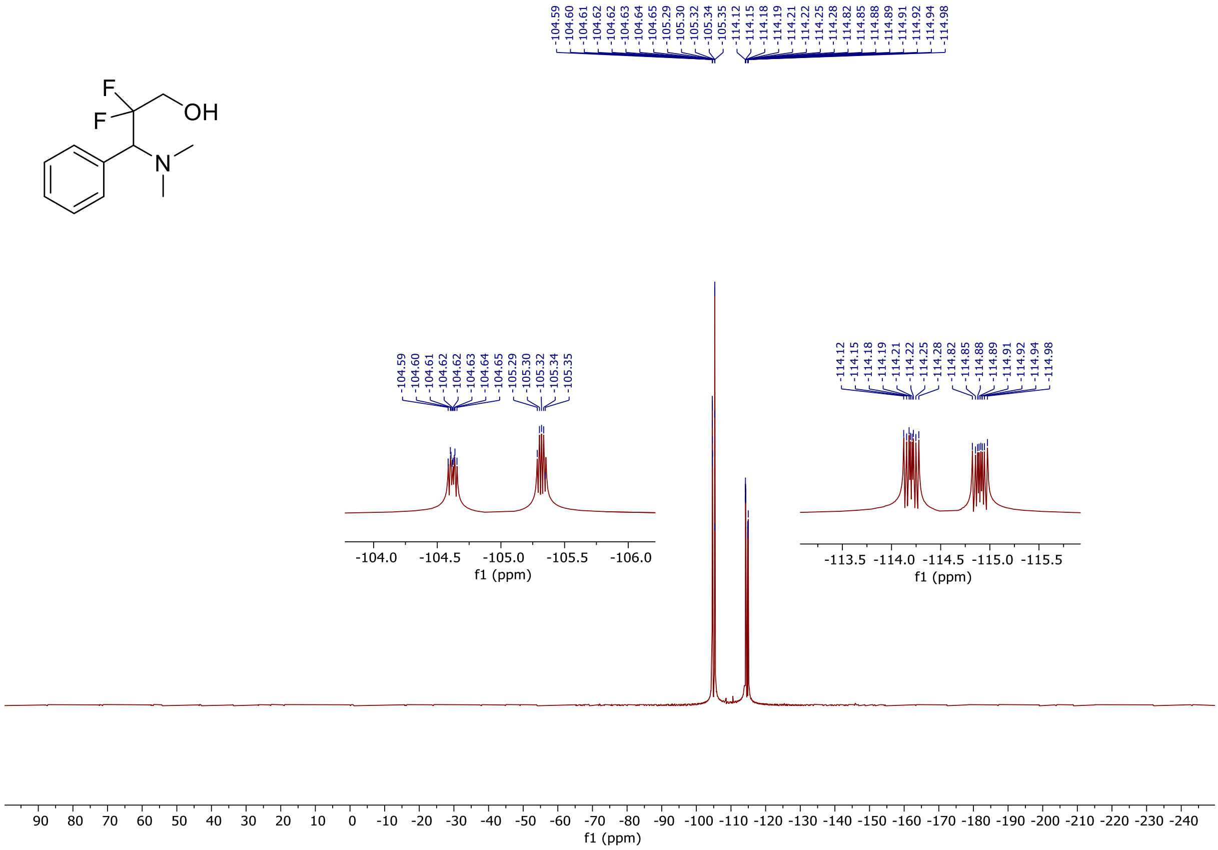

7 – ^{1}H NMR (400 MHz, $CDCl_3$)

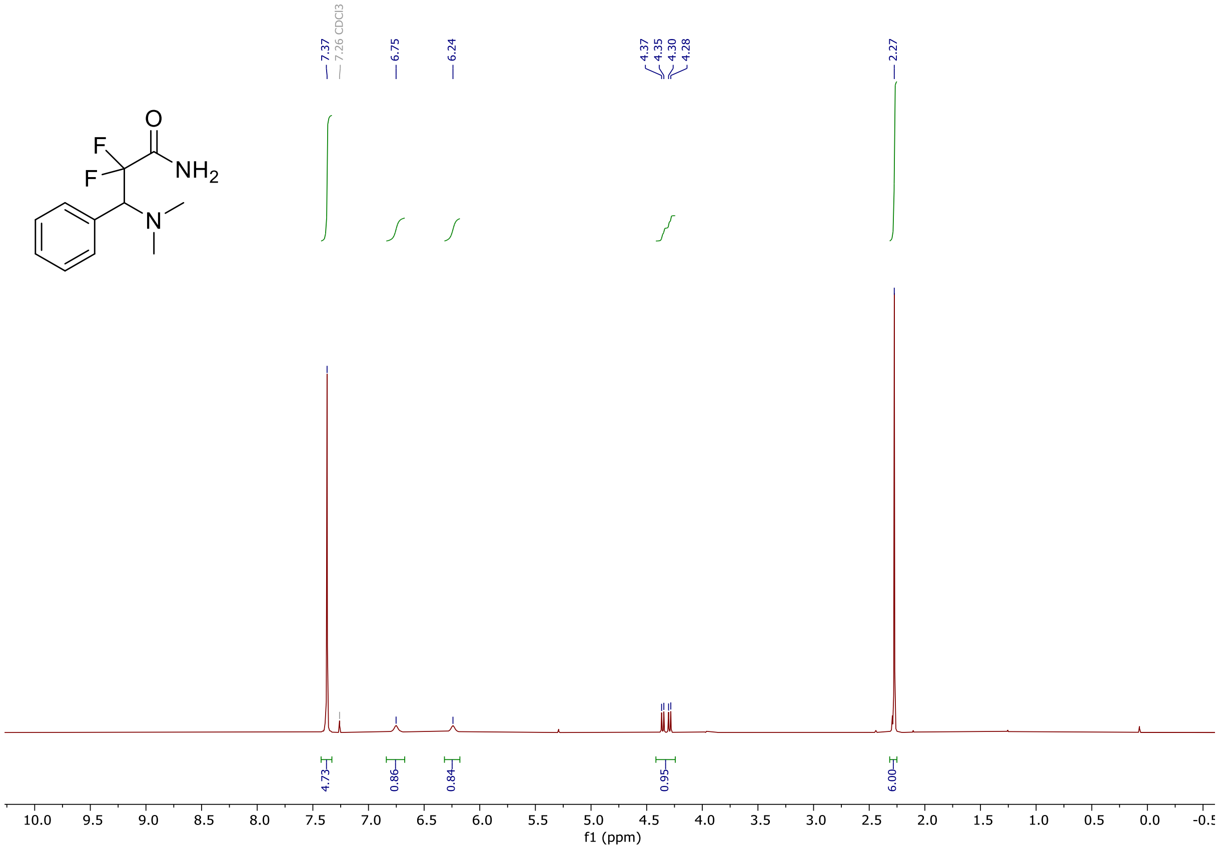

7 – ^{13}C NMR (126 MHz, $CDCl_3$)

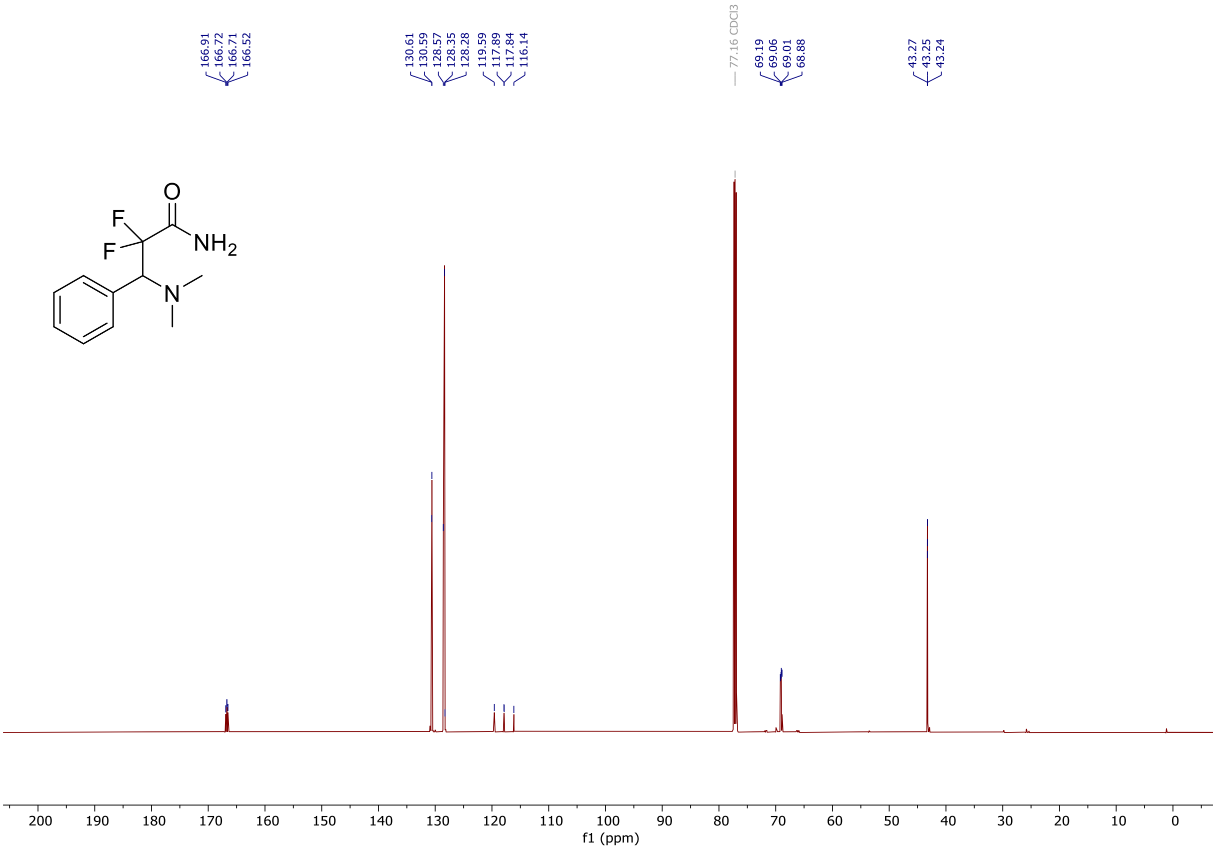

7 – ^{19}F NMR (377 MHz, $CDCl_3$)

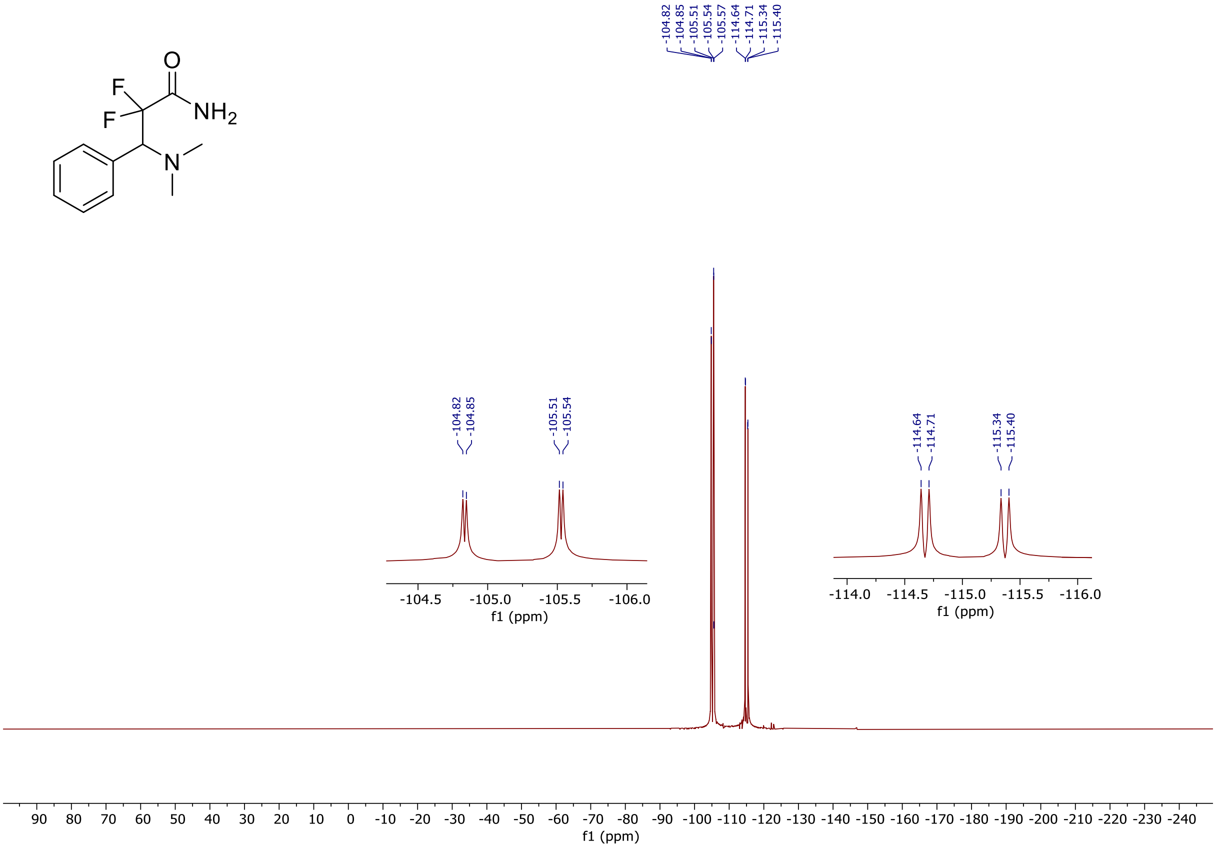

8 – ^{1}H NMR (400 MHz, $CDCl_3$)

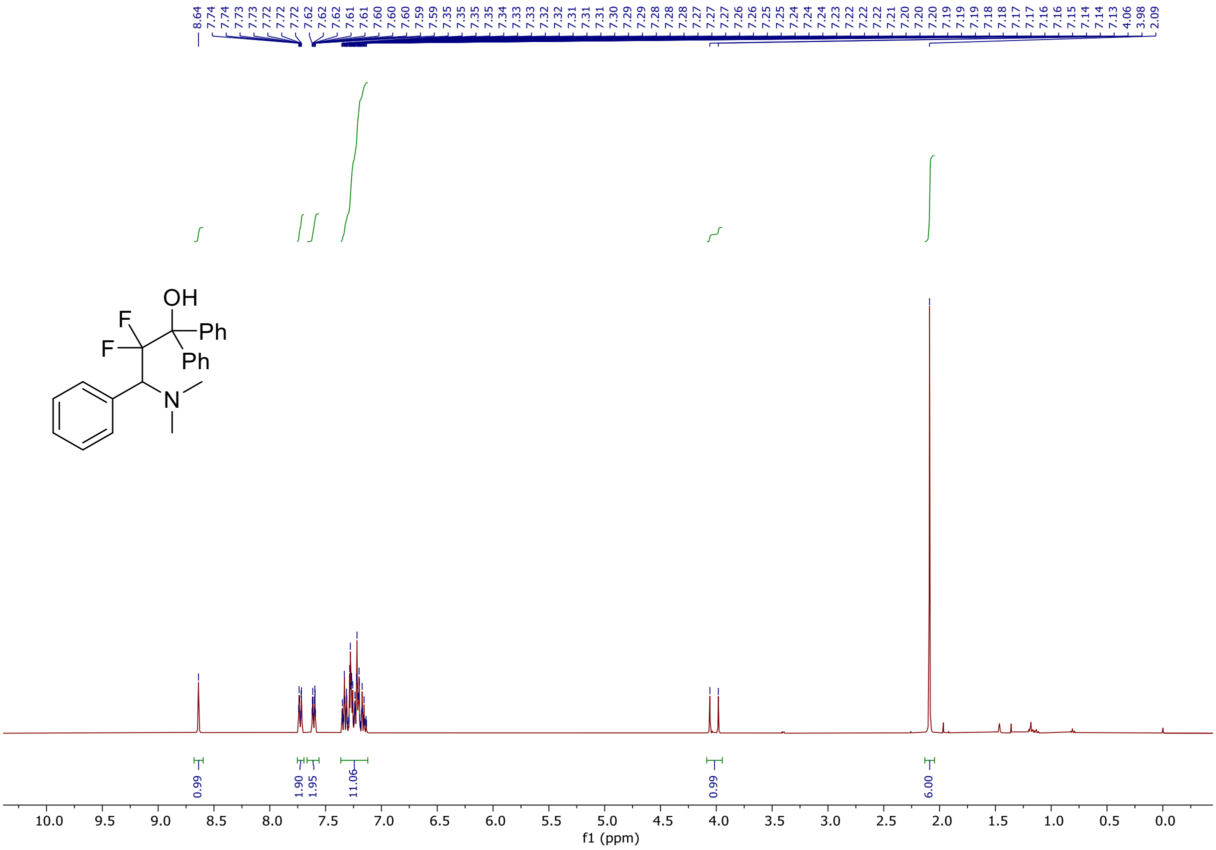

8 – ^{13}C NMR (126 MHz, $CDCl_3$)

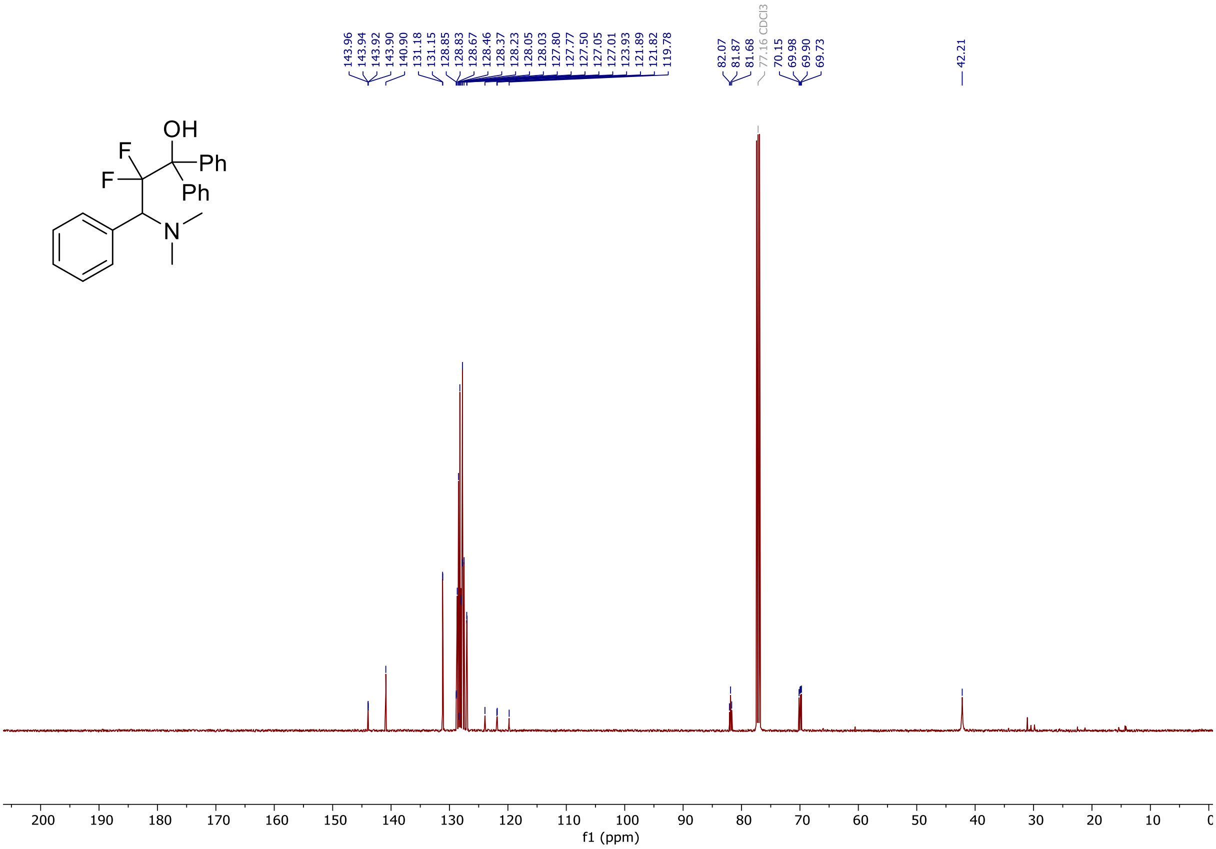

8 – ^{19}F NMR (377 MHz, $CDCl_3$)

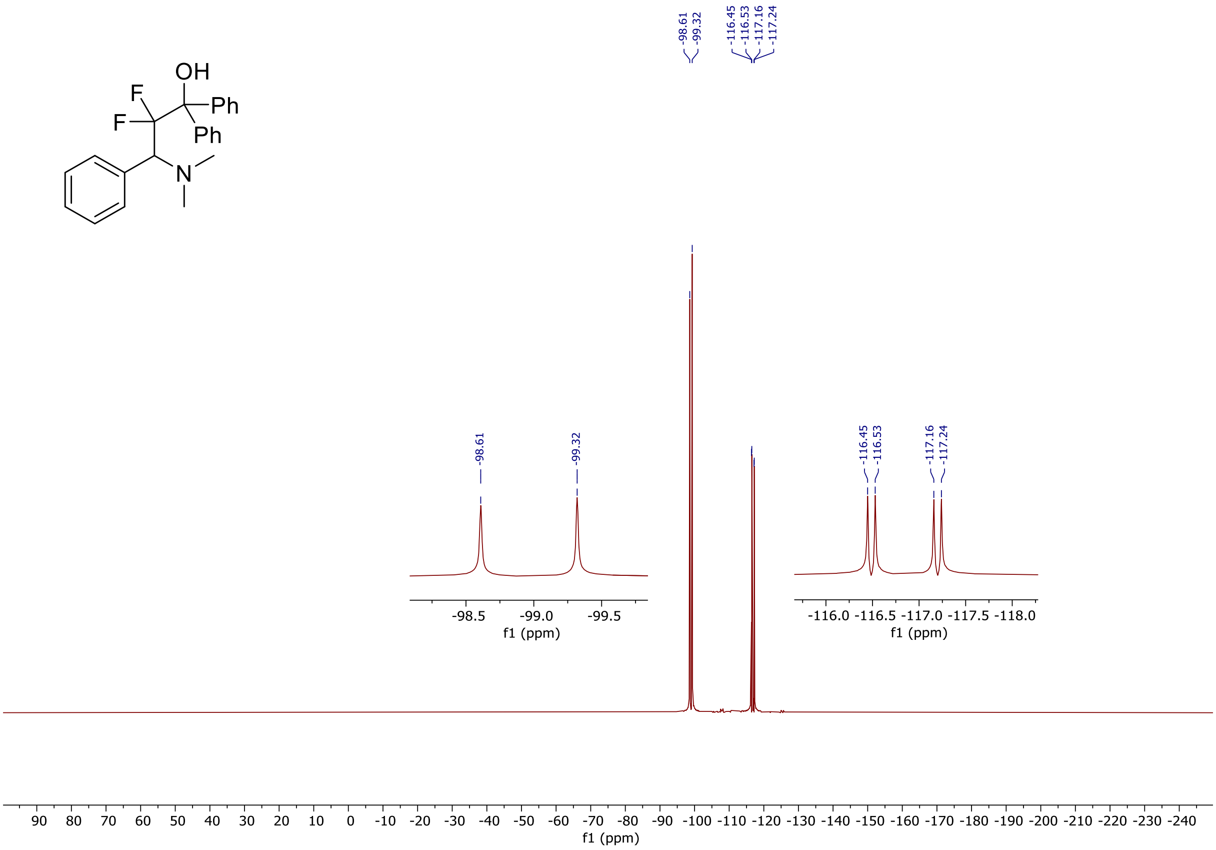

9 – ^{1}H NMR (400 MHz, D_2O)

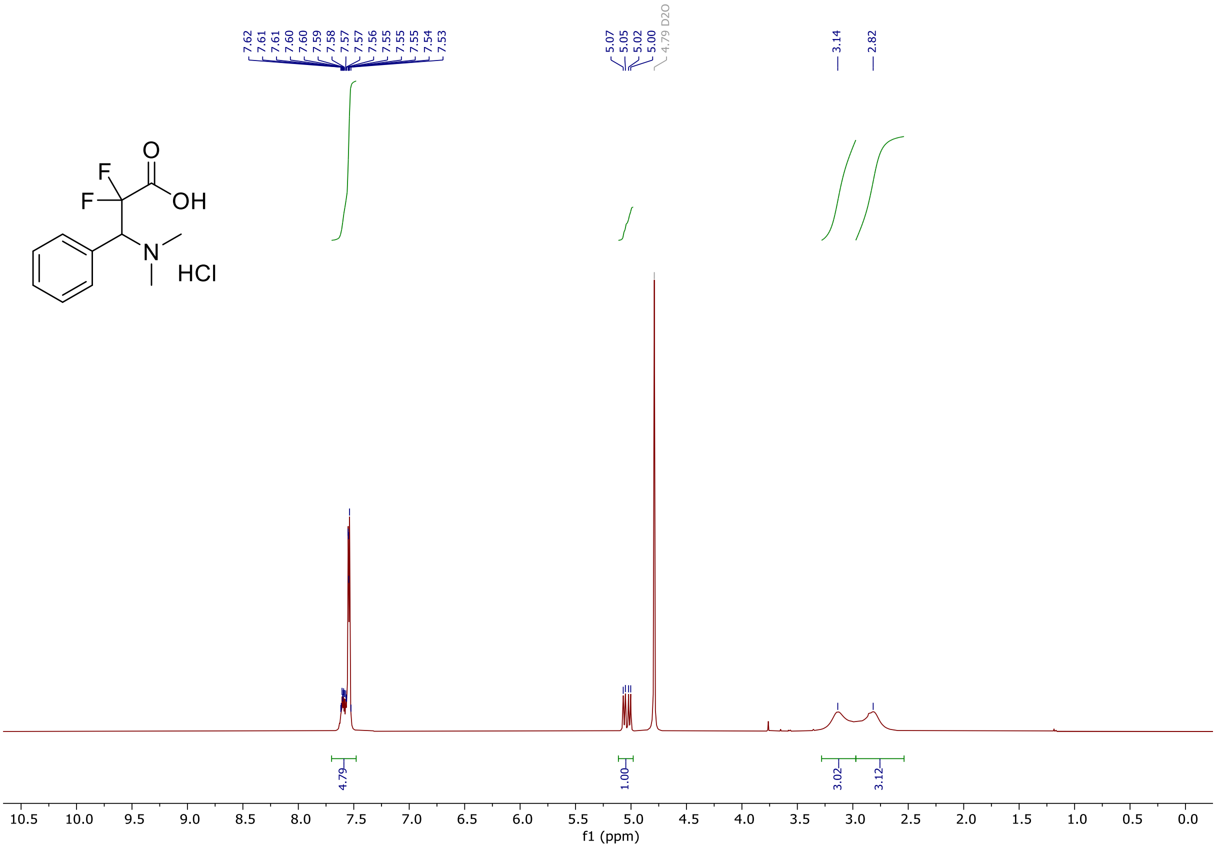

9 – ^{13}C NMR (126 MHz, D_2O)

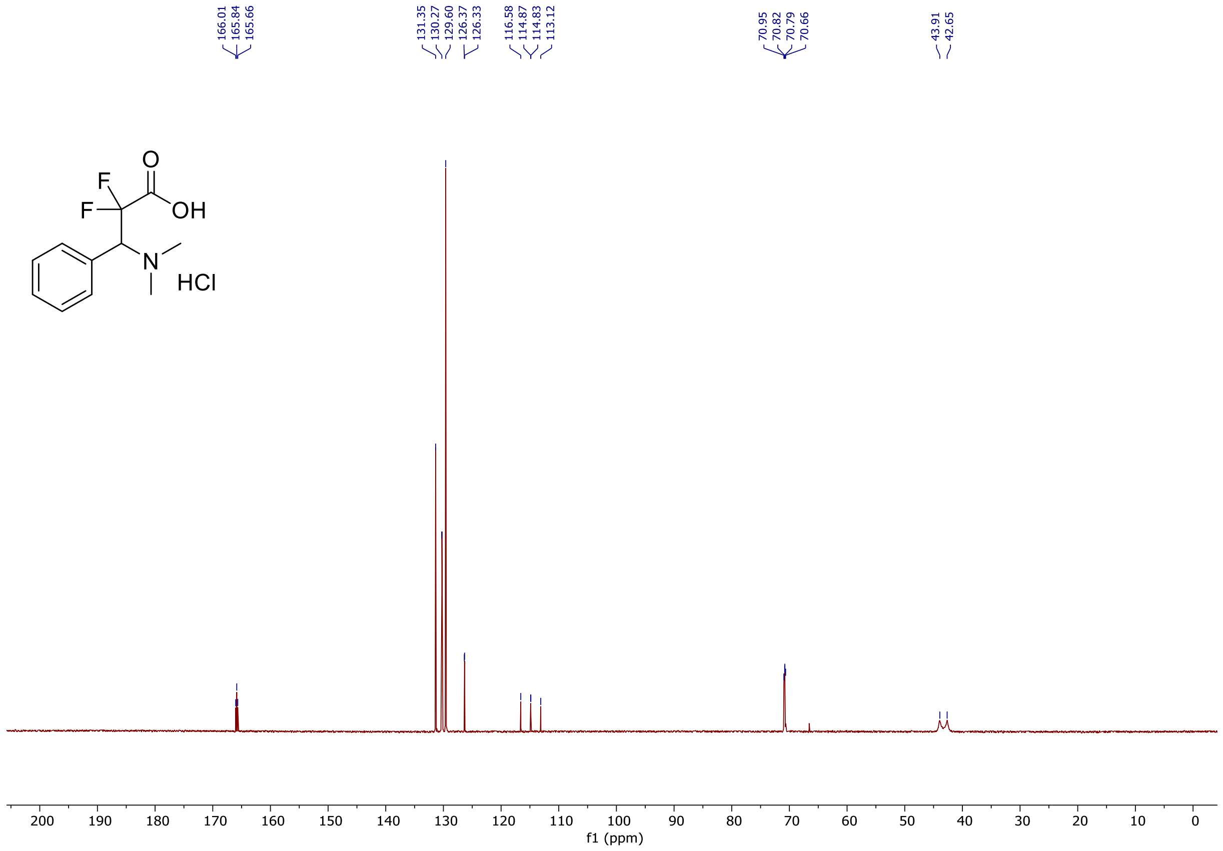

9 – ^{19}F NMR (377 MHz, D_2O)

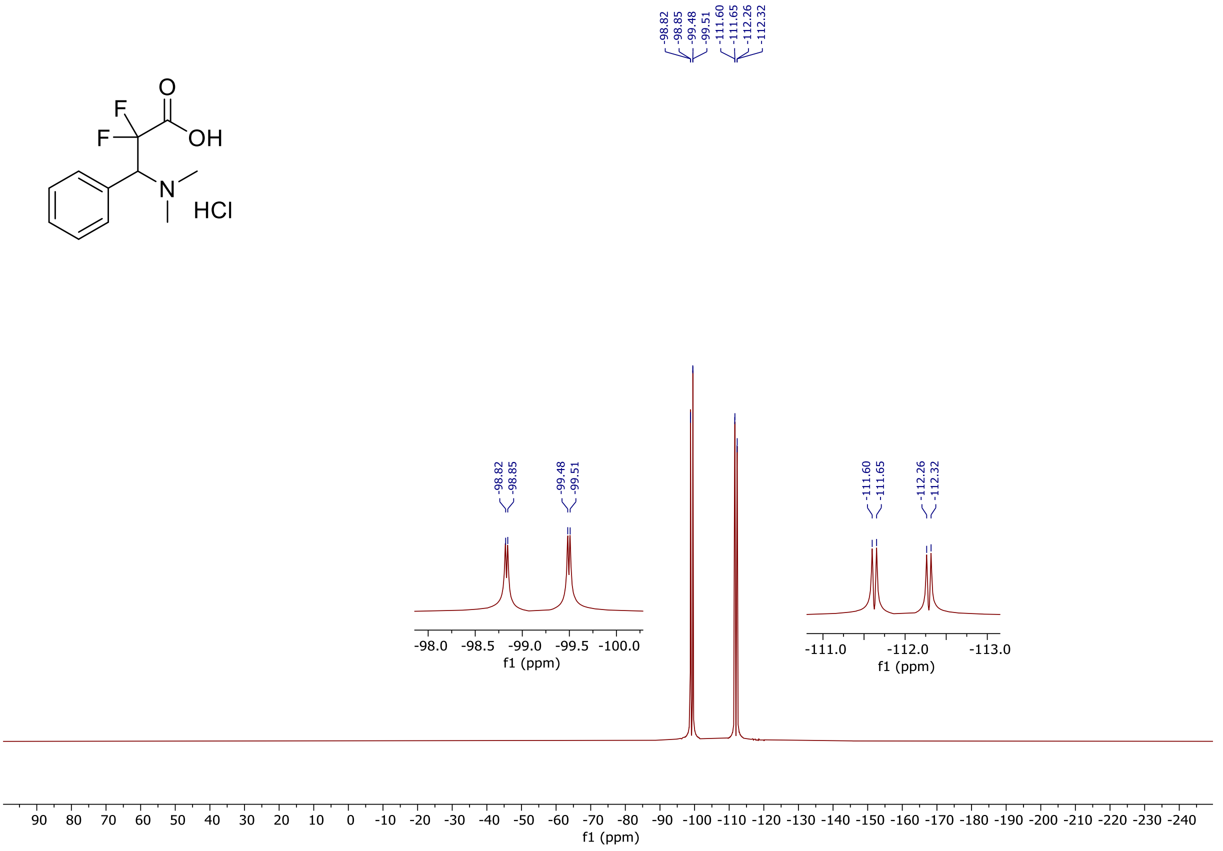

10 – ^{1}H NMR (400 MHz, $CDCl_3$)

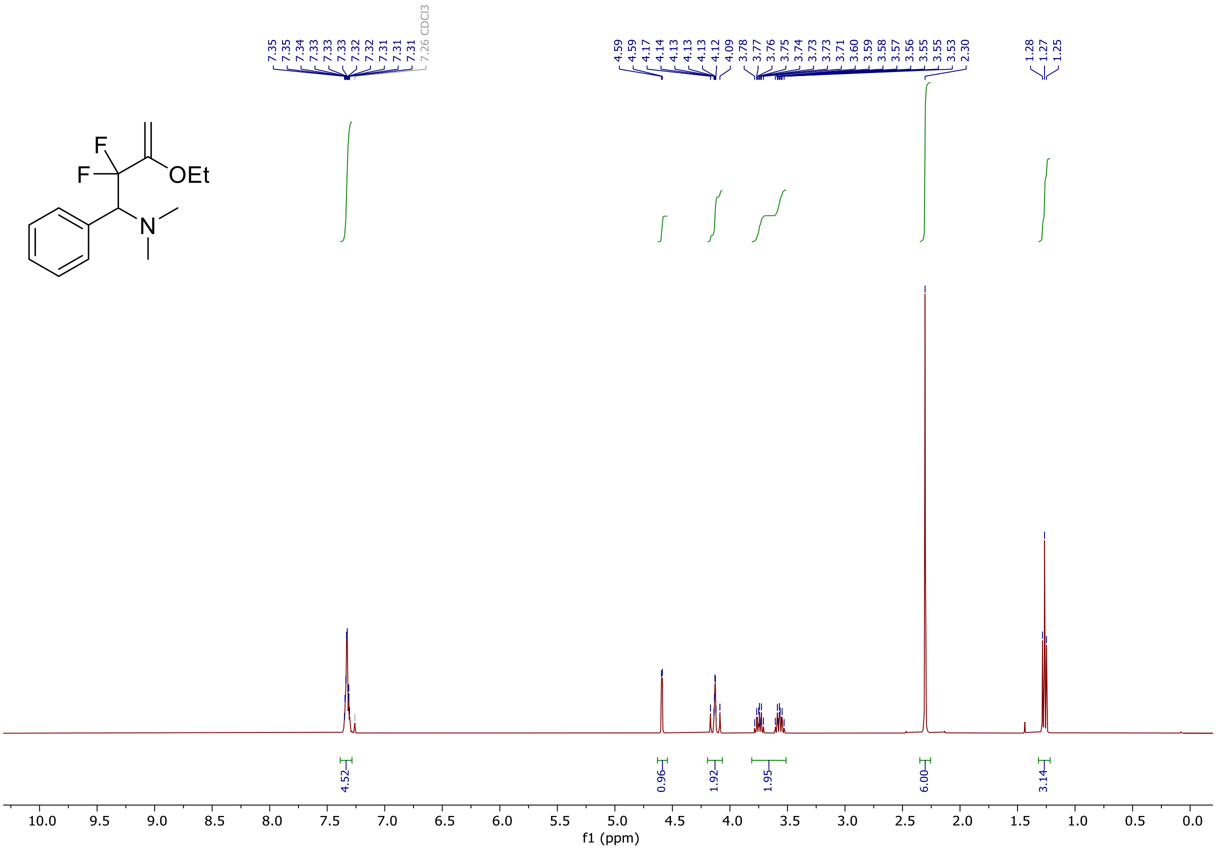

10 – ^{13}C NMR (101 MHz, $CDCl_3$)

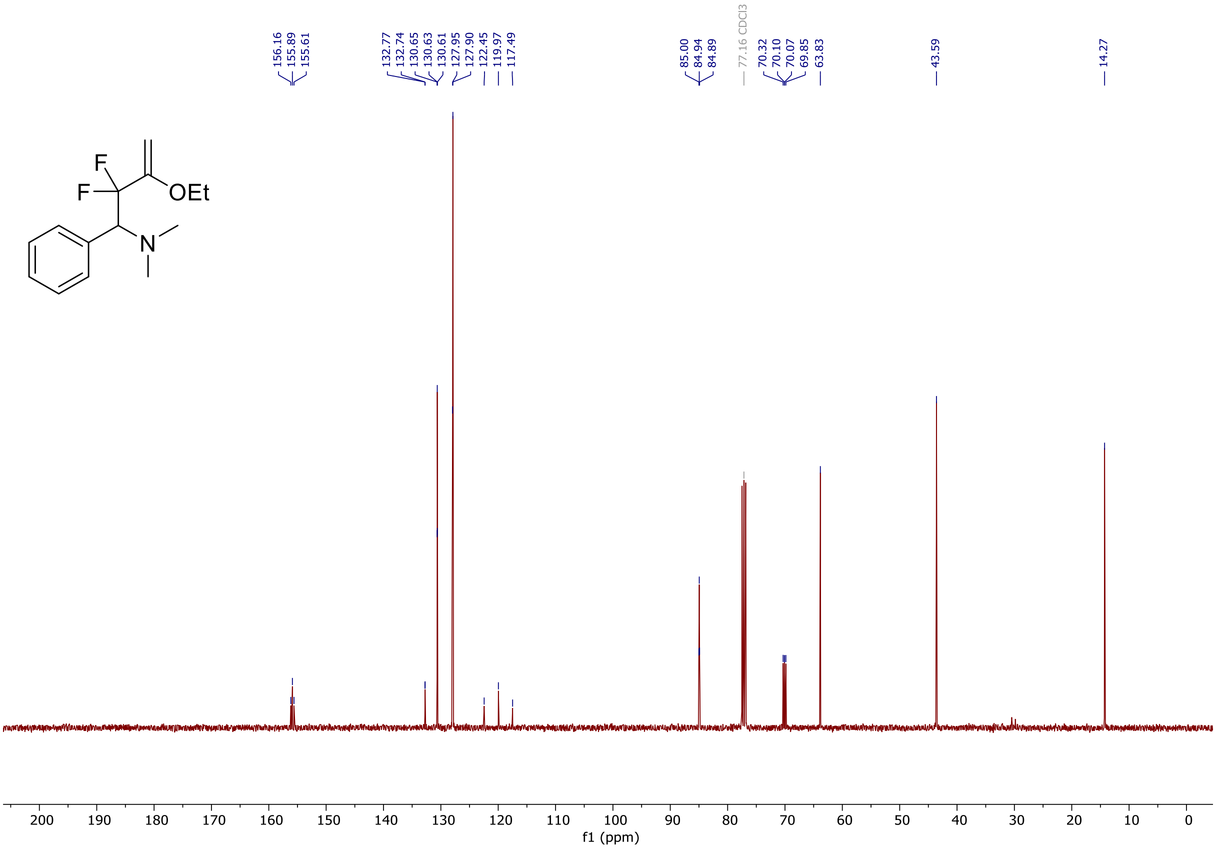

10 – ^{19}F NMR (377 MHz, $CDCl_3$)

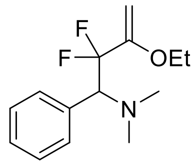

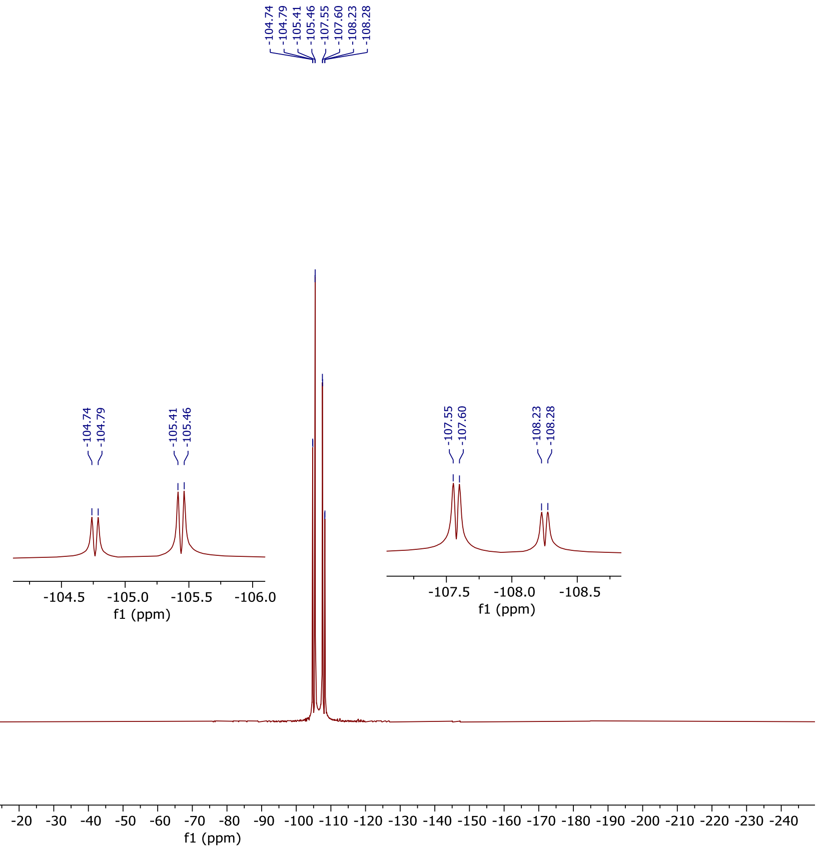

7. References

(1) Fuentes de Arriba A. L.; Lenci, E.; Sonawane, M.; Formery, O.; Dixon, D. J. Iridium-Catalyzed Reductive Strecker Reaction for Late-Stage Amide and Lactam Cyanation. *Angew. Chem. Int. Ed.* **2017**, *56*, 3655-3659.

(2) Leitch, J. A.; Fuentes de Arriba, A. L.; Tan, J.; Hoff, O.; Martinez, C. M.; Dixon, D. J. Photocatalytic reverse polarity Povarov reaction. *Chem. Sci* **2018**, *9*, 6653-6658.

(3) Xie, L.-G.; Dixon, D. J. Iridium-catalyzed reductive Ugi-type reactions of tertiary amides. *Nat Commun* **2018**, ***9*,** 2841, 1-8.

(4) Rogova, T.; Gabriel, P.; Zavitsanou, S.; Leitch, J. A.; Duarte, F.; Dixon, D. J. Reverse Polarity Reductive Functionalization of Tertiary Amides *via* a Dual Iridium-Catalyzed Hydrosilylation and Single Electron Transfer Strategy. *ACS Catal*. **2020**, *10*, 11438-11447.

(5) Ong, D. Y.; Fan, D.; Dixon, D. J.; Chiba, S. Transition-Metal-Free Reductive Functionalization of Tertiary Carboxamides and Lactams for *α*-Branched Amine Synthesis. *Angew. Chem. Int. Ed.* **2020**, *59*, 11903-11907.

(6) Chan, G. H.; Ong, D. Y.; Yen, Z.; Chiba, S. Reduction of *N,N*-Dimethylcarboxamides to Aldehydes by Sodium Hydride–Iodide Composite. *Helv. Chim. Acta* **2018**, *101*, e1800049.

(7) Ghinato, S.; Territo, D.; Maranzana, A.; Capriati, V.; Blangetti, M.; Prandi, C. A Fast and General Route to Ketones from Amides and Organolithium Compounds under Aerobic Conditions: Synthetic and Mechanistic Aspects. *Chem. Eur. J.* **2021**, *27*, 2868-2874.

(8) Otsuka, R.; Maruhashi, K.; Ohwada, T. Latent Brønsted Base Solvent-Assisted Amide Formation from Amines and Acid Chlorides. *Synthesis* **2018,** *50*, 2041-2057.

(9) Bélanger, G.; April, M.; Dauphin, É.; Roy, S. Effect of Substitution on the Intramolecular 1,3-Dipolar Cycloaddition of Alkene Tethered Münchnones. *J. Org. Chem.* **2007**, *72*, 1104-1111.

(10) Gao, T.-T.; Zhang, W.-W.; Sun, X.; Lu, H.-X.; Li, B.-J. Stereodivergent Synthesis through Catalytic Asymmetric Reversed Hydroboration. *J. Am. Chem. Soc.* **2019**, *141*, 4670-4677.

(11) Matheau-Raven, D.; Dixon, D. J. General *α*-Amino 1,3,4-Oxadiazole Synthesis *via* Late-Stage Reductive Functionalization of Tertiary Amides and Lactams. *Angew. Chem. Int. Ed.* **2021**, *60*, 19725-19729.

(12) Andersen, T. L.; Frederiksen, M. W.; Domino, K.; Skrydstrup, T. Direct Access to *α,α*-Difluoroacylated Arenes by Palladium-Catalyzed Carbonylation of (Hetero)Aryl Boronic Acid Derivatives. *Angew.Chem.Int. Ed.* **2016**, *55*, 10396-10400.

(13) Krasovskiy, A.; Knochel, P. Convenient Titration Method for Organometallic Zinc, Magnesium, and Lanthanide-Reagents. *Synthesis* **2006**, *5*, 890-891.

(14) Shi, Y. ; Kamer, P. C. J.; Cole-Hamilton, D. J.; Harvie, M.; Baxter, E. F.; Lim, K. J. C.; Pogorzelec P. A new route to *N*-aromatic heterocycles from the hydrogenation of diesters in the presence of anilines. *Chem. Sci.* **2017**, *8*, 6911-6917.

(15) Du, B.; Chan, C.-M.; Lee, P.-Y.; Cheung, L.-H.; Xu, X.; Lin, Z.; Yu W.-Y. 2,2-Difluorovinyl benzoates for diverse synthesis of *gem*-difluoroenol ethers by Ni-catalyzed cross-coupling reactions. *Nat Commun* **2012**, *12*, 412, 1-10.
